# Efficient One‐Pot Synthesis of Dibenzoxanthenes Using a Vinylimidazolium‐Based Brønsted Acidic Ionic Liquid [VSBIM][Cl]: In Silico Evaluation of Drug‐Likeness, ADMET Properties, PASS Analysis, Antiviral Activity Prediction, and Molecular Docking Studies

**DOI:** 10.1002/open.70253

**Published:** 2026-08-11

**Authors:** Talamarlla Deepthi, Barnali Maiti

**Affiliations:** ^1^ Department of Chemistry, School of Advanced Sciences Vellore Institute of Technology Vellore India

**Keywords:** 1‐vinyl‐3‐(4‐sulfobutyl) imidazolium chloride [VSBIM][Cl], 2‐naphthol, aryl aldehydes, MCR

## Abstract

Dibenzoxanthene derivatives have produced considerable interest due to their pharmacological, photophysical, and organic optoelectronic properties. Herein, a facile one‐pot methodology was developed for the synthesis of 14‐(aryl)‐14H‐dibenzo[a,j]xanthene derivatives employing 5 mol% of the Brønsted acidic ionic liquid 1‐vinyl‐3‐(4‐sulfobutyl) imidazolium chloride [VSBIM][Cl] as an efficient and recyclable catalyst under solvent‐free conditions. This protocol enables the condensation of *β*‐naphthol with varied aryl or heteroaryl aldehydes to afford dibenzoxanthenes under neat conditions at 110°C. Nineteen 14‐(aryl)‐14H‐dibenzo[a,j]xanthene derivatives were prepared in good to high yields. The synthesized dibenzoxanthenes were characterized by ^1^H and ^13^C NMR along with FT‐IR spectroscopy. Furthermore, photophysical properties, drug‐likeness (SwissADME), ADMET, PASS antiviral prediction, and molecular docking against Human Cyclooxygenase‐2 (PDB: 5IKQ) were performed as preliminary in silico studies.

## Graphical abstract

An efficient and solvent‐free sustainable one‐pot protocol is developed for the synthesis of broad range of dibenzoxanthenes using 5 mol% Bronsted acidic ionic liquid [VSBIM][Cl]. The versatility of the methodology was demonstrated by the effective synthesis of 19 dibenzoxanthene derivatives up to 90%–95% yields.

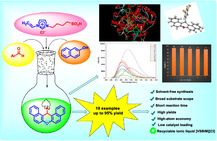

## 1 Introduction

**FIGURE 1:**
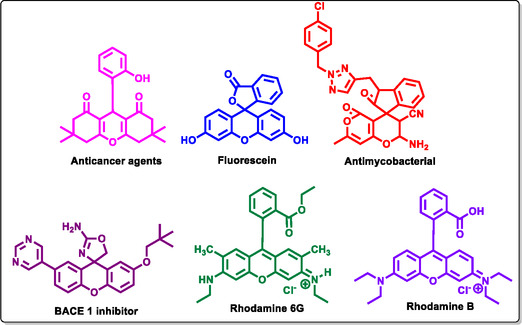
Representative biologically active molecule containing a xanthene core.

The expansion of atom‐economic and environmentally sustainable approaches for the rapid synthesis of complex heterocyclic frameworks continues to be a major goal of modern organic synthesis [1, 2]. Heterocycles bearing oxygen and nitrogen atoms constitute the core of numerous pharmaceuticals, agrochemicals, dyes, and natural products, and therefore developing efficient methods for the synthesis of these structures remains an important area of research [3]. Among such heterocyclic systems, the dibenzo[a,j]pyran (xanthene) framework represents a key structural motif present in biologically and functionally relevant molecules. Owing to their wide‐ranging applications, xanthene derivatives have attracted significant attention in medicinal chemistry, chemical biology, and materials science. Representative functional and bioactive molecules incorporating the xanthene framework are illustrated in Figure 1 [4, 5].

Xanthene‐based compounds are reported to exhibit a widespread range of biological activities [6], including antiviral [7], anti‐inflammatory [8], anti‐bacterial [9], antifungal [10, 11], anticancer [12], and antioxidant activity [13]. In addition, these compounds display attractive photophysical properties including strong fluorescence, excellent stability, and tuneable emission [14]. Because of this unique combination of biological and photophysical features, xanthene derivatives have emerged as multifunctional scaffolds in both medicinal and materials chemistry. Beyond medicinal applications, xanthene and benzo‐xanthene derivatives were widely employed as dyes, fluorescent labels in biological studies, sensitizers in dye‐sensitized solar cells (DSSCs), food‐industry additives, active materials in lasers, organic light‐emitting diode (OLED), and optoelectronic devices[15, 16, 17, 18, 19]. Upon oxidation, many xanthene derivatives form xanthium salts that exhibit intense coloration and strong fluorescence, thereby further expanding their applications as dyes and photophysical materials [20]. Collectively, the structural versatility and multifunctional applications of xanthenes have attracted broad interest across the chemical sciences. Traditional methods for preparing xanthenes usually involve the one pot condensation of aryl/alkyl aldehydes with *β*‐naphthol or reactions of acetophenones with aromatic aldehydes, or the condensation of 2‐tetralone with 2‐hydroxy aromatic aldehydes. Recently, *β*‐naphthol–aldehyde reactions have emerged as an efficient methodology for the synthesis of 14‐aryl‐14H‐dibenzo[a,j]xanthenes with good atom economy and simpler operation.

**SCHEME 1:**
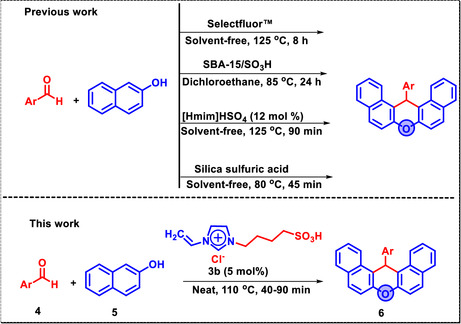
Various synthetic routes to 14‐aryl‐14H‐dibenzo[a,j]xanthene.

Numerous catalysts were explored for this transformation, such as Selectfluor, FeCl_3_, SBA‐15/SO_3_H, [Hmim]HSO_4_, *p*‐toluenesolfonic acid [21], sulfamic acid [22], cellulose sulfuric acid [23], zirconium(iv) oxide chloride [24], CBr_4_ [25], molecular ionine [26], heteropoly acid [27], silica sulfuric acid [28], and Amberlyst‐15 [29], as shown in (Scheme 1). Despite their effectiveness, many of these catalytic systems suffer from several limitation such as harsh reaction conditions, long reaction times, excessive catalyst loading, limited recyclability, or uses of toxic environment. Organic solvents play a major source of environmental hazards due to their high volatility and large‐scale uses in industry. Furthermore, according to the 12 principles of green chemistry, the use of separation agents, auxiliary substances and traditional organic solvents must be avoided wherever possible. Organic reactions carried out in water or under solvent‐free conditions are the need of the hour for accomplishing sustainable and environmentally friendly chemical synthesis. Ionic liquids are considered as eco‐friendly designer solvents because they help make chemical processes cleaner and more sustainable [30, 31, 32]. Ionic liquids have attracted attention in organic synthesis due to their non‐volatile, high thermal chemical stability, wide liquid ranges and high ionic conductivity [33]. Consequently, they have fascinated significant consideration in nanoparticle synthesis, electrochemistry, catalysis, biocatalysis and separations [34]. By suitable modification of the anion and organic unsymmetrical cation, the physicochemical properties of ionic liquids can be tuned, facilitating their separation from organic or aqueous phases [35, 36, 37]. When acidic functional groups are incorporated into the ionic liquid framework, these designer liquids can function as efficient acidic catalyst as Brønsted acids [38, 39]. Task‐specific ionic liquids are more compatible in numerous organic reactions over mineral acids and are therefore considered potential alternatives to conventional mineral acids such as sulfuric acid and hydrochloric acid [40]. Notably, such acidic task‐specific ionic liquids can function simultaneously both as solvent and catalyst, further enhancing process efficiency [41, 42, 43]. Although numerous ionic liquid catalysts have been reported, there is still a need for more efficient and recyclable catalytic systems for the synthesis of dibenzoxanthene derivatives [44, 45]. One‐pot multicomponent condensation reactions have been widely recognized as efficient strategies for creating molecular complexity in a solitary synthetic step and are therefore highly significant in organic and medicinal chemistry. Therefore, to discourse the above‐mentioned limitations and in continuance of our ongoing research interest in the expansion of highly atom‐efficient, one‐pot sustainable reaction protocol was developed for the synthesis of 14‐aryl‐14H‐dibenzo[a,j]xanthene as shown in (Scheme 1). Herein, we report a Brønsted acidic ionic liquid (BAIL) catalyzed solvent‐free, one‐pot green synthesis of 14‐aryl‐14H‐dibenzo[a,j]xanthene derivatives via the condensation of substituted aryl aldehydes with 2‐naphthol under solvent‐free conditions.

## 2 Results and Discussion

### 2.1 Catalyst Preparation

**SCHEME 2:**
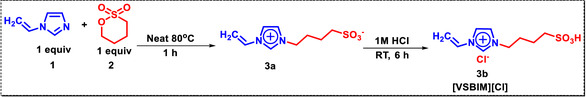
Synthesis of Brønsted acidic ionic liquid ([VSBIM][Cl]).

Task‐specific Ionic liquids (ILs) have become progressively popular as environmentally friendly catalyst as they can be custom‐designed to suit precise reaction conditions. In effort to develop an effective and recyclable catalyst for the synthesis of dibenzoxanthene derivatives, a sulfonic acid‐functionalized vinyl imidazolium ionic liquid was synthesized. The Brønsted acidic —SO_3_H group was incorporated into a vinylimidazolium framework to make a highly efficient ionic liquid catalyst. The synthesis of the Brønsted acidic ionic liquid 1‐vinyl‐3‐(4‐sulfobutyl) imidazolium chloride [VSBIM][Cl] was carried out according to a modified literature procedure (Scheme 2).

A mixture of same equivalent 1‐vinylimidazole **1** and 1,4‐butanesultone **2** was heated under neat conditions at 80°C for 1 h, affording zwitterionic intermediate **3a** as a white solid. After chilling to room temperature, the crude derivatives were washed with ethyl acetate and dried under high vacuum.

The synthesized zwitterion **3a** was then treated with aqueous 1 M HCl at room temperature and stirred for 6 h, to obtain a viscous liquid **3b**. The resultant reaction mixture was washed successively with ethyl acetate and hexane and dried under high vacuum to afford [VSBIM][Cl] **3b** as a viscous liquid. The product was characterized by ^1^H NMR, ^13^C NMR, and FT‐IR spectroscopy. Next, we commence our study by choosing benzaldehyde **(4a)** and 2‐naphthol **(5)** as model substrates in the presence of 5 mol% of ionic liquid catalyst **([VSBIM][Cl] 3b)** (Table 1). The reaction was performed in a variety of solvents under reflux conditions. In polar aprotic solvents such as acetonitrile (CH_3_CN), dimethylformamide (DMF), tetrahydrofuran (THF), chloroform (CHCl_3_), and dichloromethane (DCM), the desired product **6a** was obtained in moderate yields ranging from 30% to 45% (Table 1, entries 1–5). In contrast, isopropyl alcohol (IPA) afforded a slightly higher yield of 60% (Table 1, entry 6).

**TABLE 1:** Solvent‐optimization for the synthesis of **6a**.^a^

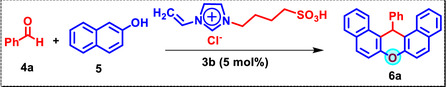				
Entry	Solvent	Temperature, °C	Time, h	Yield,^b^ %
1	CH_3_CN	Reflux	12	37
2	DMF	Reflux	12	40
3	THF	Reflux	12	32
4	CHCl_3_	Reflux	12	30
5	DCM	Reflux	12	45
6	IPA	Reflux	12	60
7	MeOH	Reflux	12	75
8	EtOH	Reflux	12	75
9	H_2_O	Reflux	12	80
10	(EtOH‐H_2_O, 1:1 v/v)	Reflux	12	85
**11** a	**Solvent‐free**	**110**°**C**	**0.66**	**95**

*Note*: Bold value indicates best optimization conditon.

a Reaction condition: 4a (1 equiv, 1 mmol), 5 (2 equiv, 2 mmol), at reflux condition, 5 mol% catalyst (3b).

b Yield of the isolated product.

Significant improvements were observed in polar protic solvents with methanol, ethanol, and water affording **6a** in 75%–80% yields (Table 1, entries 7–9). Interestingly, aqueous ethanol (EtOH‐H_2_O, 1:1 v/v) gave the highest solvent‐based yield of 85% (Table 1, entry 10). Notably, under solvent‐free conditions at 110°C, the reaction proceeded efficiently to furnish **6a** in 95% yield in 40 min (Table 1, entry 11). Based on these results, solvent‐free conditions were identified as optimal reaction condition and were therefore employed for subsequent catalyst optimization studies (Table 1).


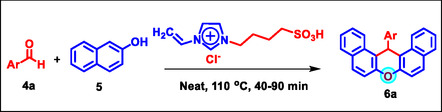


Reaction condition: Aryl aldehydes 4a (1 equiv, 1 mmol), 2‐napthol 5 (2 equiv, 2 mmol), at neat condition 110°C, 5 mol% ionic liquid catalyst.

With the optimized reaction conditions, we next evaluated the effect of different acidic catalysts and catalyst loadings mol percentage on the synthesis of xanthene derivative **6a** (Table 2). When the condensation reaction of benzaldehyde (**4a**, 1 mmol) with 2‐naphthol (**5**, 2 mmol) was carried out without any catalyst, the reaction proceeded sluggishly, affording only 10% conversion (Table 2, entry 1). In the presence of conventional Brønsted acids such as *p*‐TSA, H_2_SO_4_, and CH_3_COOH, the reaction furnished **6a** in moderate yields of 55%–62% yield (Table 2, entries 2–4). In contrast, the use of the Brønsted acidic ionic liquid as a catalyst BAILs (**3b**) under neat conditions significantly enhanced the efficiency. With 2 mol% of IL **3b**, **6a** was obtained in 65% yield after 15 min.

**TABLE 2:** Optimization of catalysts.^a^

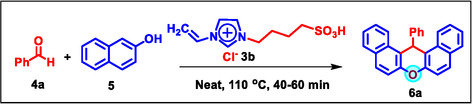				
Entry	Catalyst	Mol, %	Time, h	Yield,^b^ %
1	No catalyst	—	—	10
2	*p*‐TSA	20	28	55
3	H_2_SO_4_	20	8	62
4	CH_3_COOH	20	8	60
5	[VSBIM][Cl]	2	0.25	65
6	[VSBIM][Cl]	4	0.5	80
**7**	**[VSBIM][Cl]**	**5**	**0.66**	**95**
8	[VSBIM][Cl]	7	0.83	95
9	[VSBIM][Cl]	8	1	95

*Note*: Bold value indicates best optimization conditon.

a Reaction condition: **4a** (1 equiv, 1 mmol), **5** (2 equiv, 2 mmol), at neat condition 110°C, 5 mol% catalyst.

b Yield of the isolated product.

**FIGURE 2:**
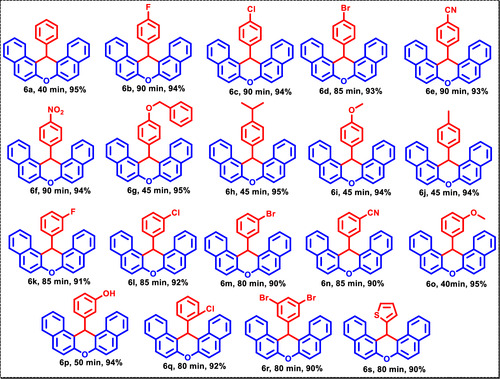
Substituted 14‐phenyl‐14H‐dibenzo[a,j]xanthene derivatives.

Increasing the catalyst loading to 4 mol% improved the yield to 80% in 30 min, while 5 mol% catalyst afforded **6a** in 95% yield in 40 min (Table 2, entries 7). Notably, further increasing the catalyst concentration beyond 5 mol% had no noteworthy effect on either yield or reaction time, indicating that 5 mol% is the optimal loading (Table 2). Therefore, a catalyst loading of 5 mol% was selected as the optimal amount for all subsequent condensation reactions. With optimized reaction conditions in hand, a broad range of substituted aromatic aldehydes were explored for the condensation reaction. The condensation reaction of 2‐naphthol (2 mmol) with various substituted aromatic aldehydes (1 mmol) was performed in neat conditions at 110°C for 40–90 min (Figure 2). Under these conditions, the desired 14‐aryl‐14H‐dibenzo[a,j]xanthenes (**6a**‐**6s**) were obtained in consistently excellent yields (90%–95%). Aryl aldehydes bearing with electron‐withdrawing groups (EWGs) such as —F, —Br, —Cl, —CN, and —NO_2_ (Table 3, entries **6a**–**6f**) afforded the corresponding xanthene products in slightly lower yields (93%–94%) compared to those with electron‐donating groups (EDGs). Notably, aromatic aldehydes with EDGs exhibited a higher reactivity with good to outstanding yields (94%–95%) (Table 3, entries **6g**–**6j**). Moreover, meta‐substituted aromatic aldehydes with (EWGs) provided lower yields (Table 3, entries **6k**–**6n**). Furthermore, thiophene‐4‐carbaldehyde, as a representative heterocyclic aldehyde, was well tolerated under neat conditions, delivering the corresponding product in excellent yield (Table 3, entry **6s**). Additionally, disubstituted aromatic aldehydes afforded significant yields (Table 3, entry **6r**).

**TABLE 3:** Substrate scope for 14‐aryl‐14H‐dibenzo[a,j]xanthenes **6a–6s**.^a^

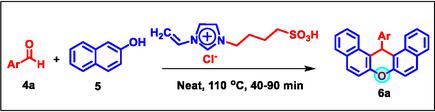						
Entry	Substituent, Ar	Product	Time, h	Yield,^b^ %	Melting point, °C	
					Determined	Reported
1	C_6_H_5_CHO	**6a**	0.66	95	182–190	183–185 [46]
2	4‐F‐C_6_H_4_	**6b**	1.5	94	240–250	236–238
3	4‐Cl‐C_6_H_4_	**6c**	1.5	94	296–298	289–291
4	4‐Br‐C_6_H_4_	**6d**	1.4	93	289–293	298–300
5	4‐CN‐C_6_H_4_	**6e**	1.5	93	305–310	293–295 [47]
6	4‐NO_2_‐C_6_H_4_	**6f**	1.5	94	305–309	310–311
7	4‐C_14_H_10_O_2_	**6g**	0.75	95	298–300	310–315 [48]
8	4‐[(CH_3_)_2_C]C_6_H_4_	**6h**	0.75	95	295–298	301–302 [49]
9	4‐OCH_3_‐C_6_H_4_	**6i**	0.75	94	220–225	204–207
10	4‐CH_3_‐C_6_H_4_	**6j**	0.75	94	215–220	225–228
11	3‐F‐C_6_H_4_	**6k**	1.4	91	240–249	258–260 [50]
12	3‐Cl‐C_6_H_4_	**6l**	1.4	92	189–191	207–209
13	3‐Br‐C_6_H_4_	**6m**	1.3	90	185–190	190–192 [51]
14	3‐CN‐C_6_H_4_	**6n**	1.4	90	189–195	New derivative
15	3‐OCH_3‐_C_6_H_4_	**6o**	1.5	95	187–190	190–191 [52]
16	3‐OH‐C_6_H_4_	**6p**	0.8	94	198–205	210–230
17	2‐Cl‐C_6_H_4_	**6q**	1.3	92	175–180	180–181 [53]
18	3,5‐Br‐C_6_H_3_	**6r**	1.3	90	300–305	New derivative
19	C_5_H_4_OS	**6s**	1.3	90	232–235	245–250 [54]

a Reaction condition: 4 (1 equiv, 1 mmol), 5 (2 equiv), at neat condition 110°C, 5 mol% catalyst.

b Yield of the isolated product.

### 2.2 Plausible Reaction Mechanism

**FIGURE 3:**
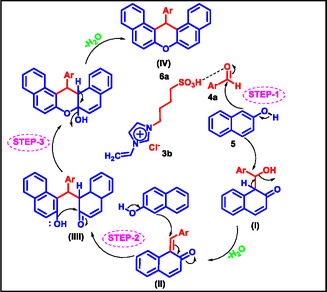
Plausible reaction mechanism.

Based on experimental results, a plausible reaction mechanism was proposed (Figure 3) [55, 56]. A viable and acceptable mechanism for the synthesis of 14‐phenyl‐14*H*‐dibenzo[a,j]xanthene is almost identical and commonly includes a Knoevenagel condensation, Michael addition, and a cyclocondensation reaction (Figure 3). The benzaldehyde (**4a**) is first activated by ionic liquid [VSBIM][Cl] **3b** catalyst. Then, 2‐naphthol (**5**), acting as a nucleophile, attacks the carbonyl group of the activated aryl aldehyde (**4a**) to form intermediate **I**. Subsequent dehydration (loss of H_2_O) leads to the formation of the Knoevenagel product, intermediate **II**. A nucleophilic attack by the second 2‐naphthol on the β‐carbonyl of the α,β‐unsaturated carbonyl group in intermediate **II** leads to the generation of intermediate **III** by Michael addition. Wherein intermediate **III** undergoes a cyclocondensation and dehydration to produce the final desired product, dibenzoxanthene **IV.** Further studies were carried out to understand the pharmaceutical and functional relevance of the synthesized xanthenes. Since xanthenes often exhibit biological and photophysical properties, we evaluated the drug‐likeness (SwissADME), pharmacokinetics (ADMET), probable biological activities (PASS), antiviral potential, and binding affinity (molecular docking) of the compounds. Photophysical studies (UV–vis and fluorescence) were also performed due to the well‐known optical behavior of xanthene scaffolds. These collective evaluations help determine whether the synthesized molecules are suitable as drug‐like candidates or functional materials. Full experimental details, computational parameters, and complete tables of SwissADME, ADMET, PASS, and docking results are provided in the Supporting Information.

### 2.3 Drug Likeness Using swissADME

The drug‐likeness properties of the synthesized compounds (**6a**–**6s**) were evaluated using the SwissADME online tool. The parameters examined included hydrogen‐bond acceptors (HBA), hydrogen‐bond donors (HBD), topological polar surface area (TPSA), cytochrome P450 (CYP) enzyme inhibition profile, and PAINS (Pan‐Assay Interference Compounds) alerts. The HBA values ranged from 1 to 3, while the HBD values ranged from 0 to 1, which are within the limits of Lipinski's Rule of Five [57, 58]. The TPSA values of the molecules (9.23–55.05 Å^2^) were below the commonly reported threshold value of 140 Å [59]. The CYP inhibition profile indicated that most compounds were not predicted to inhibit the major metabolic enzymes CYP2C9, CYP2D6, and CYP3A4, although a few derivatives showed possible inhibition of CYP1A2 and CYP2C19. In addition, all compounds showed zero PAINS alerts, indicating the absence of structural features commonly associated with false‐positive biological activity. Overall, the SwissADME analysis provided preliminary information regarding the physicochemical and drug‐likeness properties of the synthesized dibenzoxanthene derivatives. However, these results should be considered only as an initial computational screening and require further experimental validation. The detailed SwissADME drug‐likeness parameters are provided in Table S2 in the supporting Information.

### 2.4 Assessment of Absorption, Distribution, Metabolism, Excretion, and Toxicity (ADMET) Properties

The ADMET properties of the synthesized compounds (**6a**–**6s**) were evaluated using the pkCSM online tool to obtain preliminary information regarding their absorption, distribution, metabolism, excretion, and toxicity profiles [60, 61, 62]. The predicted results indicated acceptable absorption characteristics and varying levels of blood–brain barrier permeability among the compounds. Most derivatives showed limited interaction with major drug transporters and were predicted to be CYP3A4 substrates without inhibiting this enzyme. None of the compounds was predicted to exhibit hERG inhibition. The estimated LD_50_ values provided an initial indication of their toxicity profiles. Overall, the ADMET study offered preliminary information on the pharmacokinetic and toxicity‐related properties of the synthesized dibenzoxanthene derivatives. However, these findings are based on computational predictions and require further experimental validation. Detailed ADMET prediction data for all compounds (**6a**–**6s**) are provided in Table S2 of the Supporting Information.

### 2.5 Prediction of Activity Spectra for Substances (PASS)

The probable biological activities of the synthesized compounds (**6a**–**6s**) were evaluated using the PASS (Prediction of Activity Spectra for Substances) online tool. The Pa (probability of activity) and Pi (probability of inactivity) values were used to estimate the likelihood of different biological activities. Compounds with Pa values greater than 0.5 were considered more likely to exhibit the predicted activity [63]. The PASS analysis suggested that the synthesized dibenzoxanthene derivatives may possess various biological activities. However, these results are based on computational predictions and should be regarded only as preliminary indications of potential biological properties. Further experimental studies are necessary to confirm the predicted activities. The detailed PASS prediction results, including Pa and Pi values, are presented in Table S3 of the Supporting Information.

### 2.6 Antiviral Activities Evaluation

The antiviral activity of compounds **6a**–**6s** was evaluated using the AVCpred web server. Predicted inhibition values were obtained for general antiviral activity as well as against HBV, HCV, HHV, and HIV. The results showed variation in the predicted antiviral profiles of the synthesized compounds, with some derivatives exhibiting comparatively higher inhibition values against specific viral targets. These observations suggest possible differences in the antiviral behavior of the compounds. However, it should be noted that the AVCpred results are based entirely on computational models and do not represent experimental evidence of antiviral activity. Therefore, the predicted activities should be considered only as preliminary indications and require further biological evaluation for confirmation. The detailed antiviral prediction data for all compounds (**6a**–**6s**) are presented in Table S4 of the Supporting Information.

### 2.7 Molecular Docking

Molecular docking studies were carried out to investigate the possible interactions of the synthesized compounds (**6a**–**6s**) with the selected protein target (PDB ID: 5IKQ). Docking calculations were performed using PyRx 0.8, and the protein–ligand interactions were analyzed using Discovery Studio Visualizer and PyMOL. Prior to docking, the protein was prepared by removing water molecules, adding polar hydrogens, and assigning appropriate charges, while the ligand structures were energy‐minimized. The calculated binding energies ranged from −8.0 to −10.1 kcal/mol. Among the synthesized compounds, **6g** (−10.1 kcal/mol), **6n** (−9.7 kcal/mol), and **6l** (−9.3 kcal/mol) showed the lowest predicted binding energy values. For comparison, the reference COX‐2 inhibitors celecoxib and rofecoxib exhibited binding energies of −9.1 and −6.7 kcal/mol, respectively, under the same docking conditions. Under the docking conditions employed, compounds **6g**, **6n**, and **6l** showed lower predicted binding energy values than celecoxib. Analysis of the docked complexes indicated the presence of non‐covalent interactions such as hydrogen bonding, π–π interactions, π–alkyl interactions, and van der Waals contacts, which may contribute to protein–ligand stabilization. The docking results provide preliminary information regarding the interaction of the synthesized compounds with the selected protein target. However, molecular docking is only a computational approach, and the predicted binding affinities require further experimental validation. The detailed docking scores and interaction analyses for all compounds (**6a**–**6s**) are provided in Table S5 of the Supporting Information.

### 2.8 Photophysical Studies

**FIGURE 4:**
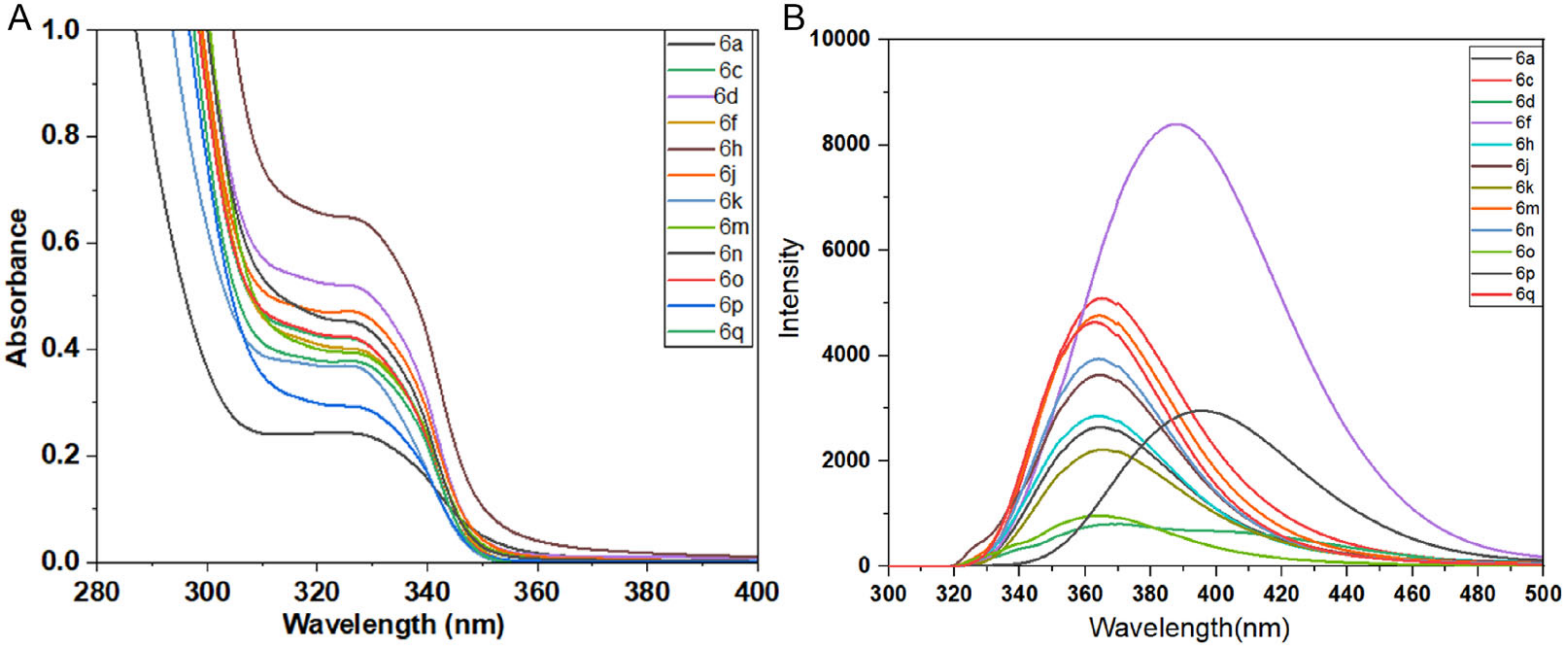
(A) UV–vis and (B) fluorescence spectra of selected xanthene derivatives (40 µM in DMSO) [32, 37, 38].

All compounds showed a characteristic absorption band in the range of 327–333 nm, corresponding to the π–π* transition of the dibenzoxanthene core. The UV–vis absorption and fluorescence spectra of the representative compounds are shown in Figure 4. The close similarity in the absorption maxima suggests that the various substituents have only a limited influence on the ground‐state electronic structure of the molecules. The fluorescence spectra exhibited emission maxima between 363 and 397 nm, which appeared at longer wavelengths than the corresponding absorption bands, consistent with normal Stokes’ shift behavior. Both electron‐donating and electron‐withdrawing groups produced noticeable changes in the emission wavelength and Stokes’ shift values, reflecting their influence on the excited‐state electronic properties of the molecules. Variations in emission intensity were also observed among the derivatives. All photophysical data, including *λ*_abs_, *λ*_em_, molar extinction coefficients, and Stokes’ shift values, are summarized in Table 4.

**TABLE 4:** Experimental photophysical data of selected 14‐aryl‐14H‐dibenzo[a,j]xanthenes.

Entry	Compound	**Absorption** ***λ***_**max,abs**_ **, nm**	**Emission** ***λ***_**max,emi**_ **, nm**	**Molar extinction coefficient × 10** ^4^ **(*ε*)** π–π*	**Stoke's shift** **Δ** ***ῡ*** **, cm^−^** ^**1**^
1	6a	330	370	1.205	3276
2	6c	332	365	2.30	2723
3	6d	333	365	2.85	2632
4	6f	332	388	2.30	4347
5	6h	328	363	3.70	2939
6	6j	328	364	2.53	3015
7	6k	328	368	1.90	3313
8	6m	327	364	2.30	3108
9	6n	331	364	2.68	2738
10	6o	326	365	2.35	3277
11	6p	330	397	1.75	5114

### 2.9 Catalyst Recyclability

**FIGURE 5:**
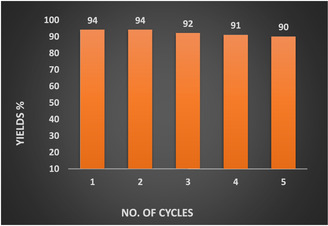
Recycling experiment of the ionic liquid **[VSBIM][Cl] (3b)**.

The recyclability of the Brønsted acidic ionic liquid [VSBIM][Cl] **3b** was performed in the model reaction under the optimized conditions. After completion of each cycle, the product precipitated in ethanol in the reaction vessel. The crude product was washed with ethanol and filtered to obtain the pure product. The ionic liquid was recovered from the filtrate, dried under reduced pressure, washed with ether, and reused directly in the subsequent run. After five consecutive cycles, a noticeable change in the yields of **6a** was not observed, as shown in Figure 5. The steady reduction in yield over consecutive runs may be due to negligible loss or probable leaching of the ionic liquid during the recovery process.

**FIGURE 6:**
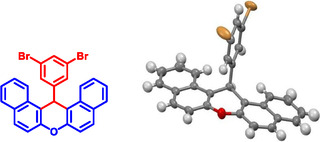
Single‐crystal X‐ray diffraction structure of compound **6r (CCDC No. 2422695) [** 64].

For better understanding, the expected 14‐(3,5‐dibromophenyl)‐14H‐dibenzo[a,j]xanthene **6r** structure was confirmed by single‐crystal X‐ray diffraction analysis (Figure 6). The ORTEP diagram clearly shows the dibromo phenyl substituent attached with the dibenzoxanthene core. Bond parameters fall within normal ranges, validating the molecular connectivity. The compound **6r** crystallized in the triclinic crystal system with space group $P\bar{1}$.

## 3 Conclusions

In conclusion, we have synthesized a task‐specific sulfonic acid functionalized ionic liquid 1‐vinyl‐3‐(4‐sulfobutyl)imidazolium chloride ([VSBIM][Cl]) **3b** and successfully employed its application as an efficient catalyst for the solvent‐free synthesis of 14‐(aryl)‐14H‐dibenzo[a,j]xanthene derivatives (**6a–6s**)**.** The present solvent‐free protocol offers several advantages, such as short reaction times, a transition metal‐free approach, low catalyst loading, simple work‐up, avoidance of column chromatographic purification, and the use of an inexpensive, non‐toxic, recyclable catalyst. A plausible reaction mechanism was proposed based on the experimental results. Moreover, the acidic ionic liquid catalyst was recycled and reused for five consecutive cycles without a significant loss of its catalytic activity. ADMET analysis provided preliminary information regarding the pharmacokinetic and drug‐likeness properties of the synthesized compounds. Molecular docking studies suggested possible interactions with the selected protein target. PASS and AVCpred analyses provided preliminary computational predictions of potential biological and antiviral activities. Overall, the combined experimental and computational studies provide preliminary insight into the potential applications of the synthesized dibenzoxanthene derivatives. In future, further experimental studies will be conducted to validate their biological and pharmacological properties.

## 4 Experimental Section

### 4.1 Material and Methods

2‐Naphthol, aromatic aldehydes, and 1‐vinyl imidazole were purchased from Sigma‐Aldrich; all organic solvents were acquired from commercial suppliers and used without any further purification. Analytical thin‐layer chromatography (TLC) was performed using 0.25‐mm silica gel‐coated Kieselgel 60 F^254^ plates. ^1^H NMR (400 MHz) and ^13^C NMR (100 MHz) spectra were recorded on a Bruker AVANCE III 400 MHz spectrometer. Chemical shifts and coupling constants are mentioned in parts per million (ppm) and Hertz (Hz), respectively, using tetra‐methyl silane (TMS) as an internal standard and the solvent resonance at (DMSO‐d6: ^1^H NMR 400 MHz and ^13^C NMR 100 MHz): δ 2.49 and 39.7 ppm. The peak multiplicities are represented as s (singlet), d (doublet), dd (doublet of doublet), m (multiplet), dt (doublet of triplet), and t (triplet). The FT‐IR spectra were obtained utilizing the Perkin‐Elmer RX‐I FTIR spectrometer in ATR mode. UV‐Vis absorption spectra were recorded on a JASCO V‐670 spectrophotometer, at room temperature in DMSO. Hitachi F‐7000 FL spectrophotometer was utilized to record emission fluorescence spectra by excitation at their respective absorption maxima.

### 4.2 General Procedure for the Synthesis of 1‐Vinyl‐3‐(4‐Sulfobutyl) Imidazolium Chloride [VSBIM][Cl^−^] Ionic Liquid 3b

A mixture of 1‐vinylimidazole (10 mmol) and 1,4‐butanesultone (10 mmol) was taken in a round‐bottom flask and heated under neat (solvent‐free) conditions at 80°C with continuous stirring for 1 h. During the reaction, the mixture gradually turned into a white solid. After completion of the reaction, the mixture was cooled to room temperature. The resulting crude product was washed with ethyl acetate (3 × 10 mL) to remove any unreacted starting materials. The residue was dried under vacuum to afford the zwitterionic compound **3a** as a white solid. The zwitterion‐ionic compound **3a** was directly treated with aqueous 1 M HCl at room temperature. The reaction mixture was stirred at room temperature for 6 h, during which the formation of a thick viscous liquid was observed. The obtained residue was successively washed with ethyl acetate and hexane to remove impurities and finally dried under high vacuum to afford the Brønsted acidic ionic liquid **3b** as a viscous liquid. The Brønsted acidic nature of [VSBIM][Cl] **3b** was evaluated using the Hammett acidity function (H_0_). The obtained H_0_ value confirmed the strong acidic character of the ionic liquid, which is responsible for its catalytic efficiency in the synthesis of dibenzoxanthene derivatives. The product was characterized using ^1^H NMR, ^13^C NMR, and FT‐IR spectroscopy.

### 4.3 Representative General Procedure for the Synthesis of 14‐Aryl‐14H‐Dibenzo[a,j]xanthene Derivatives **6a–6s**

To a dried 100‐mL round‐bottom flask, 2‐naphthol **5** (2 equiv, 2 mmol), benzaldehyde **4a** (1 equiv, 1 mmol), and **5 **mol% of **[VSBIM][Cl** ^−^ **]** ionic liquid **3b** was added and heated at 110°C under neat (solvent‐free condition) conditions for 40 min. The progress of the reaction was monitored by TLC. After completion of the reaction, ethanol (5 mL) was added to the mixture, and the solid precipitate was filtered and washed with ethanol. The final products were characterized by ^1^H NMR, ^13^C NMR, and FT‐IR spectra. The filtrate was dried under reduced pressure and high vacuum to recover the ionic liquid catalyst. The recovered ionic catalyst was used for subsequent run.

## Funding

This work was supported by the Science and Engineering Research Board (YSS/2015/000450); VIT University (SG20220031, SG20240023).

## Conflicts of Interest

The authors declare no conflicts of interest.

## Supporting information

Supplementary Material

## Data Availability

The data that support the findings of this study are available in the supplementary material of this article. Crystal structure analyses were deposited with the (CCDC No. 2422695).
