# Supplementary material for: Efficient One‐Pot Synthesis of Dibenzoxanthenes Using a Vinylimidazolium‐Based Brønsted Acidic Ionic Liquid [VSBIM][Cl]: In Silico Evaluation of Drug‐Likeness, ADMET Properties, PASS Analysis, Antiviral Activity Prediction, and Molecular Docking Studies
Source: ChemistryOpen. 2026 Aug 11;15(8):e70253. doi: 10.1002/open.70253 (PMC13458462; doi:10.1002/open.70253)
Supplement: Supplementary file 1 [file OPEN-15-e70253-s001.pdf]

Supporting Information

Efficient One-Pot Synthesis of Dibenzoxanthenes Using a Vinylimidazolium-Based Brønsted Acidic Ionic Liquid [VSBIM][Cl]: In Silico Evaluation of Drug-Likeness, ADMET Properties, PASS Analysis, Antiviral Activity Prediction, and Molecular Docking Studies

Talamarlla Deepthi, and Barnali Maiti*

*Department of Chemistry, School of Advanced Sciences, Vellore Institute of Technology, Vellore-632014, India.

E-mail: barnalimaiti.m@gmail.com

Table of Contents

S. No	Caption	Figure	Table	Page No.
1	Experimental Section	-	-	S3
2	Spectral characterization data for compound (6a-6s)	-	-	S4-S11
3	^{1}H NMR (400MHz), ^{13}C NMR (100MHz) in D_2O spectra for (compound 3b, Ionic liquid)	Fig. S1-S2	-	S12
4	DEPT-135 spectrum of 3b in D_2O and FT-IR spectra for (compound 3b, Ionic liquid)	Fig. S3-S4	-	S13
5	^{1}H NMR (400MHz), ^{13}C NMR (100MHz) in $CDCl_3$, FT-IR spectra for compound (6a-6s)	Fig. S5- S61	-	S14-S51
6	Calculating Drug-Likeness using swissADME data for compound (6a-6s)	-	Table-S1	S52
7	Assessment of (absorption, distribution, metabolism, excretion and toxicity (ADMET) properties data for compound (6a-6s)		Table-S2	S53-S54
8	PASS Prediction (Pa/Pi values for major biological activities) data for compound (6a-6s)	-	Table-S3	S55-S56
9	Predicted Antiviral Activity Scores data for compound (6a-6s)	-	Table-S4	S57

10	Molecular docking and 2D/3D interaction analyses with Human Cyclooxygenase-2 using Discovery Studio Visualizer for compound (6a-6s)	**Fig. S62- S175**	**-**	**S58-S118**
11	Binding affinity of Cyclooxygenase-2 and ligand calculated from Molecular docking studies (kcal mol-1) using PYRX data for compound (6a-6s)	**-**	**Table-S5**	**S119**
12	Single-Crystal X-ray Diffraction Data and Results for compound 6r	**-**	**Table-S6-S12**	**S120-S127**
13	Determination of Hammett Acidity (H_0) of the Synthesized Ionic Liquid Using UV–Vis Spectroscopy	**Fig. S176- S177**	**Table-S13**	**S128**
14	Comparison with Reported Catalysts	**-**	**Table-S14**	**S129**
15	Green chemistry metrics calculation	**-**	**Table-S15**	**S130-132**

Materials and Methods

2-naphthol, aromatic aldehydes, 1-vinyl imidazole, were purchased from Sigma-Aldrich, all organic solvents were acquired from commercial suppliers and used without any further purification. Analytical thin-layer chromatography (TLC) was performed using 0.25-mm silica gel-coated Kieselgel 60 F^{254} plates. ^{1}H NMR (400 MHz) and ^{13}C NMR (100 MHz) spectra were recorded on a Bruker AVANCE III 400 MHz spectrometer. Chemical shifts and coupling constants are mentioned in parts per million (ppm) and Hertz (Hz) respectively using tetra-methyl silane (TMS) as an internal standard and the solvent resonance at (DMSO-d6: ^{1}H NMR 400 MHz and ^{13}C NMR 100 MHz): δ 2.49 and 39.7 ppm; (CDCl3: ^{1}H NMR 400 MHz and ^{13}C NMR 100 MHz): δ 7.24and 77.0 ppm. The peak multiplicities are represented as s (singlet), d (doublet), dd (doublet of doublet), m (multiplet), dt (doublet of triplet), and t (triplet). The FT-IR spectra were obtained utilising the Perkin-Elmer RX-I FTIR spectrometer in ATR mode.

General procedure for the synthesis of 1-Vinyl-3-(4-sulfobutyl) imidazolium chloride [VSBIM][Cl^{-}] ionic liquid 3b.

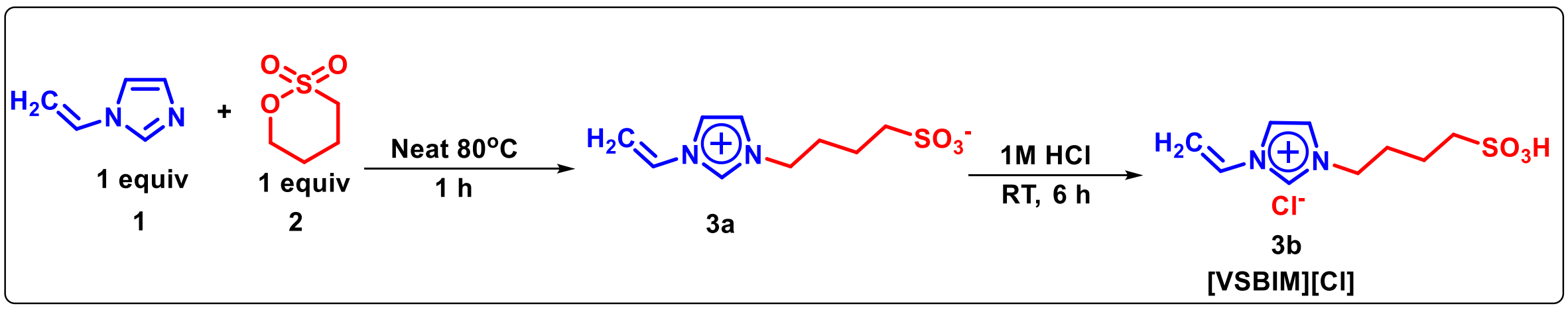

A mixture of 1-vinylimidazole (1.0 equiv) and 1,4-butanesultone (1.0 equiv) was taken in a round-bottom flask and heated under neat (solvent-free) conditions at 80 °C with continuous stirring for 1 h. During the reaction, the mixture gradually turned into a white solid. After completion of the reaction the reaction was cooled to room temperature. The resulting crude product was washed with ethyl acetate (3 × 10 mL) to remove any unreacted starting materials. The residue was dried under vacuum to afford the zwitterionic compound **3a** as a white solid. The zwitterionic compound **3a** was directly treated with aqueous 1 M HCl at room temperature. The reaction mixture was stirred at room temperature for 6 h, during which the formation of a thick viscous liquid was observed. The obtained residue was successively washed with ethyl acetate and hexane to remove impurities and finally dried under high vacuum to afford the Brønsted acidic ionic liquid **3b** as a viscous liquid, which partially solidified upon standing at room temperature The product was characterized using ^{1}H NMR, ^{13}C NMR, and FT-IR spectroscopy.

Spectral characterization data

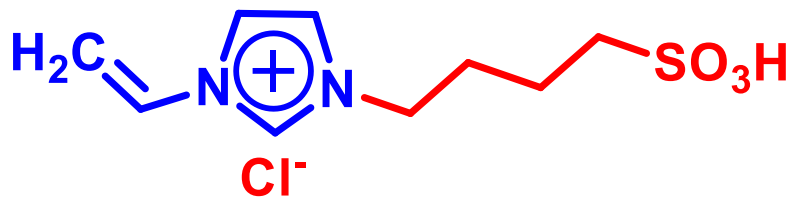

1-Vinyl-3-(4-sulfobutyl) imidazolium chloride [VSBIM][Cl$^-$] 3b.[1–4] Green viscous ionic liquid; Yield: 95**%; ^{1}H NMR (400 MHz, D2O, ppm)** 8.98 (s, 1H), 7.69 (s, 1H), 7.51 (s, 1H), 7.05 (dd, *J* = 15.6, 8.7 Hz, 1H), 5.71 (dd, *J* = 15.6, 2.8 Hz, 1H), 5.33 (dd, *J* = 8.7, 2.8 Hz, 1H), 4.21 (t, *J* = 7.1 Hz, 2H), 3.00 – 2.66 (m, 2H), 2.12 – 1.85 (m, 2H), 1.82 – 1.51 (m, 2H); **^{13}C NMR (100 MHz, D2O, ppm) δ**134.46, 128.23, 122.79, 119.58, 109.32, 50.01, 49.26, 27.93, 20.90. **DEPT-135 NMR (100 MHz, D_2O, ppm) δ** 109.31, 50.00, 49.28, 27.93, 20.89**; FT-IR (ATR-cm^{-1})**: 3348, 3086, 2920, 1616, 1462, 1169, 1026; **Mol. Formula**: $C_9H_{15}ClN_2O_3S^-$; **Mol. Wt:** 266.0497.

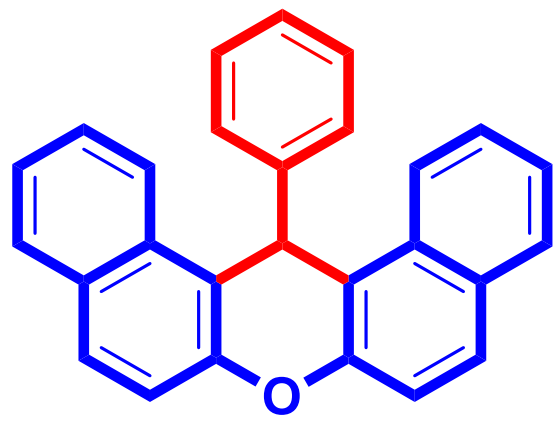

14-phenyl-14H-dibenzo[a,j]xanthene 6a. White solid; Yield: 95% ; **mp (°C):** 183-185; **^{1}H NMR (400 MHz, $CDCl_3$)** δ 8.31 (d, *J* = 8.5 Hz, 2H), 7.72 (dd, *J* = 14.3, 8.5 Hz, 4H), 7.45 (ddd, *J* = 24.0, 15.5, 8.0 Hz, 6H), 7.32 (t, *J* = 7.4 Hz, 2H), 7.06 (t, *J* = 7.7 Hz, 2H), 6.90 (t, *J* = 7.4 Hz, 1H), 6.40 (s, 1H); **^{13}C NMR (100 MHz, $CDCl_3$) δ** 148.74, 145.00, 143.37, 131.46, 131.06, 130.39, 130.33, 129.45, 129.02, 128.86, 128.80, 128.48, 128.26, 127.94, 126.80, 126.38, 125.49, 125.02, 124.25, 123.21, 122.91, 122.69, 118.02, 117.33, 112.00, 38.04**; FT-IR (ATR-cm^{-1})**: 3049, 1682, 1619, 1593, 1512, 1458, 1402, 1251, 1081, 1030, 962, 827, 802, 743, 701, 609, 510, 414.

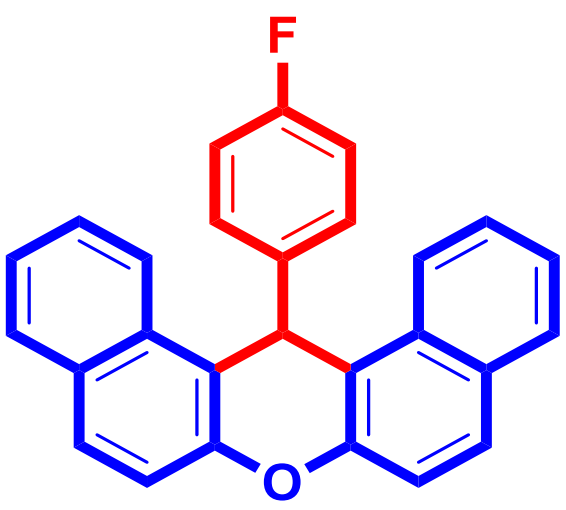

14-(4-fluorophenyl)-14H-dibenzo[a,j]xanthene 6b. white solid: Yield: 94% ; **mp (°C):** 240-250; **^{1}H NMR (400 MHz, $CDCl_3$)** δ 8.27 (d, *J* = 8.5 Hz, 2H), 7.75 (dd, *J* = 13.1, 8.5 Hz, 4H), 7.57 – 7.48 (m, 2H), 7.41 (d, *J* = 8.9 Hz, 2H), 7.35 (t, *J* = 7.5 Hz, 2H), 7.27 (d, *J* = 7.8 Hz, 1H),

7.11 – 6.96 (m, 2H), 6.61 (td, $J = 8.4, 1.9$ Hz, 1H), 6.43 (s, 1H); **^{13}C NMR (101 MHz, $CDCl_3$) δ** 148.72, 131.28, 131.06, 129.65, 129.57, 128.89, 126.87, 124.33, 122.46, 118.02, 117.09, 115.42, 115.21**; FT-IR (ATR-cm^{-1})**: 3082, 1594, 1503, 1493, 1431, 1401, 1245, 1161, 1097, 960, 815, 744, 660, 608, 523, 485.

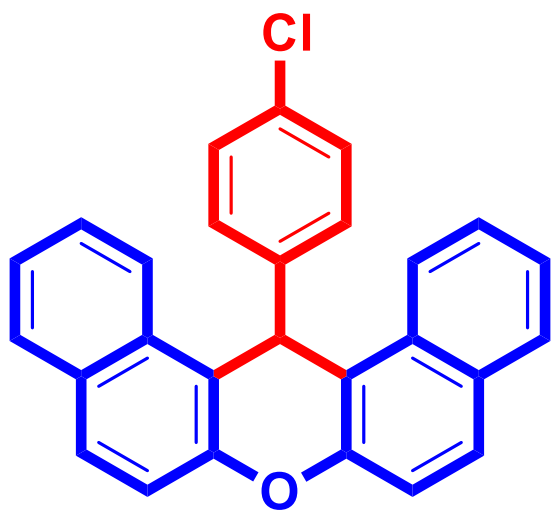

14-(4-chlorophenyl)-14H-dibenzo[a,j]xanthene 6c. white solid: Yield: 94%; **mp (ºC):** 296-298; **^{1}H NMR (400 MHz, $CDCl_3$)** δ 8.24 (d, $J = 8.5$ Hz, 2H), 7.74 (dd, $J = 14.1, 8.5$ Hz, 4H), 7.51 (dd, $J = 11.2, 4.1$ Hz, 2H), 7.43 – 7.30 (m, 6H), 7.02 (d, $J = 8.5$ Hz,2H), 6.39 (s, 1H); **^{13}C NMR (100 MHz, $CDCl_3$) δ** 148.69, 143.46, 132.08, 131.24, 131.05, 129.49, 129.09, 128.91, 128.64, 126.92, 124.37, 122.40, 120.21, 118.02, 116.75, 37.38; **FT-IR (ATR-cm^{-1})**: 3078, 1628, 1594, 1503, 1460, 1400, 1396, 1239, 1211, 1160, 1092, 1034, 960, 834, 814, 743, 699, 655, 607, 523, 485, 418.

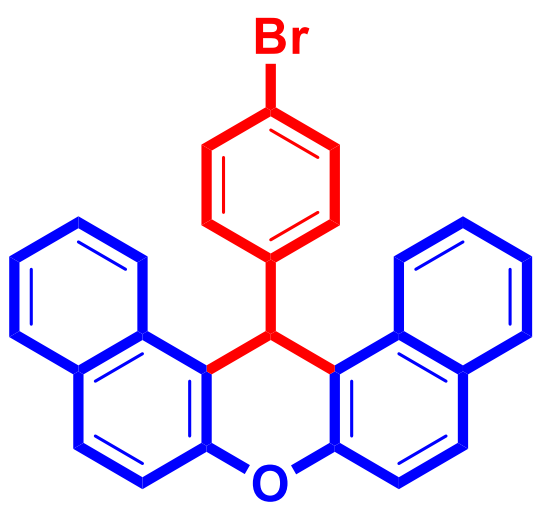

14-(4-bromophenyl)-14H-dibenzo[a,j]xanthene 6d. white solid: Yield: 93%; **mp (ºC):** 289-293; **^{1}H NMR (400 MHz, $CDCl_3$)** δ 8.24 (d, $J = 8.5$ Hz, 2H), 7.75 (dd, $J = 13.8, 8.5$ Hz, 4H), 7.54 – 7.48 (m, 2H), 7.43 – 7.28 (m, 6H), 7.19 – 7.15 (m, 2H), 6.39 (s, 1H); **^{13}C NMR (100 MHz, $CDCl_3$) δ** 148.70, 143.97, 131.58, 131.25, 131.06, 129.86, 129.10, 128.91, 126.92, 124.37, 122.38, 120.21, 118.01, 116.67; **FT-IR (ATR-cm^{-1})**: 3064, 1624, 1592, 1483, 1432, 1402, 1239, 1156, 1081, 1007, 963, 831, 807, 741, 707, 696, 608, 514, 429.

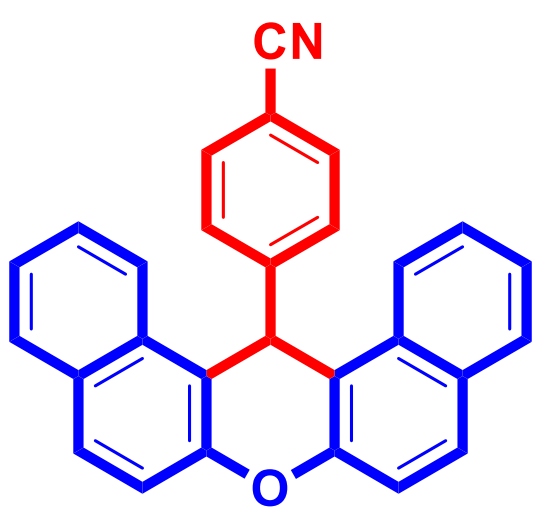

4-(14H-dibenzo[a,j]xanthen-14-yl)benzonitrile 6e. White solid: Yield: 93%; **mp (ºC):** 305-310; **^{1}H NMR (400 MHz, $CDCl_3$)** δ 8.20 (d, *J* = 8.5 Hz, 2H), 7.77 (t, *J* = 8.8 Hz, 4H), 7.58 – 7.49 (m, 4H), 7.44 – 7.34 (m, 6H), 6.48 (s, 1H); **^{13}C NMR (100 MHz, $CDCl_3$) δ** 150.03, 148.82, 132.41, 131.13, 131.06, 129.50, 129.04, 128.88, 127.14, 124.55, 122.08, 118.04, 115.94, 110.32, 38.03; **FT-IR (ATR-cm^{-1})**: 2226, 1591, 1512, 1457, 1401, 1239, 1142, 1063, 959, 840, 810, 738, 696, 660, 611, 563, 547;

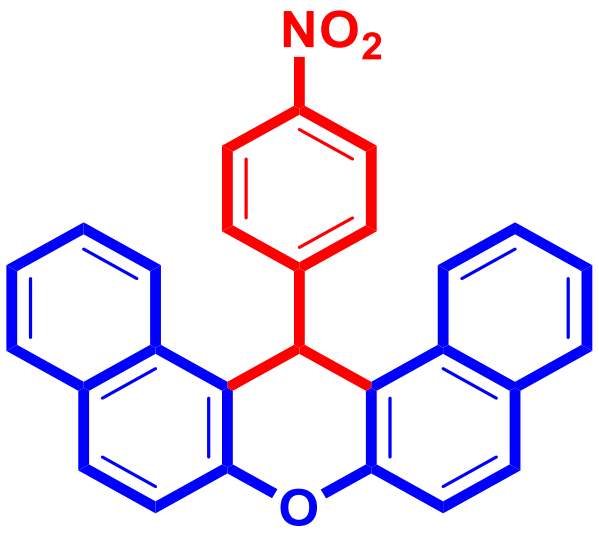

14-(4-nitrophenyl)-14H-dibenzo[a,j]xanthene 6f. White solid: Yield: 94%; **mp (ºC):** 305-309; **^{1}H NMR (400 MHz, $CDCl_3$)** δ 8.21 (d, *J* = 8.5 Hz, 2H), 7.93 (d, *J* = 8.8 Hz, 2H), 7.81 – 7.74 (m, 4H), 7.60 (d, *J* = 8.8 Hz, 2H), 7.53 (dd, *J* = 11.3, 4.1 Hz, 2H), 7.43 (d, *J* = 8.9 Hz, 2H), 7.37 (t, *J* = 7.2 Hz, 2H), 6.54 (s, 1H); **^{13}C NMR (100 MHz, $CDCl_3$) δ** 152.00, 148.60, 146.32, 131.10, 131.08, 120.00, 120.07, 128.96, 127.10, 124.56, 123.66, 122.04, 116.06, 116.76, 37.67; **FT-IR (ATR-cm^{-1})**: 3073, 1589, 1514, 1458, 1429, 1401, 1339, 1234, 1106, 963, 851, 826, 808, 741, 695, 607, 512, 441.

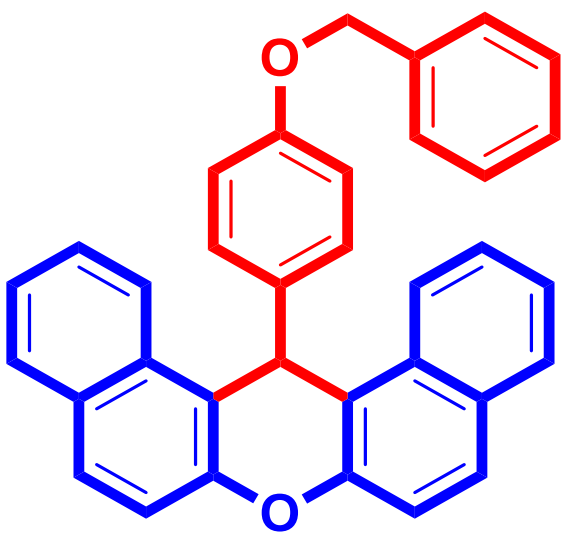

14-(4-(benzyloxy)phenyl)-14H-dibenzo[a,j]xanthene 6g. White solid: Yield: 95%; **mp (ºC):** 298-300 ; **^{1}H NMR (400 MHz, $CDCl_3$)** δ 8.31 (s, 1H), 8.29 (s, 1H), 7.76 (s, 1H), 7.74 (s, 1H), 7.72 (s, 1H), 7.69 (s, 1H), 7.50 (t, *J* = 7.3 Hz, 2H), 7.39 (d, *J* = 8.9 Hz, 2H), 7.32 (d, *J* = 6.8 Hz, 4H), 7.18 (dd, *J* = 8.5, 5.5 Hz, 5H), 6.66 (d, *J* = 8.7 Hz, 2H), 6.38 (s, 1H), 4.77 (s, 2H); **^{13}C NMR (100 MHz, $CDCl_3$) δ** 148.68, 137.37, 131.40, 131.07, 129.14, 128.79, 128.71, 128.75, 126.20, 122.67, 117.99, 117.52, 113.84, 55.06, 37.08**; FT-IR (ATR-cm^{-1})**: 3034, 1591, 1507, 1454, 1399, 1240, 1178, 1028, 964, 822, 800, 740, 697, 607, 502.

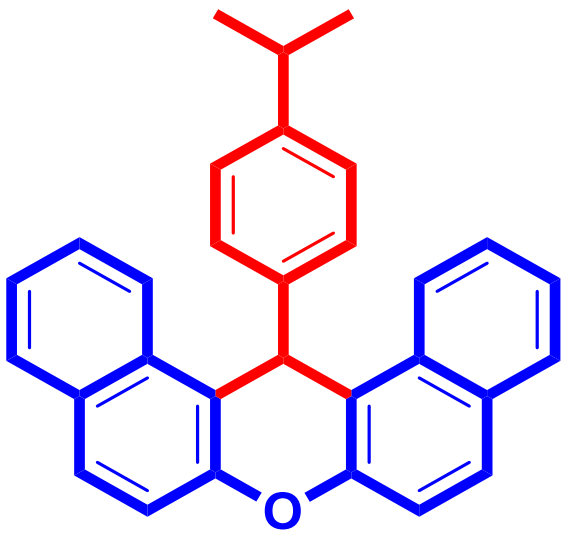

14-(4-isopropylphenyl)-14H-dibenzo[a,j]xanthene 6h. White solid: Yield: 95%; **mp (ºC):** 295-298; **^{1}H NMR (400 MHz, $CDCl_3$) δ** 8.33 (d, J = 8.5 Hz, 2H), 7.78–7.70 (m, 4H), 7.56–7.48 (m, 2H), 7.43–7.34 (m, 4H), 6.90 (d, J = 8.2 Hz, 2H), 6.39 (s, 1H), 2.62 (sept, J = 6.9 Hz, 1H), 0.99 (d, J = 6.9 Hz, 6H).**^{13}C NMR (100 MHz, $CDCl_3$) δ** 148.81, 146.62, 142.24, 131.50, 131.04, 128.74, 128.68, 128.00, 126.70, 126.52, 124.17, 122.79, 118.02, 117.64, 37.53, 33.46, 30.94, 23.75; **FT-IR (ATR-cm^{-1})**: 3216, 2944, 1617, 1589, 1517, 1452, 1429, 1394, 1242, 1137, 1015, 963, 832, 798, 738, 693, 543, 515, 455.

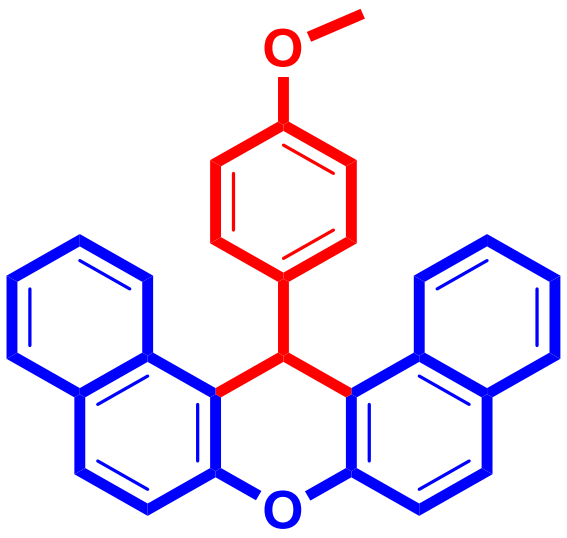

14-(4-methoxyphenyl)-14H-dibenzo[a,j]xanthene 6i. White solid: Yield: 94%; **mp (ºC):** 220-225; **^{1}H NMR (400 MHz, $CDCl_3$)** 8.30 (d, *J* = 8.5 Hz, 2H), 7.81 – 7.64 (m, 4H), 7.49 (t, *J* = 7.2 Hz, 2H), 7.41 – 7.29 (m, 6H), 6.59 (d, *J* = 8.7 Hz, 1H), 6.37 (s, 1H), 3.53 (s, 3H); **^{13}C NMR (100 MHz, $CDCl_3$) δ** 148.68, 137.37, 131.40, 131.07, 129.14, 128.79, 128.71, 126.75, 124.20, 122.67, 117.99, 117.52, 113.84, 55.06, 37.08; **FT-IR (ATR-cm^{-1})**: 3069, 1594, 1508, 1462, 1429, 1397, 1241, 1175, 1106, 1032, 959, 831, 807, 742, 655, 611, 518.

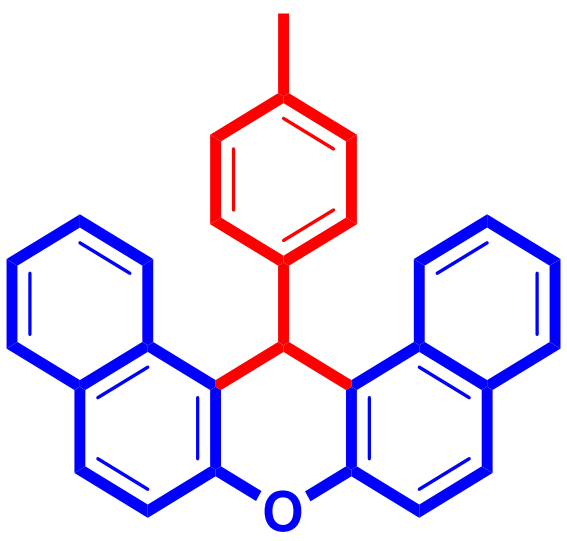

14-(p-tolyl)-14H-dibenzo[a,j]xanthene 6j. White solid: Yield: 94%; **mp (ºC):** 215-220; **^{1}H NMR (400 MHz, $CDCl_3$)** δ 8.30 (d, *J* = 8.5 Hz, 2H), 7.71 (dd, *J* = 15.8, 8.5 Hz, 4H), 7.52 – 7.44 (m, 2H), 7.39 (d, *J* = 8.9 Hz, 2H), 7.31 (dd, *J* = 11.9, 4.2 Hz, 4H), 6.86 (d, *J* = 7.9 Hz, 2H), 6.37 (s, 1H), 2.04 (s, 3H); **^{13}C NMR (100 MHz, $CDCl_3$) δ** 148.67, 142.11, 135.89, 131.44, 131.07, 129.17, 128.77, 128.74, 128.09, 126.75, 124.20, 122.69, 117.99, 117.44, 37.61,

20.89; **FT-IR (ATR-cm^{-1})**: 3069, 1591, 1509, 1454, 1431, 1397, 1237, 1080, 958, 807, 737, 655, 607, 515, 486.

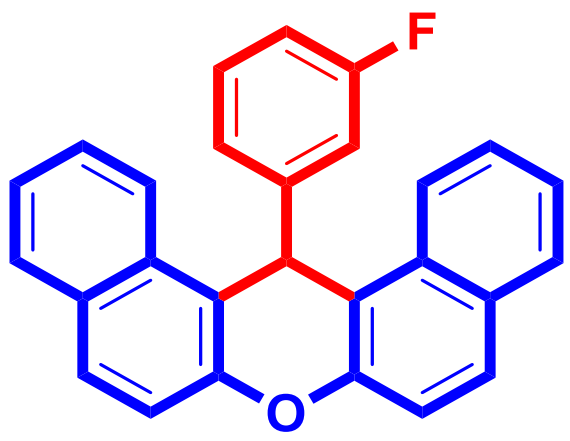

<u>**14-(3-fluorophenyl)-14H-dibenzo[a,j]xanthene**</u> **6k.** White solid: Yield: 91%; **mp (ºC):** 240-249; **^{1}H NMR (400 MHz, CDCl$_3$)** δ 8.26 (d, *J* = 8.5 Hz, 2H), 7.75 (dd, *J* = 13.2, 8.5 Hz, 4H), 7.51 (t, *J* = 7.3 Hz, 2H), 7.41 (d, *J* = 8.9 Hz, 2H), 7.35 (t, *J* = 7.3 Hz, 2H), 7.26 (d, *J* = 7.8 Hz, 1H), 7.04 (dt, *J* = 14.0, 8.8 Hz, 2H), 6.61 (td, *J* = 8.3, 2.0 Hz, 1H), 6.42 (s, 1H); **^{13}C NMR (100 MHz, CDCl$_3$) δ** 148.71, 140.79, 131.27, 131.06, 129.65, 129.57, 128.99, 128.89, 126.88, 124.33, 122.46, 118.02, 117.08, 115.43, 115.21, 37.18**; FT-IR (ATR-cm^{-1})**: 3052, 1589, 1517, 1482, 1431, 1402, 1241, 1159, 1078, 964, 806, 777, 741, 695, 663, 623.

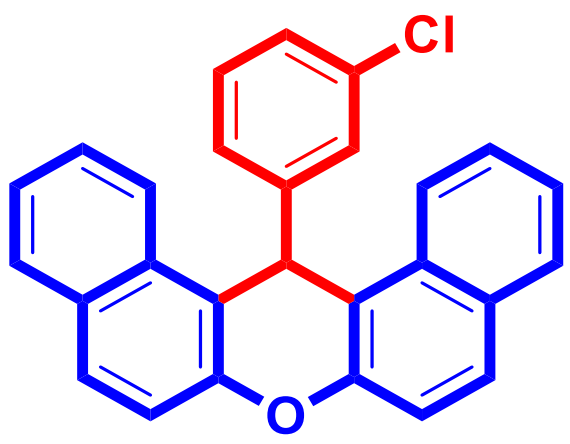

<u>**14-(3-chlorophenyl)-14H-dibenzo[a,j]xanthene**</u> **6l.** White solid: Yield: 92%; **mp (ºC):** 189-191; **^{1}H NMR (400 MHz, CDCl$_3$)** δ 8.24 (d, *J* = 8.5 Hz, 2H), 7.74 (dd, *J* = 13.1, 8.5 Hz, 4H), 7.55 – 7.49 (m, 2H), 7.41 (d, *J* = 8.9 Hz, 2H), 7.35 (ddd, *J* = 8.9, 7.7, 3.4 Hz, 4H), 7.00 (t, *J* = 7.9 Hz, 1H), 6.91 – 6.86 (m, 1H), 6.39 (s, 1H); **^{13}C NMR (100 MHz, CDCl$_3$) δ** 148.77, 146.87, 134.41, 131.41, 131.25, 131.05, 129.60, 129.17, 128.90, 128.33, 126.97, 126.75, 126.40, 124.39, 122.39, 118.08, 116.56; **FT-IR (ATR-cm^{-1})**: 3060, 1625, 1590, 1511, 1564, 1427, 1405, 1248, 1077, 962, 806, 776, 742, 697, 617, 507.

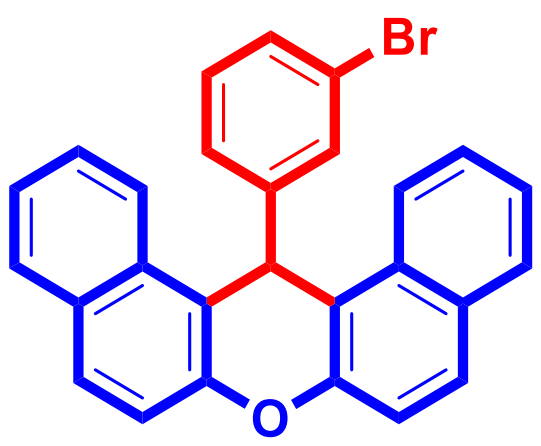

<u>**14-(3-bromophenyl)-14H-dibenzo[a,j]xanthene**</u> **6m**. White solid: Yield: 90%; **mp (ºC):** 185-190; **^{1}H NMR (400 MHz, CDCl$_3$)** 8.24 (d, *J* = 8.5 Hz, 2H), 7.75 (dd, *J* = 13.4, 8.5 Hz,

4H), 7.52 (t, J = 7.6 Hz, 3H), 7.41 (d, J = 8.7 Hz, 3H), 7.35 (t, J = 7.5 Hz, 2H), 7.04 (d, J = 7.7 Hz, 1H), 6.94 (t, J = 7.8 Hz, 1H), 6.38 (s, 1H); **^{13}C NMR (100 MHz, $CDCl_3$) δ** 148.77, 147.14, 131.24, 131.16, 131.05, 129.92, 129.66, 129.18, 128.90, 126.97, 126.87, 124.39, 122.74, 122.37, 118.08, 116.54, 37.74; **FT-IR (ATR-cm^{-1})**: 3065, 1592, 1511, 1429, 1398, 1240, 1143, 1061, 960, 832, 808, 774, 745, 691, 612, 511.

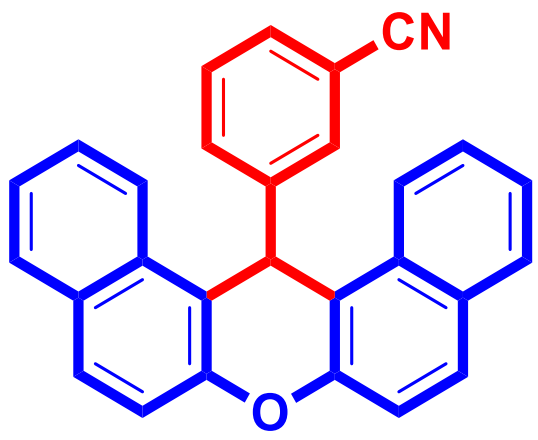

3-(14H-dibenzo[a,j]xanthen-14-yl)benzonitrile 6n. White solid: Yield: 90%; **mp (°C):**189-195; **^{1}H NMR (400 MHz, $CDCl_3$)** δ 8.20 (d, J = 8.5 Hz, 2H), 7.78 (t, J = 9.0 Hz, 4H), 7.72 – 7.66 (m, 2H), 7.54 (dd, J = 11.3, 4.0 Hz, 2H), 7.43 (d, J = 8.9 Hz, 2H), 7.38 (t, J = 7.2 Hz, 2H), 7.25 – 7.15 (m, 1H), 6.46 (s, 1H); **^{13}C NMR (100 MHz, $CDCl_3$) δ** 148.83, 146.35, 132.52, 131.61, 131.06, 130.22, 129.32, 129.05, 127.19, 124.55, 122.03, 118.84, 118.10, 116.00, 112.53, 37.55; **FT-IR (ATR-cm^{-1})**: 3061, 2228, 1619, 1589, 1513, 1483, 1455, 1433, 1398, 1236, 1141, 1070, 957, 801, 739, 691, 580, 508, 418.

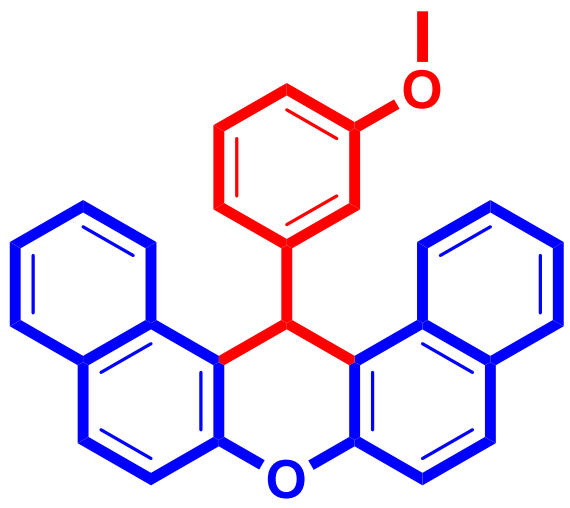

14-(3-methoxyphenyl)-14H-dibenzo[a,j]xanthene 6o. White solid: Yield: 95%; **mp (°C):** 187-190; **^{1}H NMR (400 MHz, $CDCl_3$)** δ 8.32 (d, J = 8.5 Hz, 2H), 7.73 (dd, J = 15.2, 8.5 Hz, 4H), 7.54 – 7.46 (m, 2H), 7.40 (d, J = 8.9 Hz, 2H), 7.33 (t, J = 7.5 Hz, 2H), 7.08 (d, J = 7.8 Hz, 1H), 7.02 – 6.94 (m, 2H), 6.45 (dd, J = 7.9, 2.2 Hz, 1H), 6.39 (s, 1H), 3.55 (s, 3H); **^{13}C NMR (100 MHz, $CDCl_3$) δ** 159.60, 148.76, 146.49, 131.46, 131.05, 129.27, 128.85, 128.77, 126.78, 124.23, 122.71, 120.78, 118.02, 117.18, 114.92, 110.98, 55.02, 37.96; **FT-IR (ATR-cm^{-1})**: 3016, 1580, 1512, 1486, 1452, 1431, 1399, 1237, 1138, 1049, 959, 866, 808, 773, 741, 699, 507.

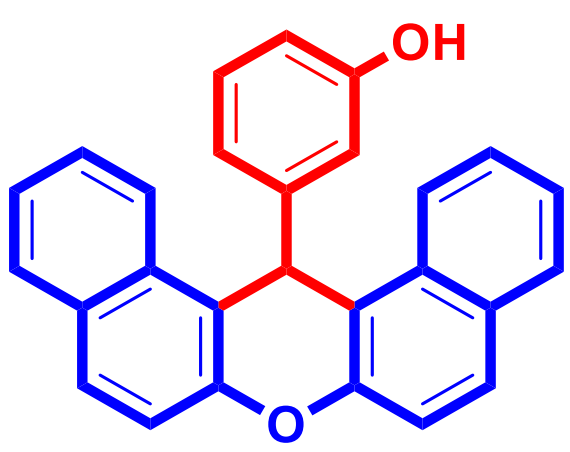

3-(14H-dibenzo[a,j]xanthen-14-yl)phenol 6p. White solid: Yield: 94%; **mp (°C):** 198-205 ; **^{1}H NMR (400 MHz, CDCl$_3$)** δ 8.30 (d, *J* = 8.5 Hz, 2H), 7.73 (dd, *J* = 15.5, 8.5 Hz, 4H), 7.50 (t, *J* = 7.6 Hz, 2H), 7.43 – 7.30 (m, 2H), 7.10 (d, *J* = 7.7 Hz, 1H), 6.94 (t, *J* = 7.9 Hz, 1H), 6.80 (s, 1H), 6.37 (s, 1H), 4.58 (s, 1H); **^{13}C NMR (100 MHz, CDCl$_3$) δ** 155.56, 148.75, 131.44, 131.05, 129.42, 128.89, 128.78, 126.81, 124.27, 122.69, 120.82, 118.00, 117.13, 115.37, 113.58, 37.79; **FT-IR (ATR-cm^{-1})**: 3457, 1588, 1520, 1452, 1428, 1233, 1178, 1139, 1081, 960, 857, 813, 744, 697, 578, 511.

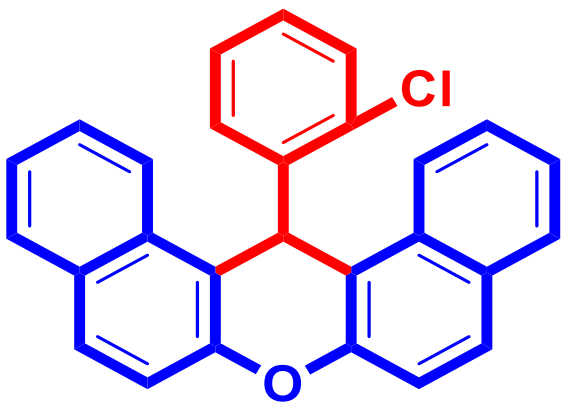

14-(2-chlorophenyl)-14H-dibenzo[a,j]xanthene 6q. White solid: Yield: 92%; **mp (°C):** 175-180; **^{1}H NMR (400 MHz, CDCl$_3$)** δ 8.67 (d, J = 1.6 Hz, 1H), 8.65 (d, J = 1.6 Hz, 1H), 7.78–7.75 (m, 2H), 7.73–7.69 (m, 2H), 7.58–7.51 (m, 3H), 7.49–7.39 (m, 4H), 7.35–7.28 (m, 3H), 7.24–7.16 (m, 2H), 6.88–6.73 (m, 3H). **^{13}C NMR (100 MHz, CDCl$_3$) δ** 148.98, 143.57, 131.80, 131.76, 130.90, 130.14, 129.61, 129.07, 128.64, 127.94, 127.87, 126.92, 124.43, 123.45, 118.11, 118.03, 34.64**; FT-IR (ATR-cm^{-1})**: 3056, 1592, 1510, 1460, 1402, 1253, 1065, 1034, 963, 826, 809, 750, 713, 689, 607, 520, 489, 424.

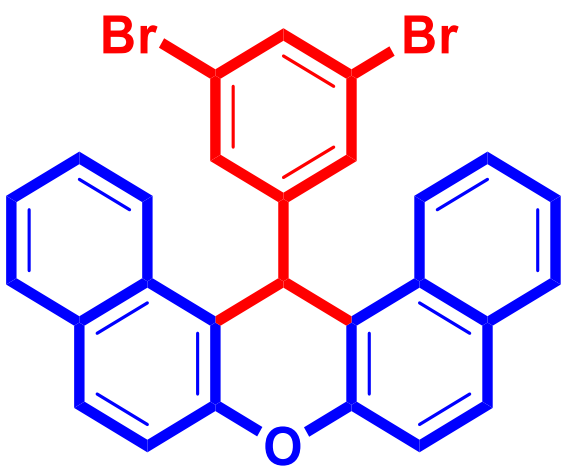

14-(3,5-dibromophenyl)-14H-dibenzo[a,j]xanthene 6r. White solid: Yield: 90%; **mp (°C):** 300-305; **^{1}H NMR (400 MHz, CDCl$_3$)** δ 8.32 (d, *J* = 8.5 Hz, 2H), 7.78 (dd, *J* = 18.4, 8.5 Hz, 4H), 7.56 (t, *J* = 7.2 Hz, 2H), 7.42 – 7.34 (m, 4H), 6.86 (d, *J* = 5.1 Hz, 1H), 6.76 (s, 1H), 6.73 (d, *J* = 3.5 Hz, 1H), 6.62 (dd, *J* = 5.0, 3.6 Hz, 1H); **^{13}C NMR (100 MHz, CDCl$_3$) δ** 148.80, 148.62, 132.34, 131.07, 129.94, 129.50, 129.03, 127.18, 124.55, 123.09, 122.11, 118.14, 115.84, 114.33, 37.54; **FT-IR (ATR-cm^{-1})**: 3067, 1593, 1550, 1516, 1398, 1246, 1078, 961, 851, 805, 739, 675, 613, 507.

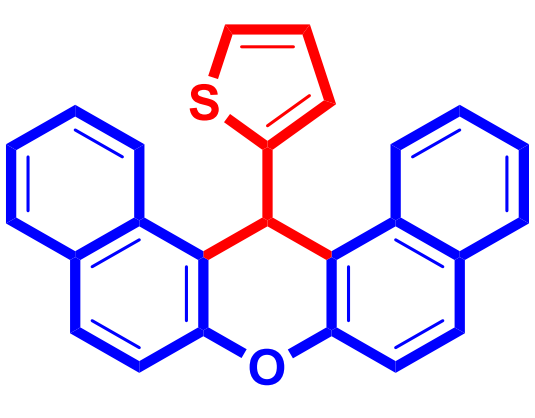

<u>14-(thiophen-2-yl)-14H-dibenzo[a,j]xanthene</u> 6s. White solid: Yield: 90%; **mp (ºC):** 232-235; **^{1}H NMR (400 MHz, $CDCl_3$)** δ 8.33 (d, *J* = 8.5 Hz, 2H), 7.83 – 7.71 (m, 4H), 7.56 (t, *J* = 7.2 Hz, 2H), 7.43 – 7.35 (m, 4H), 6.89 – 6.83 (m, 1H), 6.77 (s, 1H), 6.73 (d, *J* = 3.4 Hz, 1H), 6.62 (dd, *J* = 5.0, 3.6 Hz, 1H); **^{13}C NMR (100 MHz, $CDCl_3$) δ** 184.81, 148.47, 138.48, 136.61, 131.29, 131.10, 129.73, 129.46, 126.06, 127.48, 126.61, 125.47, 125.21, 125.11, 123.91, 118.09, 117.32, 56.50, 19.03**; FT-IR (ATR-cm^{-1})**: 3053, 1649, 1623, 1592, 1509, 1435, 1232, 1108, 1016, 811, 742, 663, 507.

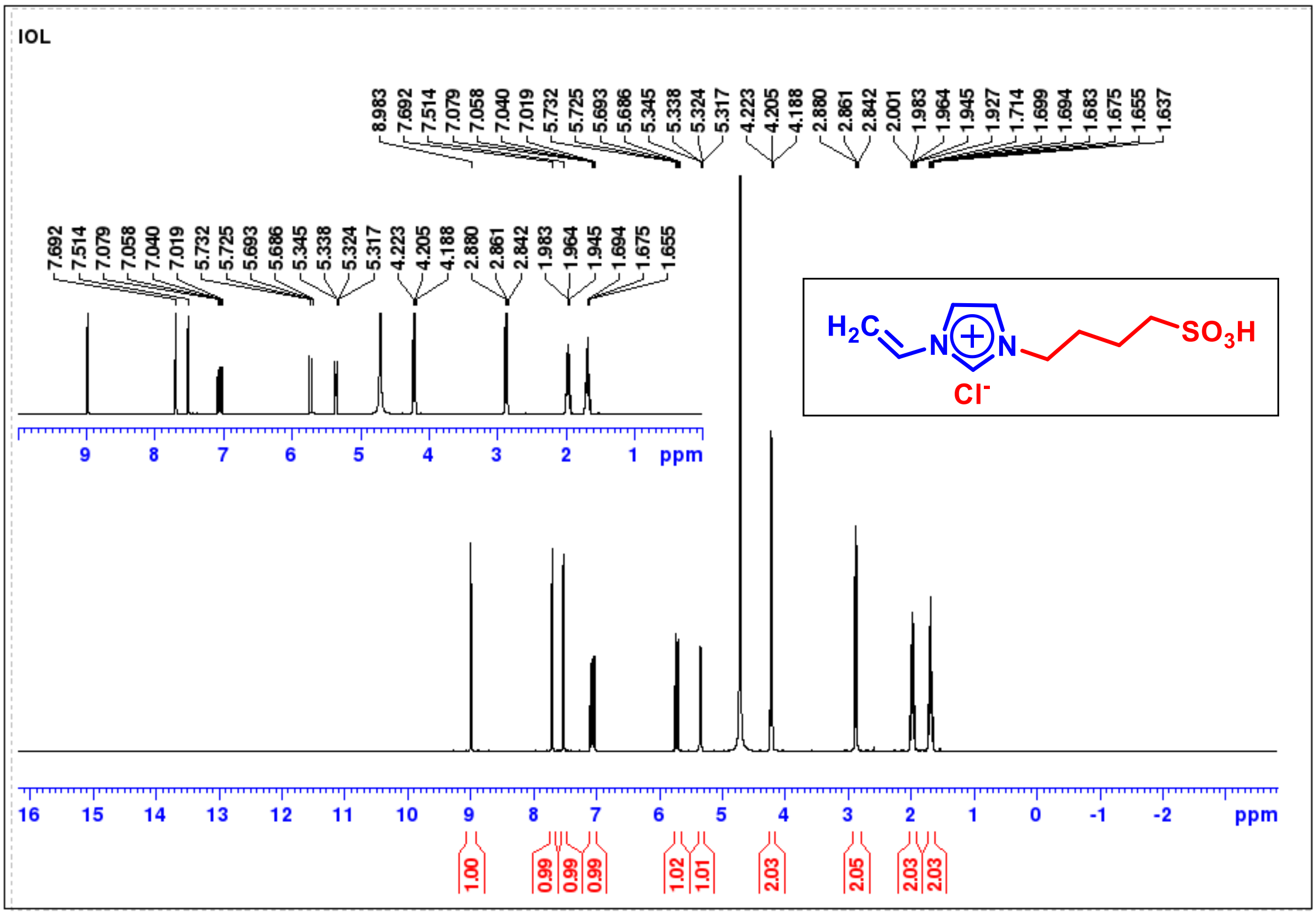

Fig. S1. ^{1}H NMR spectrum (400 MHz) of 3b

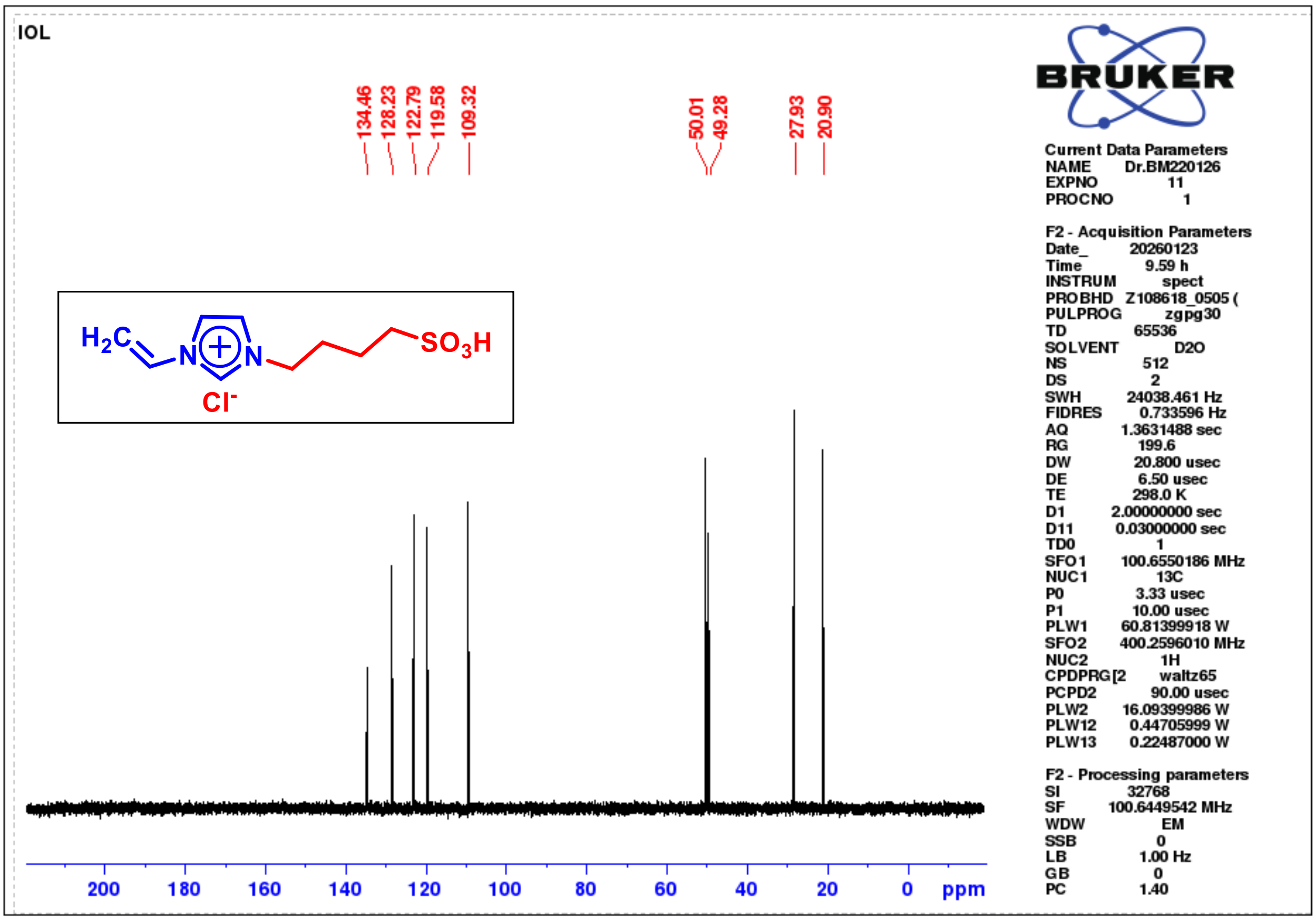

Fig. S2. ^{13}C NMR spectrum (100 MHz) of 3b in D2O.

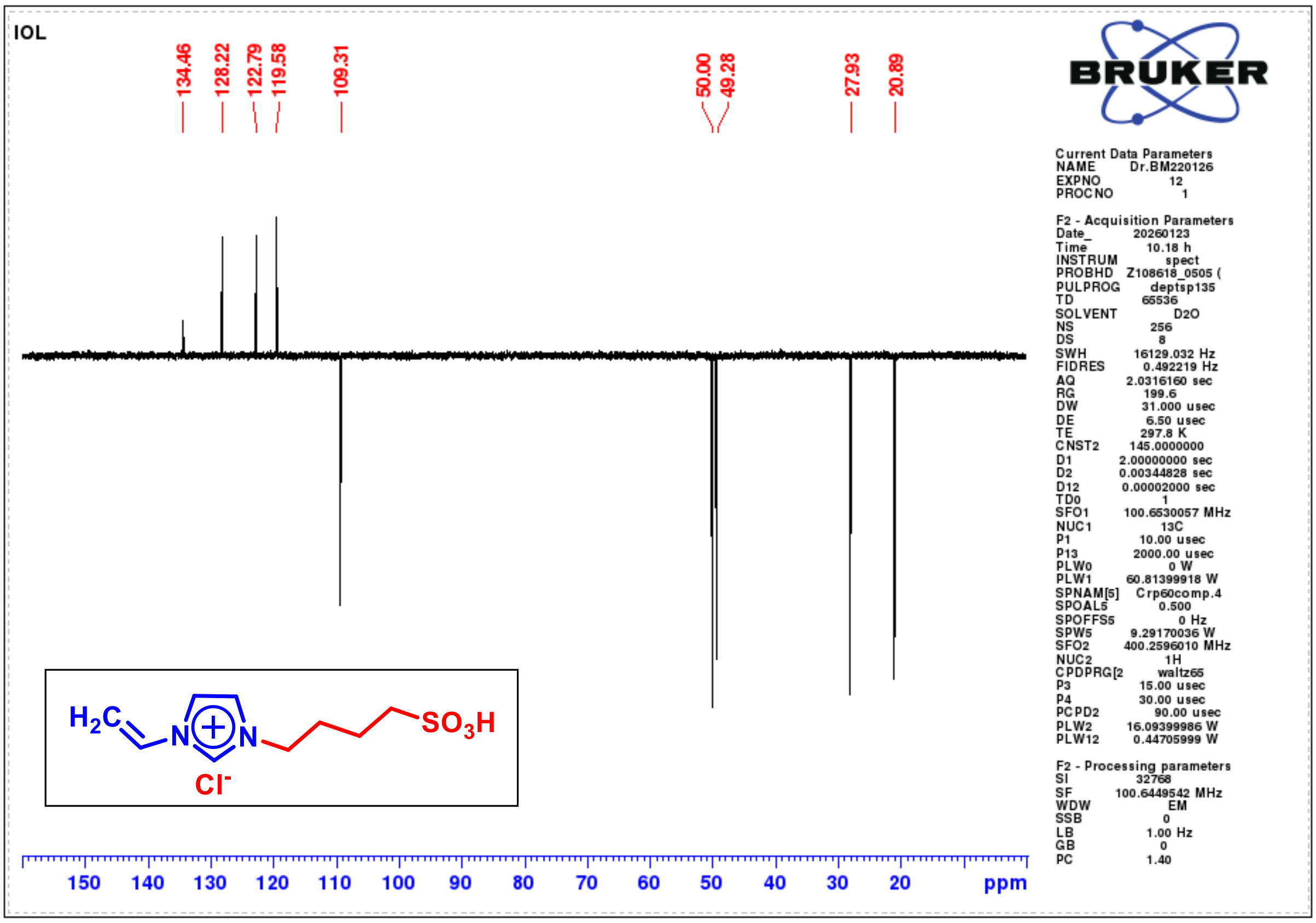

Fig. S3. DEPT-135 spectrum of (100 MHz) 3b in D2O

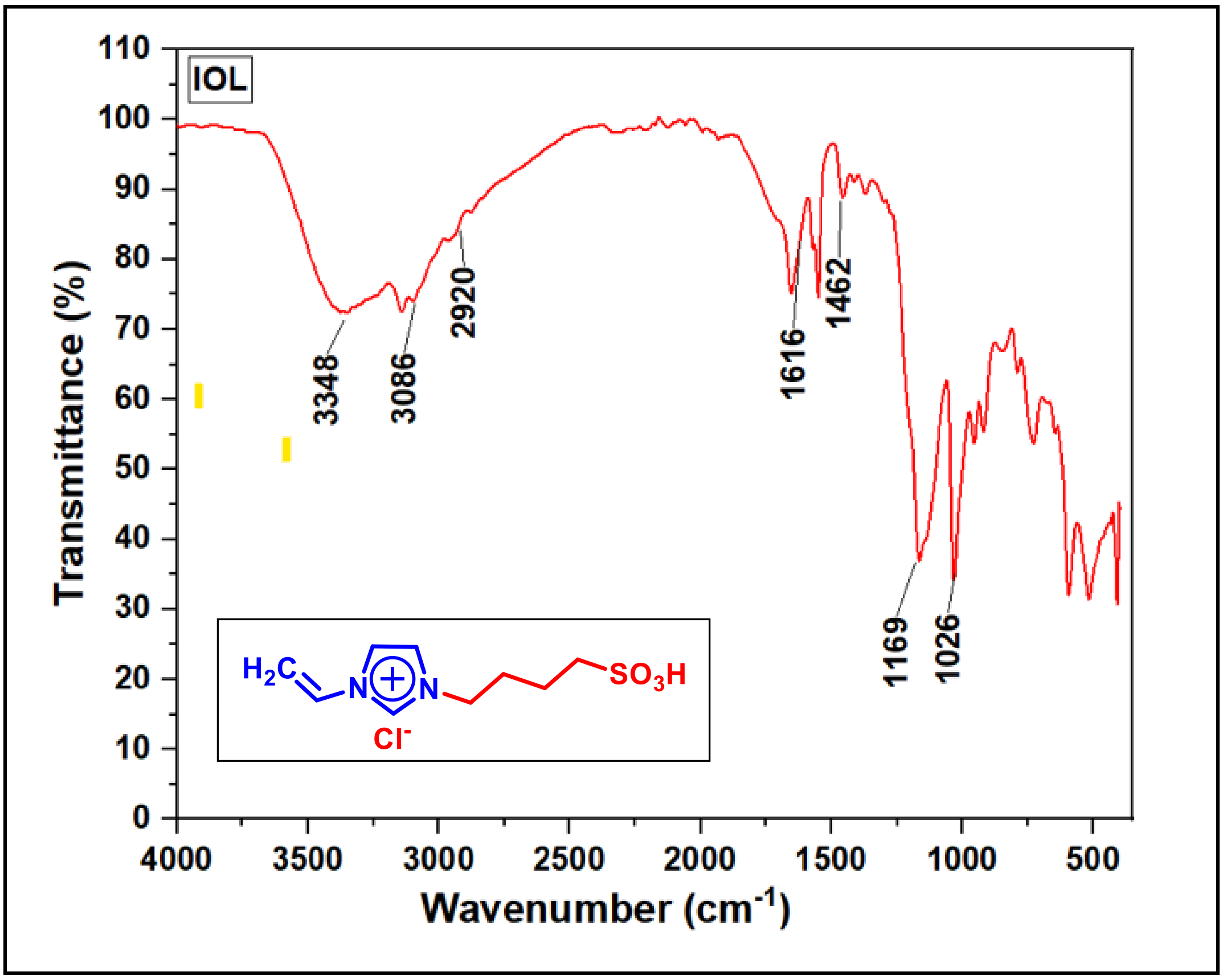

Fig. S4. FT-IR spectrum of compound 3b

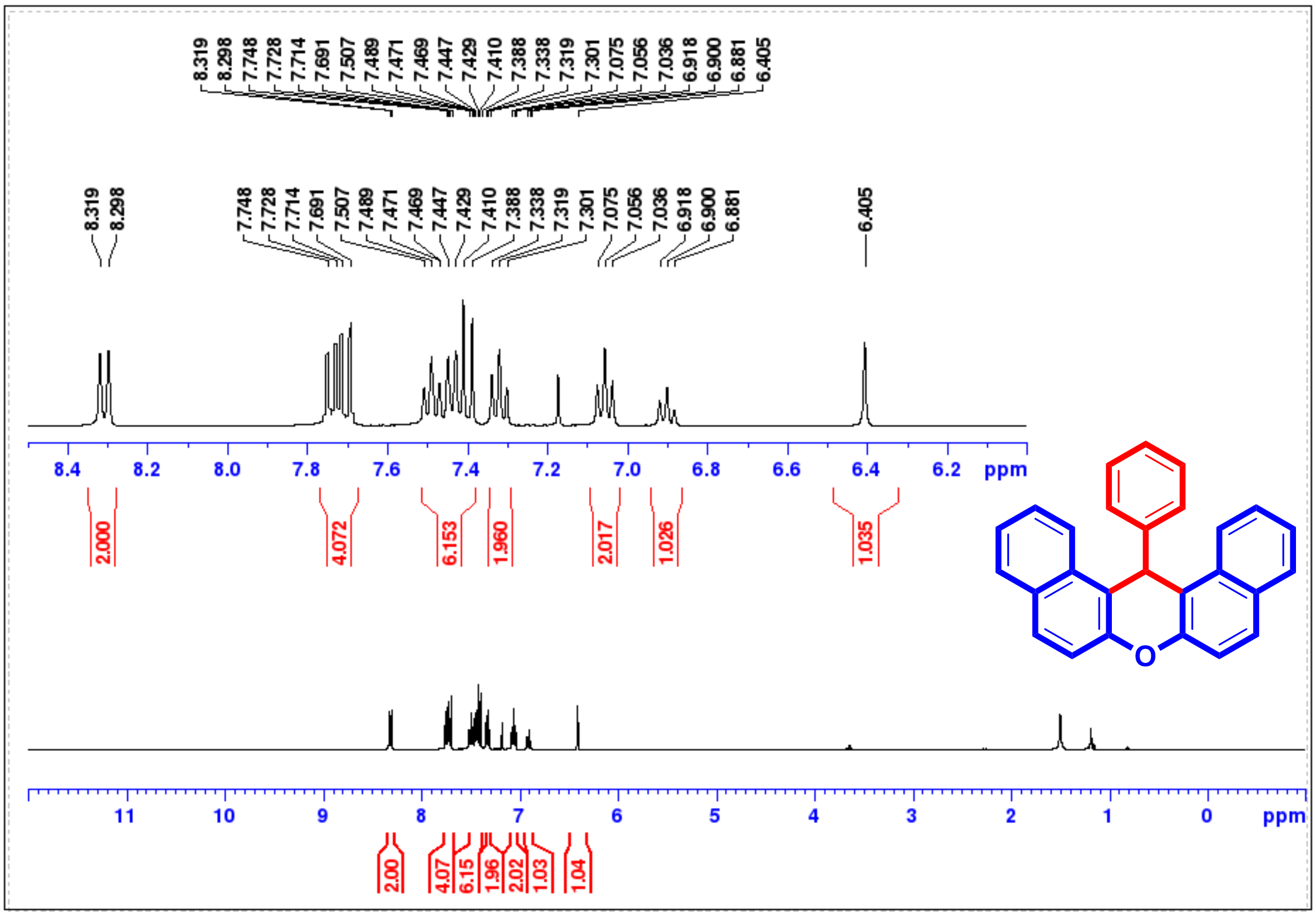

Fig. S5. ^{1}H NMR spectrum (400 MHz) of compound 6a in $CDCl_3$

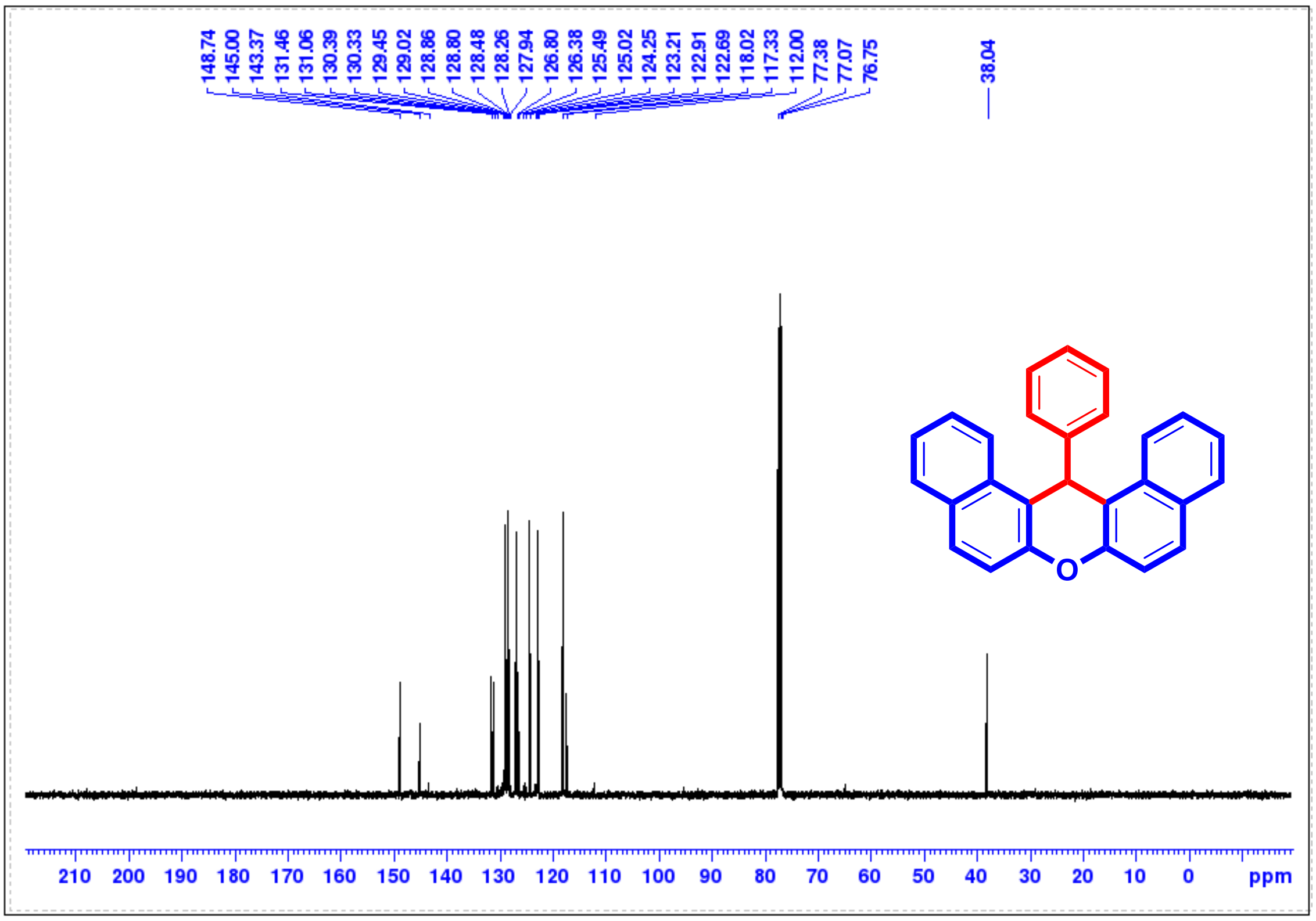

Fig. S6. ^{13}C NMR spectrum (100 MHz) of compound 6a in $CDCl_3$

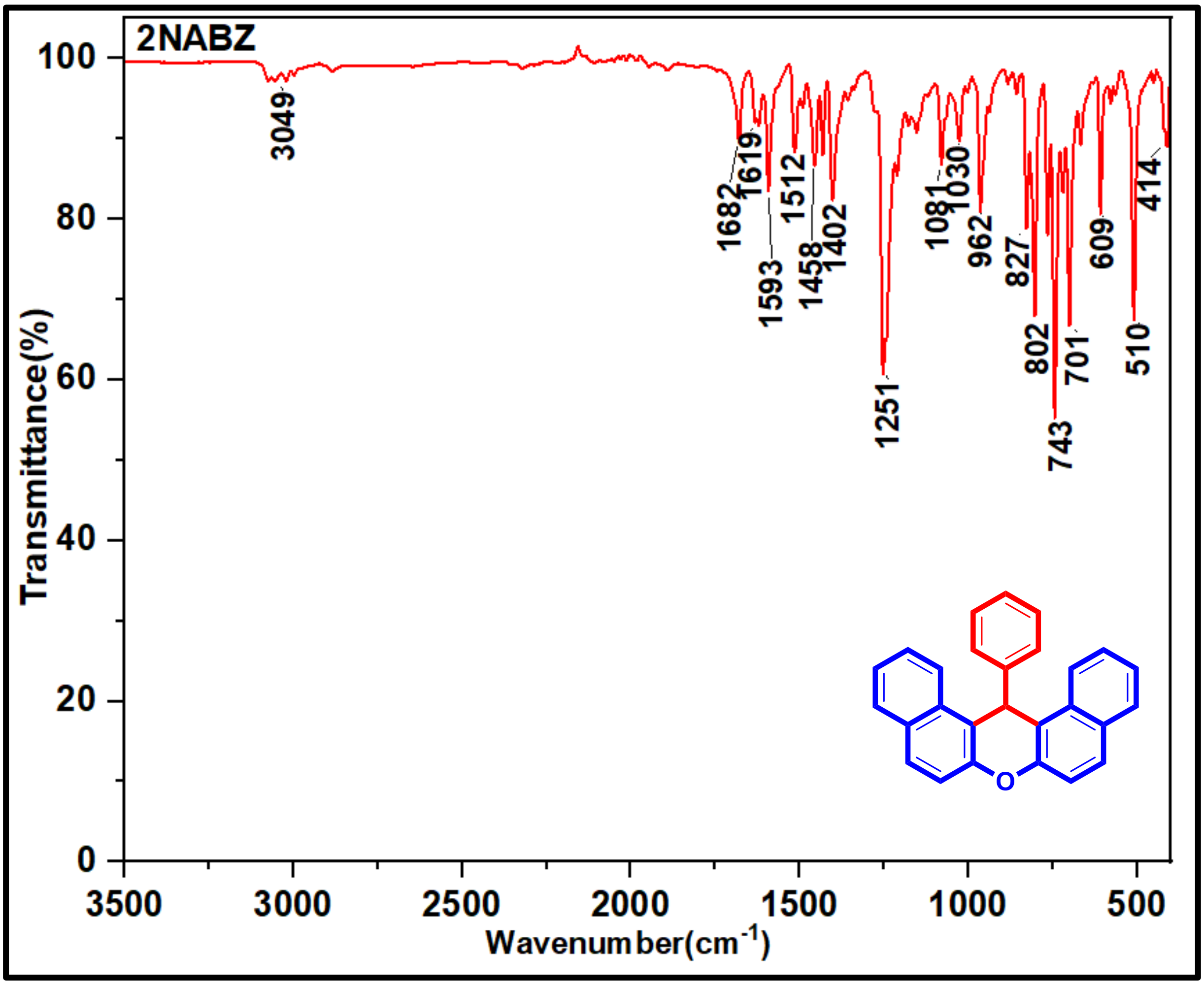

Fig. S7. FT-IR spectrum of compound 6a

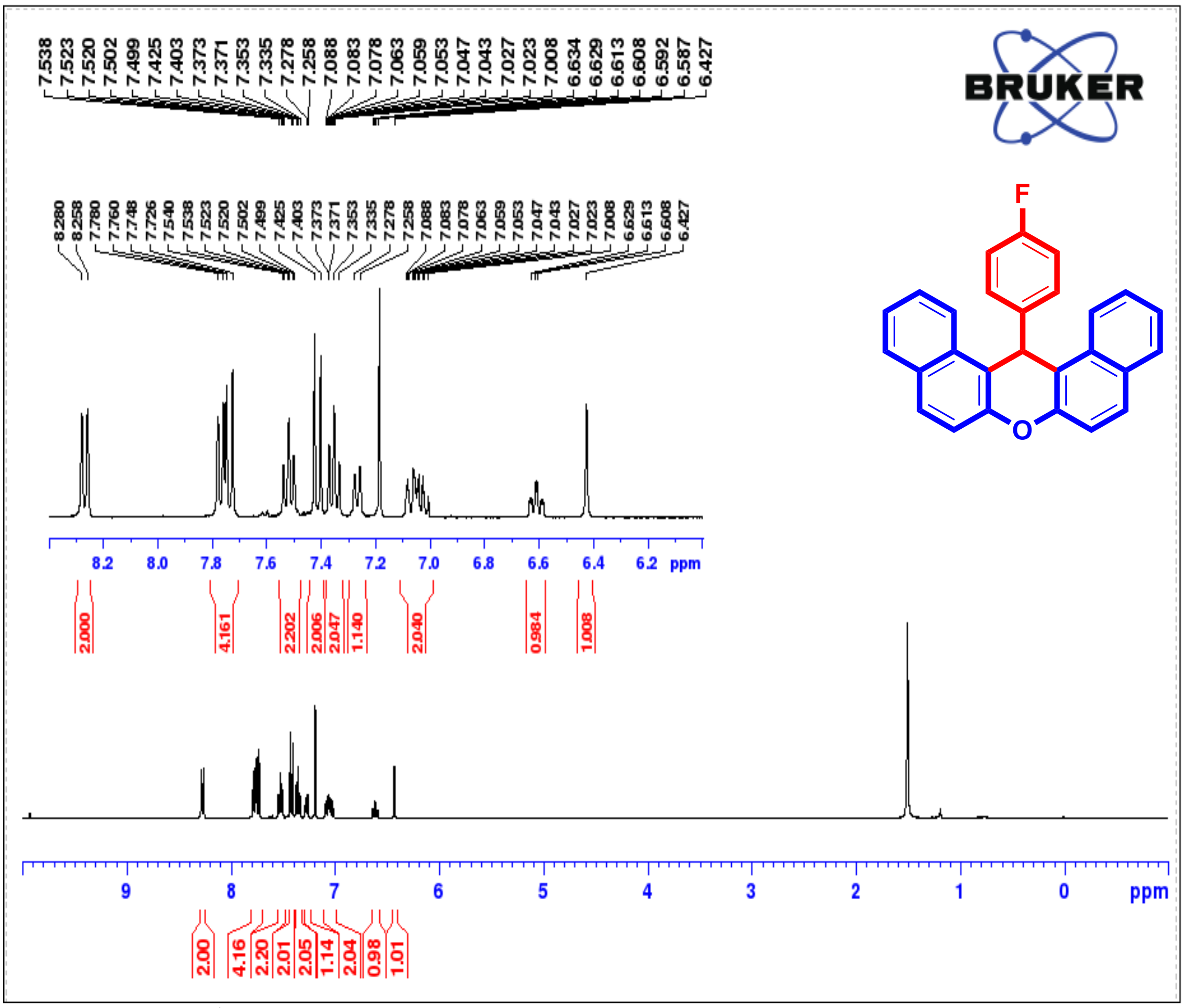

Fig. S8. ^{1}H NMR spectrum (400 MHz) of compound 6b in $CDCl_3$

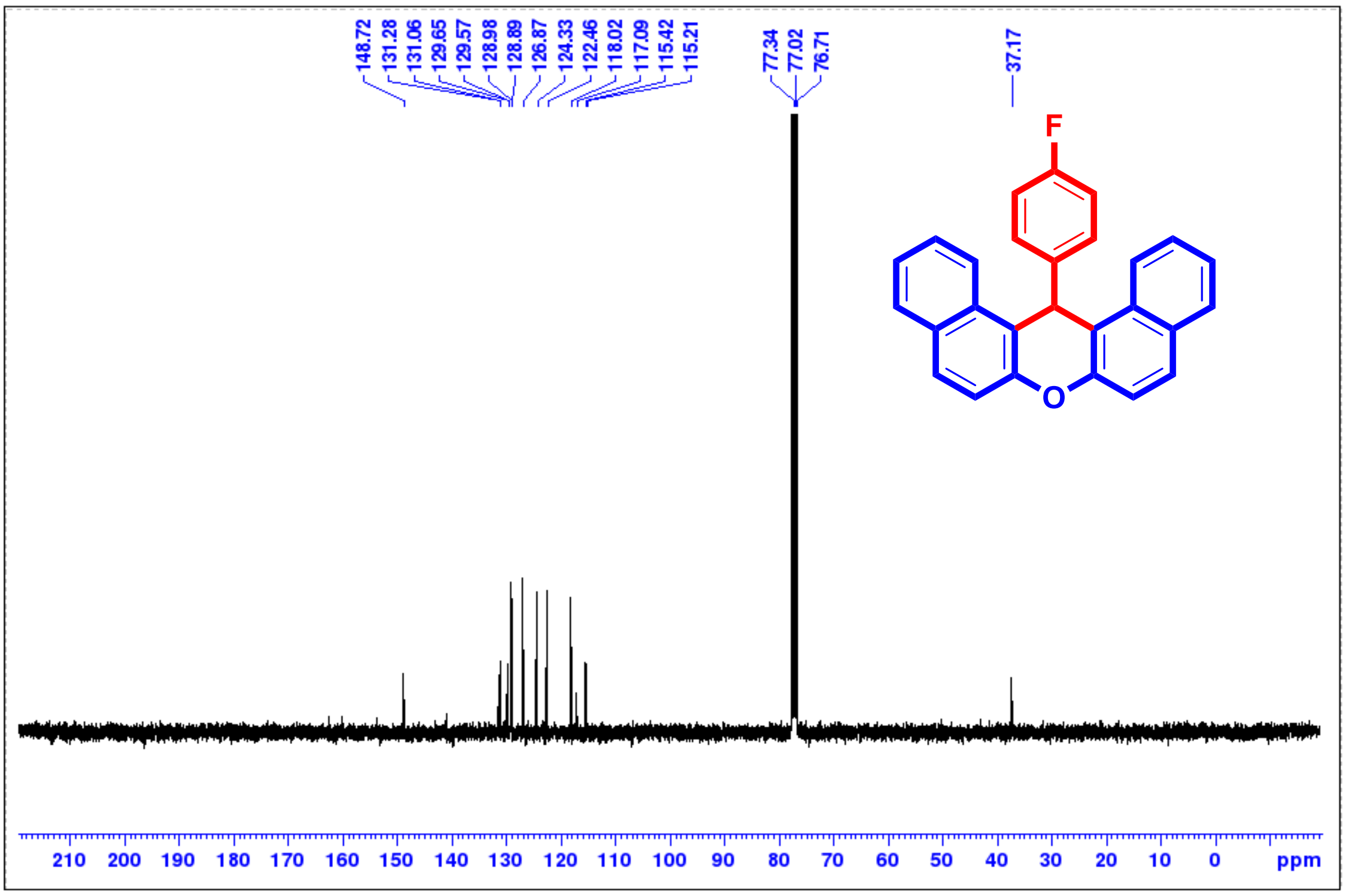

Fig. S9. ^{13}C NMR spectrum (100 MHz) of compound 6b in $CDCl_3$

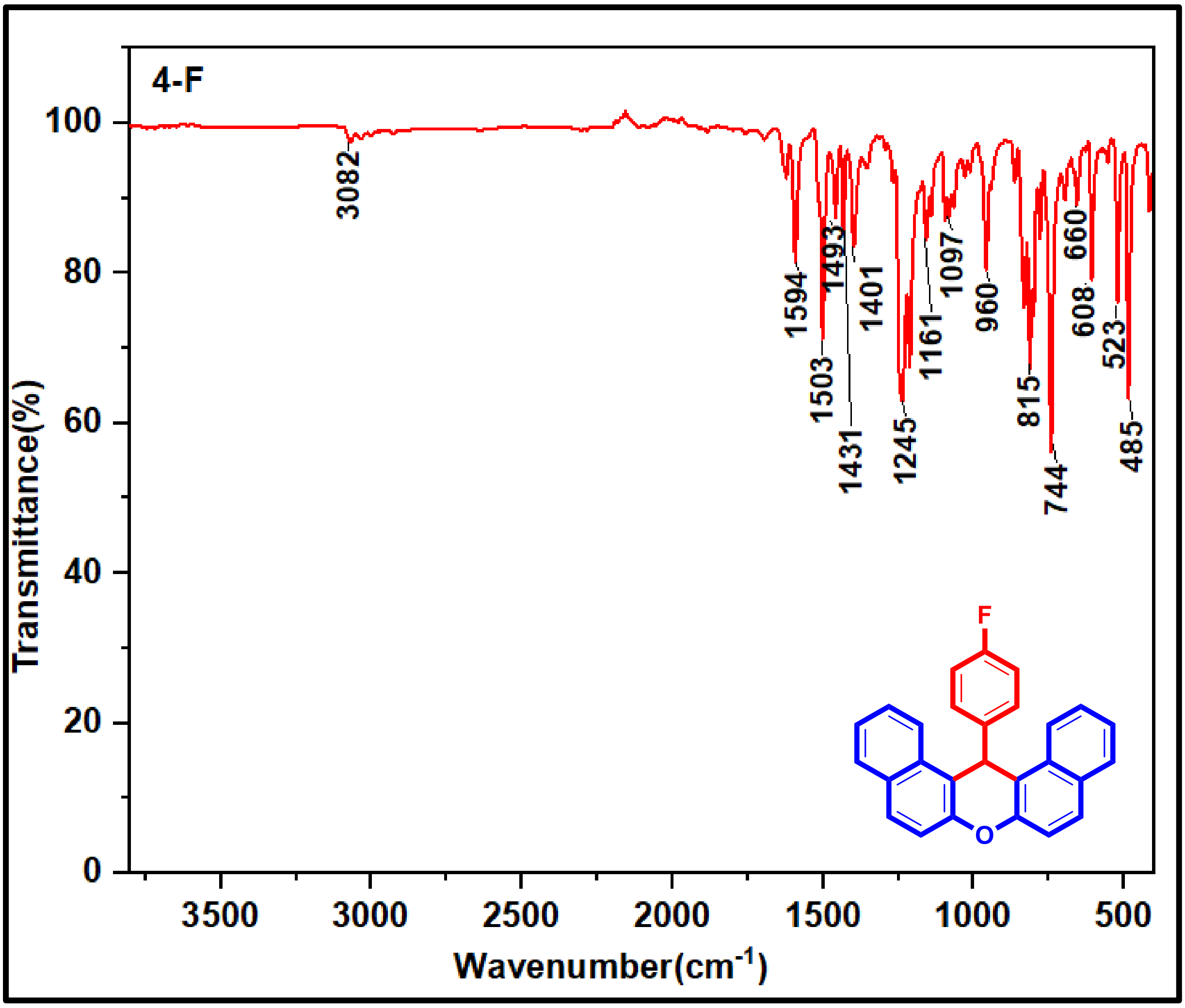

Fig. S10. FT-IR spectrum of compound 6b

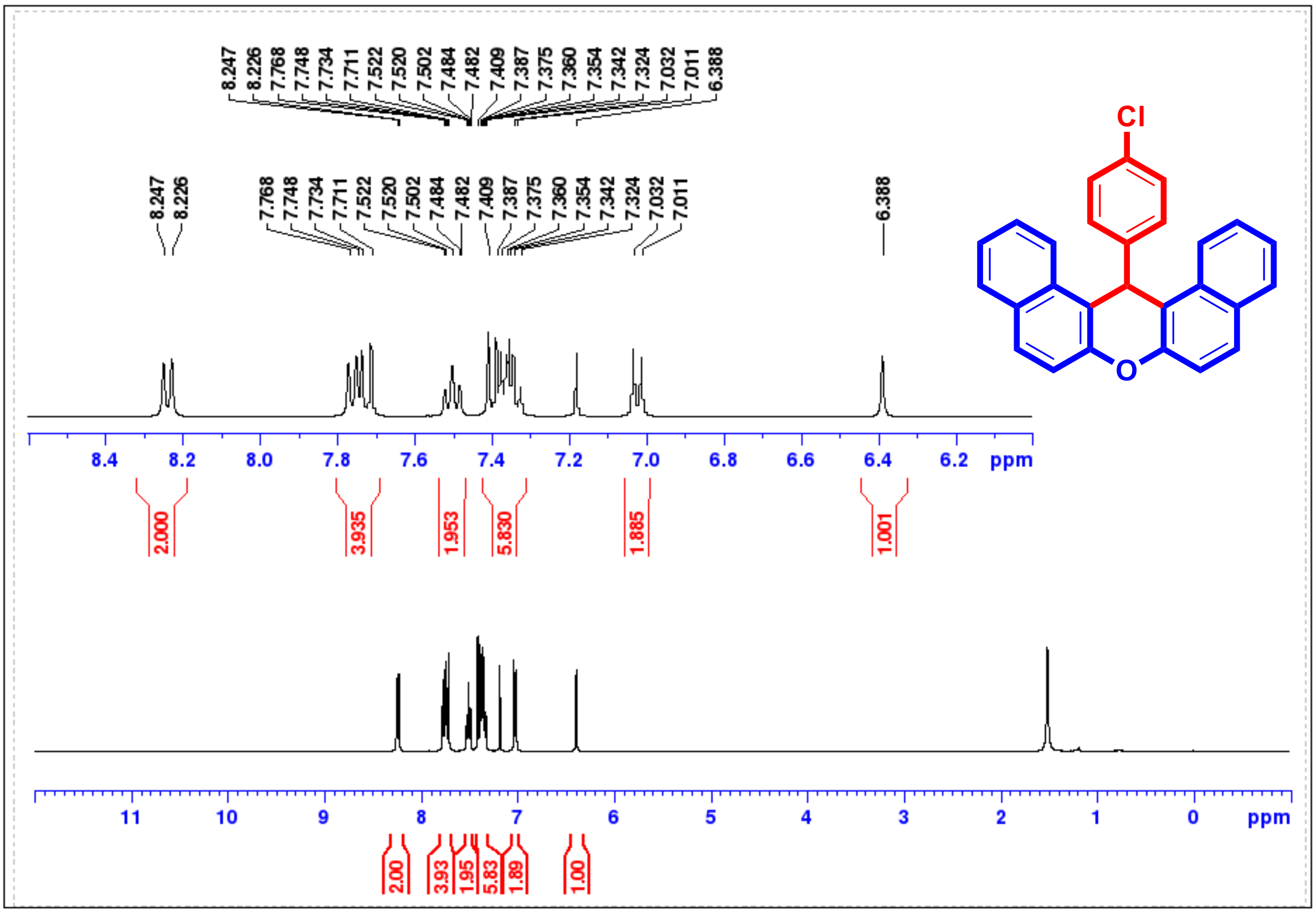

Fig. S11. ^{1}H NMR spectrum (400 MHz) of compound 6c in $CDCl_3$

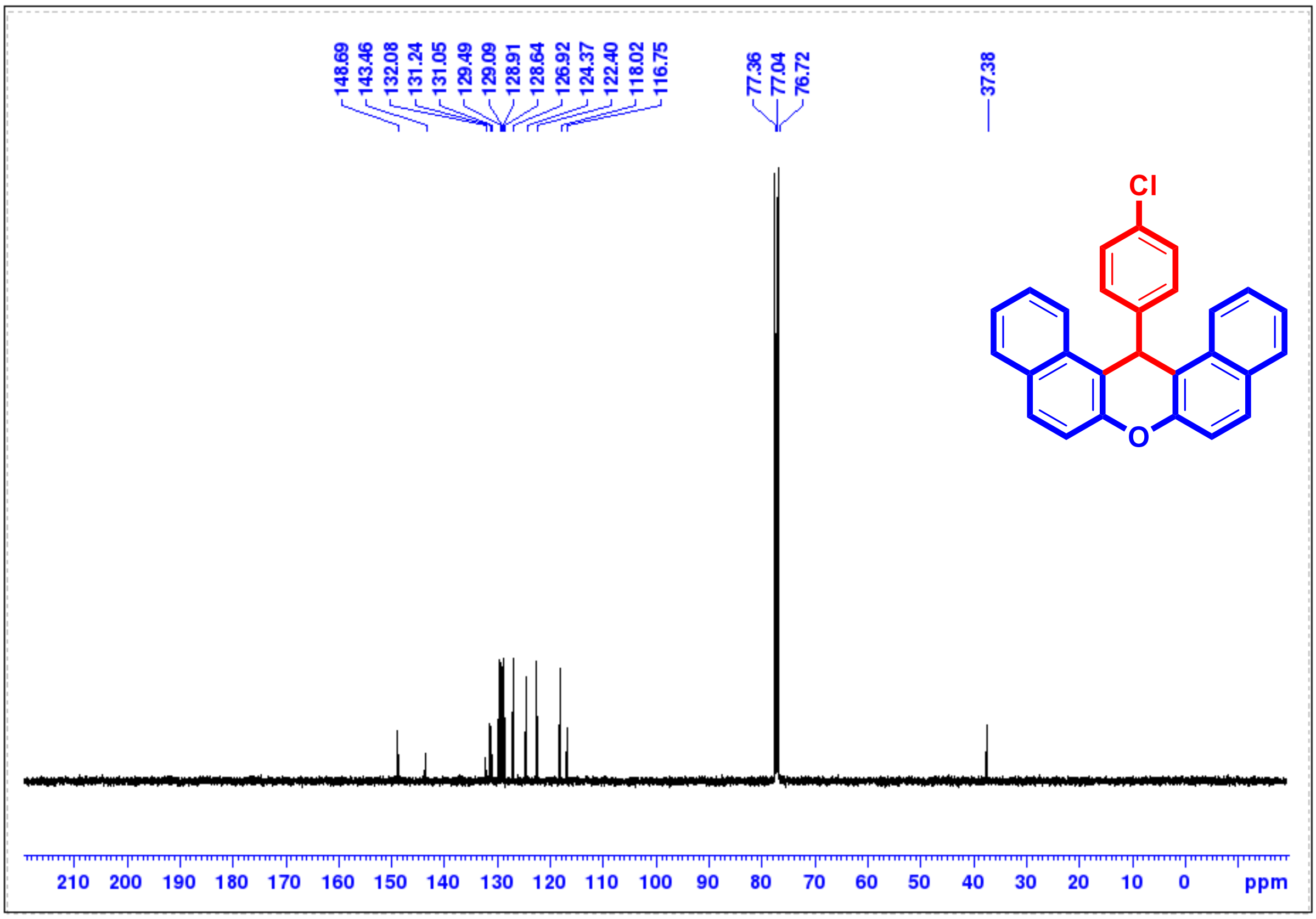

Fig. S12. ^{13}C NMR spectrum (100 MHz) of compound 6c in $CDCl_3$

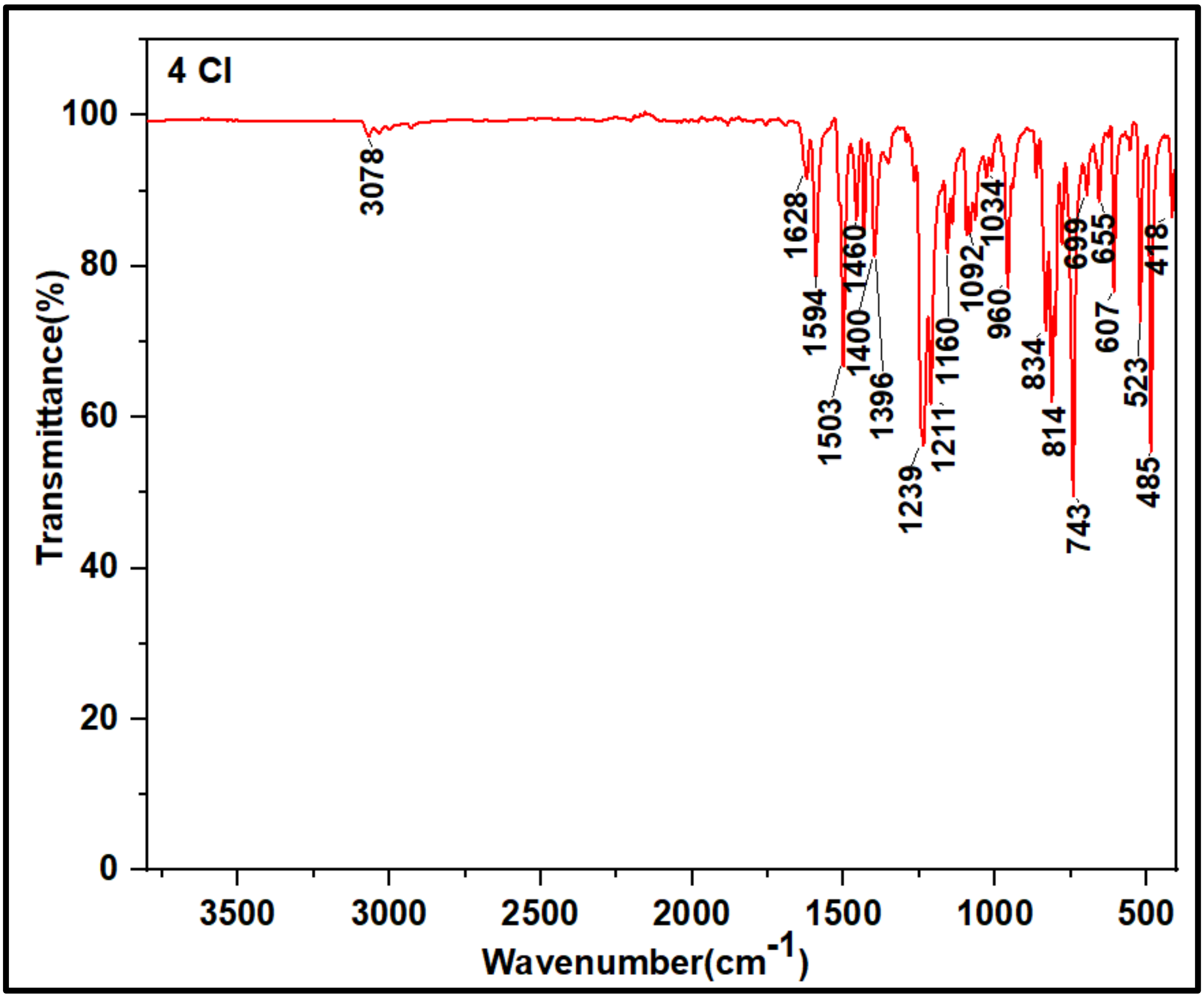

Fig. S13. FT-IR spectrum of compound 6c

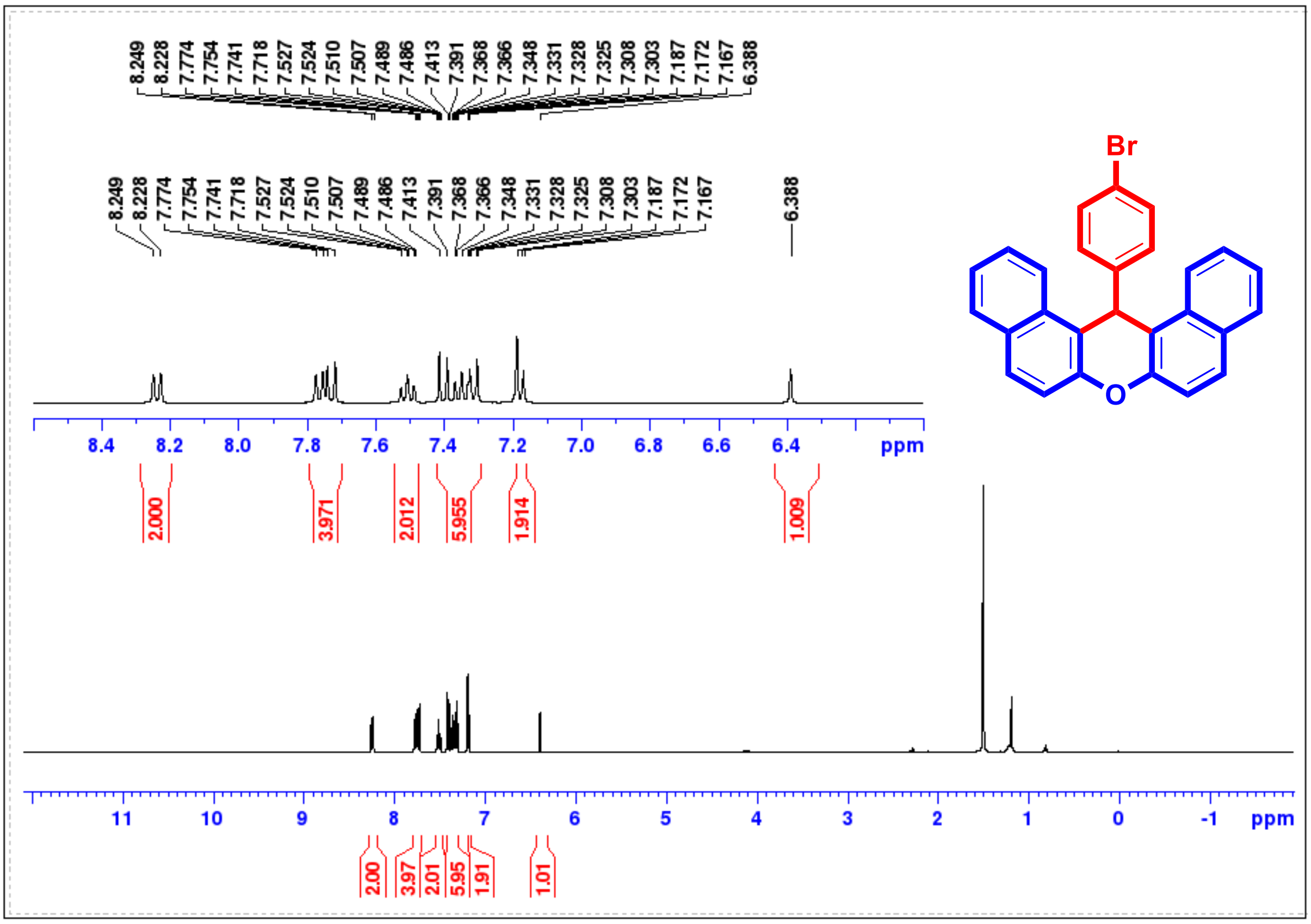

Fig. S14. ^{1}H NMR spectrum (400 MHz) of compound 6d in $CDCl_3$

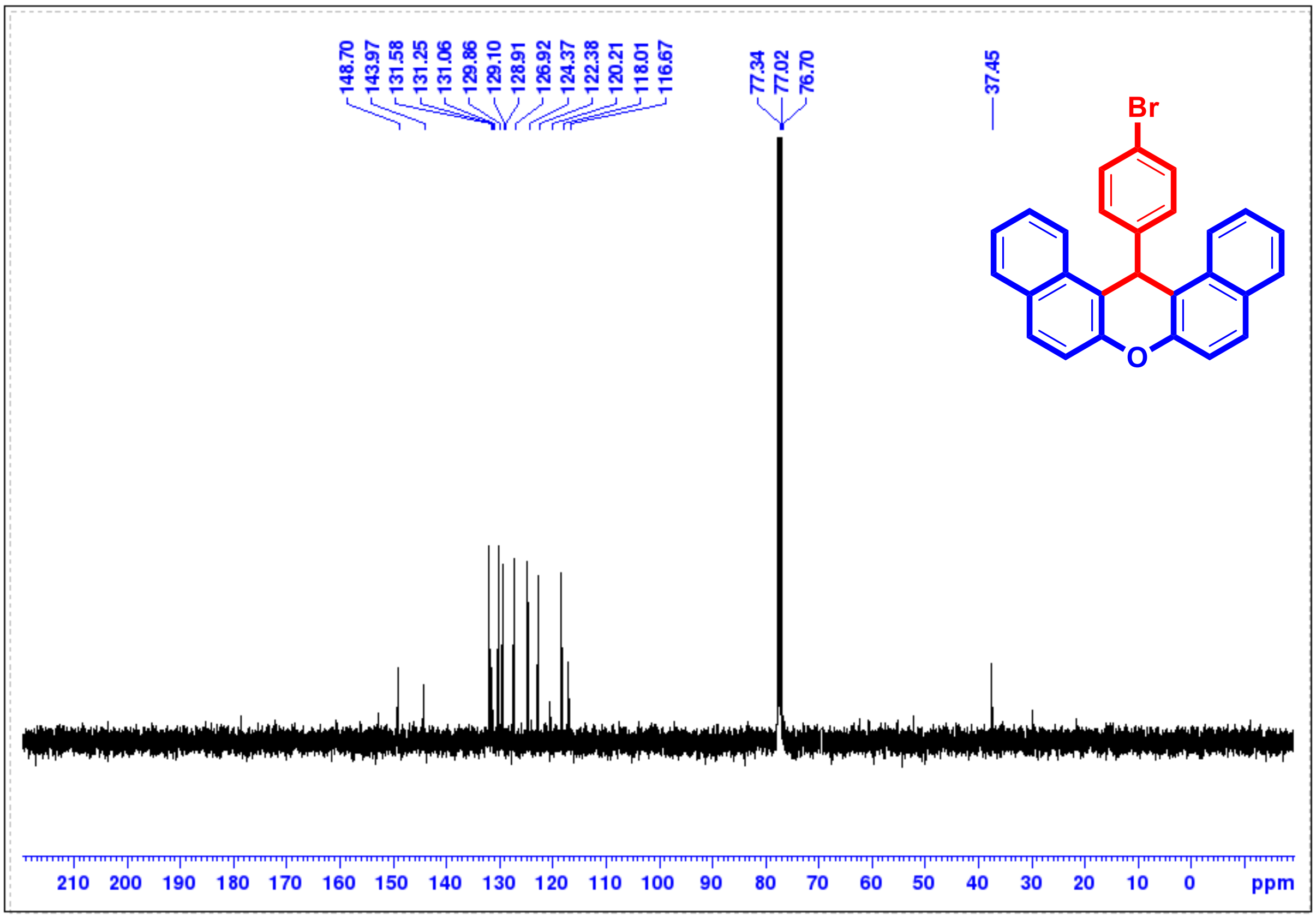

Fig. S15. ^{13}C NMR spectrum (100 MHz) of compound 6d in $CDCl_3$

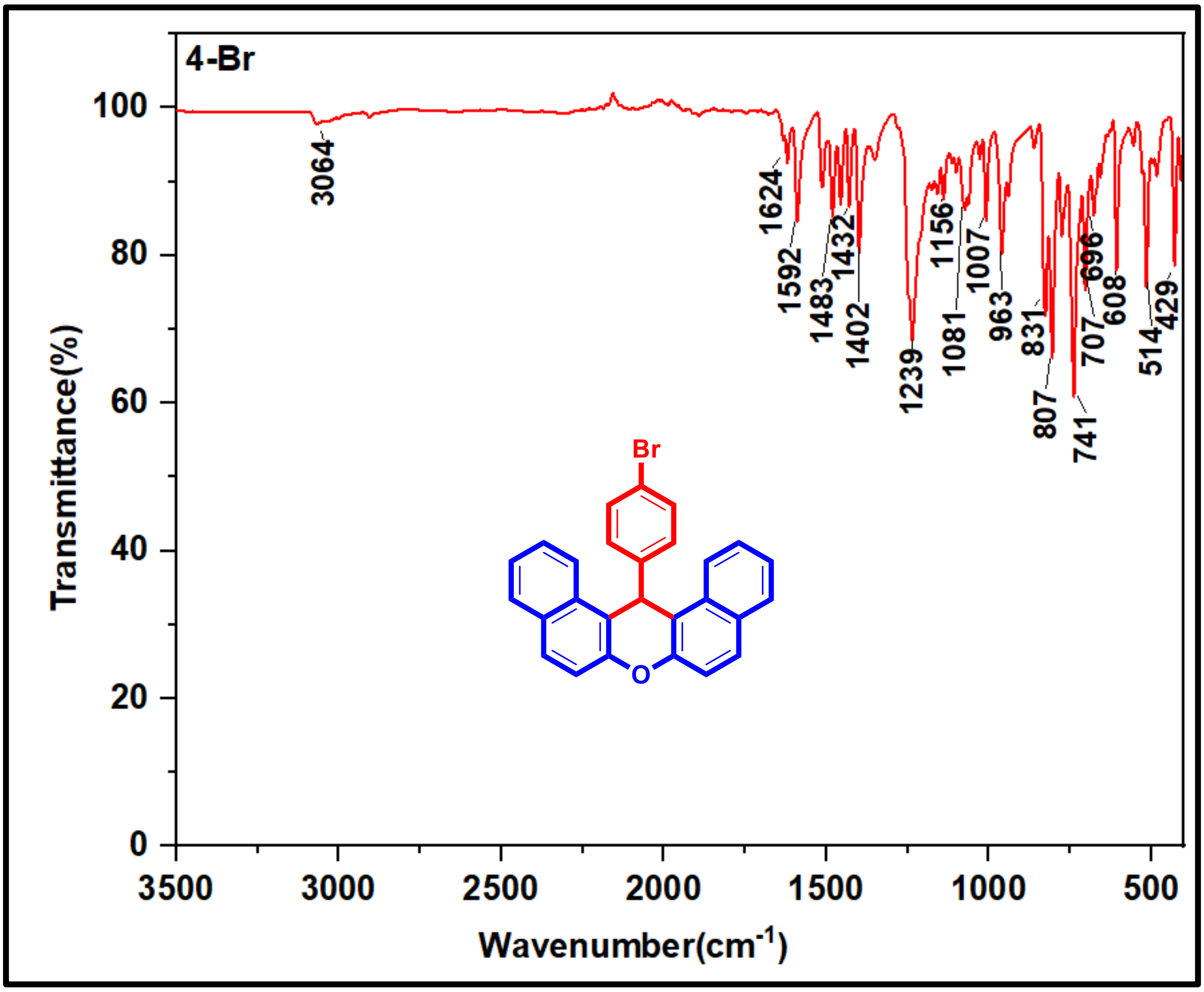

Fig. S16. FT-IR spectrum of compound 6d

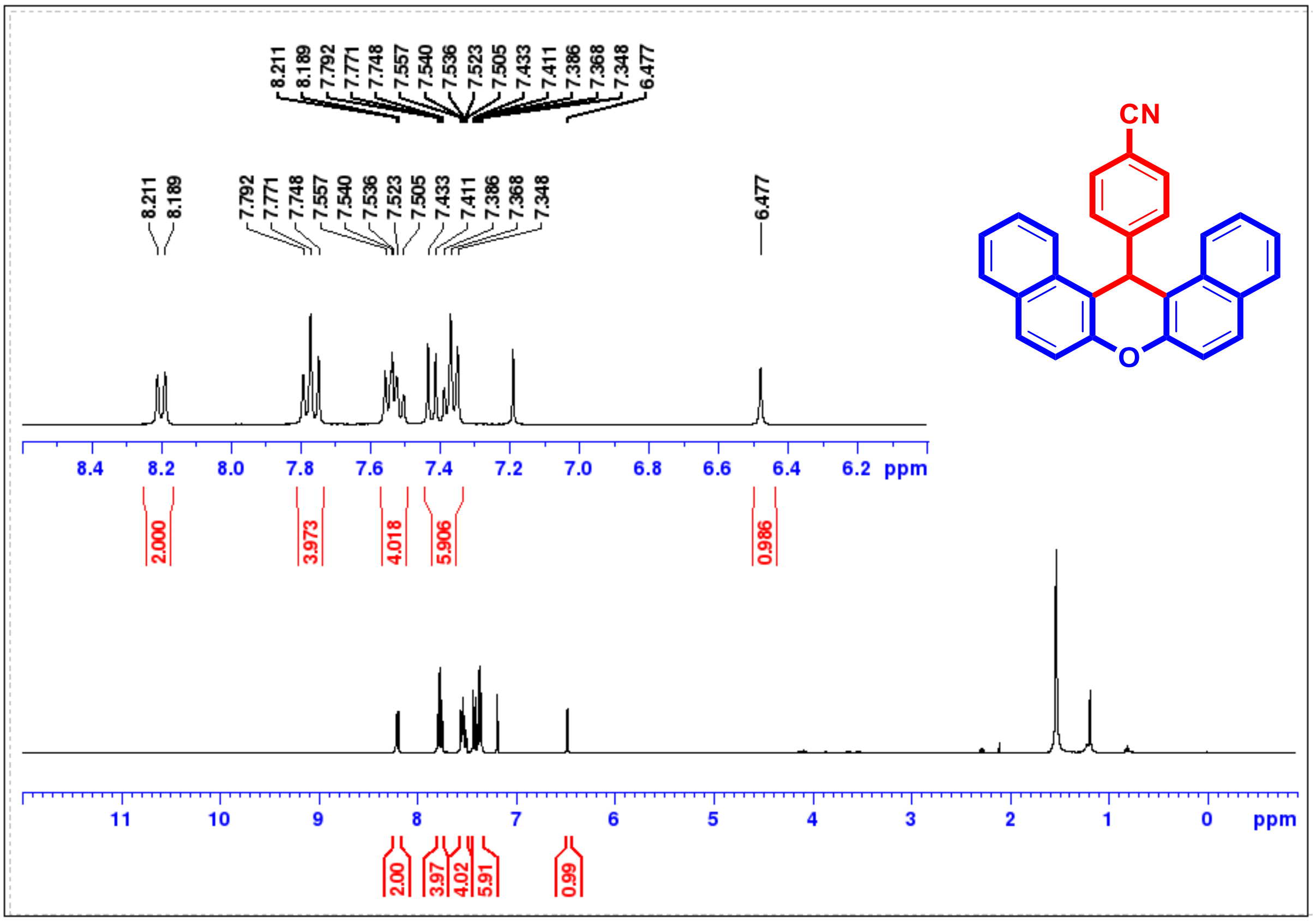

Fig. S17. ^{1}H NMR spectrum (400 MHz) of compound 6e in $CDCl_3$

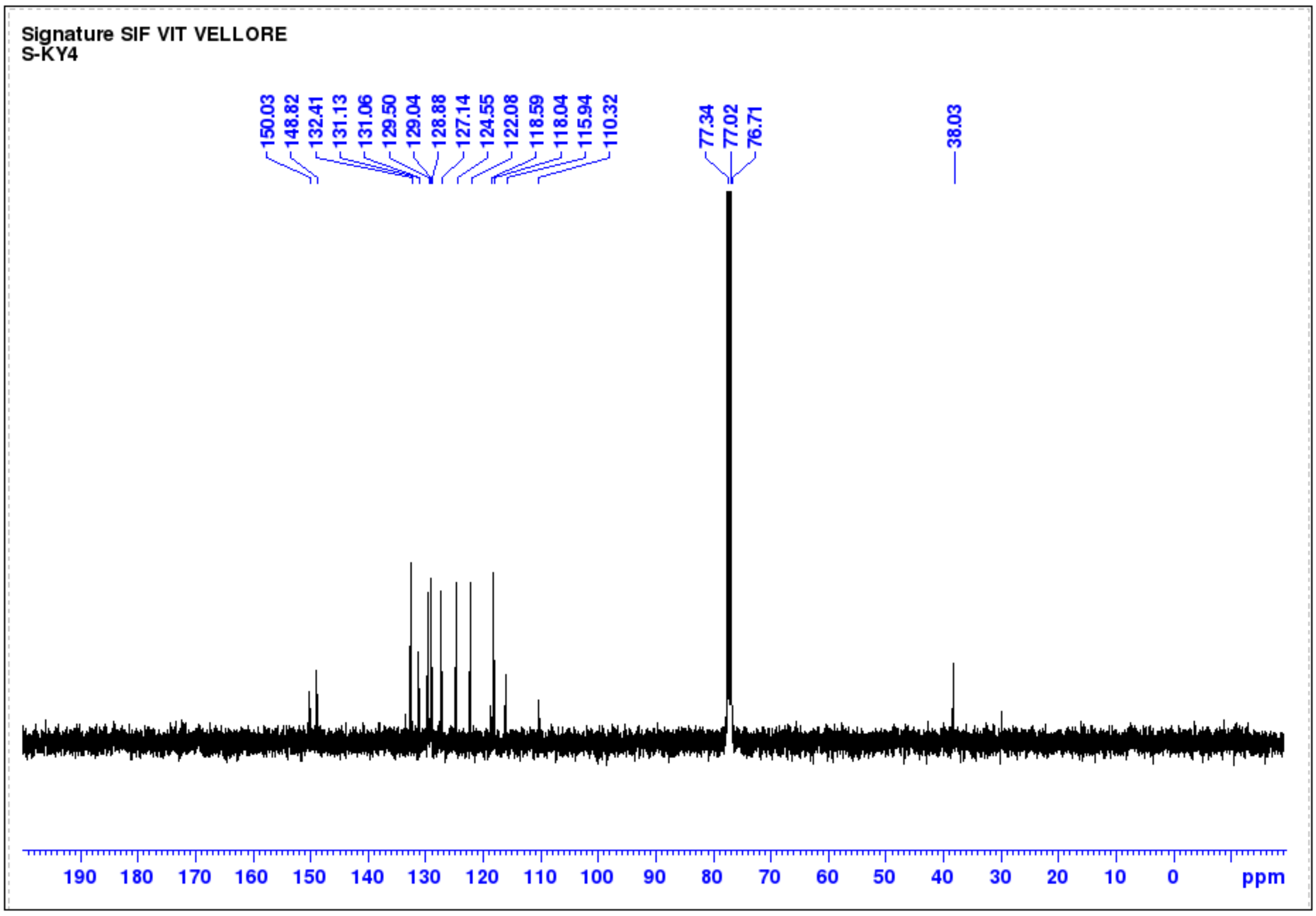

Fig. S18. ^{13}C NMR spectrum (100 MHz) of compound 6e in $CDCl_3$

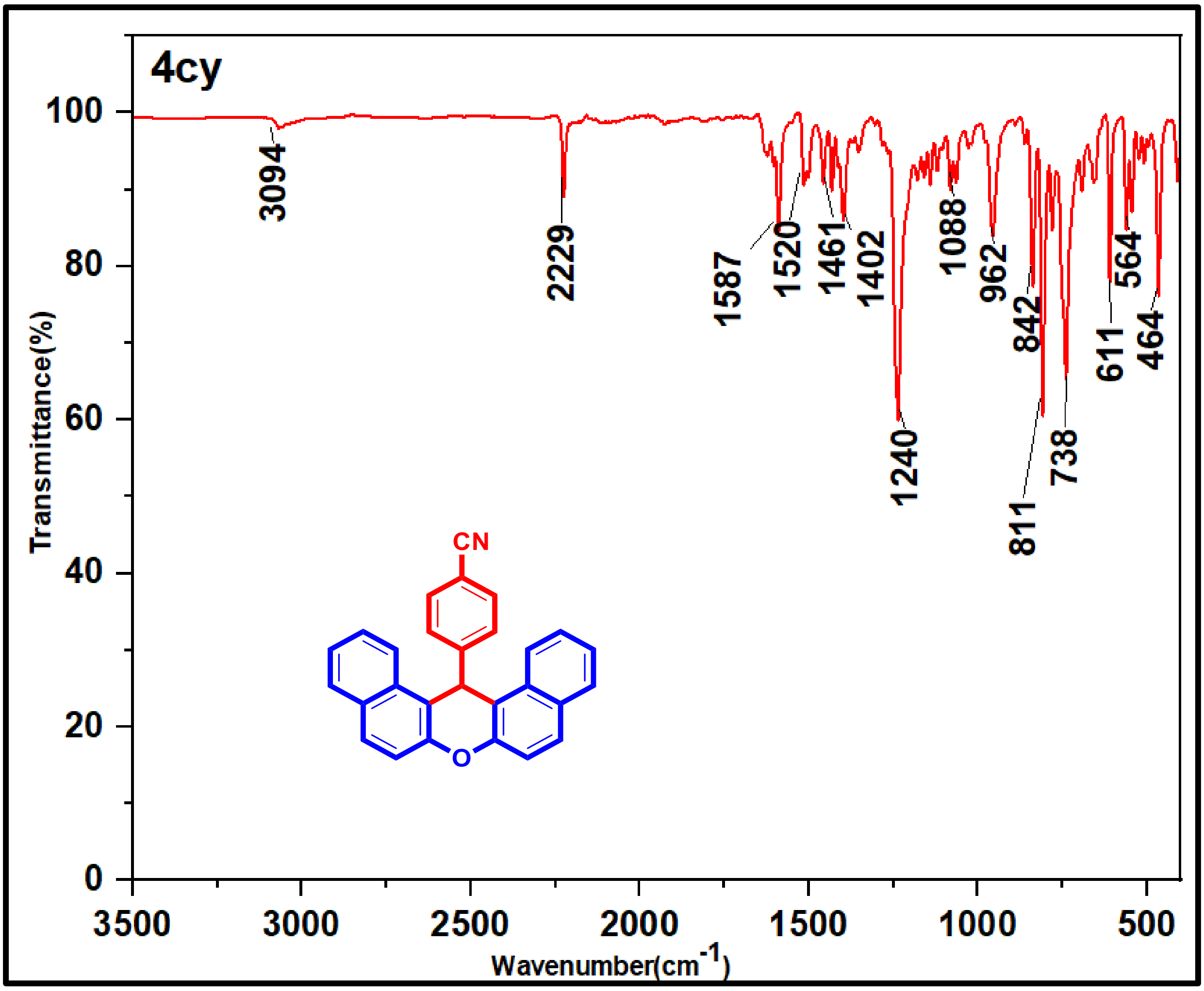

Fig. S19. FT-IR spectrum of compound 6e

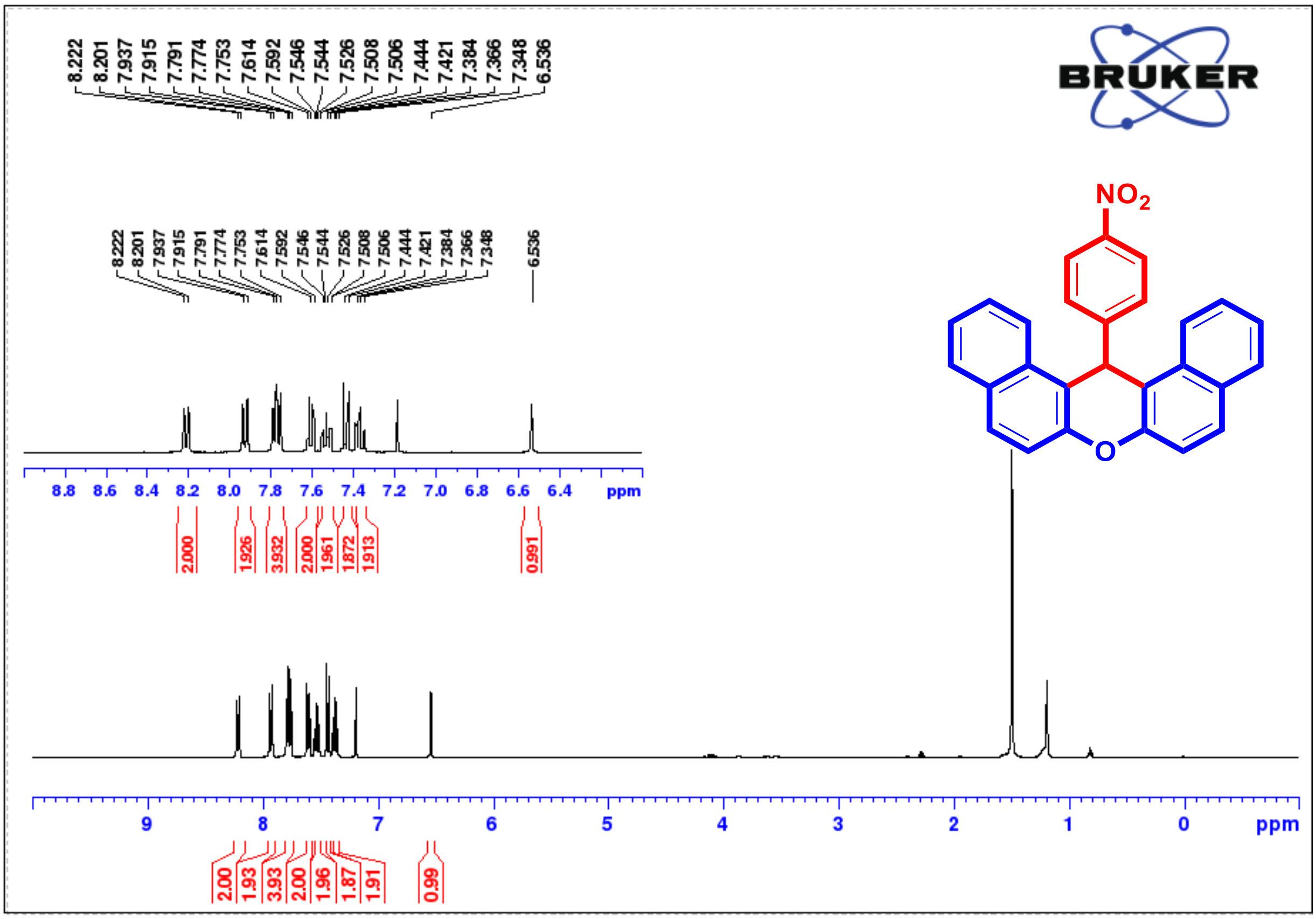

Fig. S20. ^{1}H NMR spectrum (400 MHz) of compound 6f in $CDCl_3$

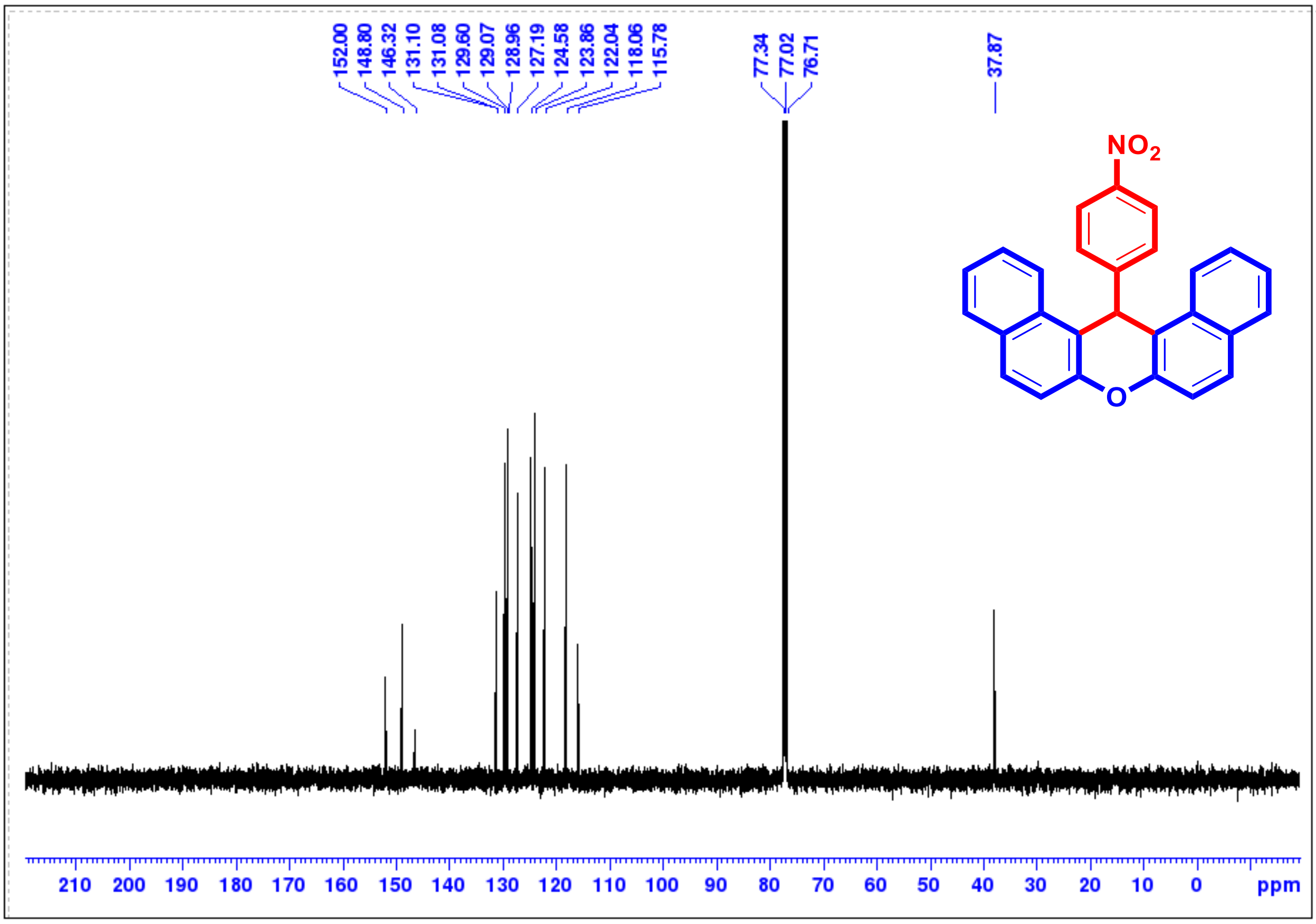

Fig. S21. ^{13}C NMR spectrum (100 MHz) of compound 6f in $CDCl_3$

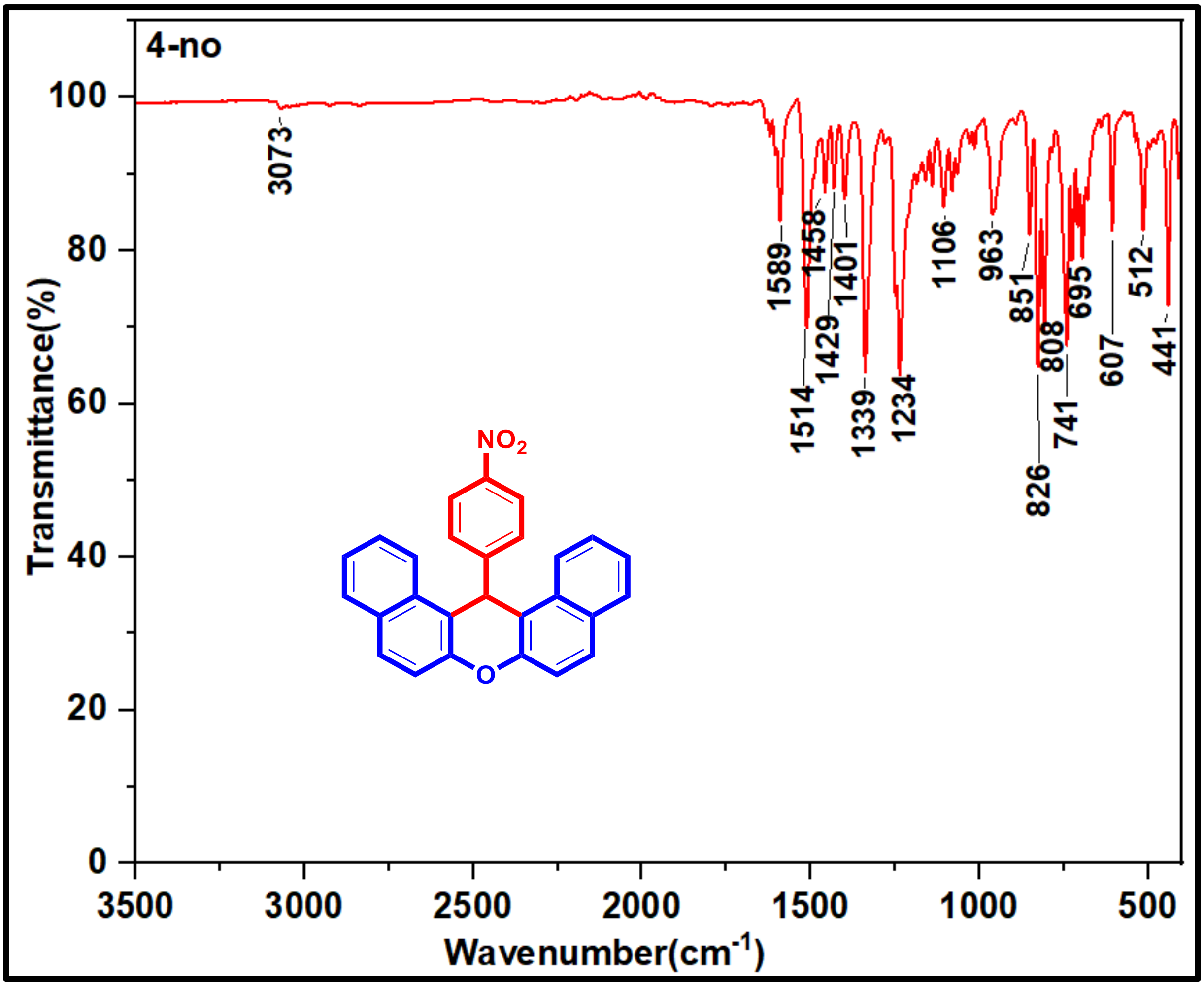

Fig. S22. FT-IR spectrum of compound 6f

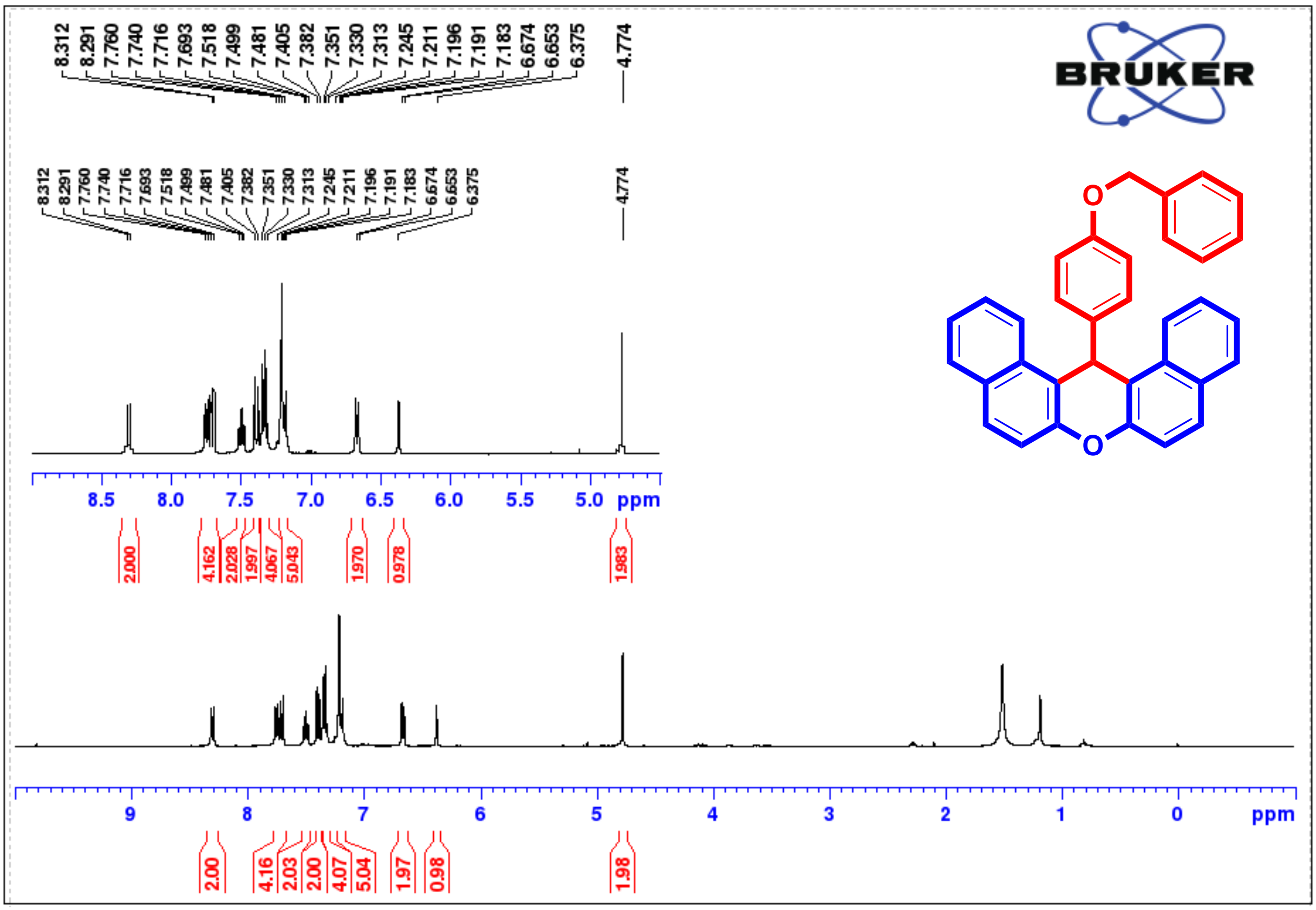

Fig. S23. ^{1}H NMR spectrum (400 MHz) of compound 6g in $CDCl_3$

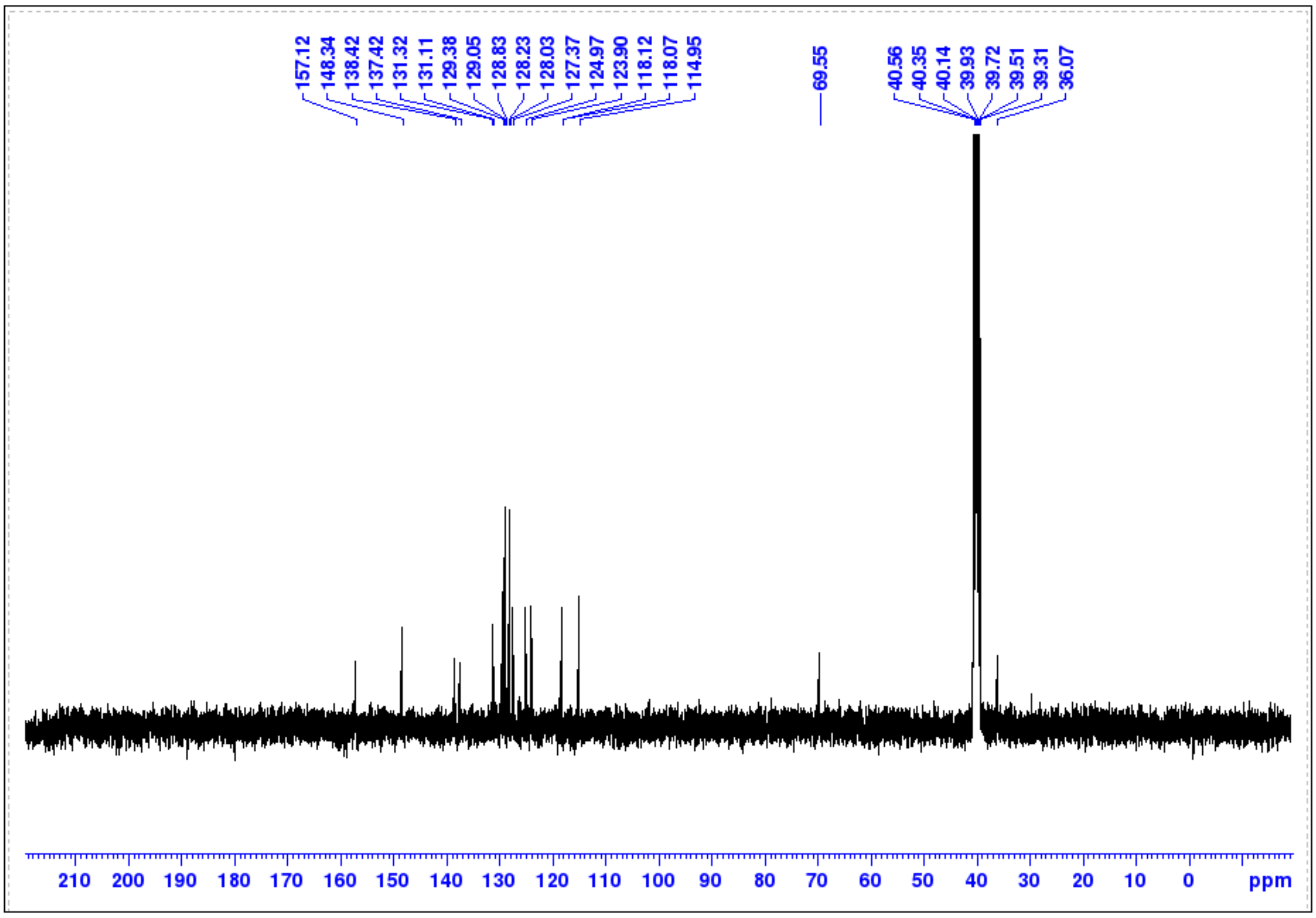

Fig. S24. ^{13}C NMR spectrum (100 MHz) of compound 6g in $CDCl_3$

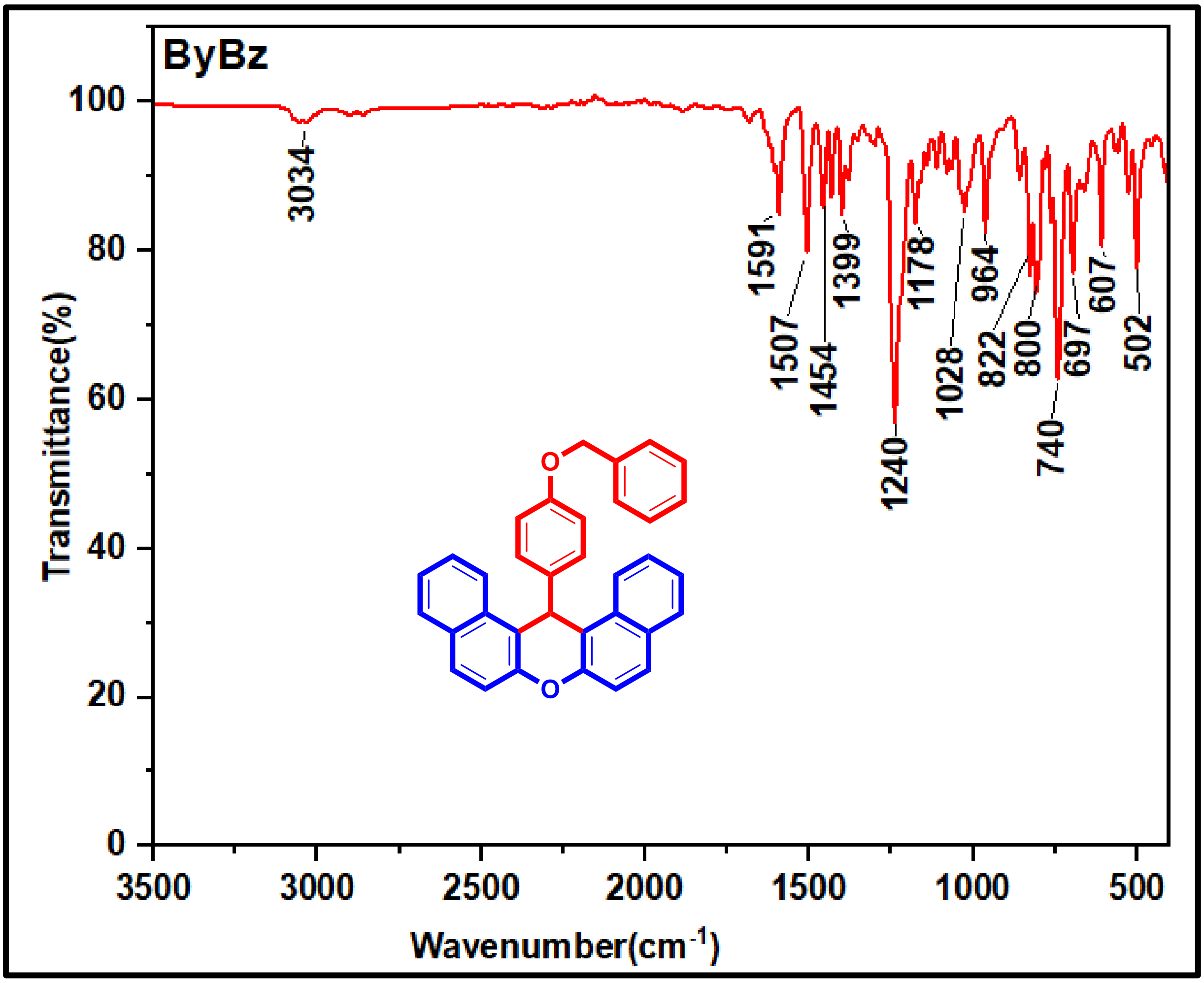

Fig. S25. FT-IR spectrum of compound 6g

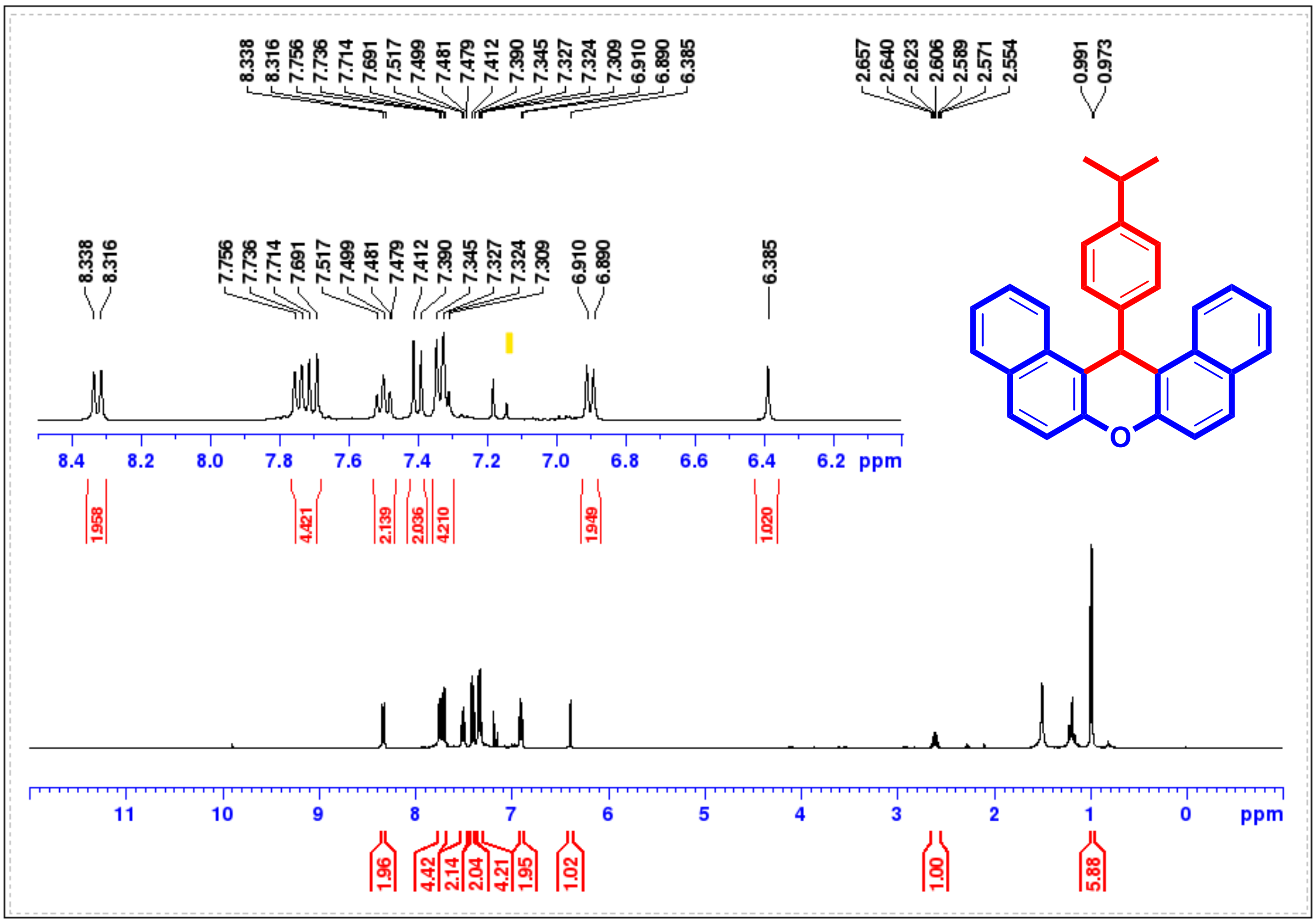

Fig. S26. ¹H NMR spectrum (400 MHz) of compound **6h** in $CDCl_3$

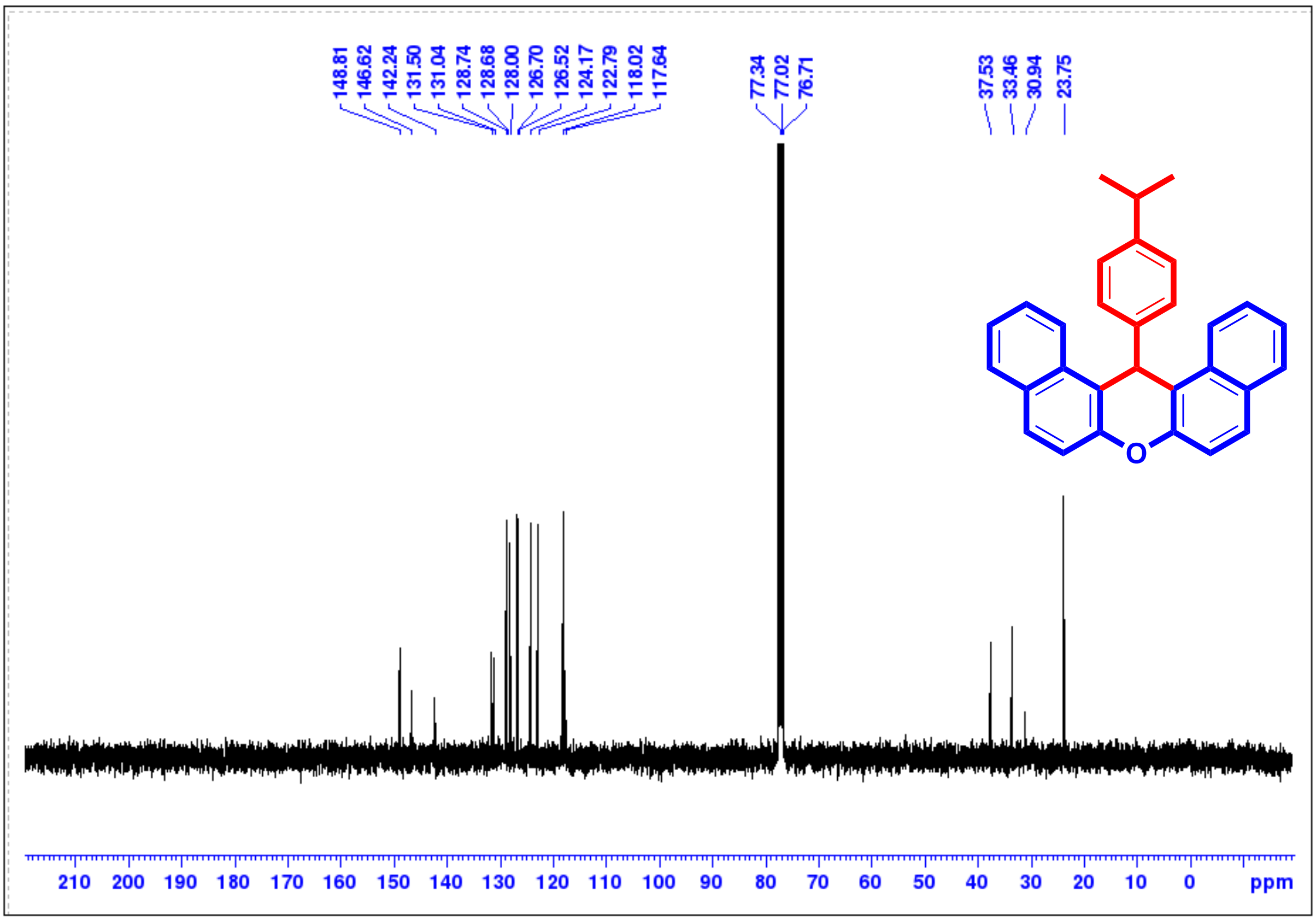

Fig. S27. ¹³C NMR spectrum (100 MHz) of compound **6h** in $CDCl_3$

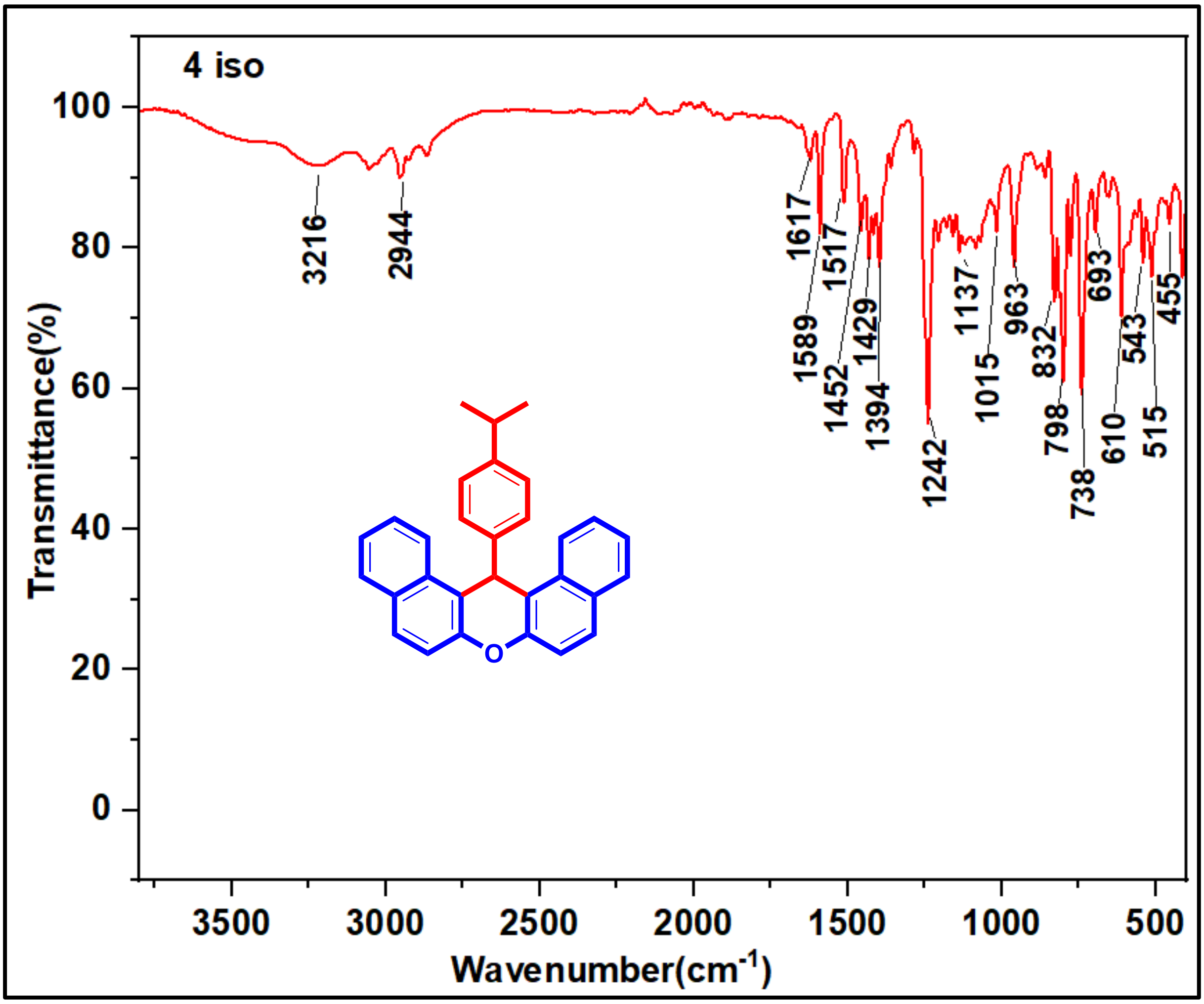

Fig. S28. FT-IR spectrum of compound 6h

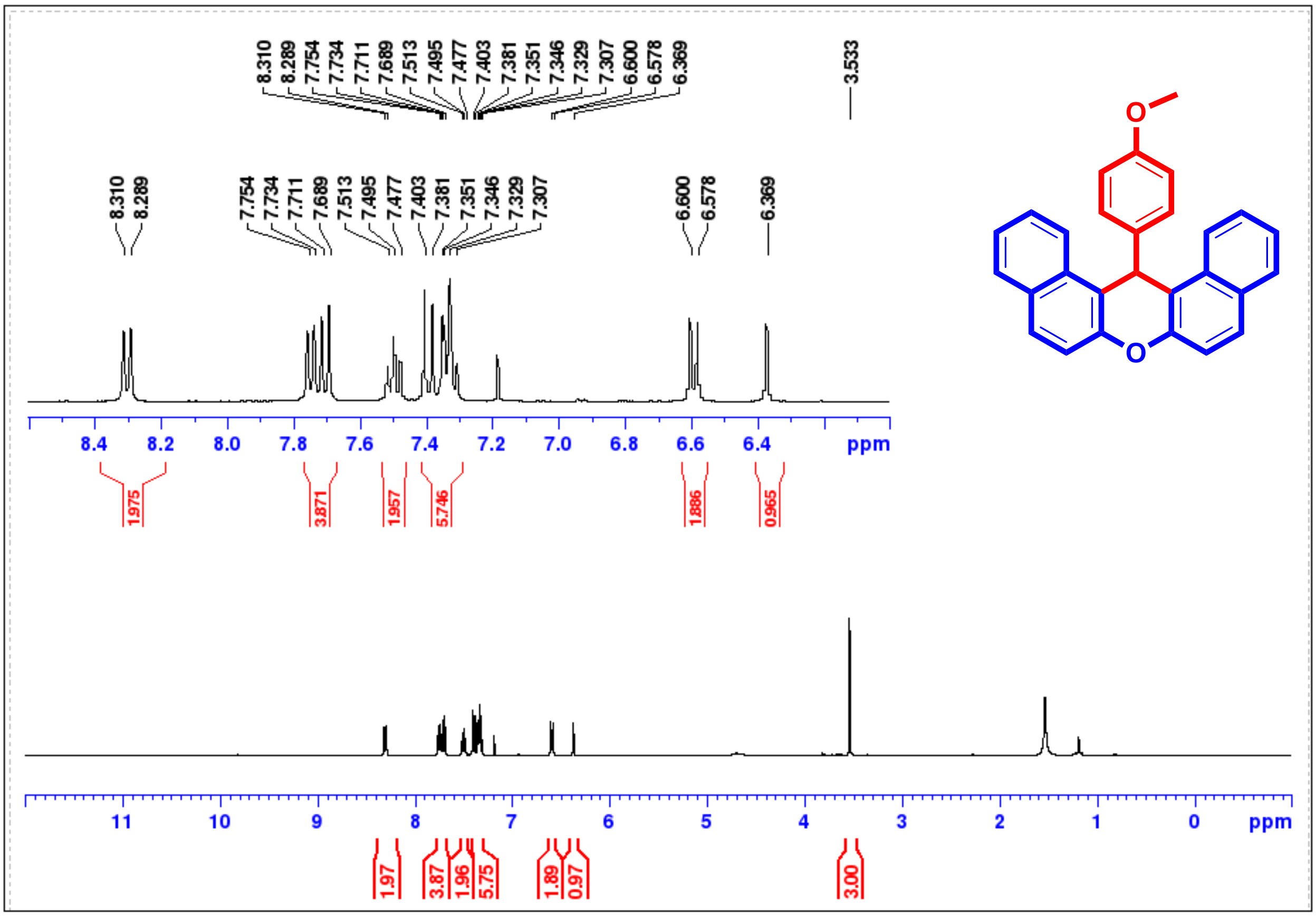

Fig. S29. ^{1}H NMR spectrum (400 MHz) of compound 6i in $CDCl_3$

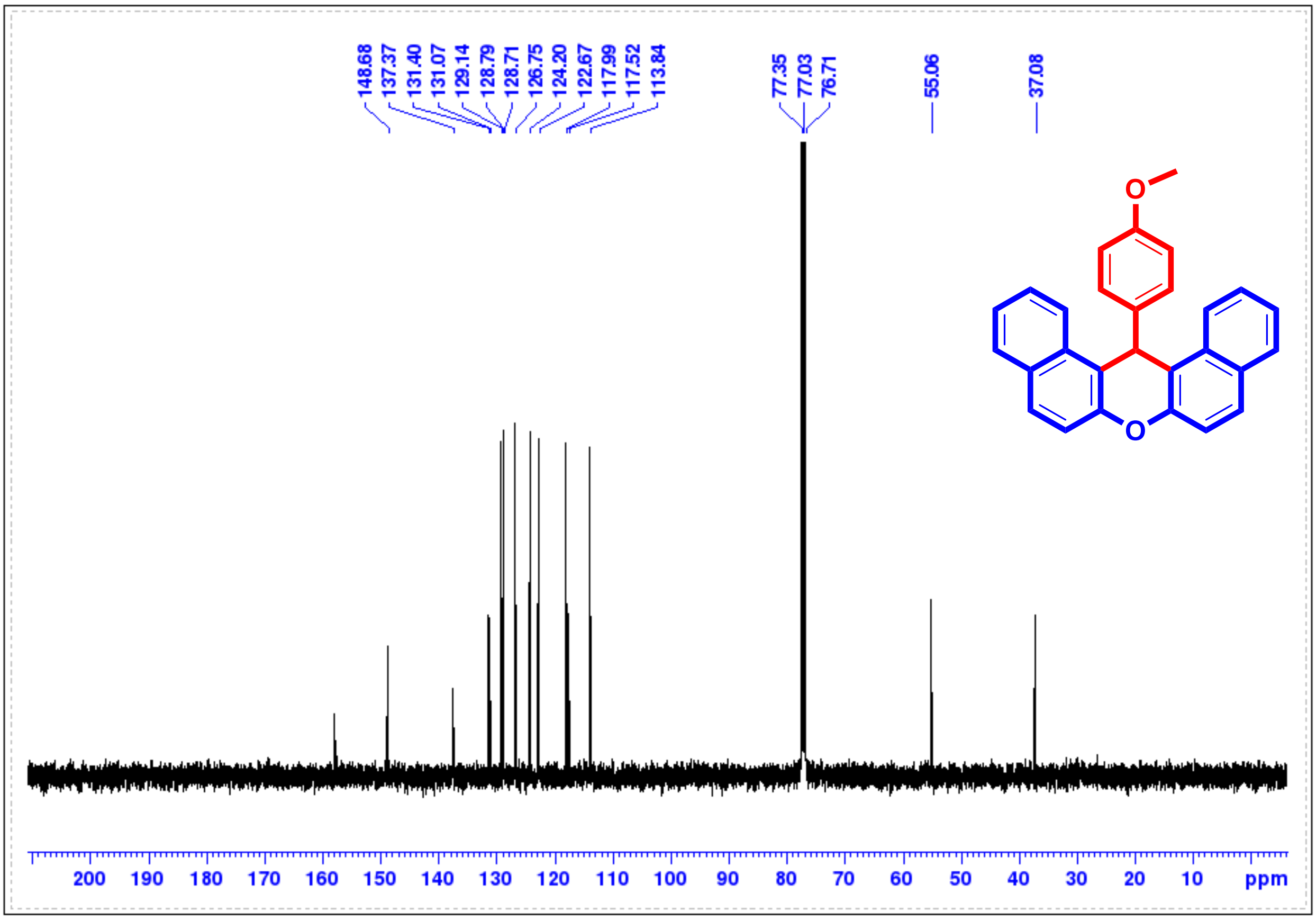

Fig. S30. ^{13}C NMR spectrum (100 MHz) of compound 6i in $CDCl_3$

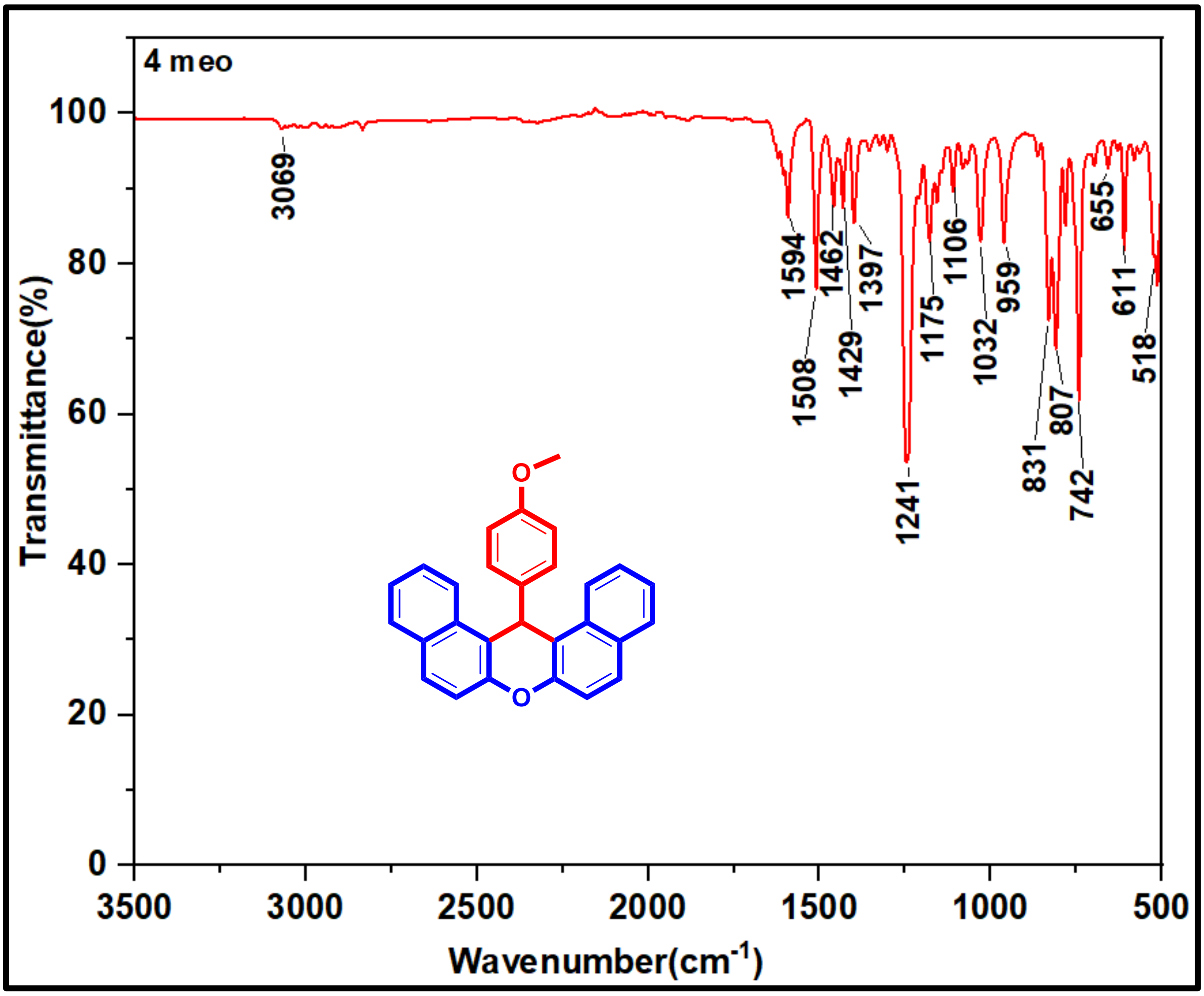

Fig. S31. FT-IR spectrum of compound 6i

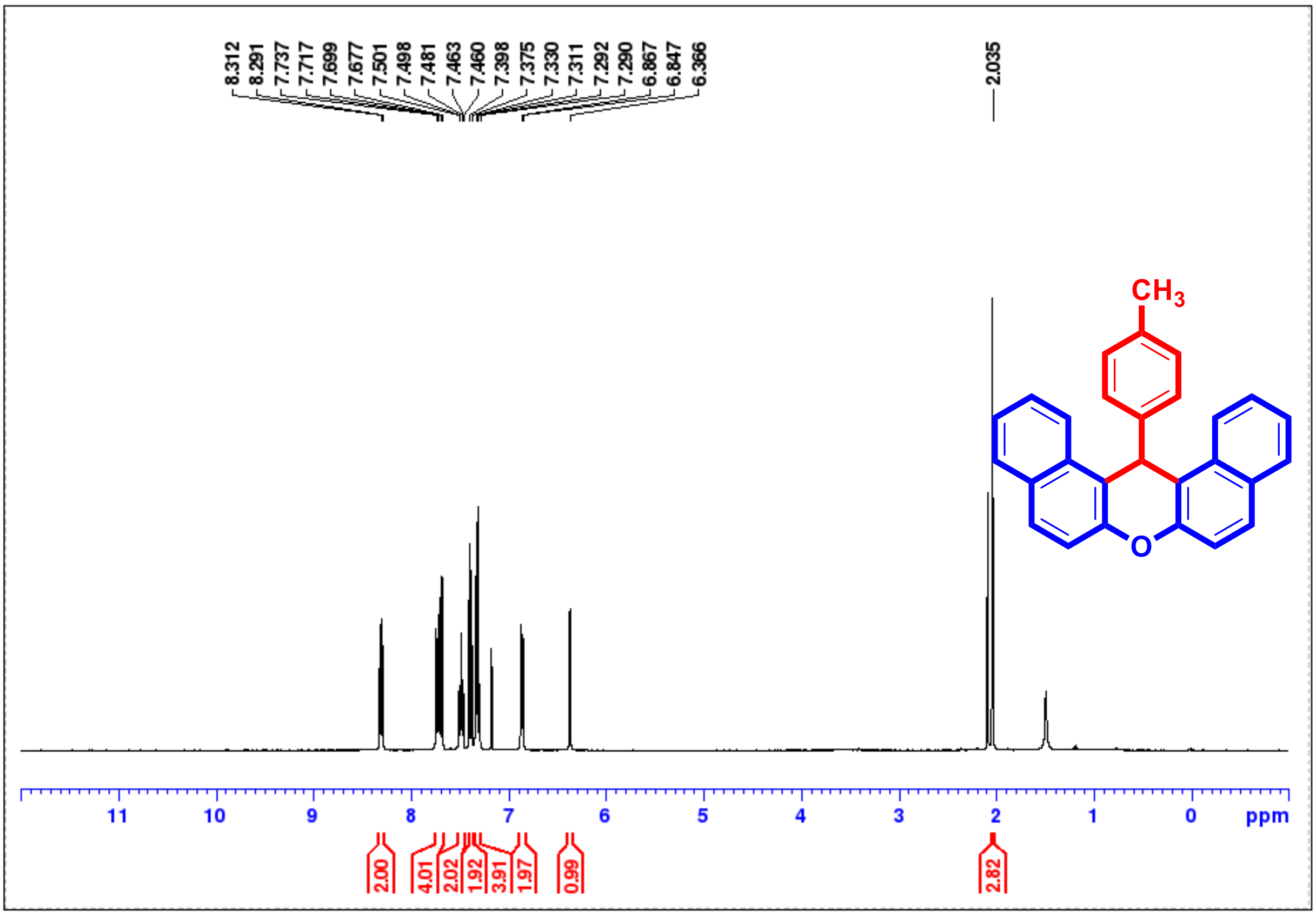

Fig. S32. [1]**H NMR spectrum (400 MHz) of compound 6j in CDCl3**

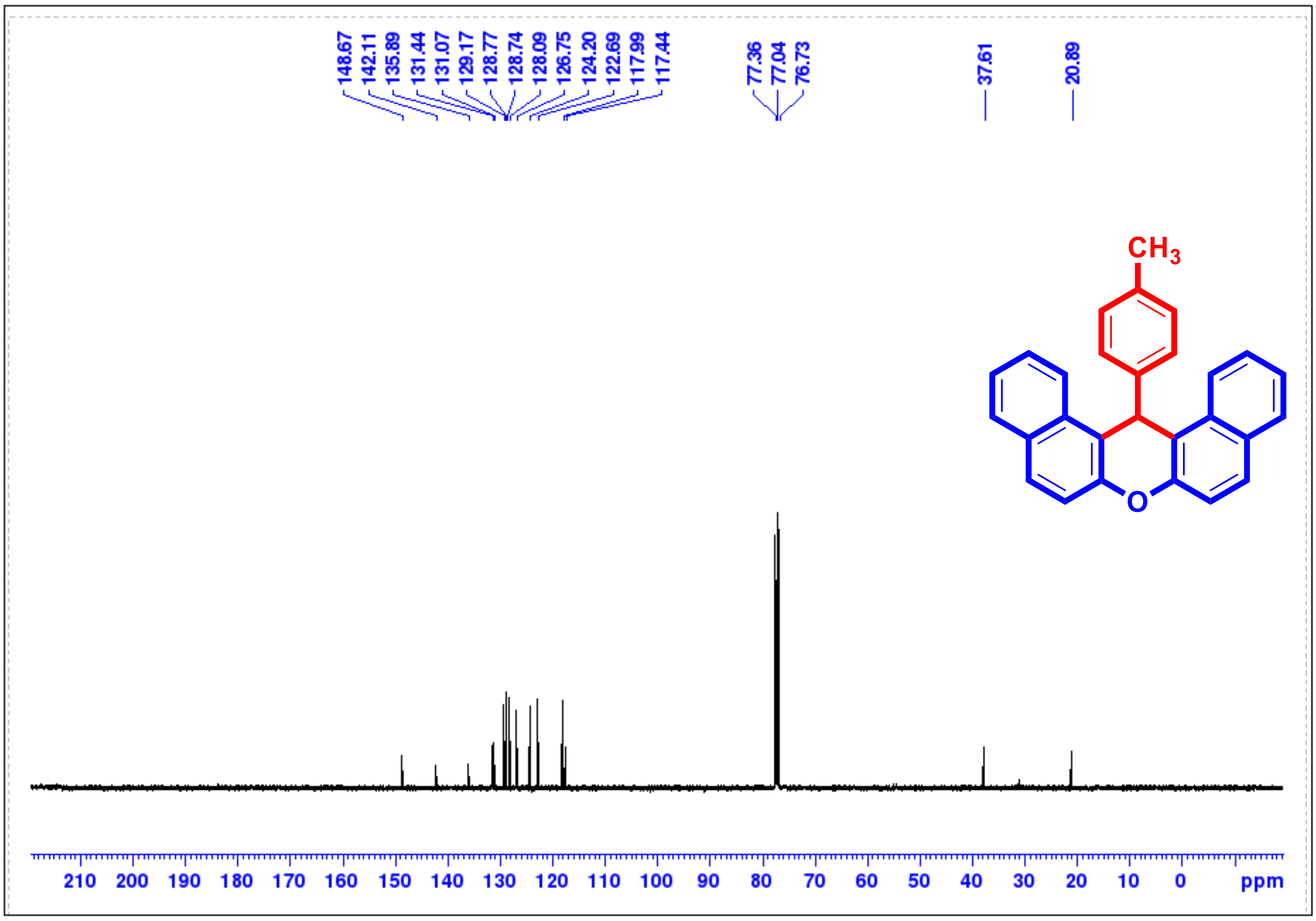

Fig. S33. [13]**C NMR spectrum (100 MHz) of compound 6j in CDCl3**

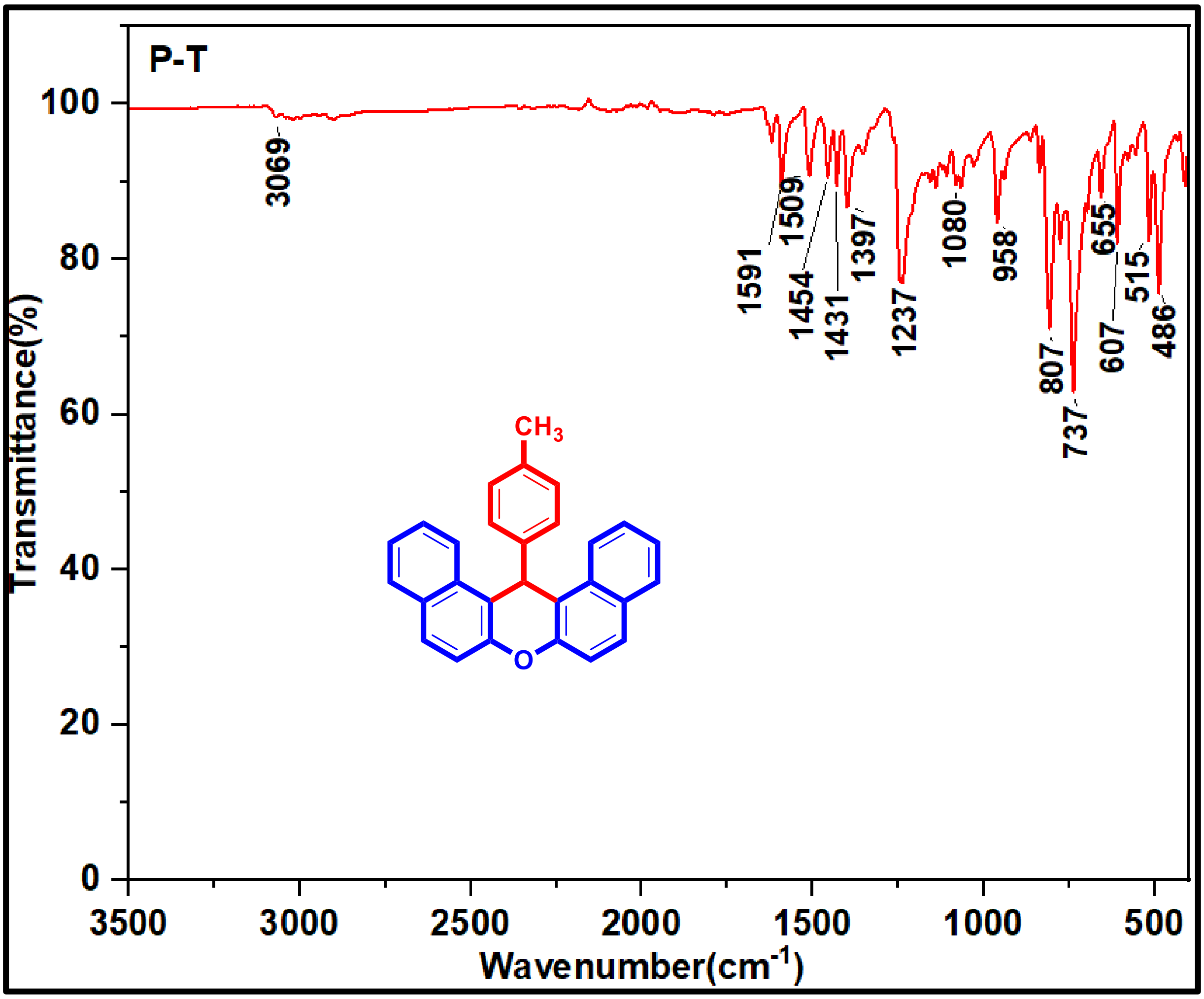

Fig. S34. FT-IR spectrum of compound 6j

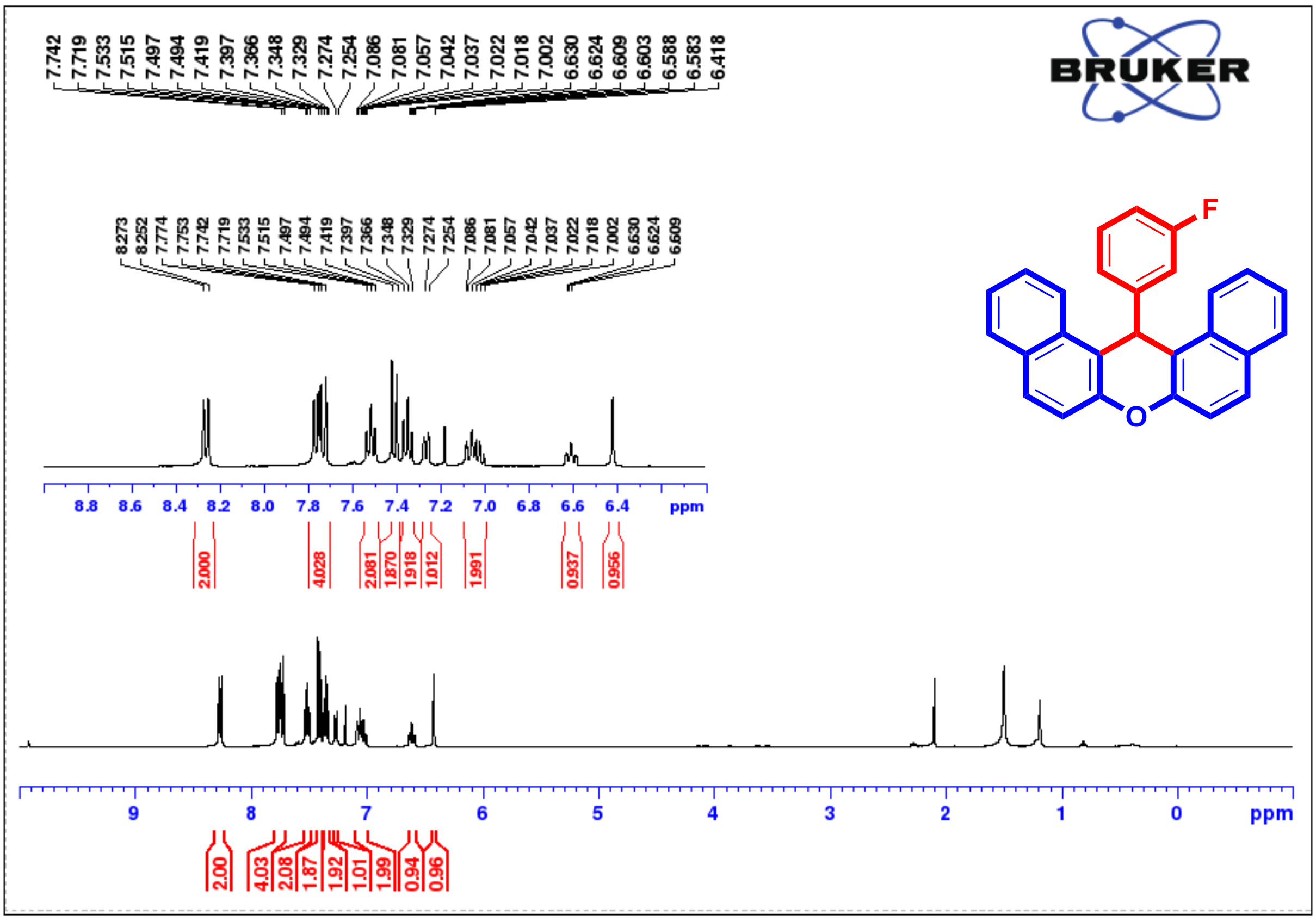

Fig. S35: ^{1}H NMR spectrum (400 MHz) of compound 6k in $CDCl_3$

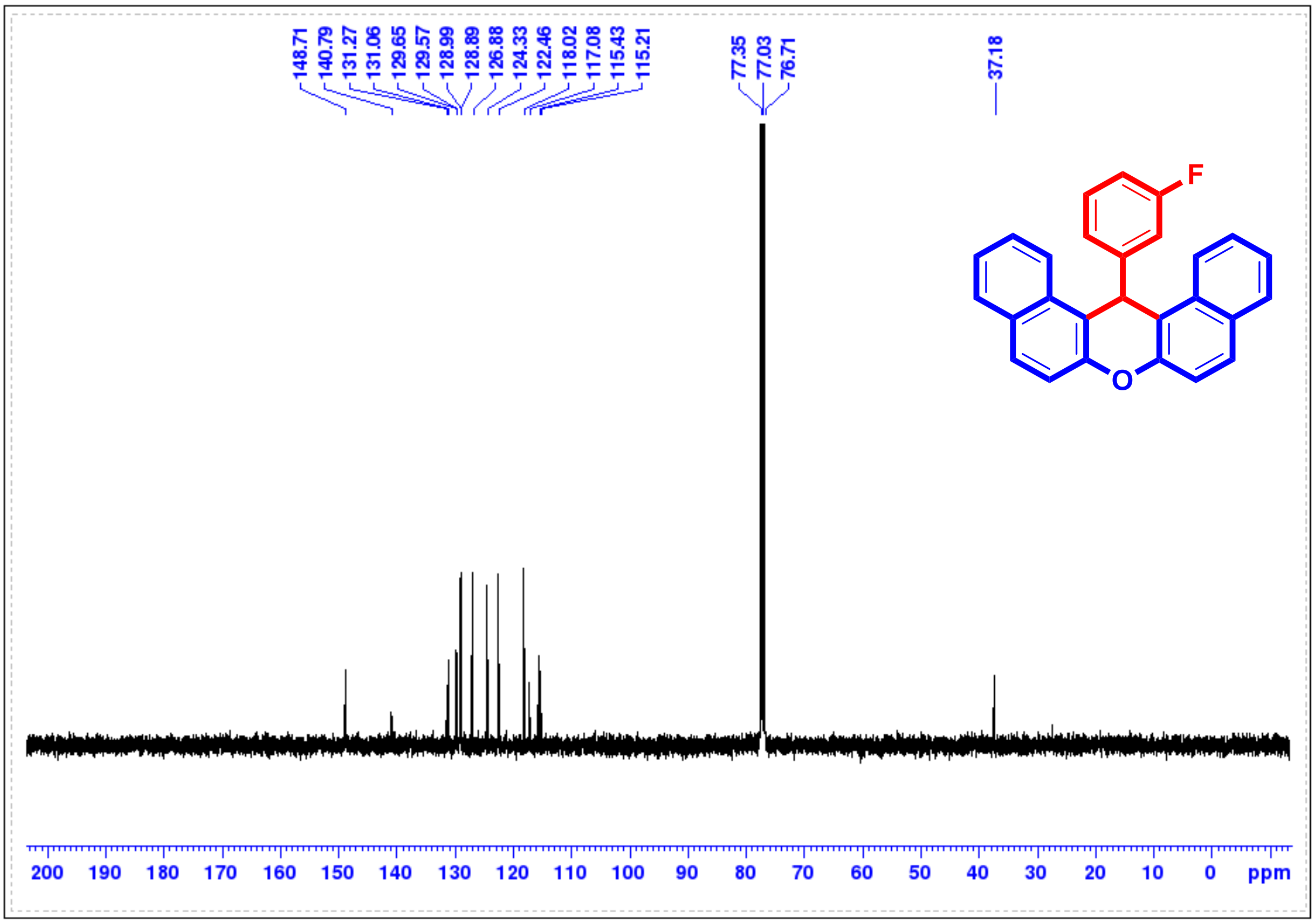

Fig. S36: ^{13}C NMR spectrum (100 MHz) of compound 6k in $CDCl_3$

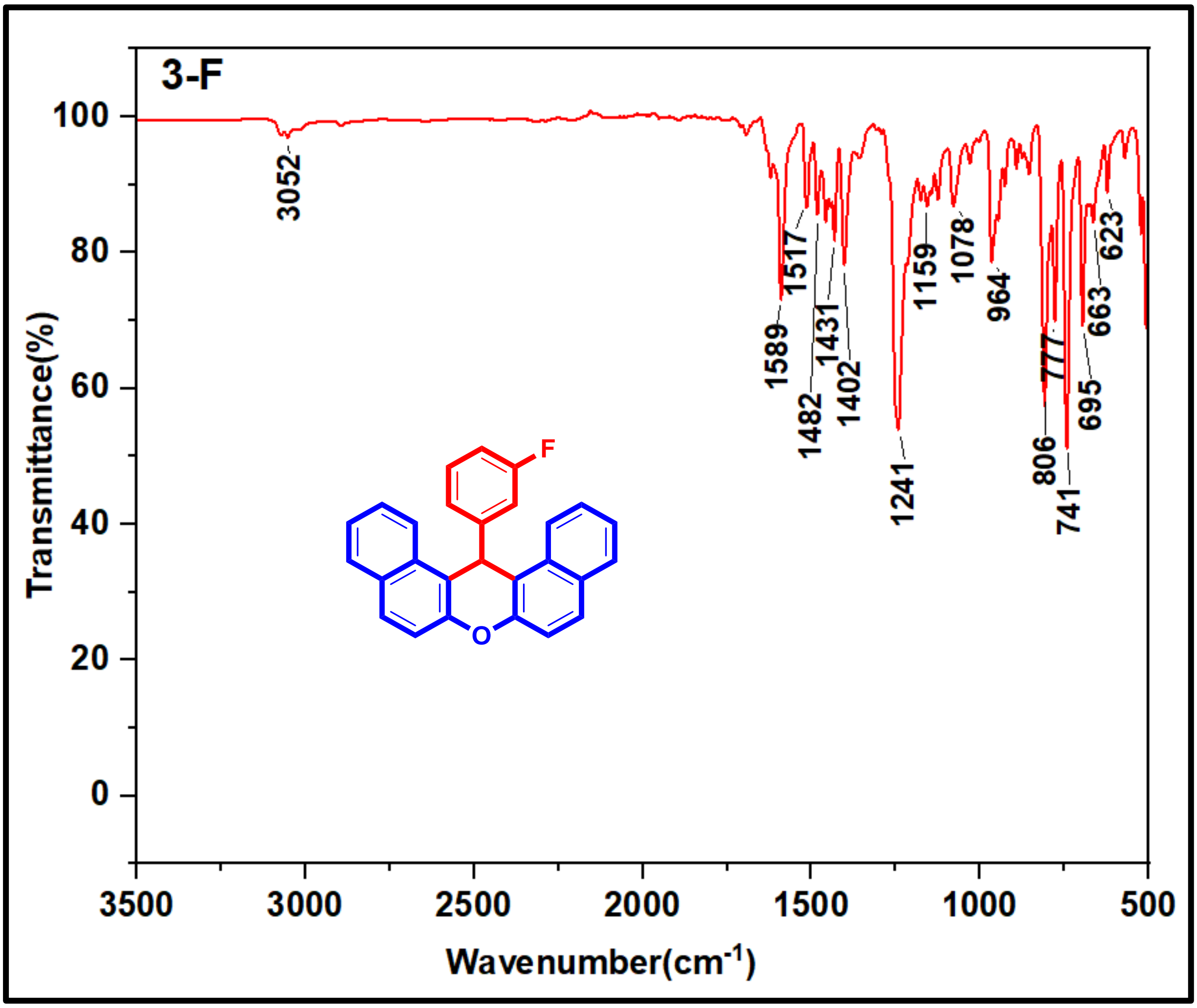

Fig. S37. FT-IR spectrum of compound 6k

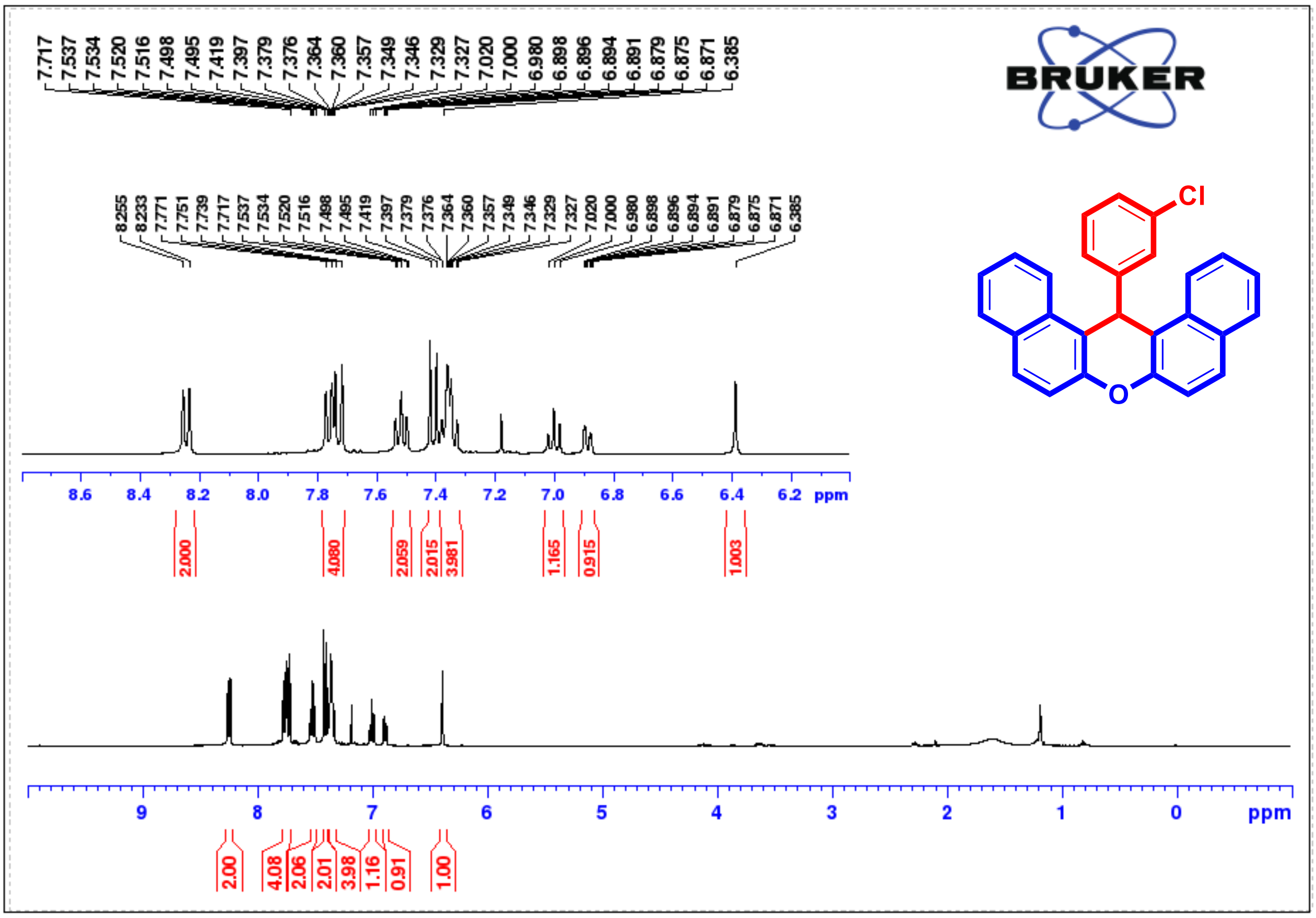

Fig. S38: ^{1}H NMR spectrum (400 MHz) of compound 6l in $CDCl_3$

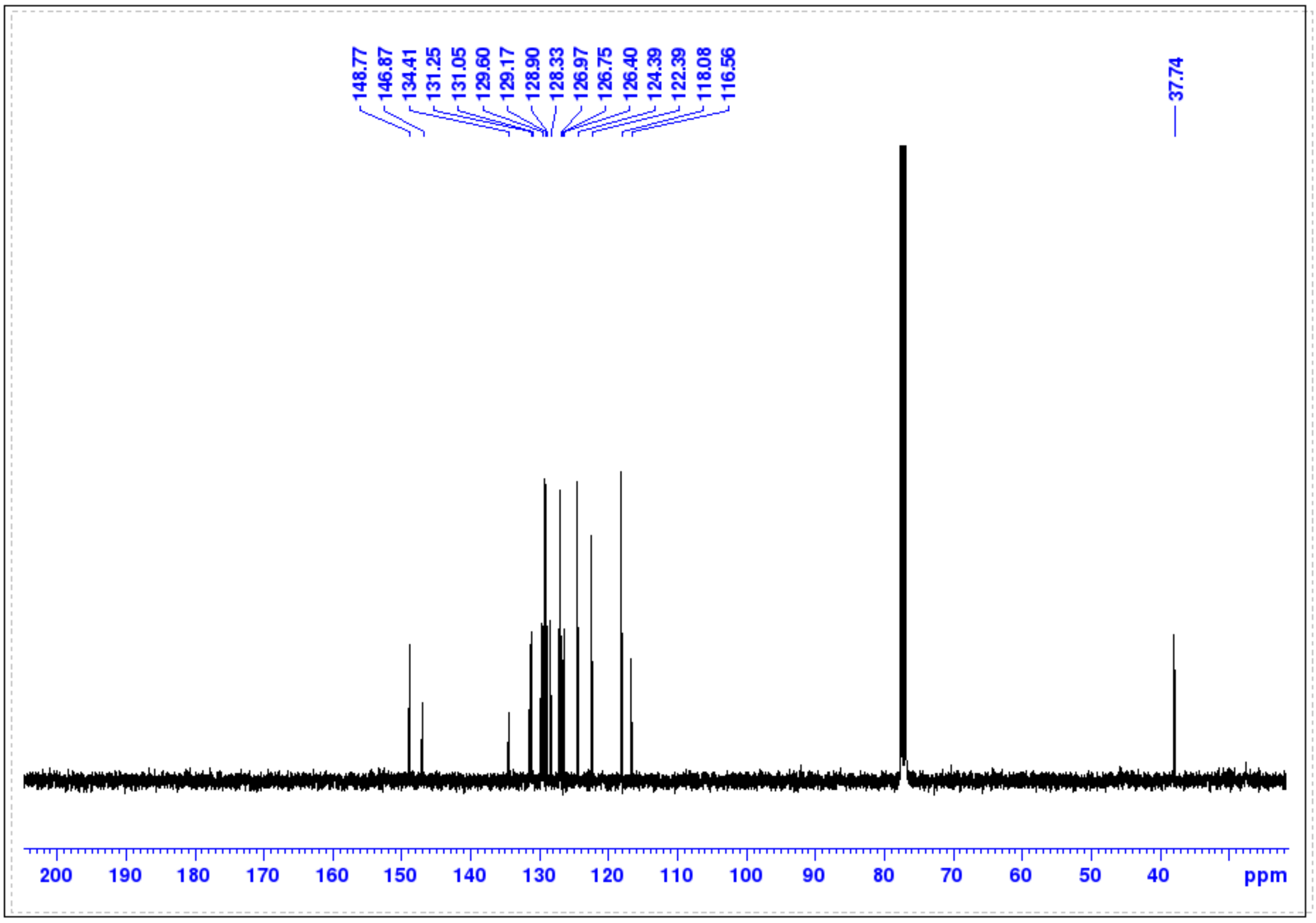

Fig. S39: ^{13}C NMR spectrum (100 MHz) of compound 6l in $CDCl_3$

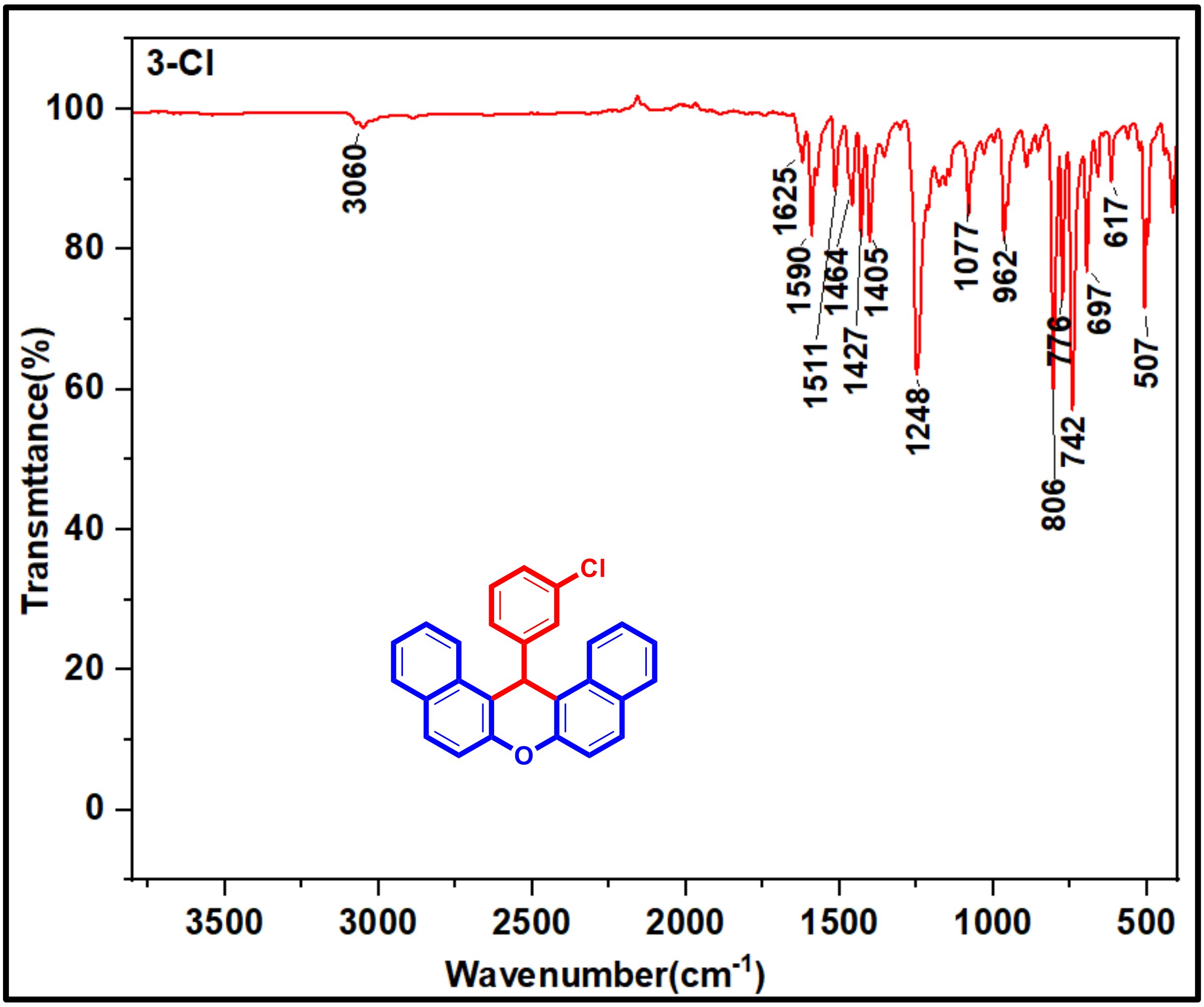

Fig. S40. FT-IR spectrum of compound 6l

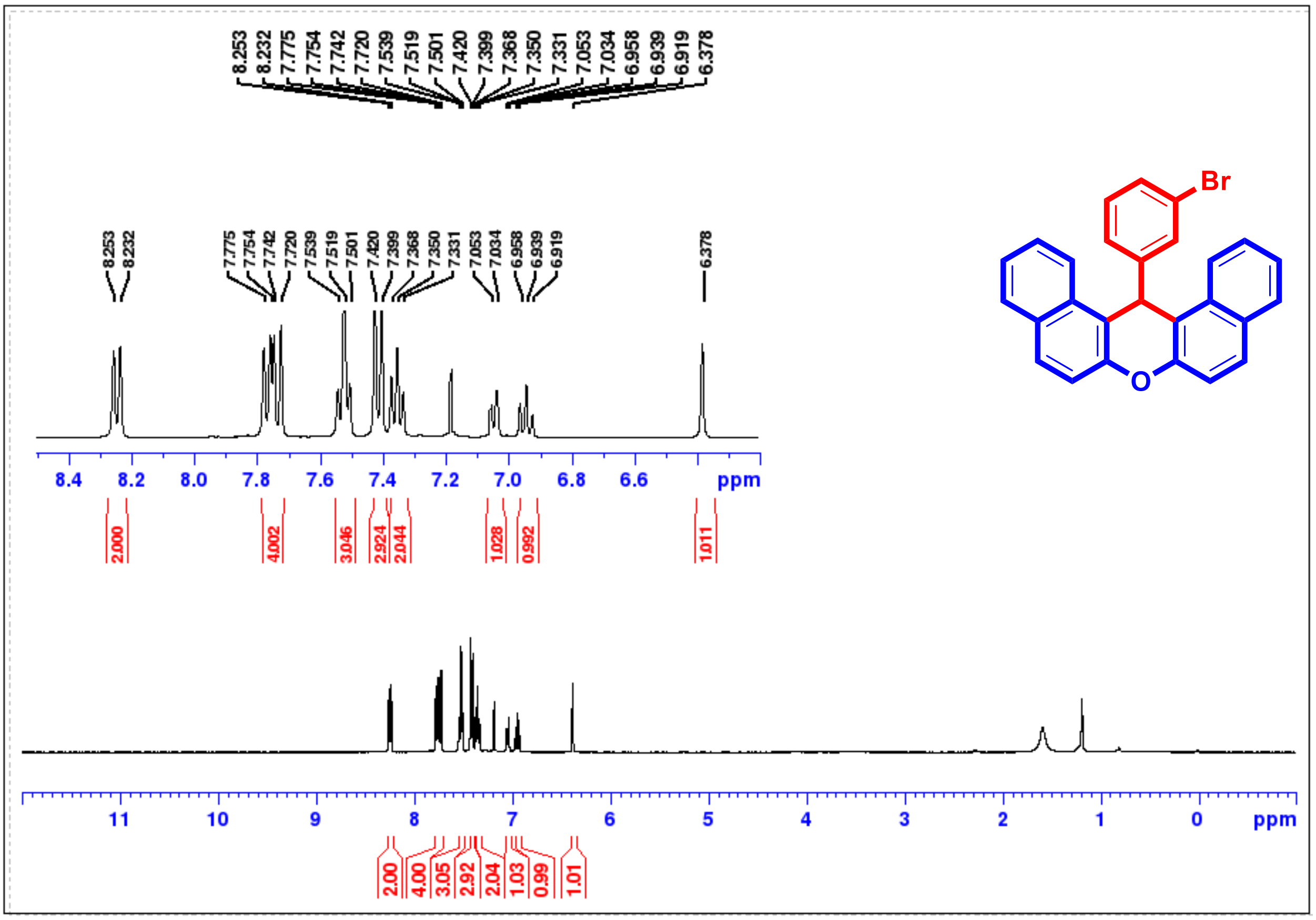

Fig. S41: ^{1}H NMR spectrum (400 MHz) of compound 6m in $CDCl_3$

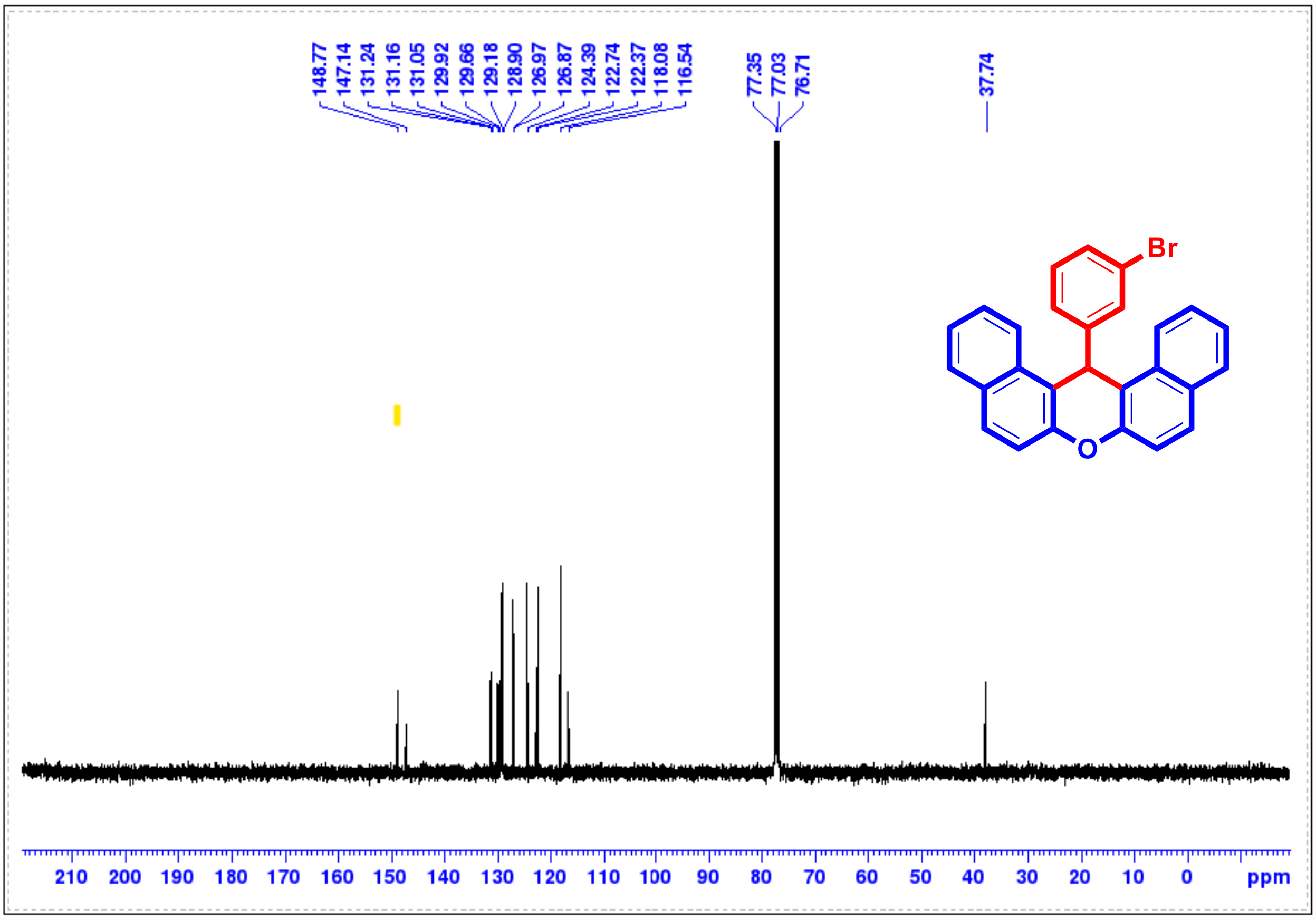

Fig. S42: ^{13}C NMR spectrum (100 MHz) of compound 6m in $CDCl_3$

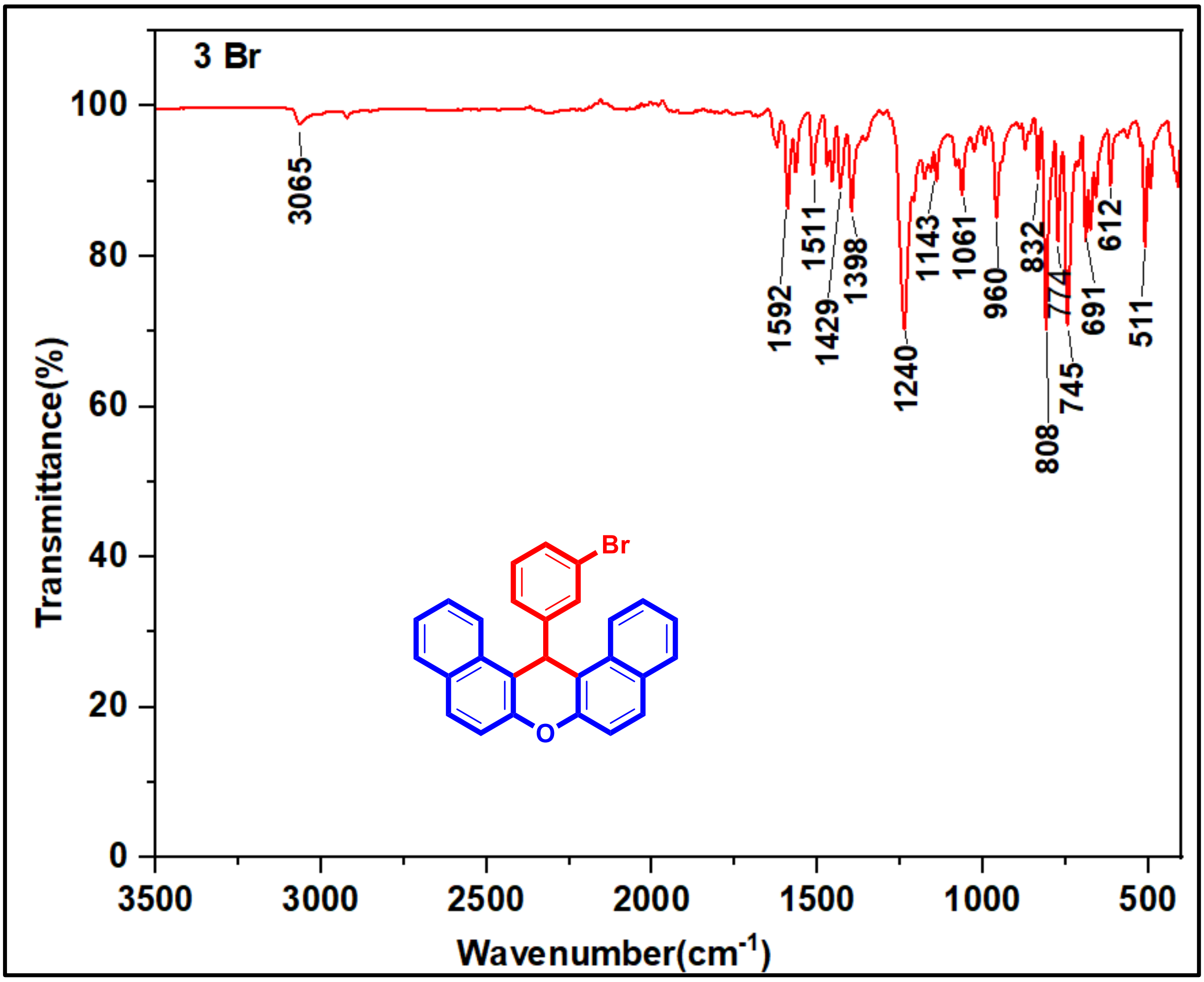

Fig. S43. FT-IR spectrum of compound 6m

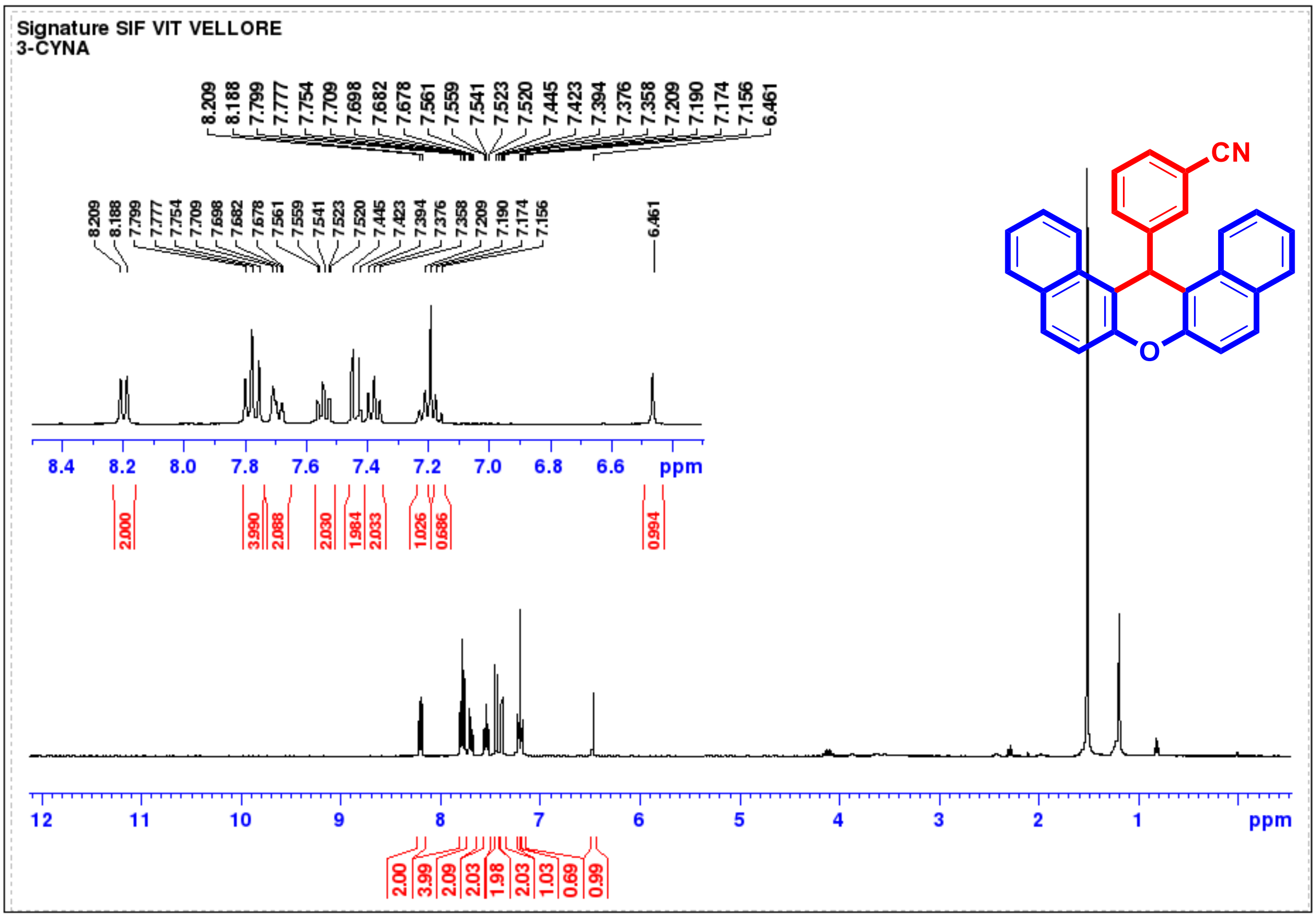

Fig. S44: ^{1}H NMR spectrum (400 MHz) of compound 6n in $CDCl_3$

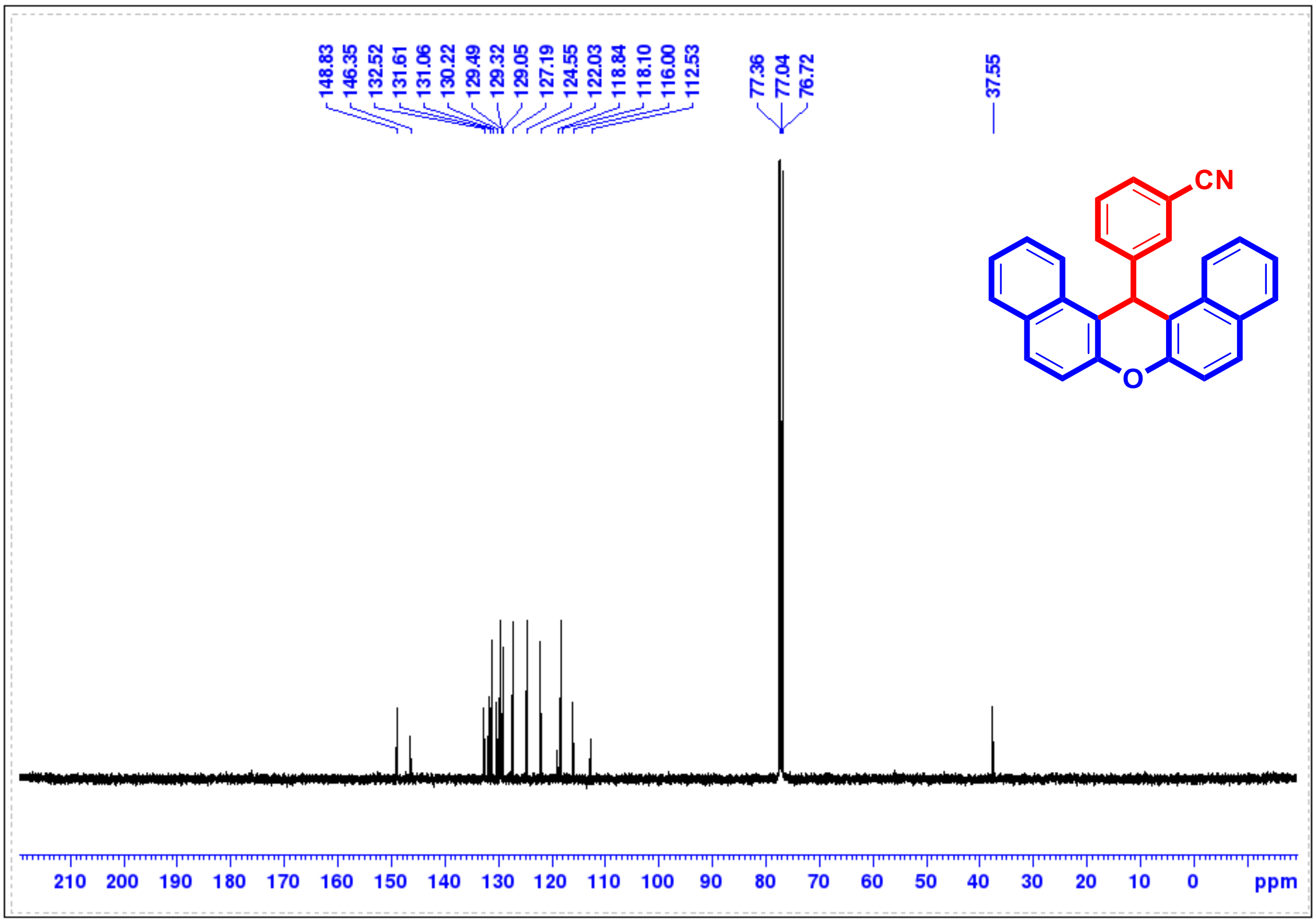

Fig. S45: ^{13}C NMR spectrum (100 MHz) of compound 6n in $CDCl_3$

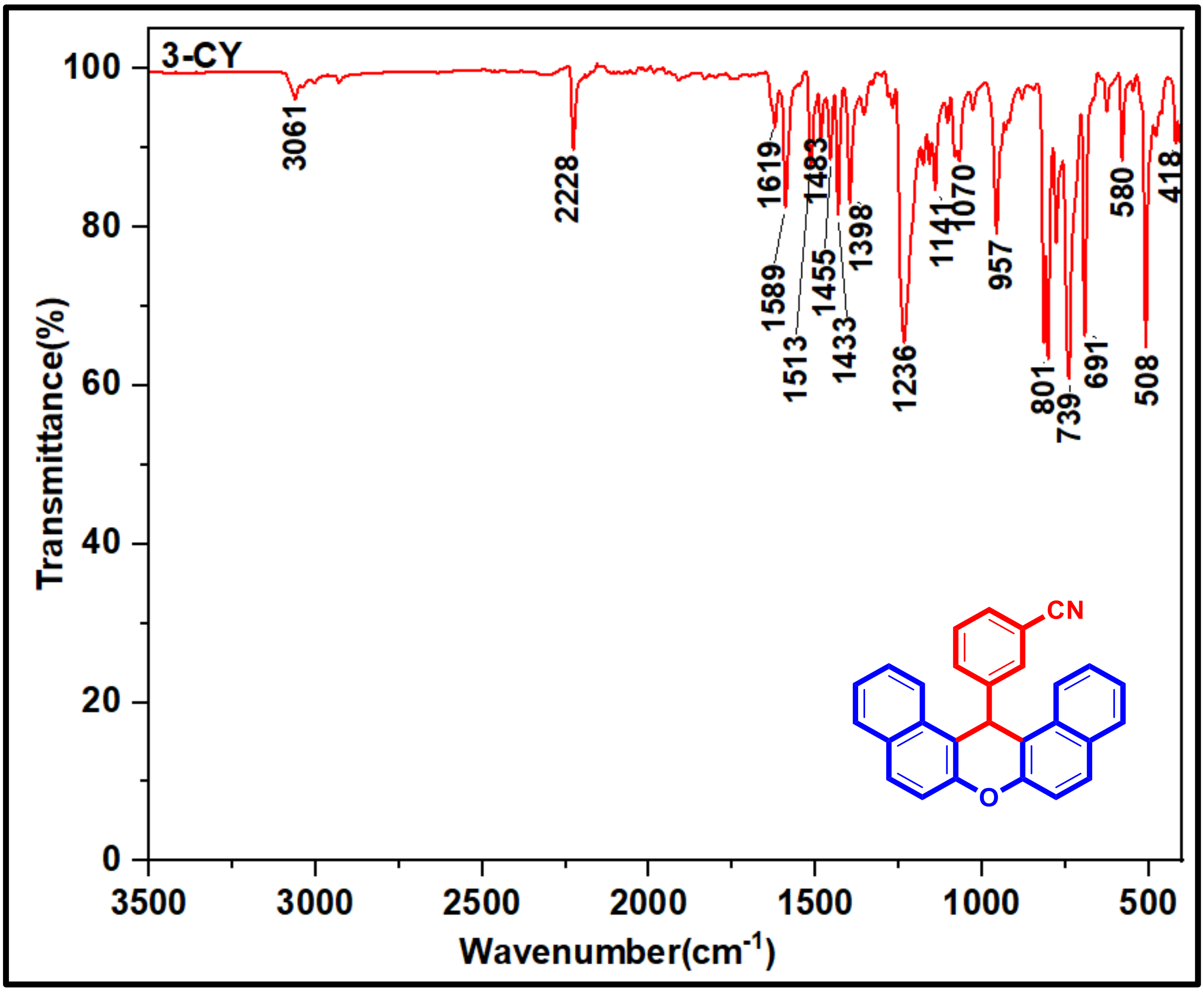

Fig. S46. FT-IR spectrum of compound 6n

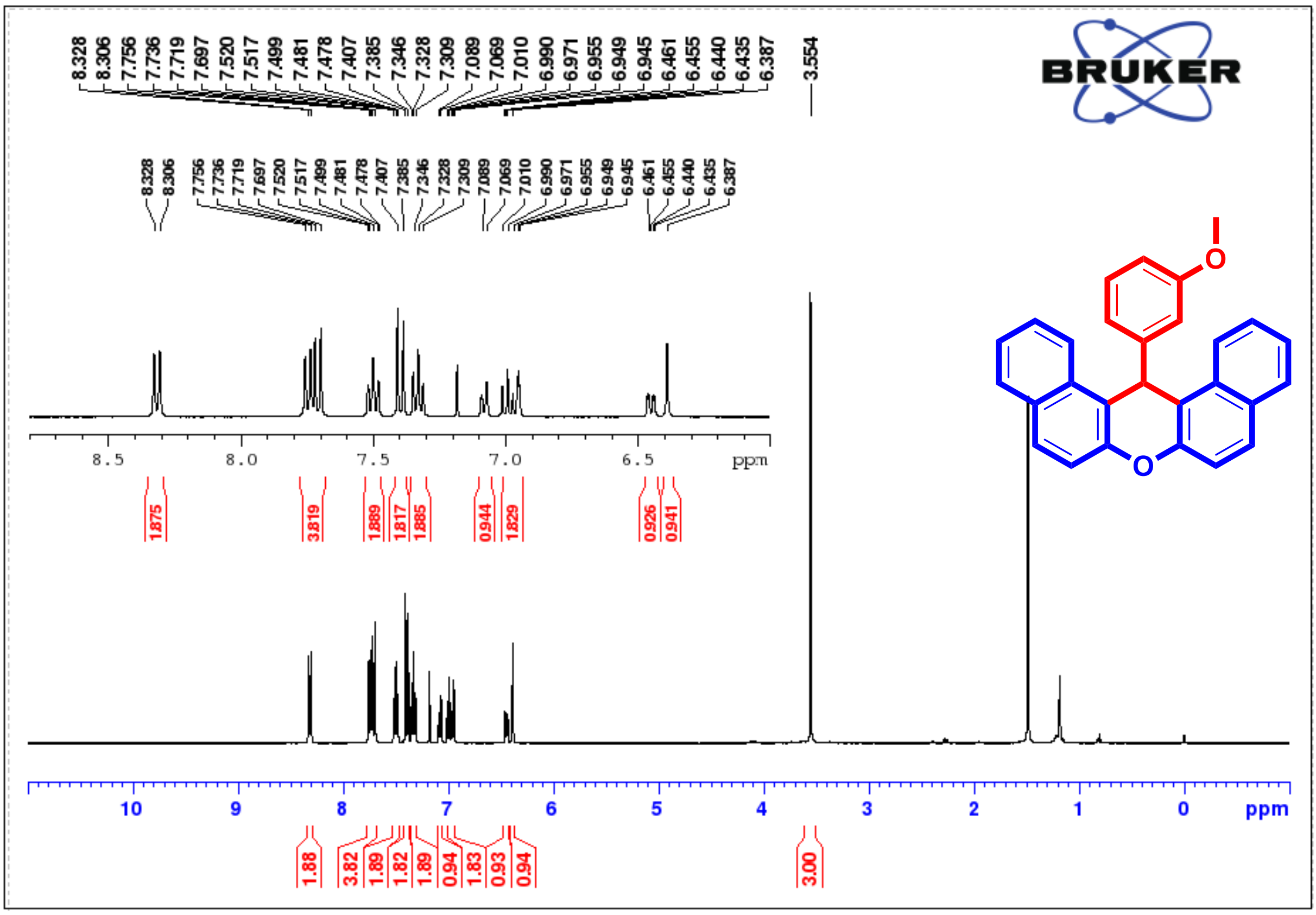

Fig. S47: ^{1}H NMR spectrum (400 MHz) of compound 6o in $CDCl_3$

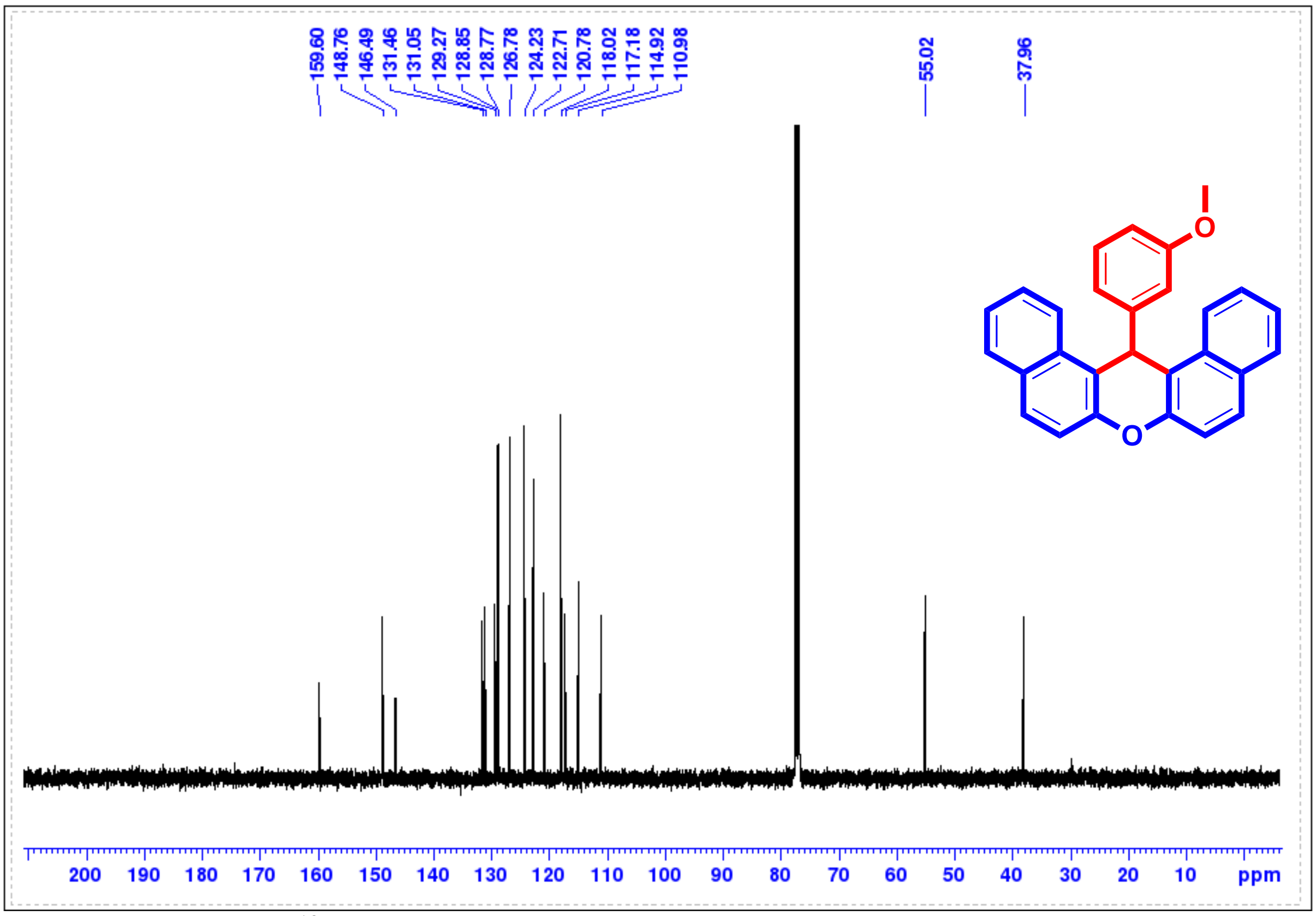

Fig. S48: ^{13}C NMR spectrum (100 MHz) of compound 6o in $CDCl_3$

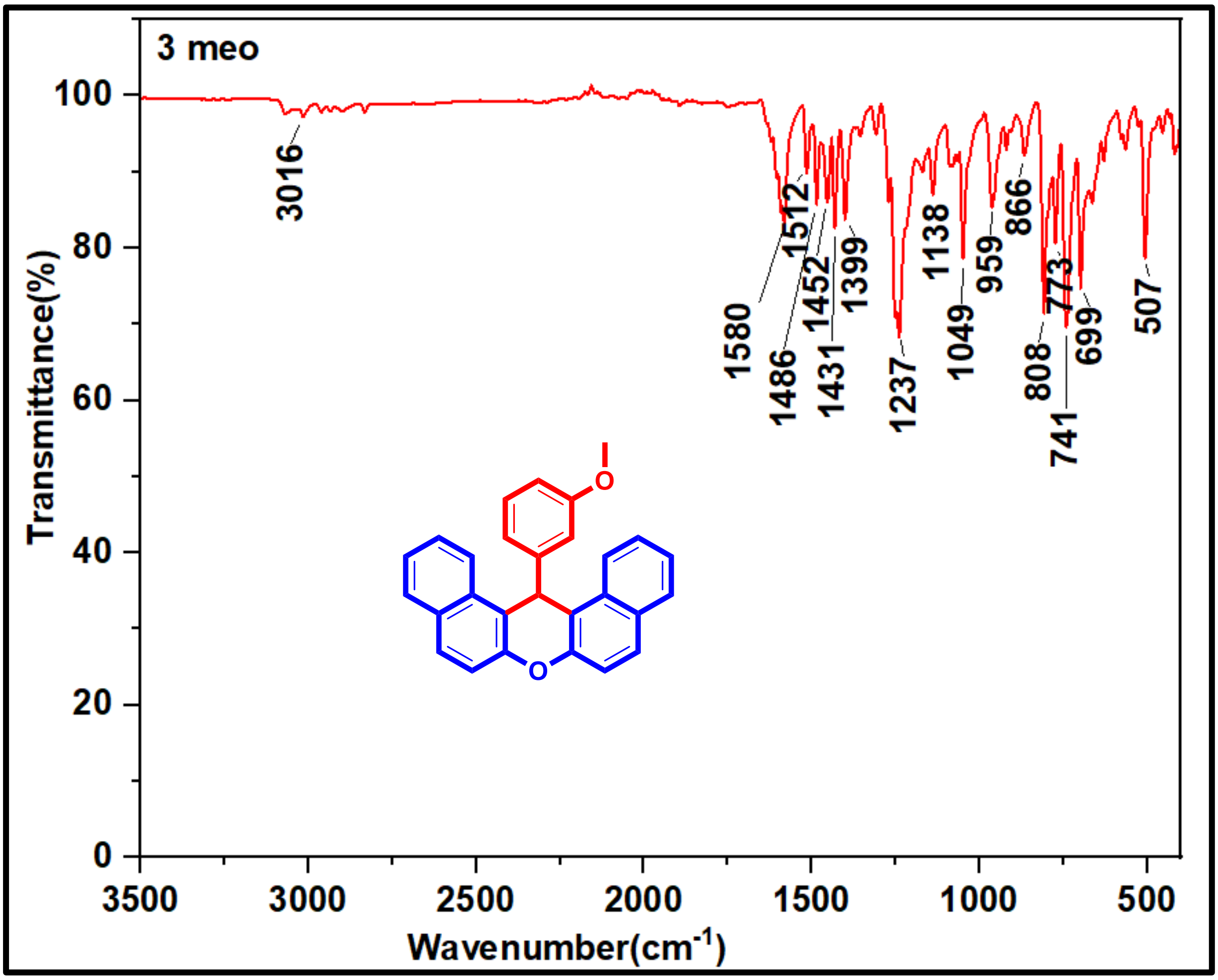

Fig. S49. FT-IR spectrum of compound 6o

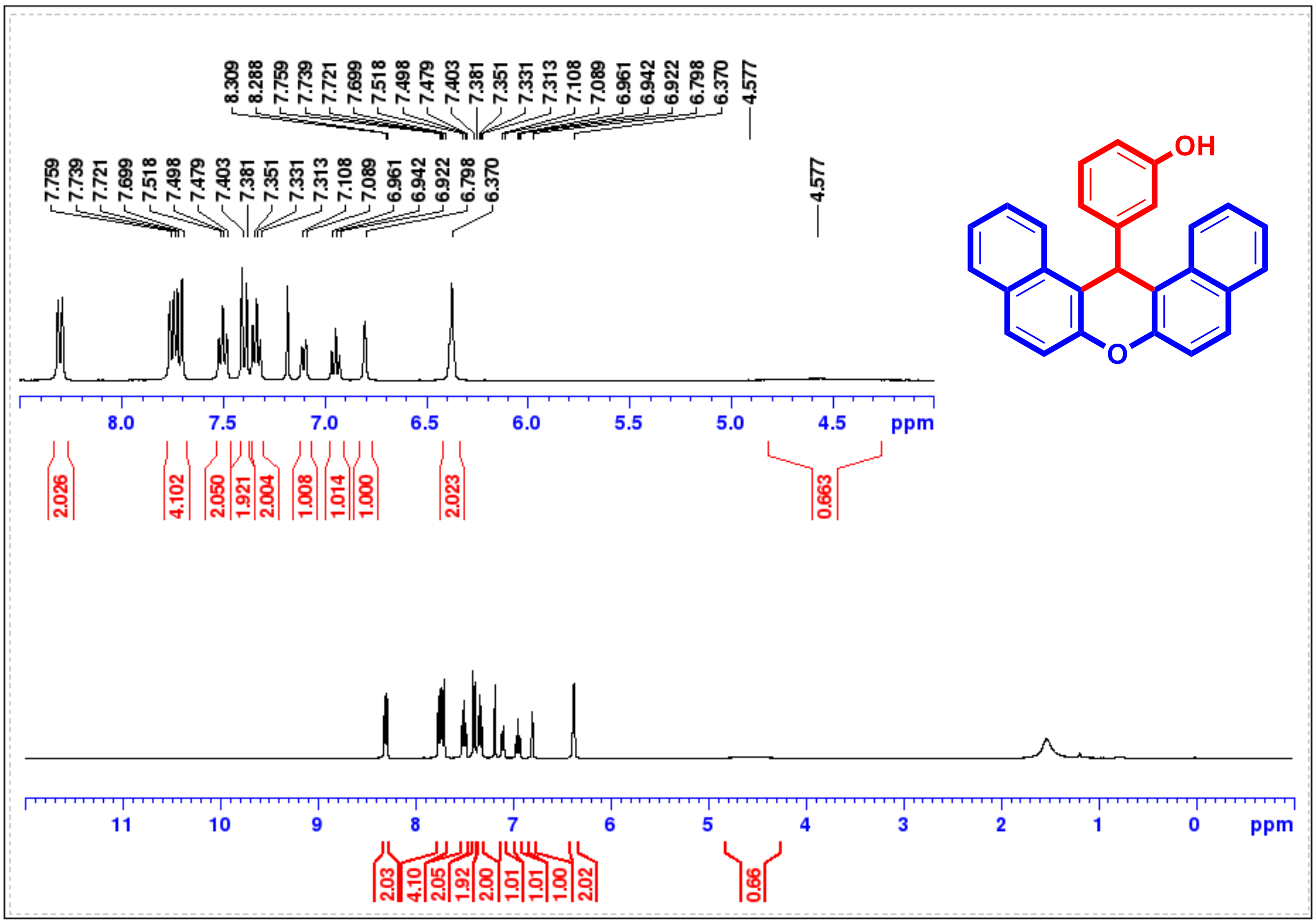

Fig. S50: ^{1}H NMR spectrum (400 MHz) of compound 6p in $CDCl_3$

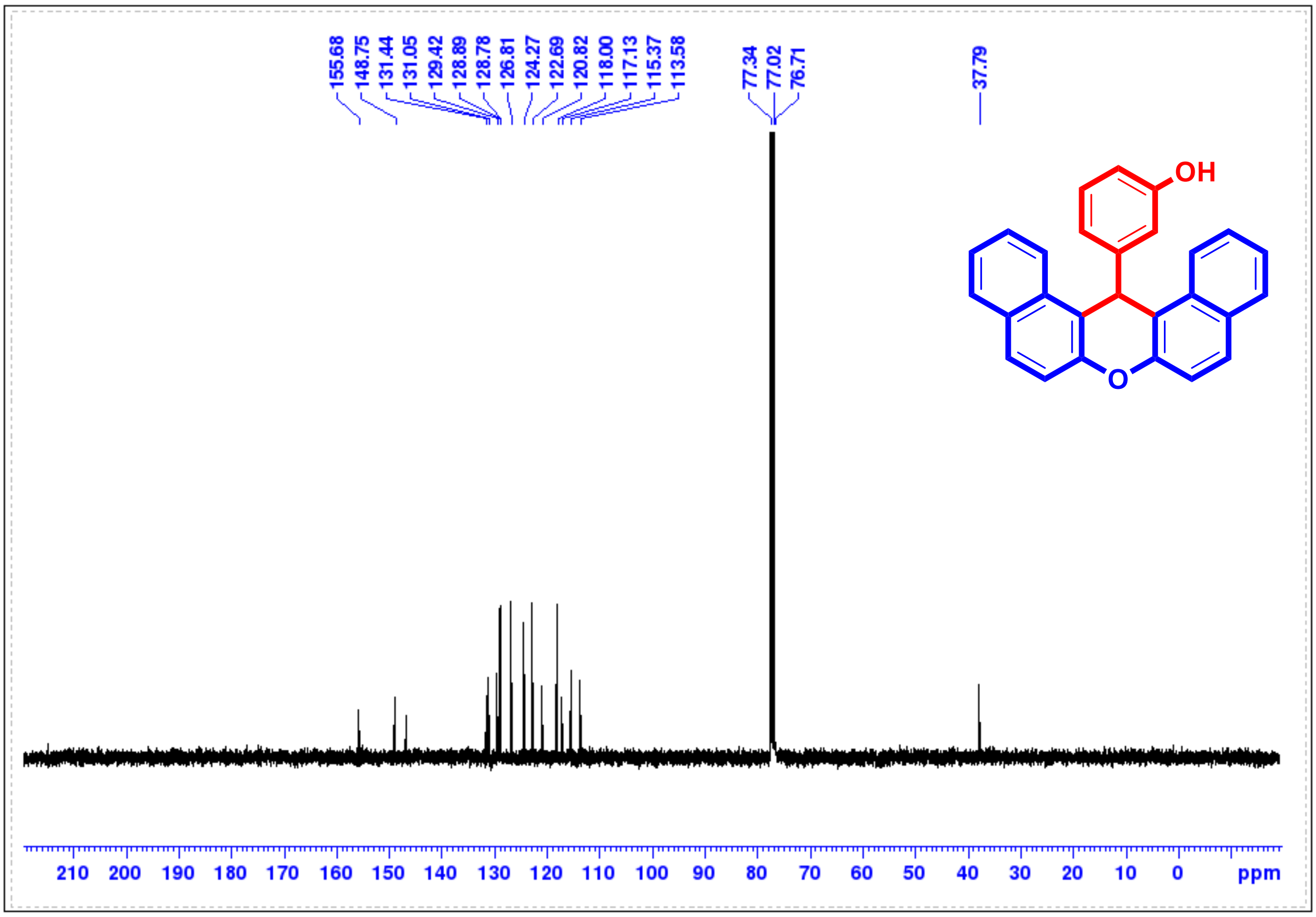

Fig. S51: ^{13}C NMR spectrum (100 MHz) of compound 6p in $CDCl_3$

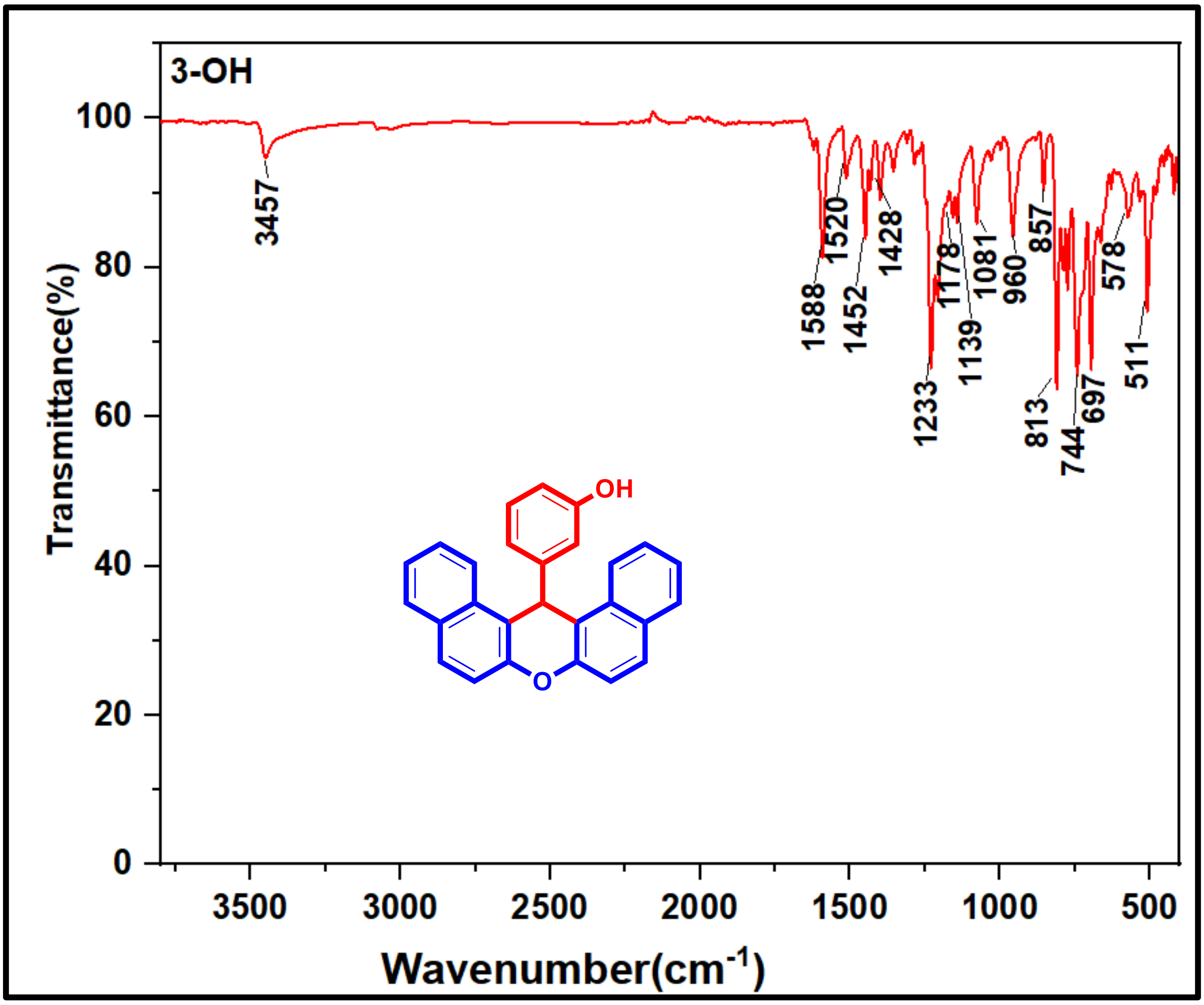

Fig. S52. FT-IR spectrum of compound 6p

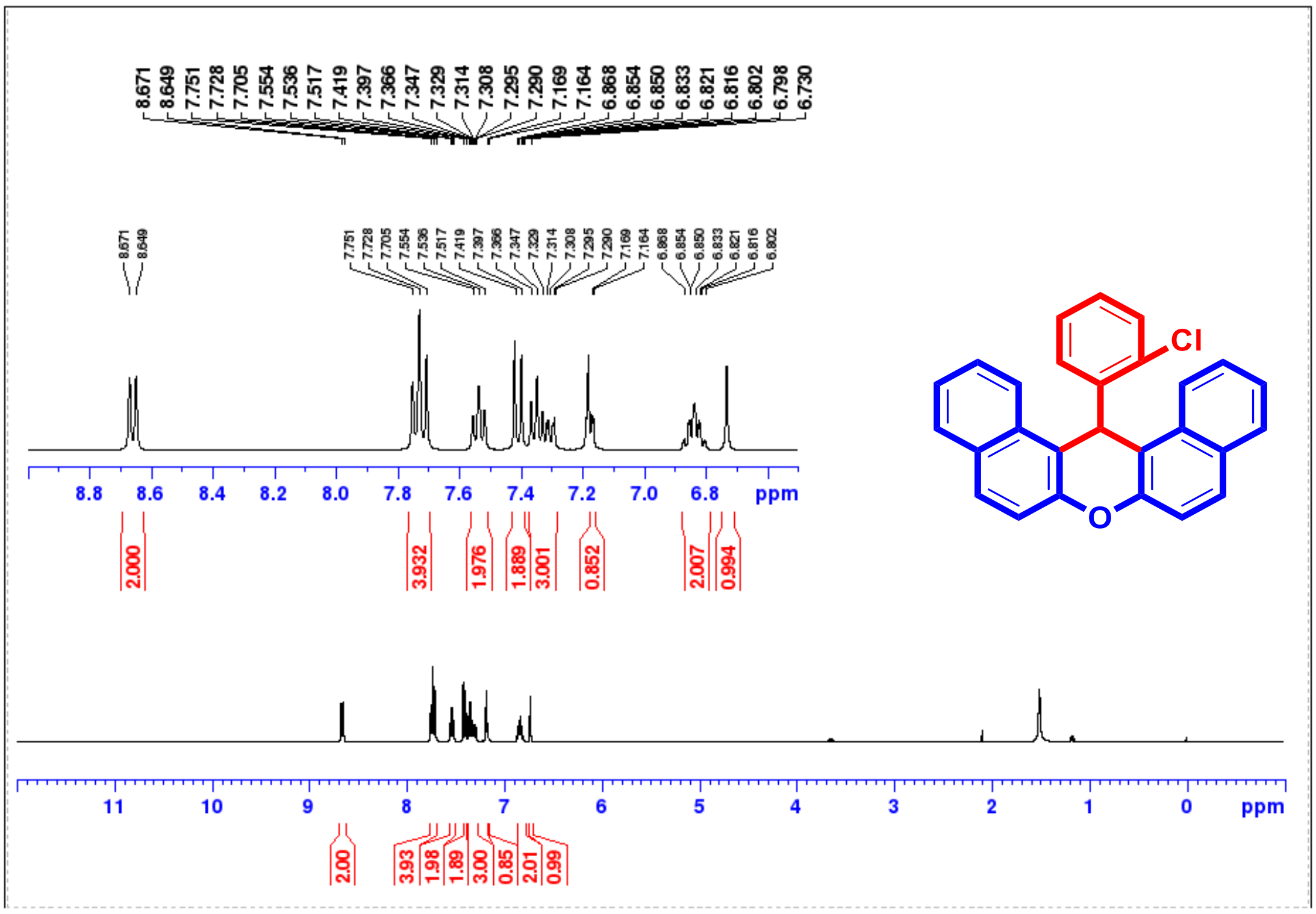

Fig. S53: ^{1}H NMR spectrum (400 MHz) of compound 6q in $CDCl_3$

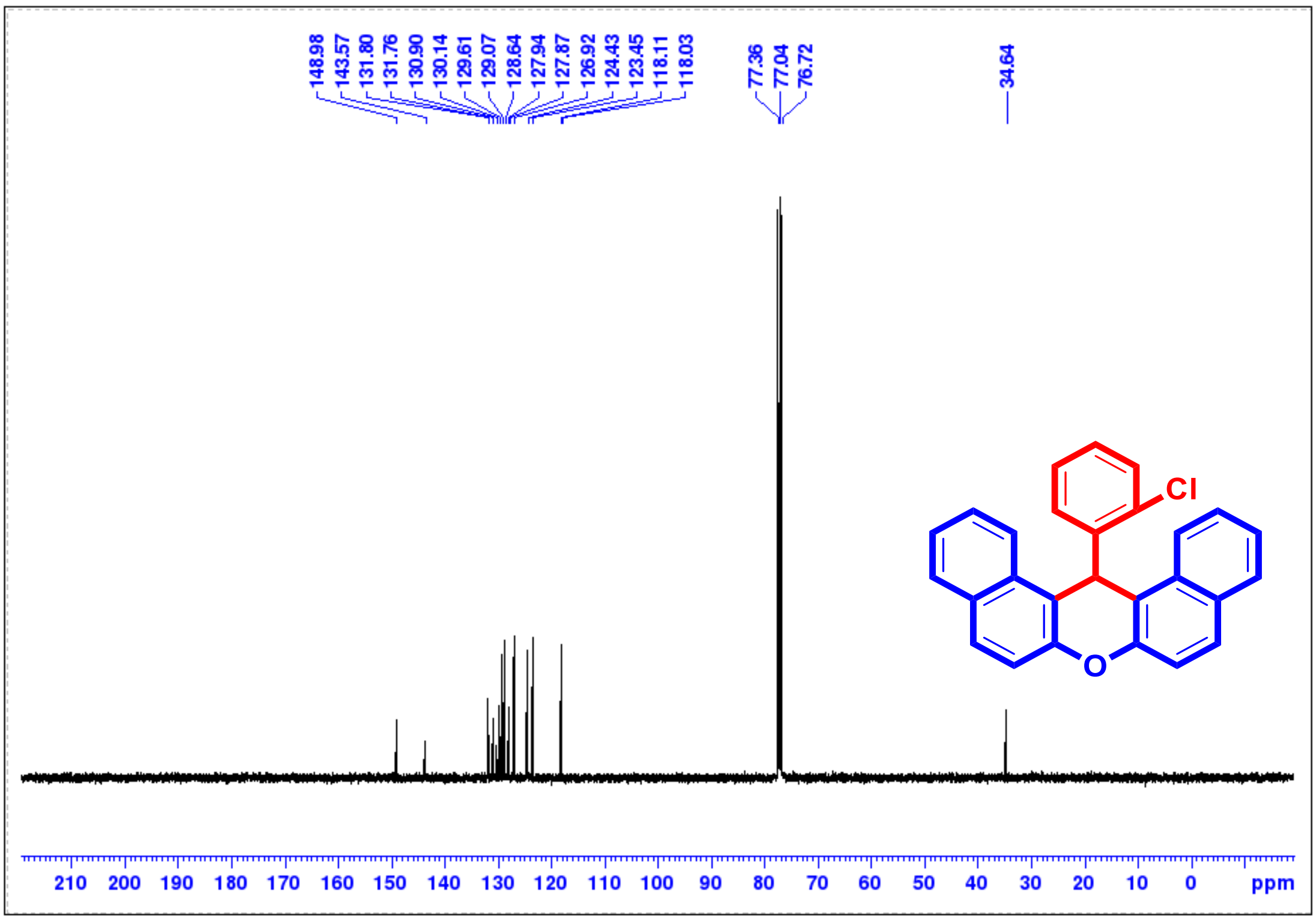

Fig. S54: ^{13}C NMR spectrum (100 MHz) of compound 6q in $CDCl_3$

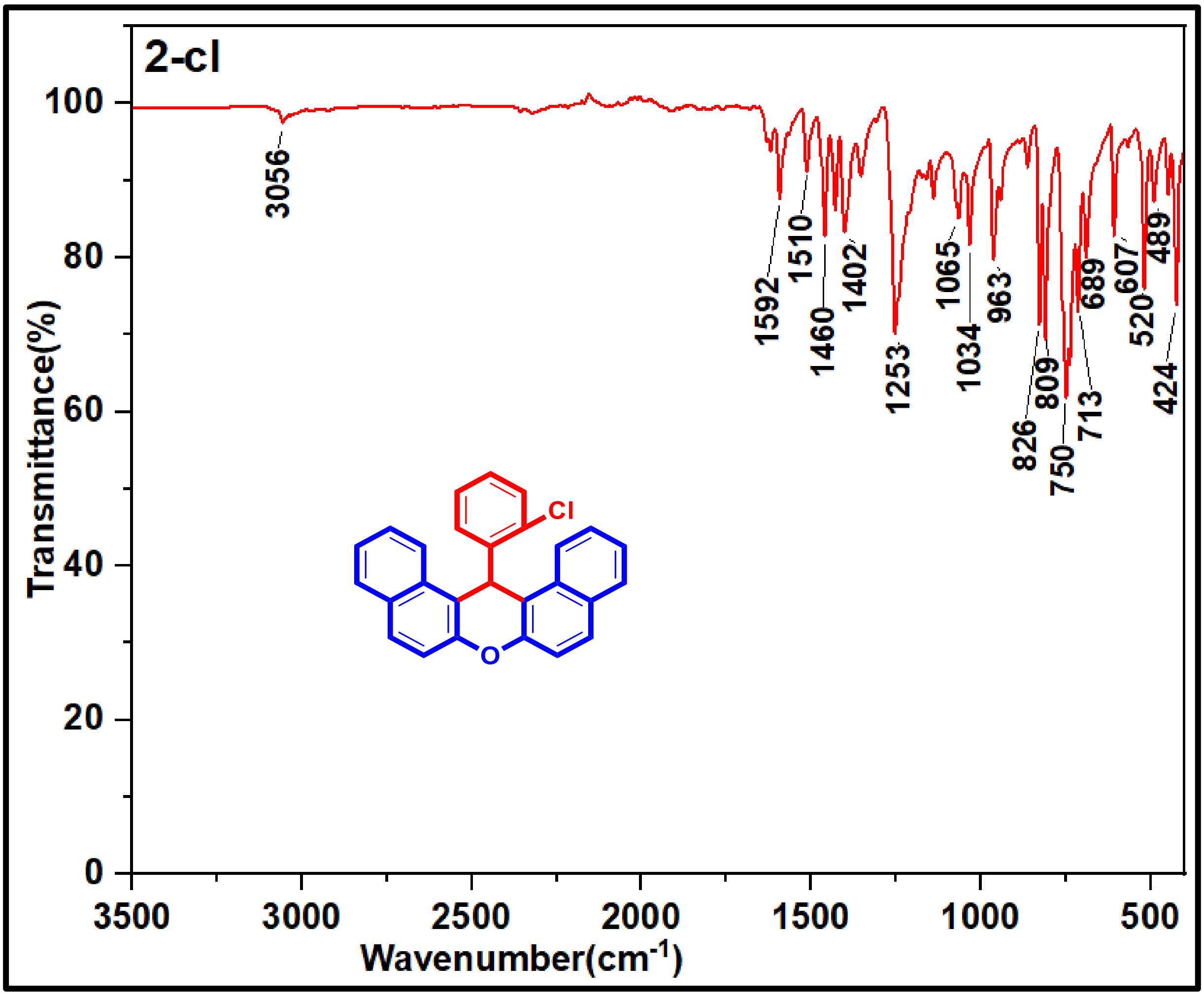

Fig. S55. FT-IR spectrum of compound 6q

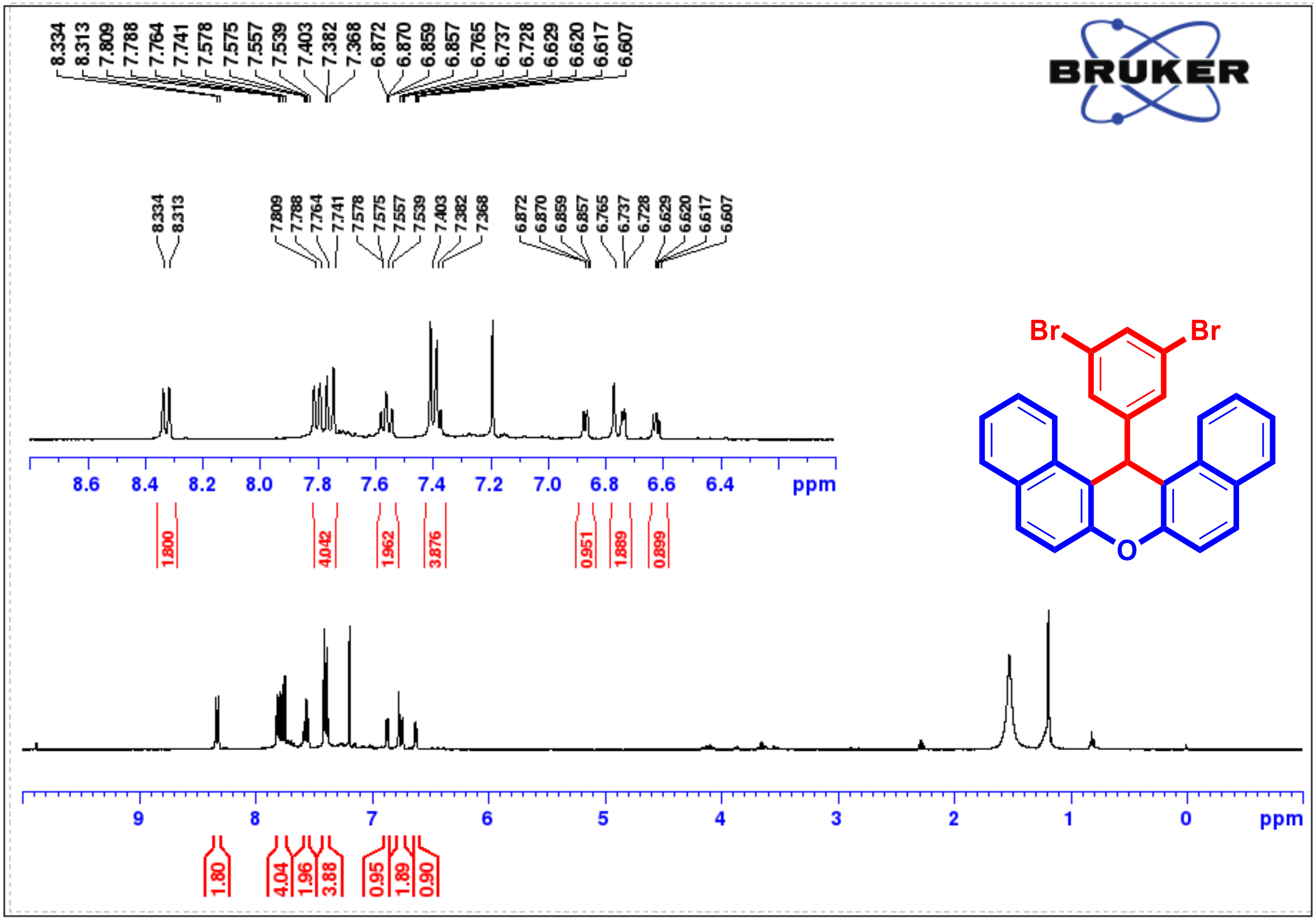

Fig. S56: ^{1}H NMR spectrum (400 MHz) of compound 6r in $CDCl_3$

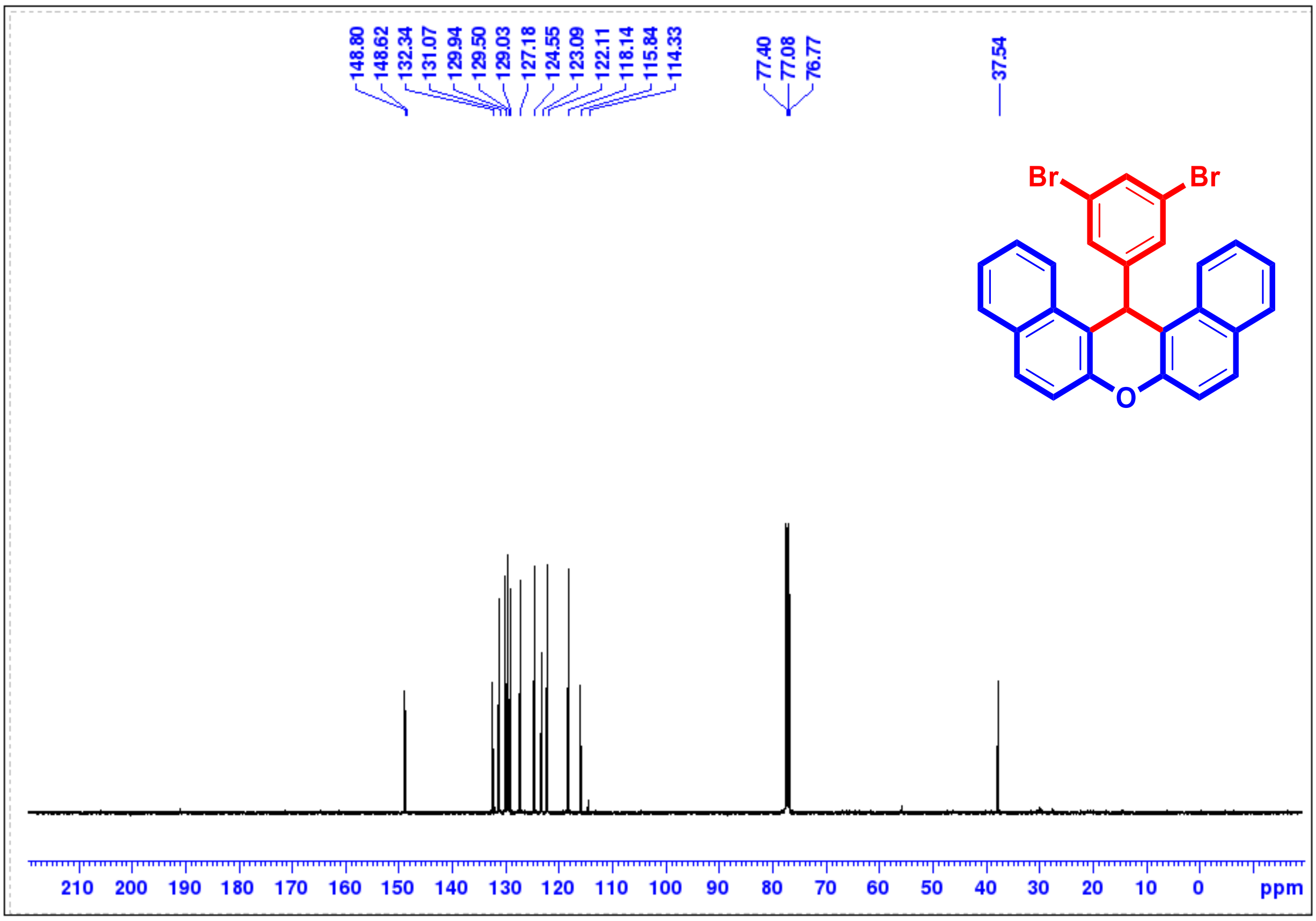

Fig. S57: ^{13}C NMR spectrum (100 MHz) of compound 6r in $CDCl_3$

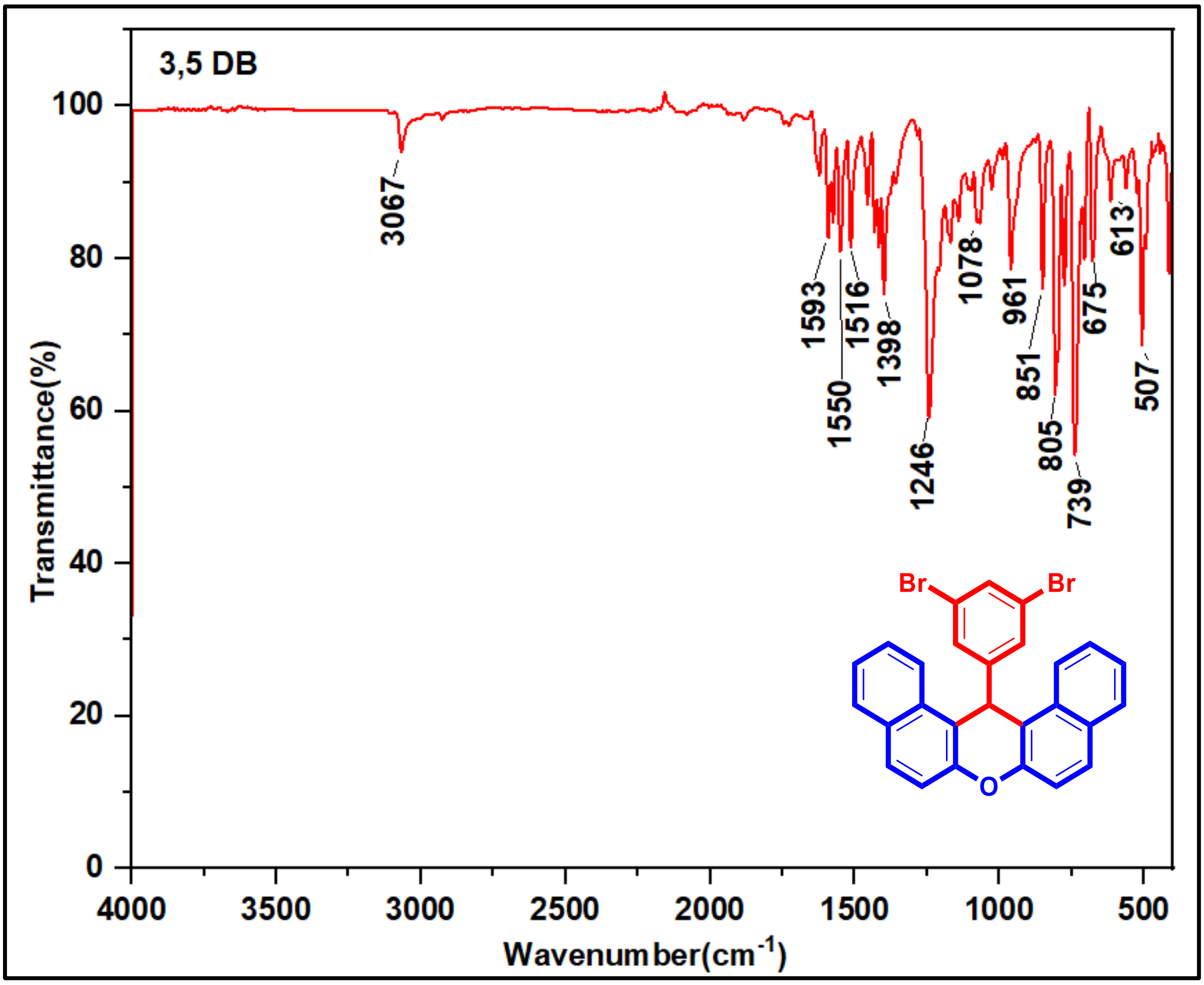

Fig. S58. FT-IR spectrum of compound 6r

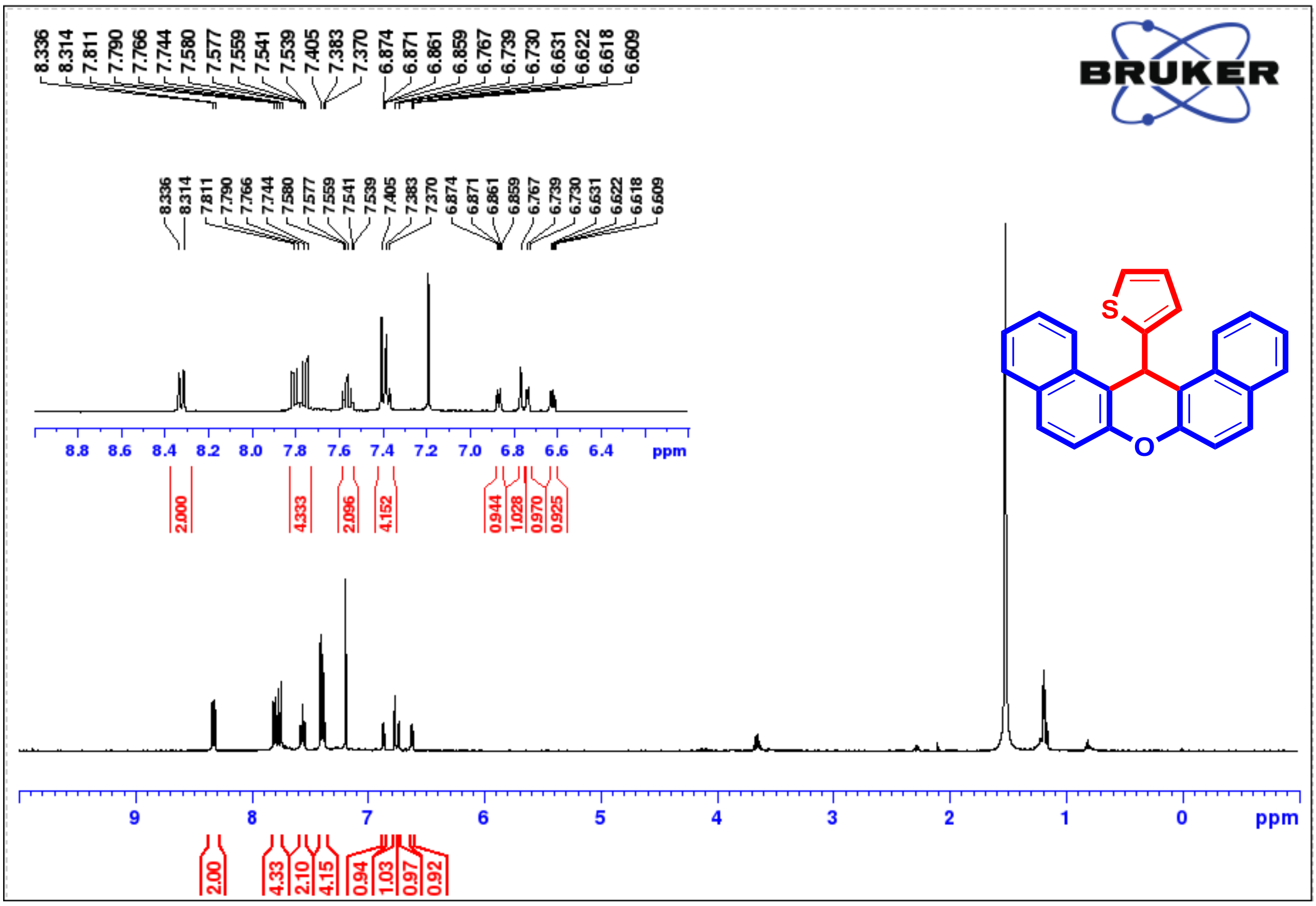

Fig. S59: ^{1}H NMR spectrum (400 MHz) of compound 6s in $CDCl_3$

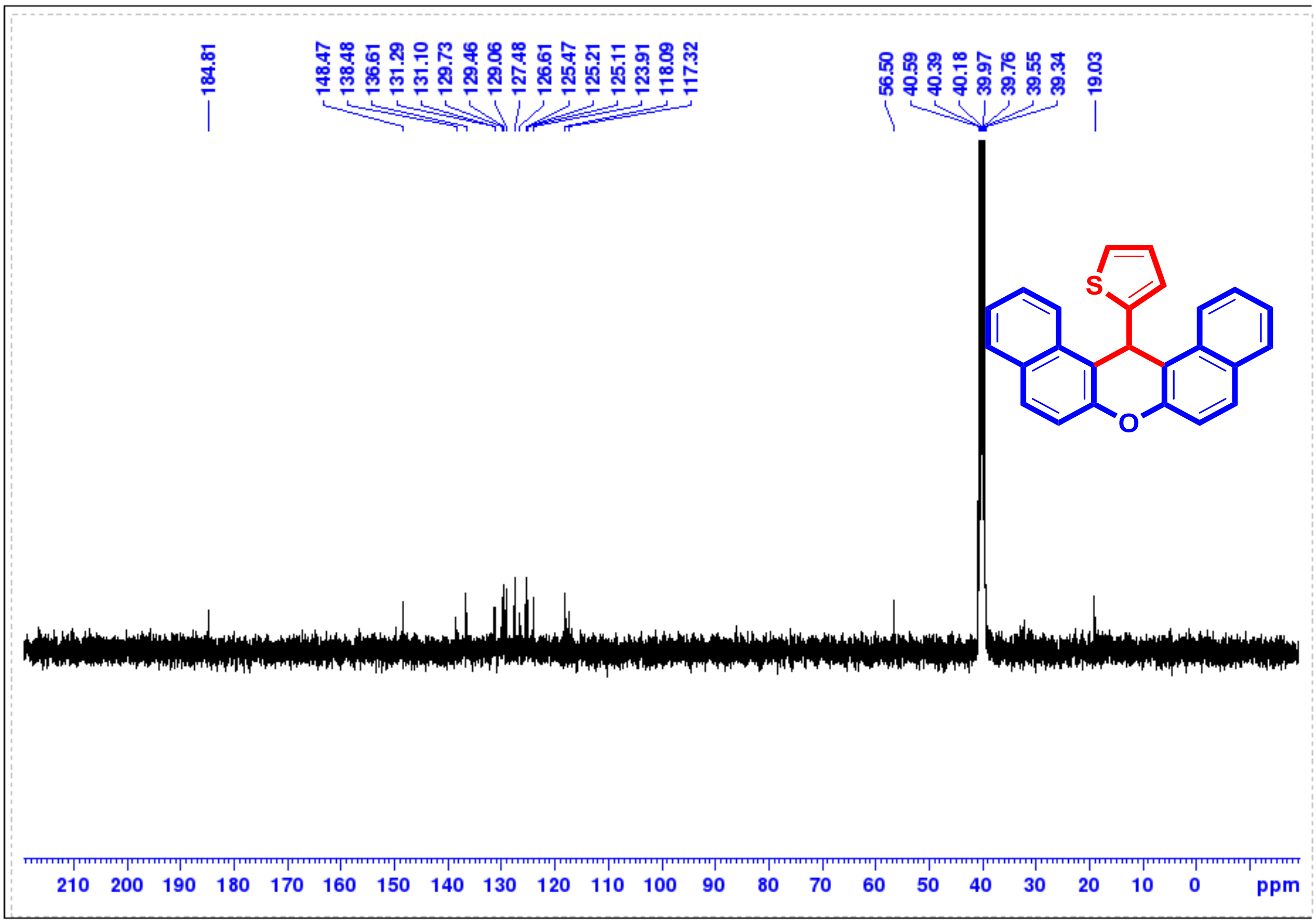

Fig. S60: ^{13}C NMR spectrum (100 MHz) of compound 6s in DMSO-d_6

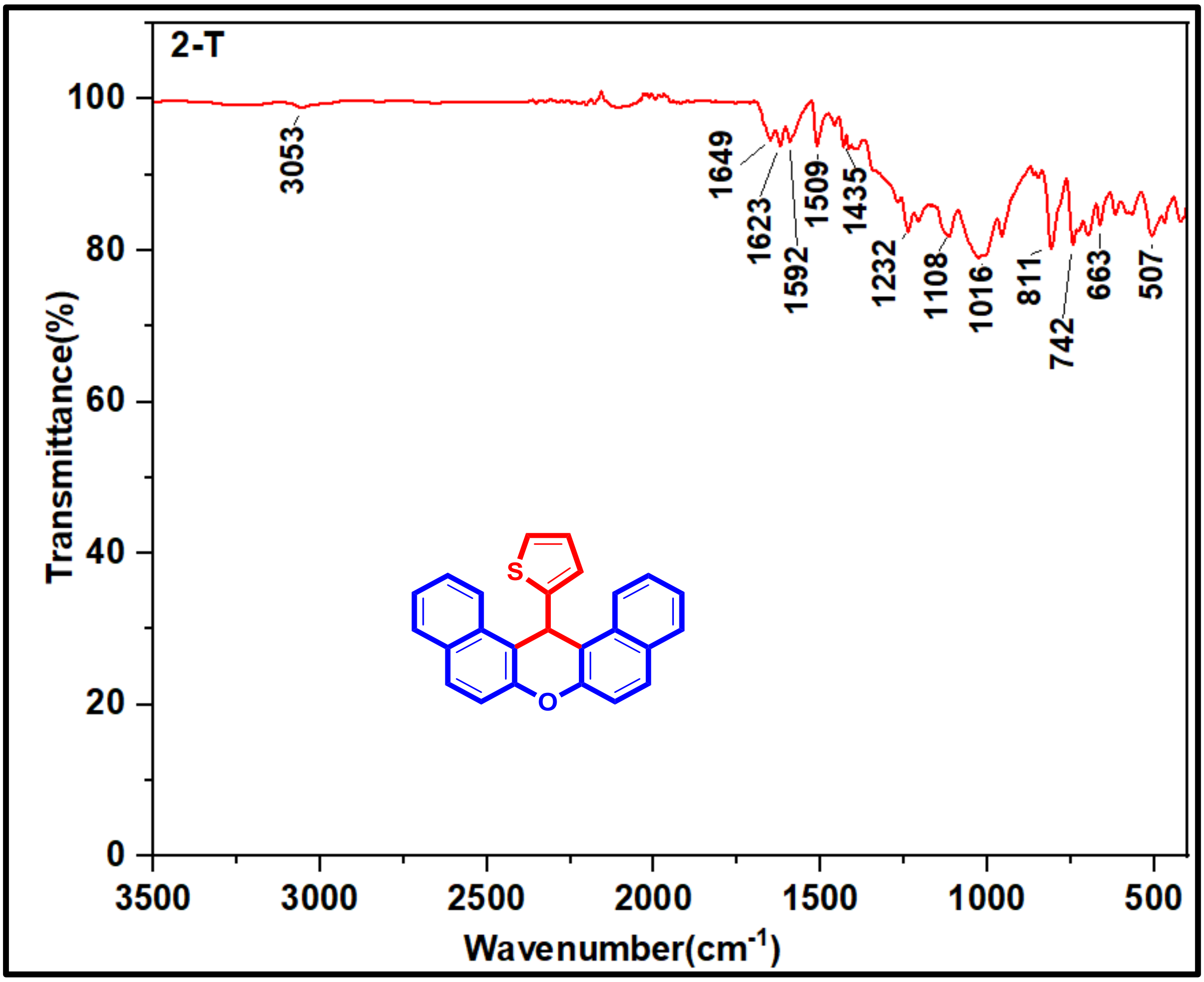

Fig. S61. FT-IR spectrum of compound 6s

Table S1. Calculating Drug-Likeness using swissADME data for compound (6a-6s)

ADME and drug-likeness predictions were performed using the SwissADME web tool (http://www.swissadme.ch). The chemical structures of compounds (6a–6s) were drawn in ChemDraw, and the corresponding SMILES notations were generated using the Copy as SMILES option. These SMILES strings were used as input for SwissADME. The calculated parameters included physicochemical properties (molecular weight, HBA, HBD, TPSA, log P), pharmacokinetics (GI absorption, BBB permeation, P-gp substrate prediction), and CYP450 enzyme inhibition profiles. All values reported in the manuscript and Supporting Information were taken directly from the SwissADME output tables.[5]

Drug	HB Acceptors	HB Donors	TPSA	CYP1A2 inhibitor	CYP2C19 inhibitor	CYP2C9 inhibitor	CYP2D6 inhibitor	CYP3A4 inhibitor	PAIN alerts
**6a**	1	0	9.23	NO	YES	NO	NO	NO	0
**6b**	2	0	9.23	NO	YES	NO	NO	NO	0
**6c**	1	0	9.23	NO	YES	NO	NO	NO	0
**6d**	1	0	9.23	NO	YES	NO	NO	NO	0
**6e**	2	0	33.02	YES	YES	NO	NO	NO	0
**6f**	3	0	55.05	YES	YES	NO	NO	NO	0
**6g**	2	0	18.46	NO	YES	NO	NO	NO	0
**6h**	1	0	9.23	NO	YES	NO	NO	NO	0
**6i**	2	0	18.46	YES	YES	NO	NO	NO	0
**6j**	1	0	9.23	NO	YES	NO	NO	NO	0
**6k**	2	0	9.23	NO	YES	NO	NO	NO	0
**6l**	1	0	9.23	NO	YES	NO	NO	NO	0
**6m**	1	0	9.23	NO	YES	NO	NO	NO	0
**6n**	2	0	33.02	YES	YES	NO	NO	NO	0
**6o**	2	0	18.46	YES	YES	NO	NO	NO	0
**6p**	2	1	29.46	YES	YES	NO	NO	NO	0
**6q**	1	0	9.23	NO	YES	NO	NO	NO	0
**6r**	1	0	9.23	NO	NO	NO	NO	NO	0
**6s**	1	0	37.47	YES	YES	NO	NO	NO	0

*HB=Hydrogen bond, TPSA=Topological polar surface area, PAINS=assay interference compound

Table S2. Assessment of (absorption, distribution, metabolism, excretion and toxicity (AdMET) properties for compound 6a-6s.

Absorption				Distribution		Metabolism	Excretion	Toxicity	
**Drug**	**C2P**	**HIA (%)**	**P-gpI**	**BBB**	**CNS**	**CYP3A4 Substrate**	**Total clearance**	**hERG Inhibitor**	**Toxicity (LD_{50})**
**6a**	0.949	100%	NO	1.219	-1.024	YES	0.213	NO	3.39
**6b**	0.948	100%	NO	1.228	-0.935	YES	0.058	NO	3.081
**6c**	0.947	100%	NO	1.166	-0.908	YES	0.116	NO	3.067
**6d**	0.887	100%	NO	1.197	-0.951	YES	0.138	NO	3.07
**6e**	0.934	100%	NO	-0.241	-1.043	YES	0.309	NO	3.571
**6f**	0.909	100%	YES	1.071	-1.054	YES	0.284	NO	3.515
**6g**	0.957	100%	YES	0.994	-0.857	YES	0.258	NO	3.196
**6h**	0.956	100%	YES	1.301	-0.811	YES	0.176	NO	4.176
**6i**	0.916	100%	NO	0.837	-1.08	YES	0.2	NO	3.185
**6j**	0.949	100%	YES	1.201	-0.908	YES	0.191	NO	3.68
**6k**	0.944	100%	NO	1.192	-0.934	YES	0.074	NO	3.493
**6l**	0.898	100%	NO	1.186	-0.968	YES	-0.112	NO	3.028
**6m**	0.896	100%	NO	1.184	-0.968	YES	-0.134	NO	3.031
**6n**	0.934	100%	NO	-0.241	-1.043	YES	0.309	NO	3.571
**6o**	0.926	100%	YES	0.883	-1.12	YES	0.195	NO	3.109
**6p**	0.95	100%	YES	1.056	-0.836	YES	0.231	NO	3.62
**6q**	0.936	100%	YES	1.206	-0.97	YES	-0.052	NO	3.608
**6r**	0.87	100%	NO	1.225	-1.016	YES	-0.242	NO	3.116
**6s**	0.96	100%	NO	1.141	-1.106	YES	0.031	NO	3.26
Fluconazole	1.191	87.821	NO	-1.2	-3.221	NO	0.386	NO	2.21

C2P = Caco-2 permeability (log Papp in 10^{-6} cm/s, >0.90 indicates high permeability); HIA = Human intestinal absorption (% absorbed, >30% is better absorbed); P-gpI = P glycoprotein inhibitor; BBB is expressed in logBB (logBB >-1.0 is moderately cross blood brain barrier); CNS is expressed as logPS (logPS>-2.0 can easily penetrate the CNS); Total clearance is expressed in log ML/min/kg; Toxicity is calculated in oral rat acute toxicity (mol/kg).

Pharmacokinetic (ADME) and toxicity properties of the synthesized compounds were predicted using the pkCSM web server (https://biosig.lab.uq.edu.au/pkcsm) SMILES strings for all compounds were generated in ChemDraw and submitted to pkCSM. Predicted parameters included absorption (Caco-2 permeability, water solubility, intestinal absorption, P-gp substrate/inhibitor status), distribution (volume of distribution, unbound fraction, BBB penetration), metabolism (CYP450 enzyme inhibition/substrate), excretion (clearance, renal transport), and toxicity endpoints (AMES mutagenicity, LD_{50}, hepatotoxicity, etc.)[6]

Table S3. PASS Prediction (Pa/Pi values for major biological activities) data for compound (6a-6s).

Biological activity spectra (potential pharmacological effects, toxicity, etc.) of all synthesized compounds were predicted using PASS Online (https://way2drug.com/PassOnline/). SMILES strings for all compounds were generated from ChemDraw and submitted to PASS Online. Reported activities (probability of activity, Pa) and inactivity (Pi) values were directly extracted from the server output.[7]

Molecules	P_a [a]	P_i [b]	Activity
6a	0.849<br>0.722<br>0.683<br>0.566	0.017<br>0.033<br>0.045<br>0.047	Phobic disorders treatment<br>Antiseborrheic<br>Antineurotic<br>Antidyskinetic
6b	0.768<br>0.756<br>0.675<br>0.562	0.023<br>0.051<br>0.021<br>0.040	Antineurotic<br>Phobic disorders treatment<br>Antidyskinetic<br>Neurotransmitter uptake inhibitor
6c	0.898<br>0.770<br>0.745<br>0.611	0.005<br>0.025<br>0.028<br>0.036	Phobic disorders treatment<br>Antiseborrheic<br>Antineurotic<br>Antidyskinetic
6d	0.739<br>0.713<br>0.597	0.030<br>0.070<br>0.042	Antineurotic<br>Phobic disorders treatment<br>Kidney function stimulant
6e	0.832<br>0.718<br>0.637	0.003<br>0.068<br>0.059	Neurotransmitter uptake inhibitor<br>Phobic disorders treatment<br>Antineurotic
6f	0.657<br>0.515<br>0.418<br>0.446	0.096<br>0.055<br>0.027<br>0.078	Phobic disorders treatment<br>Neurotransmitter uptake inhibitor<br>Antituberculosis<br>Antiviral (Picornavirus)
6g	0.898<br>0.654<br>0.641<br>0.593	0.005<br>0.025<br>0.028<br>0.036	Phobic disorders treatment<br>Antiseborrheic<br>Antineurotic<br>Antidyskinetic
6h	0.681<br>0.785<br>0.581	0.040<br>0.080<br>0.032	Antineurotic<br>Phobic disorders treatment<br>Kidney function stimulant
6i	0.838<br>0.751<br>0.594	0.012<br>0.053<br>0.013	Antiseborrheic<br>Phobic disorders treatment<br>Neurotransmitter antagonist

6j	0.834<br>0.651<br>0.698<br>0.596	0.017<br>0.043<br>0.055<br>0.047	Phobic disorders treatment<br>Antiseborrheic<br>Antineurotic<br>Antidyskinetic
6k	0.772<br>0.722<br>0.638<br>0.538	0.022<br>0.066<br>0.029<br>0.047	Antineurotic<br>Phobic disorders treatment<br>Antidyskinetic<br>Neurotransmitter uptake inhibitor
6l	0.909<br>0.771<br>0.730<br>0.560	0.005<br>0.022<br>0.032<br>0.048	Phobic disorders treatment<br>Antineurotic<br>Antiseborrheic<br>Antidyskinetic
6m	0.697<br>0.672<br>0.538	0.042<br>0.089<br>0.047	Antineurotic<br>Phobic disorders treatment<br>Neurotransmitter uptake inhibitor
6n	0.832<br>0.718<br>0.637	0.003<br>0.068<br>0.059	Neurotransmitter uptake inhibitor<br>Phobic disorders treatment<br>Antineurotic
6o	0.563<br>0.549<br>0.469	0.040<br>0.090<br>0.078	Neurotransmitter uptake inhibitor<br>Antineurotic<br>Antiseborrheic
6p	0.748<br>0.730<br>0.694<br>0.515	0.028<br>0.063<br>0.039<br>0.055	Antineurotic<br>Phobic disorders treatment<br>Antiseborrheic<br>Neurotransmitter uptake inhibitor
6q	0.663<br>0.549<br>0.569	0.040<br>0.090<br>0.078	Neurotransmitter uptake inhibitor<br>Antineurotic<br>Antiseborrheic
6r	0.673<br>0.570<br>0.502<br>0.541	0.042<br>0.082<br>0.028<br>0.026	Antiseborrheic<br>Antineurotic<br>Antiallergic<br>Neurotransmitter antagonist
6s	0.848<br>0.780<br>0.674<br>0.675	0.028<br>0.063<br>0.039<br>0.055	Antineurotic<br>Phobic disorders treatment<br>Antiseborrheic<br>Neurotransmitter uptake inhibitor

Pa = Probability to be active, Pi = probability to be inactive

Table S4. **Predicted Antiviral Activity Scores data for compound (6a-6s).**

Antiviral-compound (AVC) predictions were conducted using the AVCpred web server (http://crdd.osdd.net/servers/avcpred/; RRID:SCR_018505). SMILES strings of all compounds were submitted to AVCpred, and predicted inhibition percentages/ antiviral-potential values were taken directly from the server output.[8]

Drug	General	HBV	HCV	HHV	HIV
6a	67.949	19.549	13.586	13.586	52.369
6b	65.796	28.615	41.929	6.605	64.522
6c	65.796	28.114	41.833	32.341	64.469
6d	67.961	19.354	38.238	13.395	52.368
6e	41.949	17.324	42.233	9.984	52.432
6f	61.986	21.097	27.915	4.642	52.432
6g	28.719	25.033	37.029	24.884	76.154
6h	61.986	21.097	27.915	4.642	52.432
6i	69.82	19.424	34.899	13.241	52.368
6j	69.679	19.349	33.876	13.395	52.368
6k	45.126	23.3	43.781	57.256	52.432
6l	27.999	29.703	43.011	29.757	64.522
6m	36.555	17.356	41.629	37.014	52.368
6n	51.784	22.766	38.248	35.652	76.154
6o	69.82	19.42	34.906	9.824	52.432
6p	56.207	16.339	30.204	4.24	52.369
6q	24.77	20.566	37.416	44.303	52.368
6r	25.581	20.203	47.496	21.416	76.154
6s	27.999	29.703	43.011	29.757	59.08

HBV=Hepatitis B virus; HCV=Hepatitis C virus; HHV=Human herpesvirus; HIV=Human immunodeficiency virus.

Molecular docking and 2D/3D interaction analyses with Human Cyclooxygenase-2 using Discovery Studio Visualizer for compound (6a-6s)

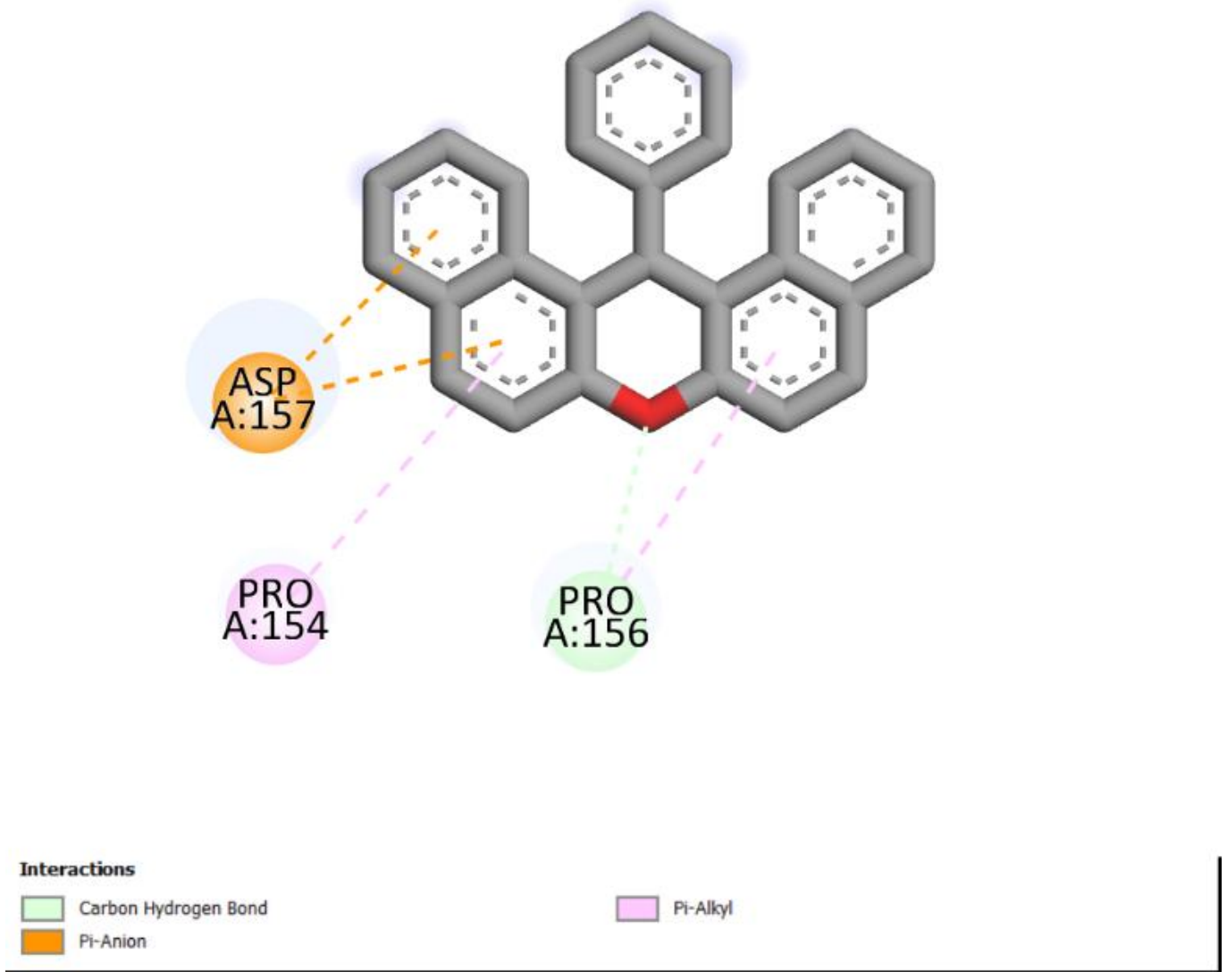

Fig. S62. Compound 6a. 2D-Interaction of ligand-protein

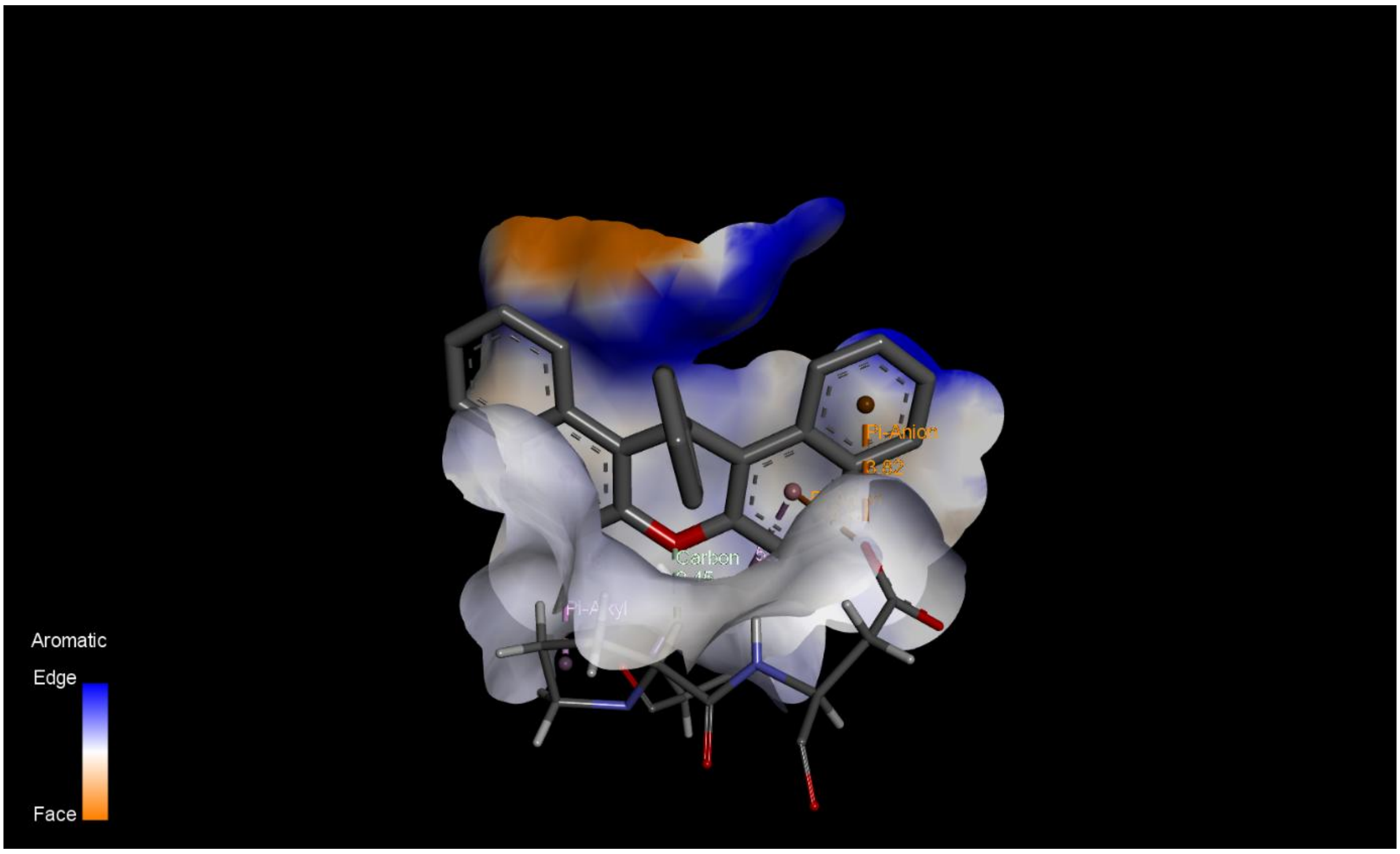

Fig. S63. Compound 6a. Visualization of aromatic face-edge interaction

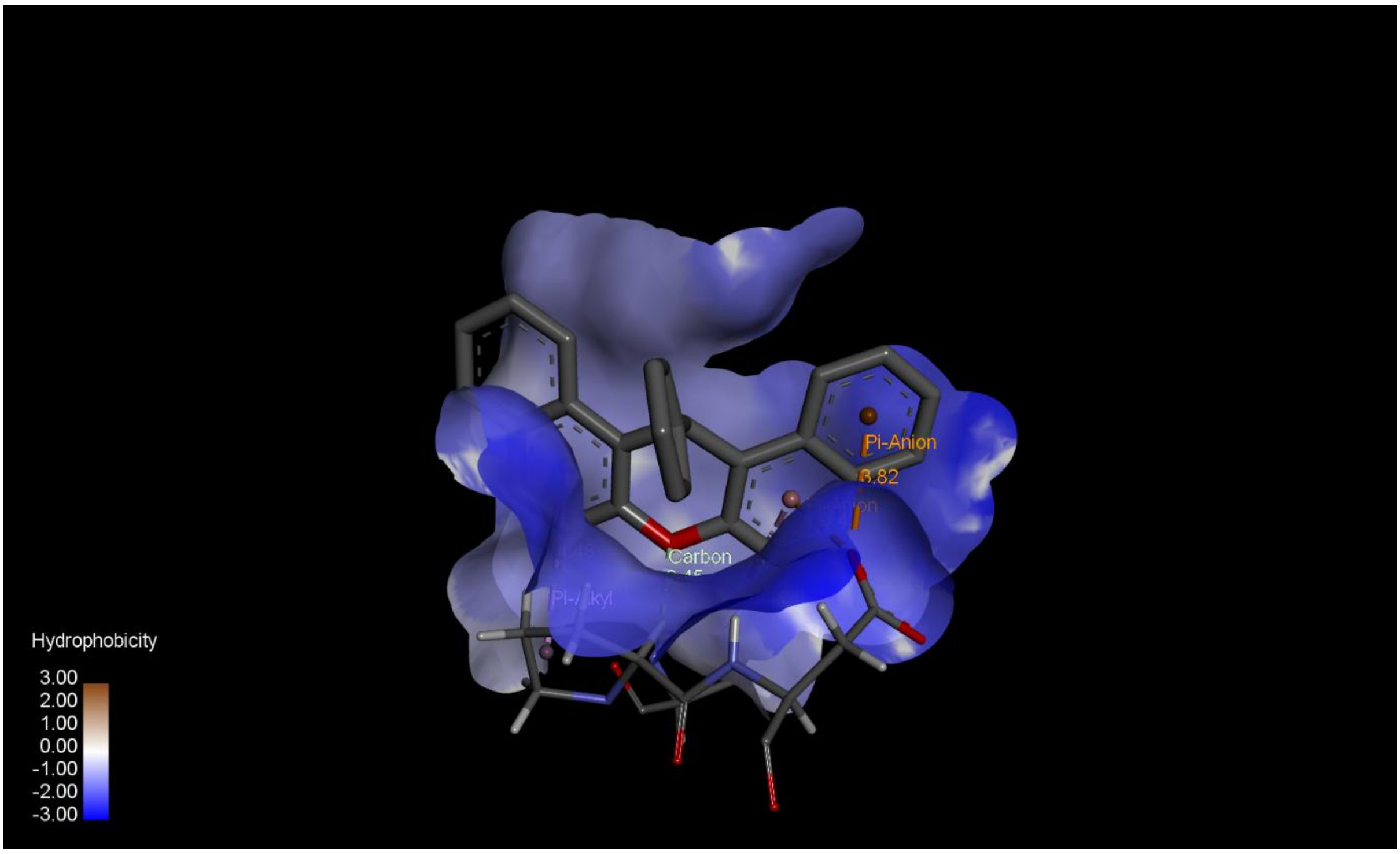

Fig. S64. Compound 6a. Visualization of hydrophobic interaction in the active site

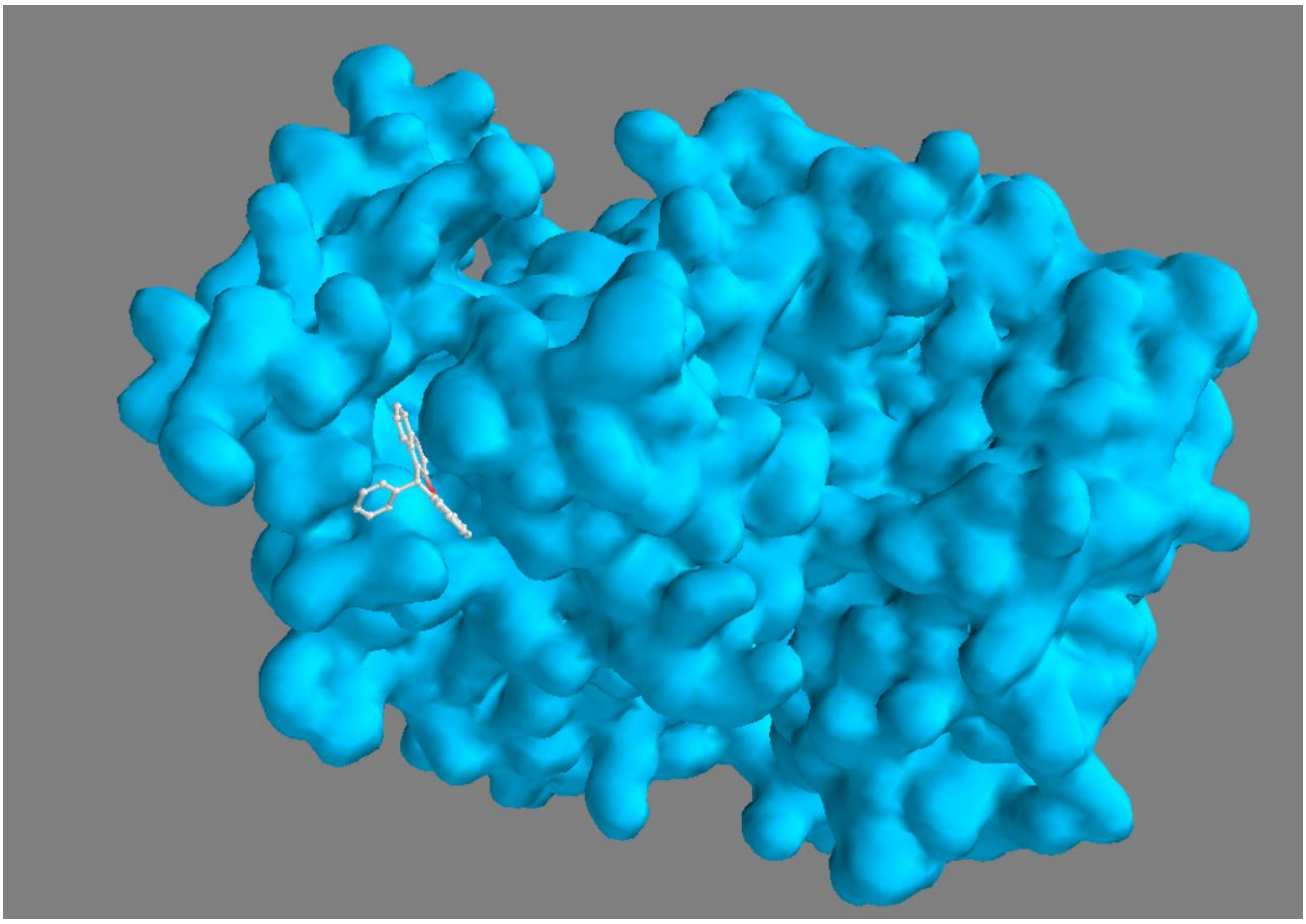

Fig. S65: Compound 6a. Protein surface visualization with bounded ligand

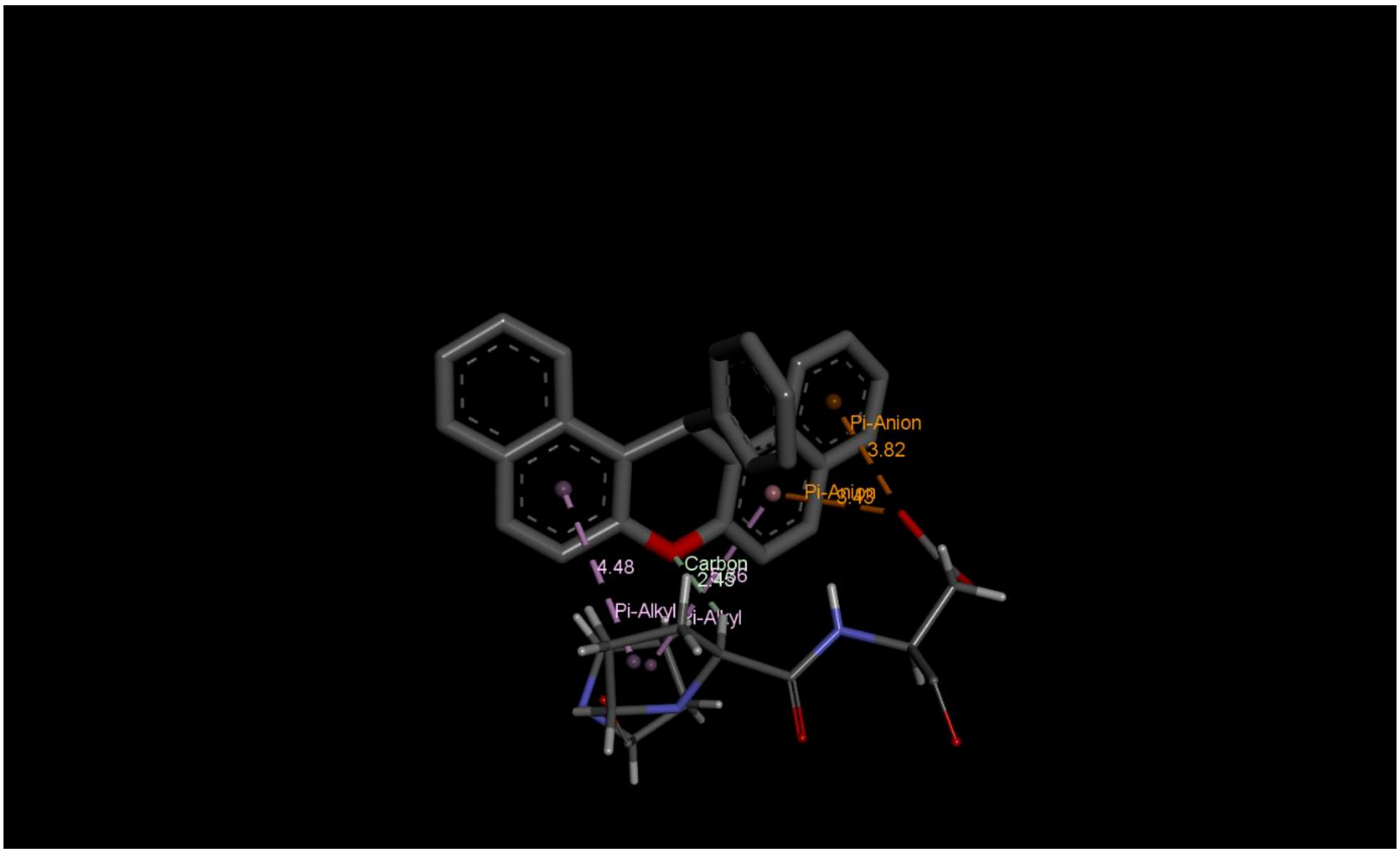

Fig. S66. Compound 6a. Binding pose of the ligand with the active site

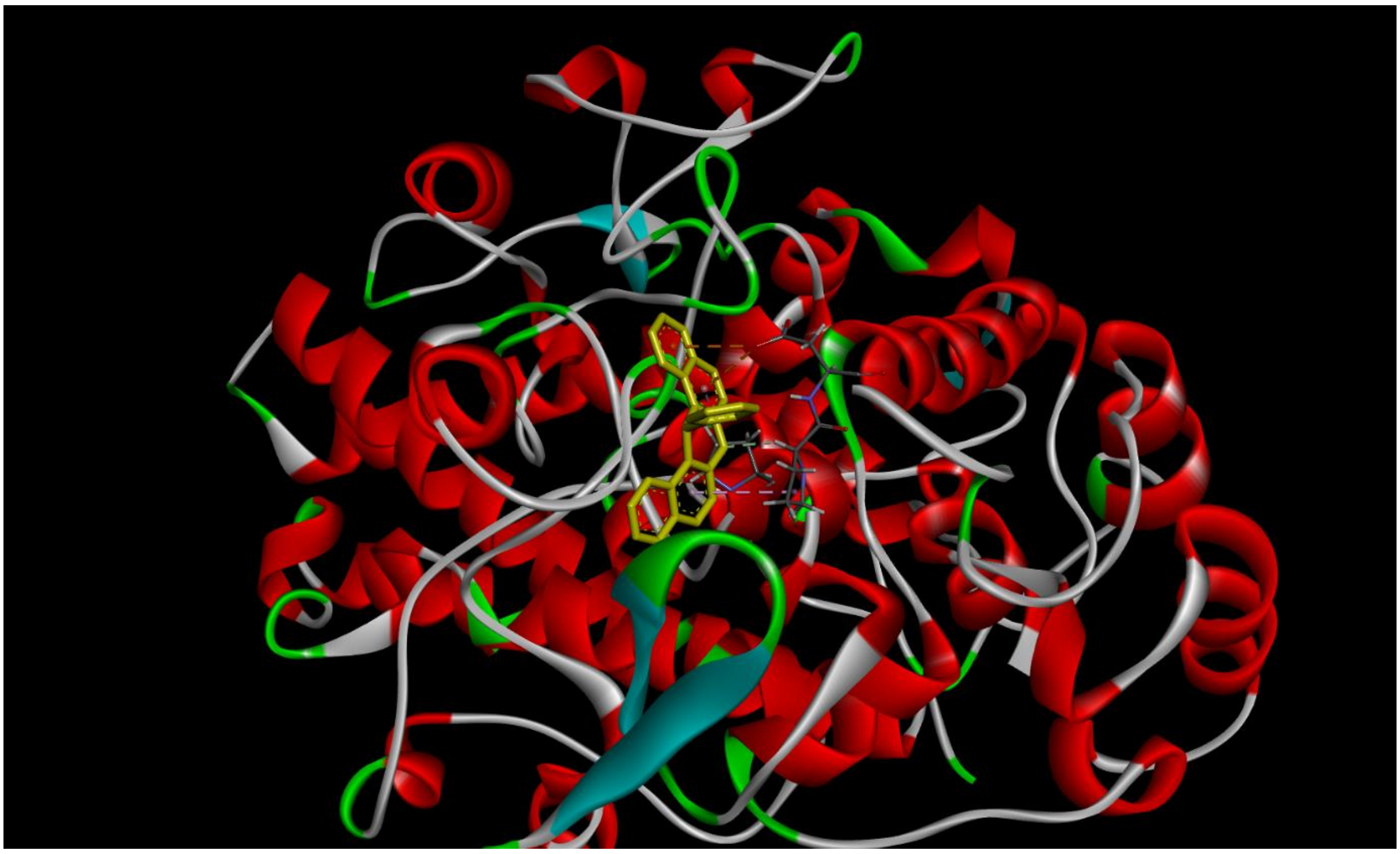

Fig. S67. Compound 6a. Ribbon diagram of the protein-ligand interaction

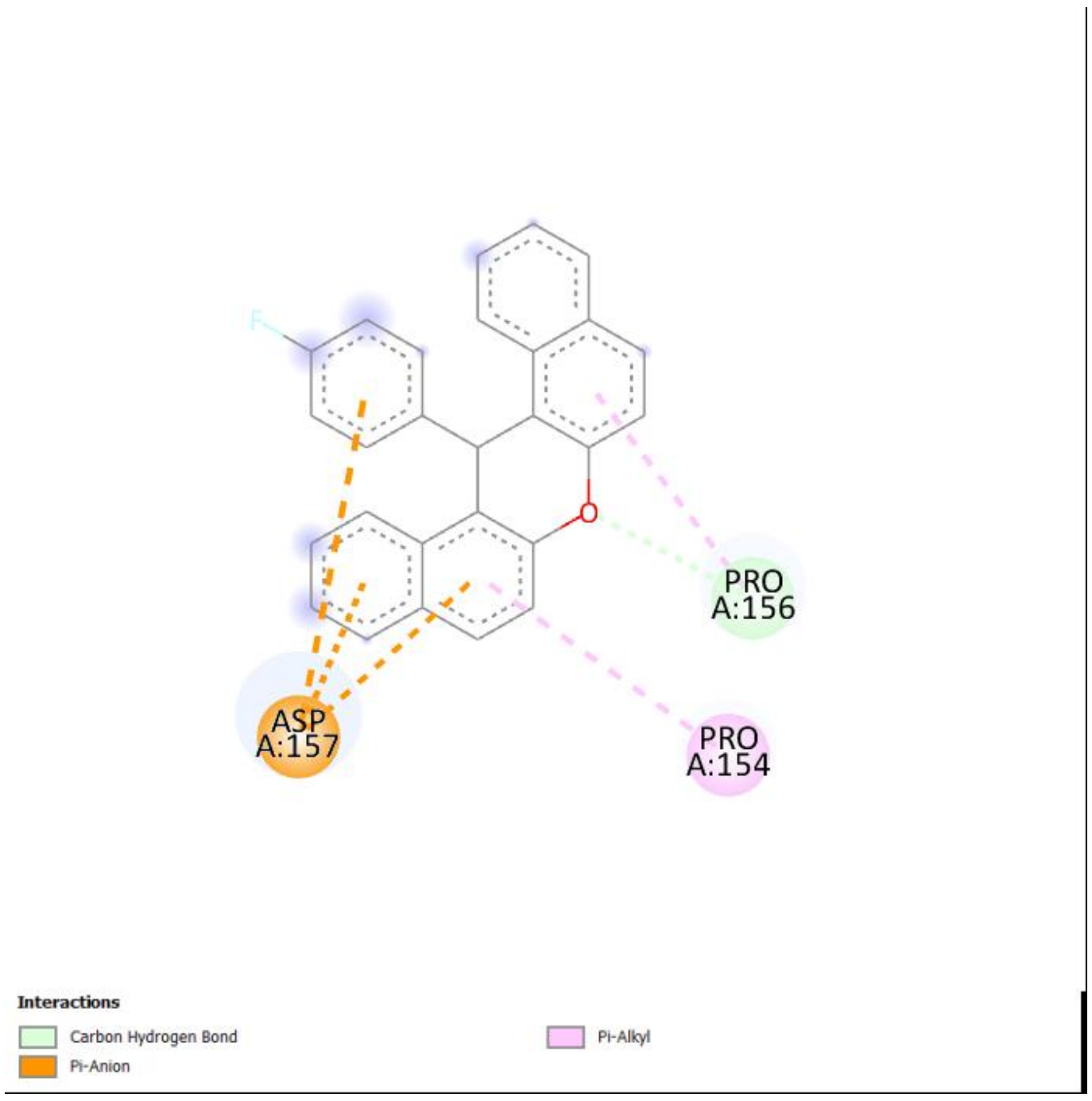

Fig. S68: Compound 6b. 2D-Interaction of ligand-protein

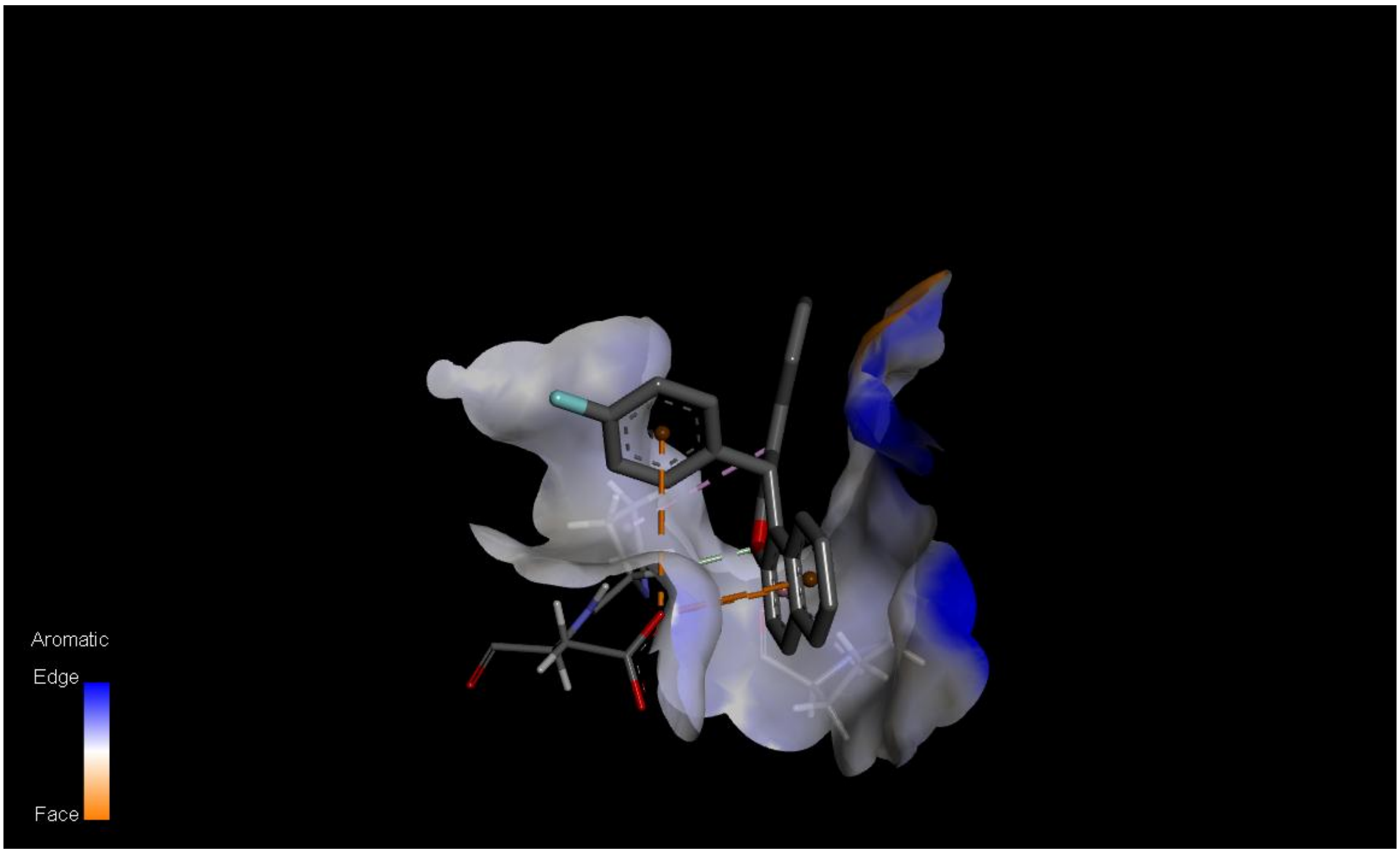

Fig. S69: Compound 6b. Visualization of aromatic face-edge interaction

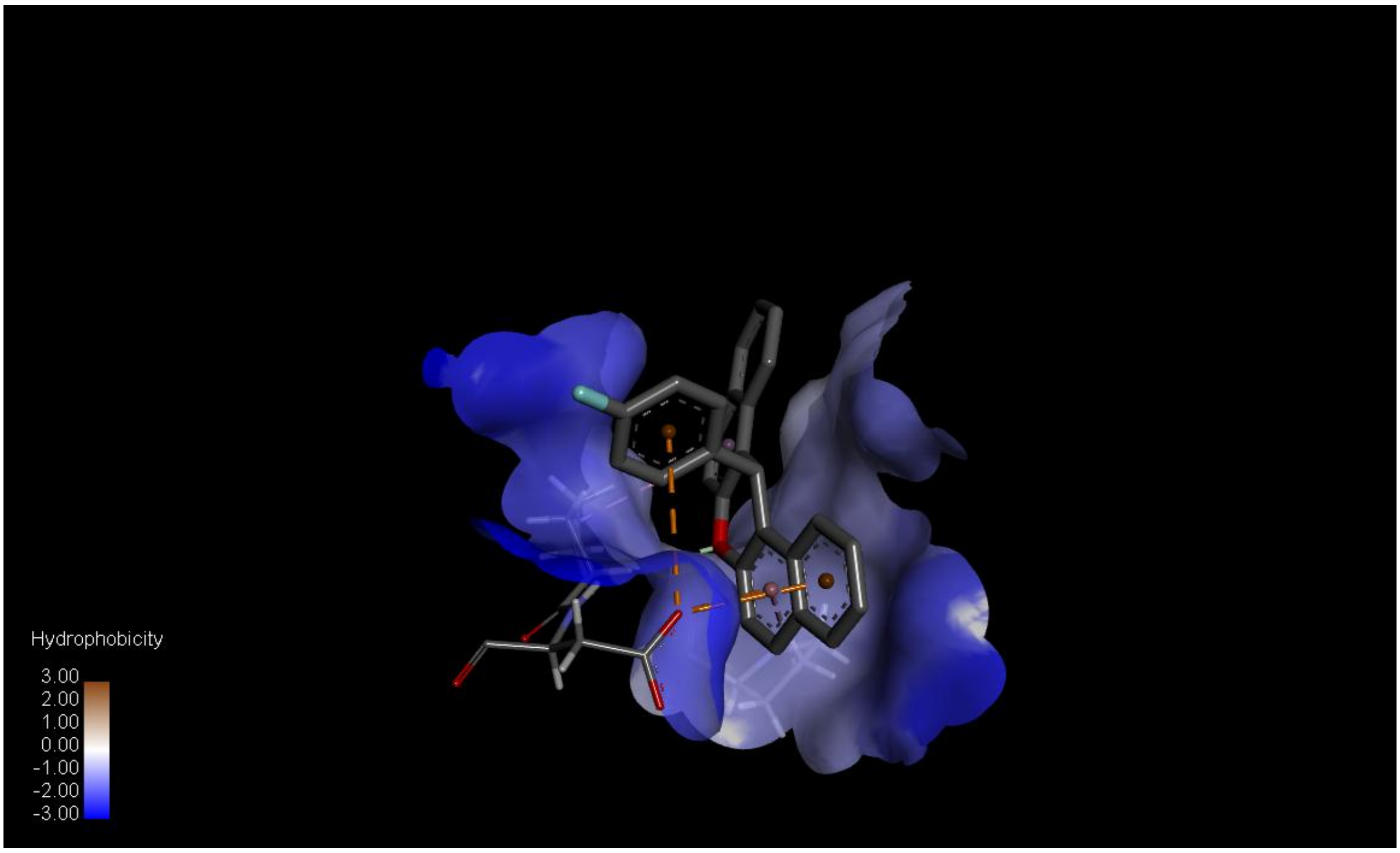

Fig. S70: Compound 6b. Visualization of hydrophobic interaction in the active site

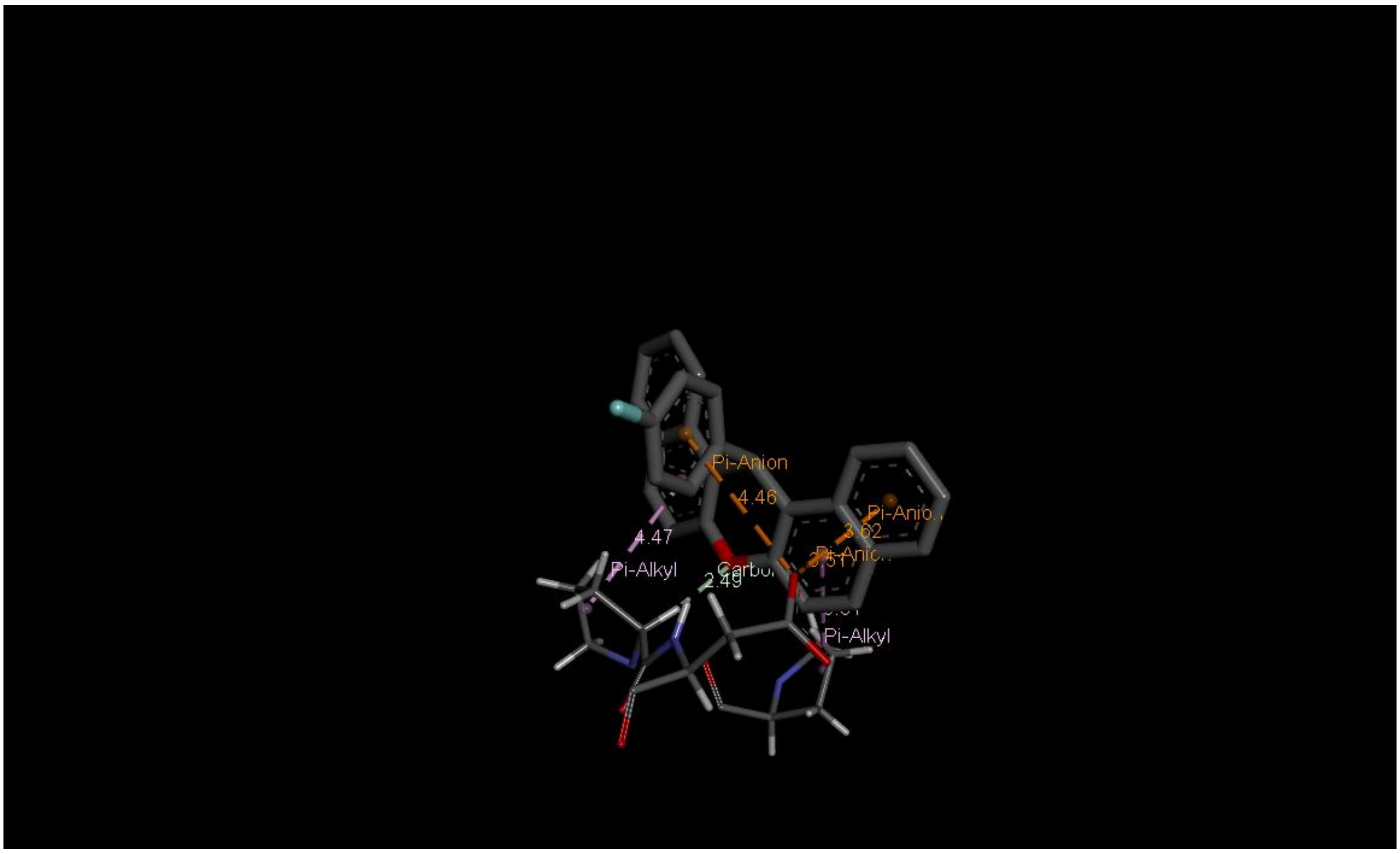

Fig. S71: Compound 6b. Binding pose of the ligand with the active site

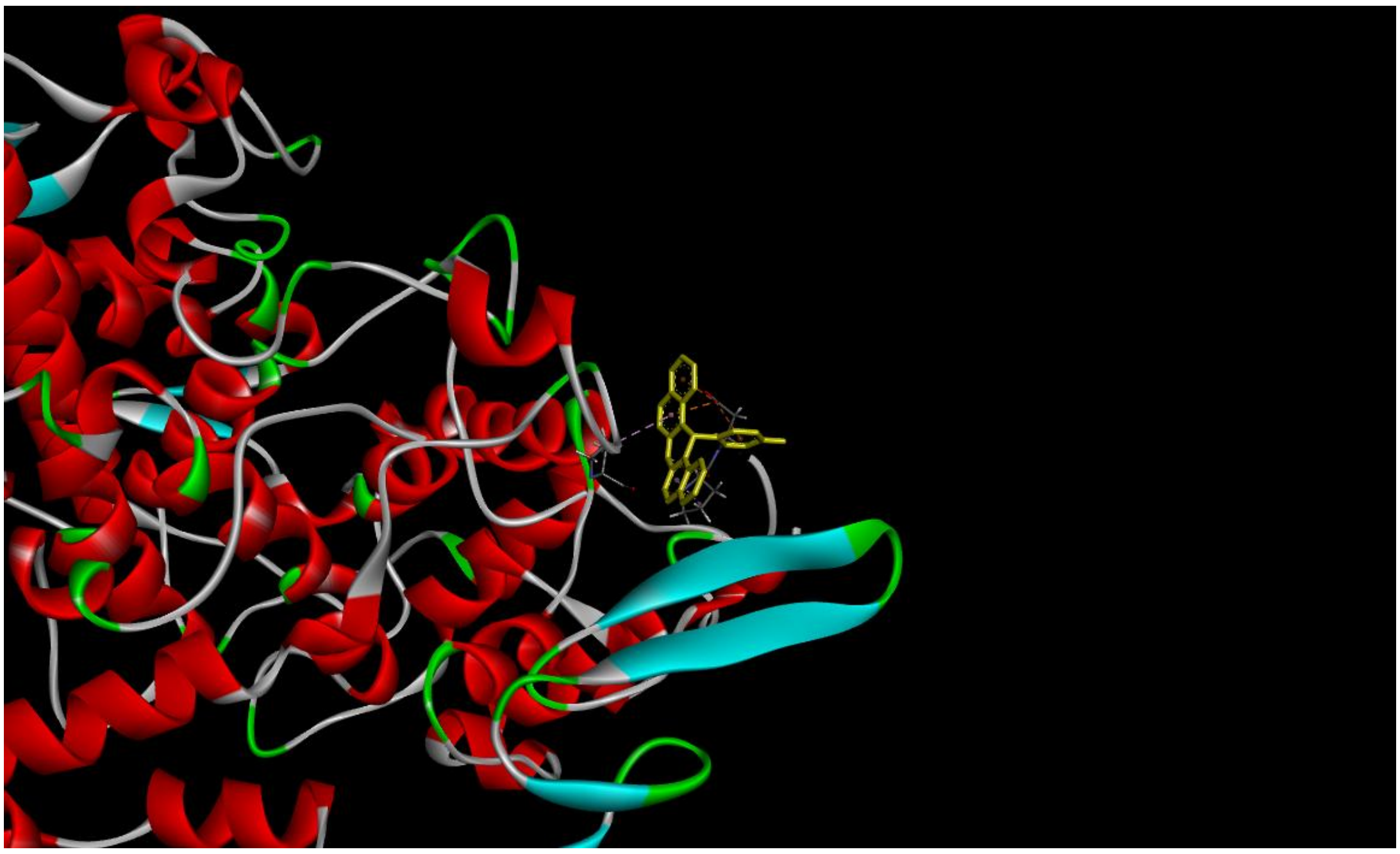

Fig. S72: Compound 6b. Ribbon diagram of the protein-ligand interaction

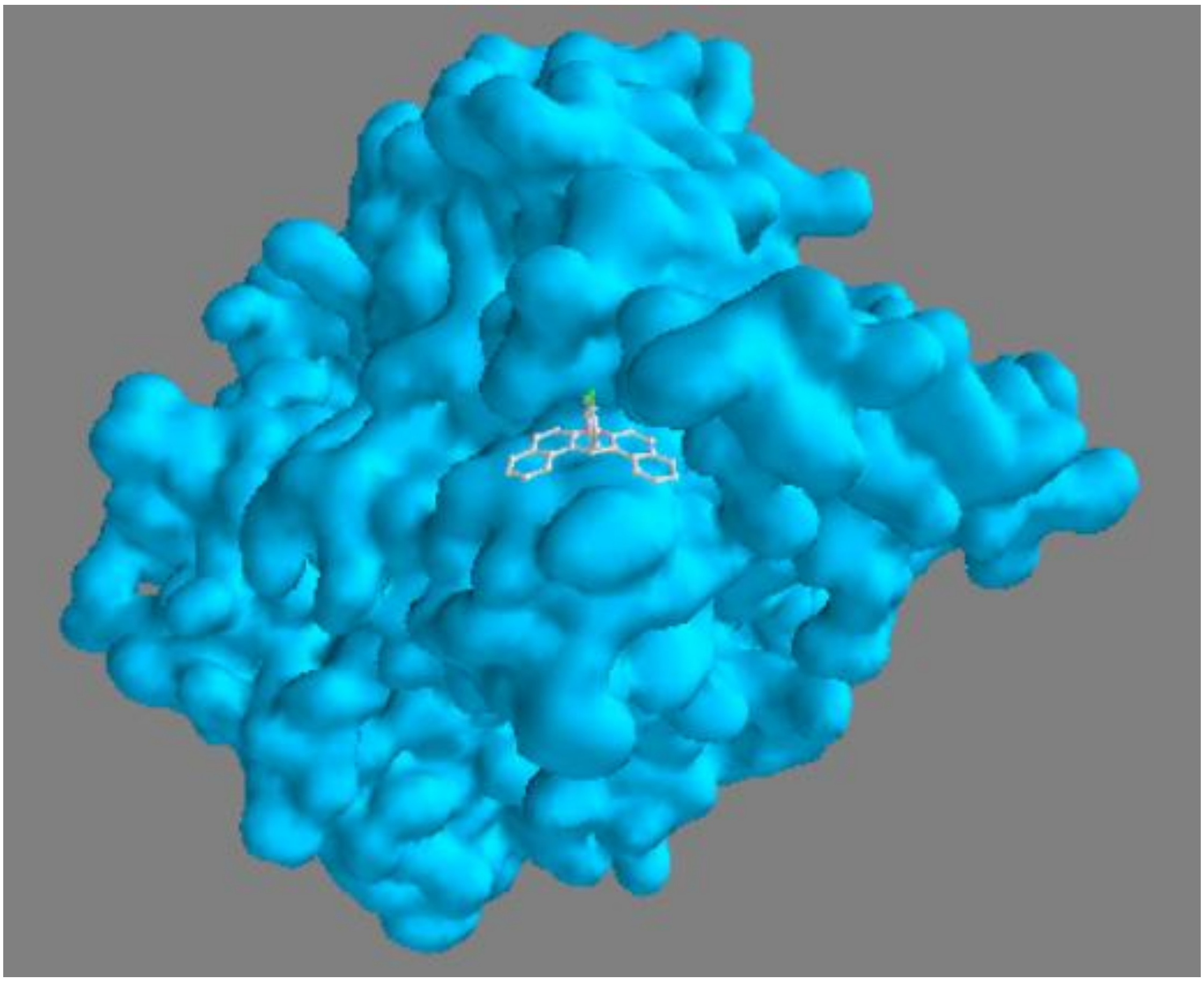

Fig. S73: Compound 6b. Protein surface visualization with bounded ligand

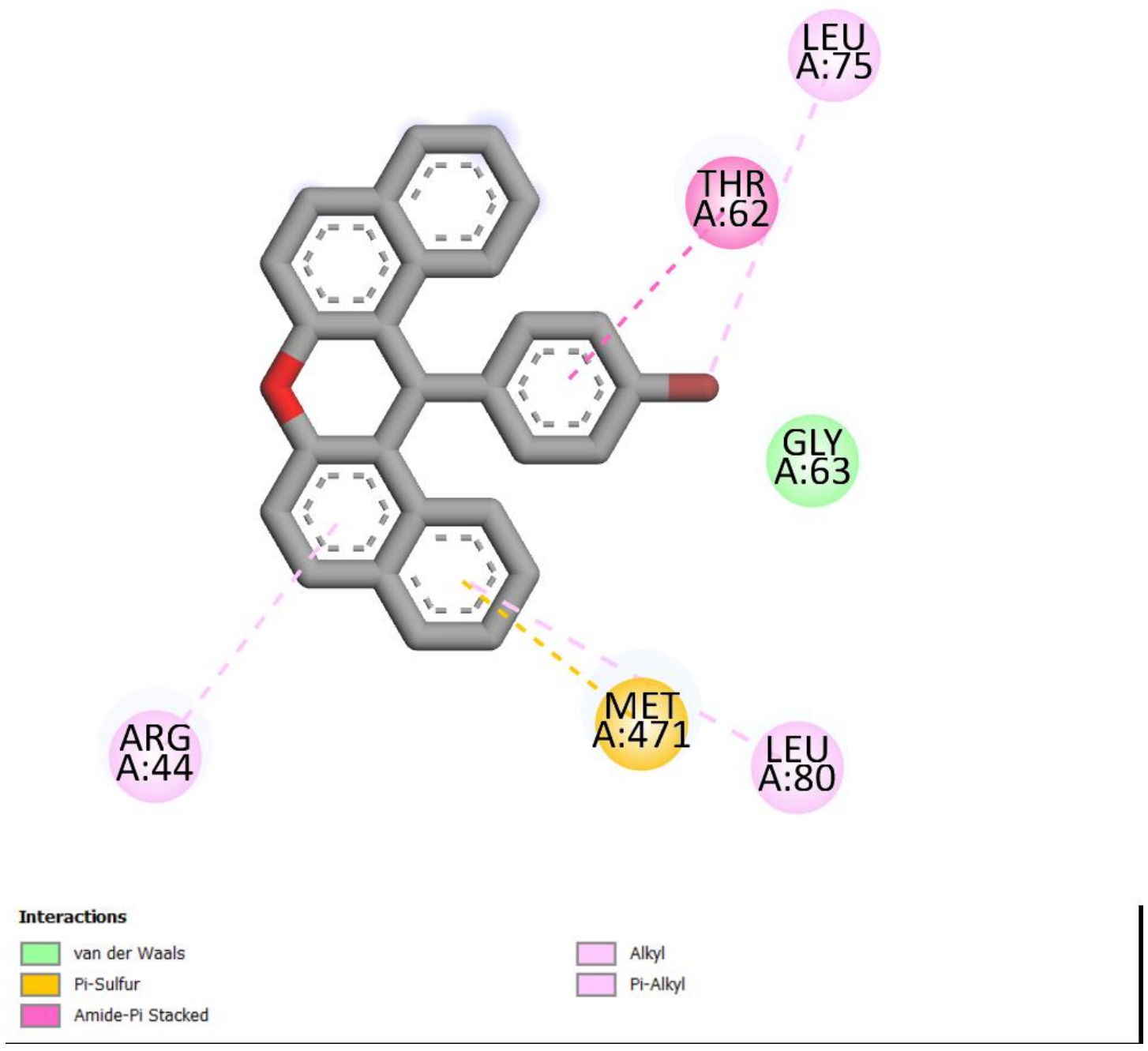

Fig. S74. Compound 6c .2D-Interaction of ligand-protein

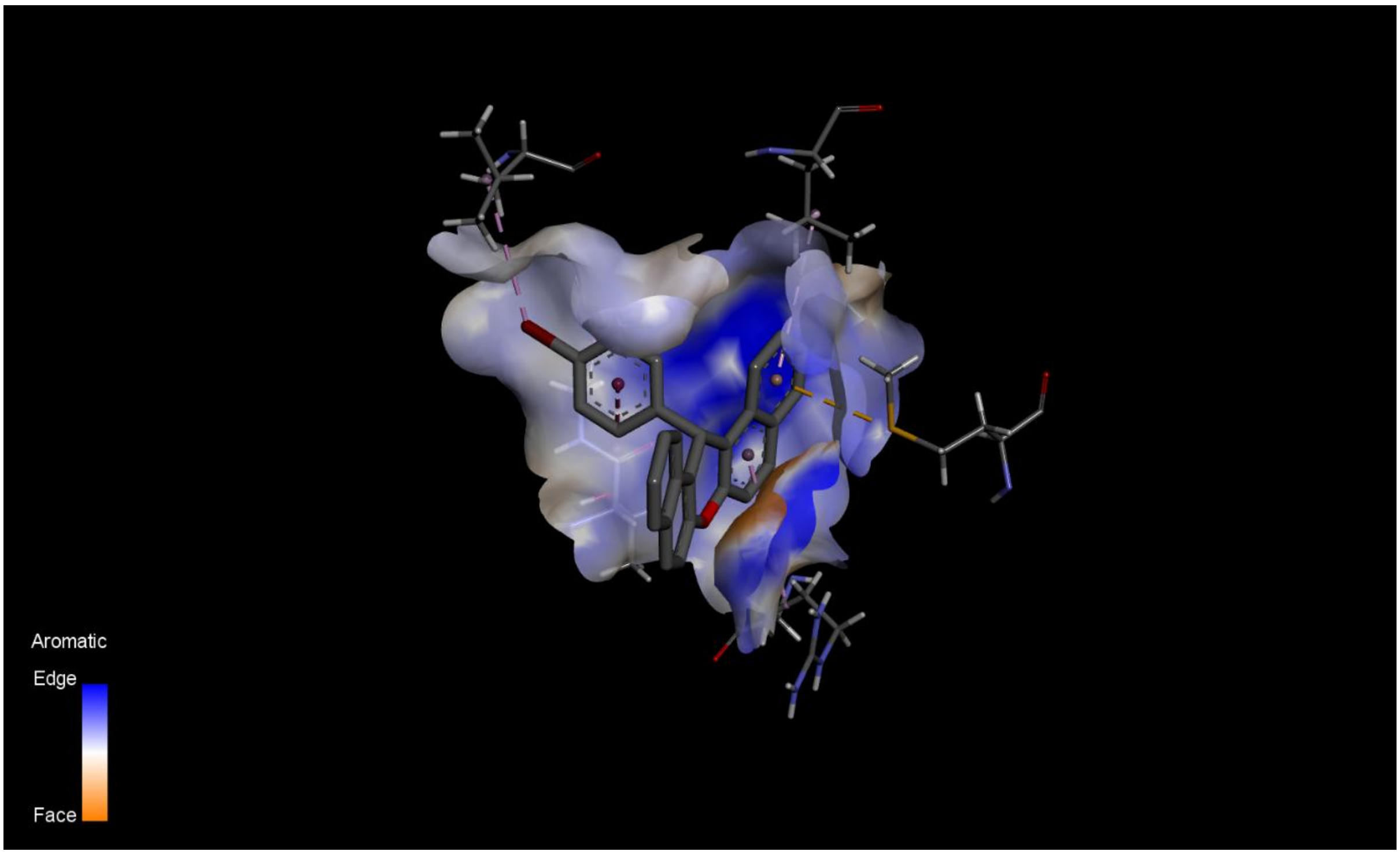

Fig. S75. Compound 6c. Visualization of aromatic face-edge interaction

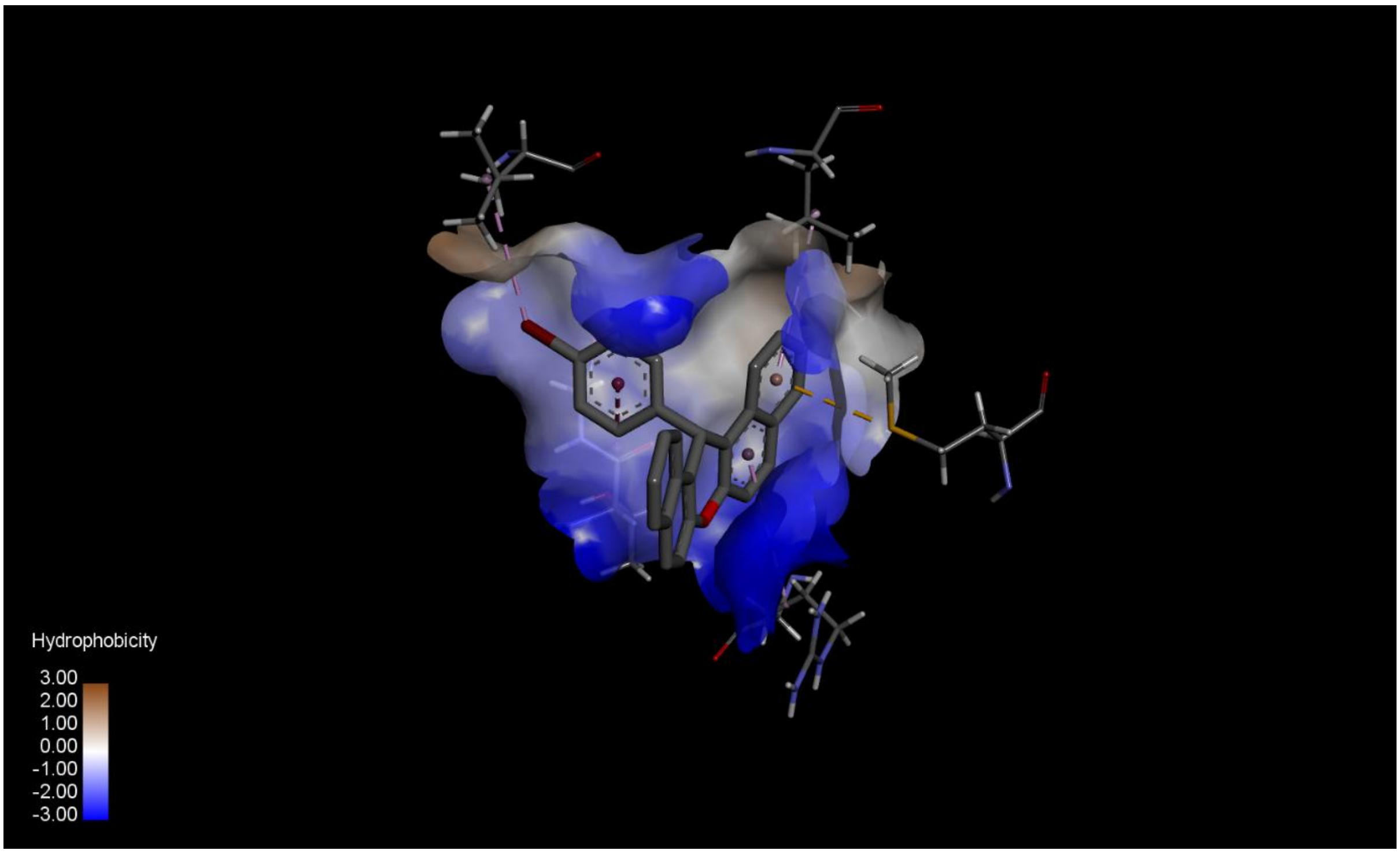

Fig. S76. Compound 6c .2D- Visualization of hydrophobic interaction in the active site

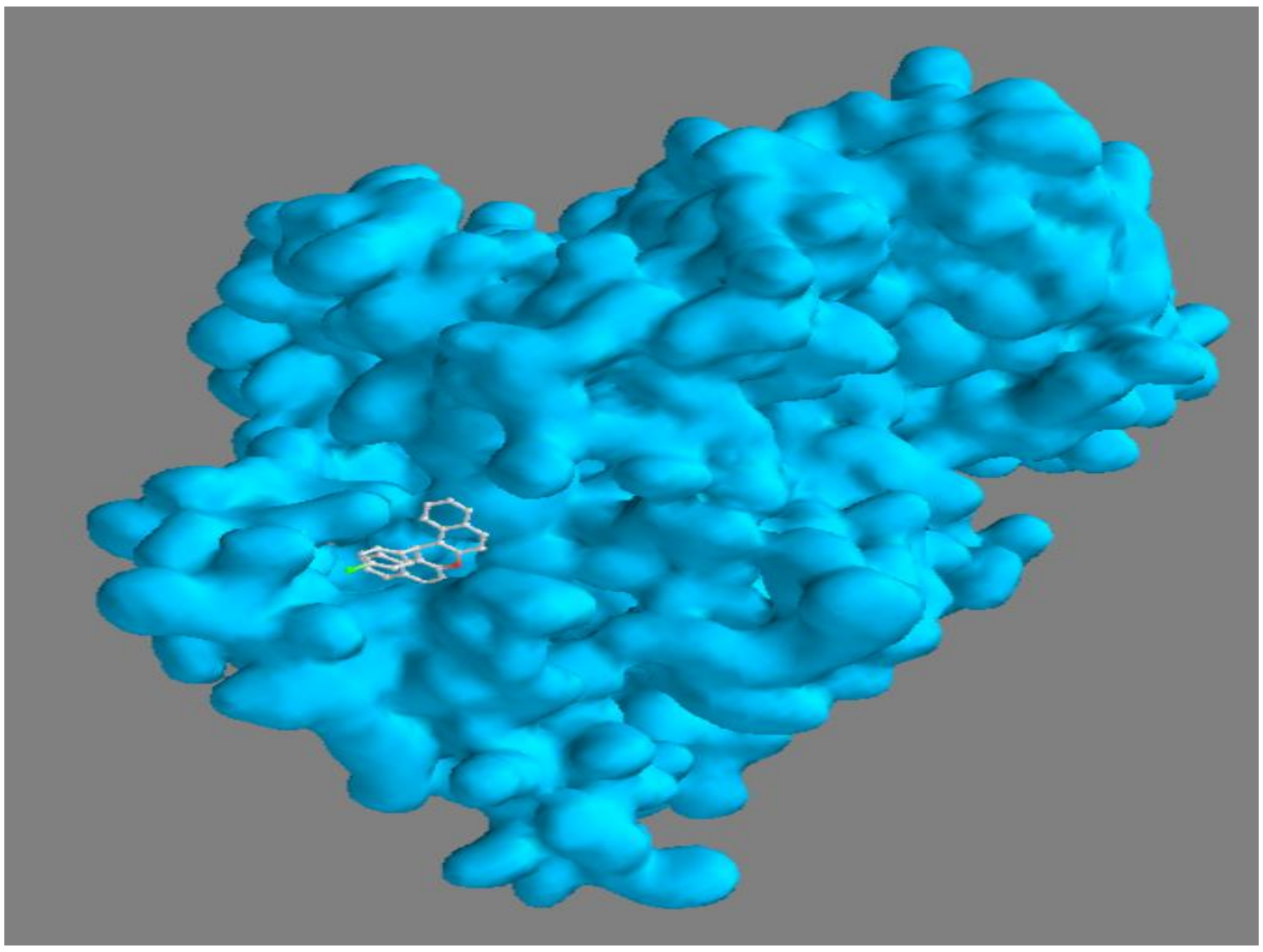

Fig. S77. Compound 6c.2D- Protein surface visualization with bounded ligand

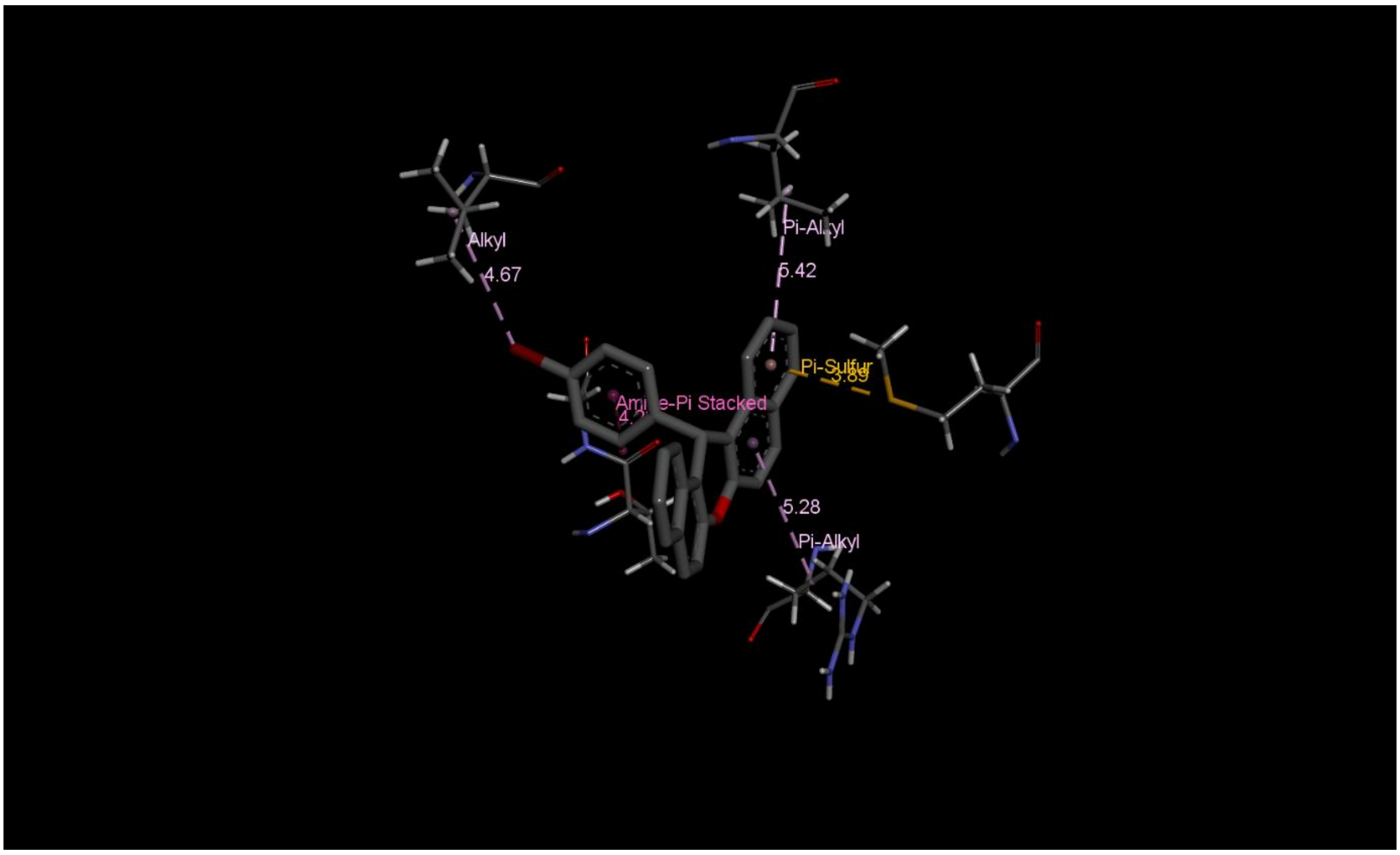

Fig. S78. Compound 6c. Binding pose of the ligand with the active site

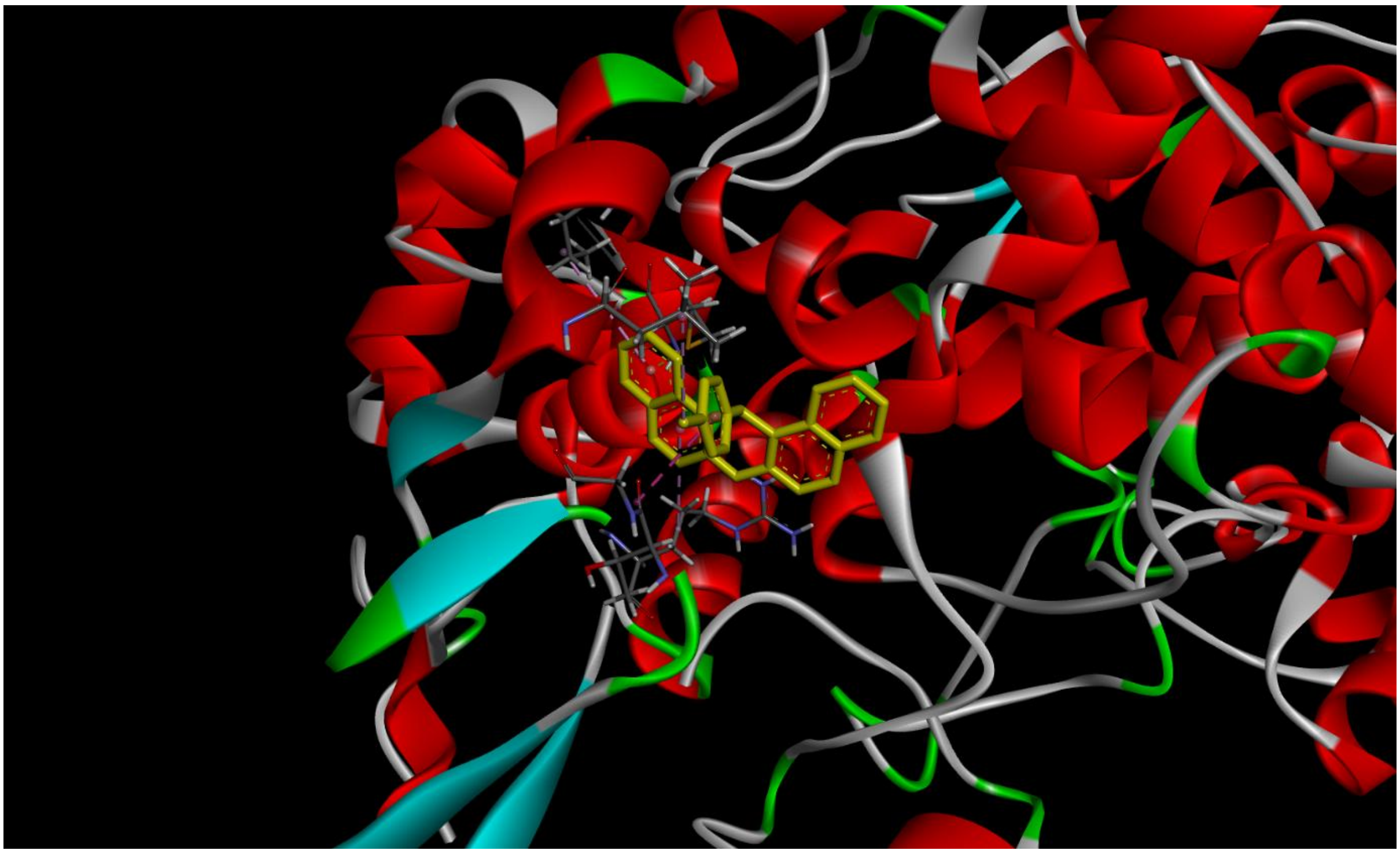

Fig. S79. Compound 6c. Ribbon diagram of the protein-ligand interaction

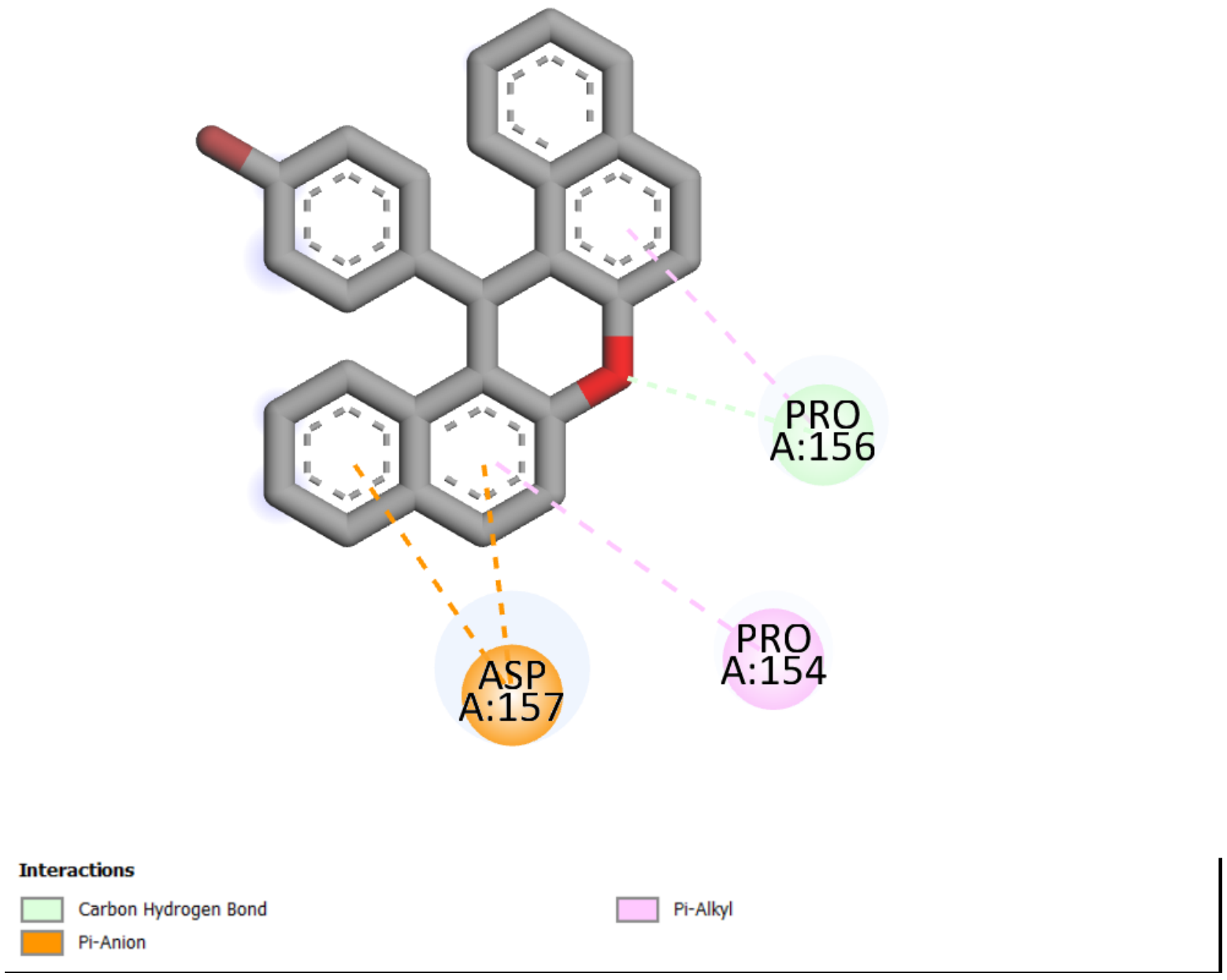

Fig. S80. Compound 6d.2D-Interaction of ligand-protein

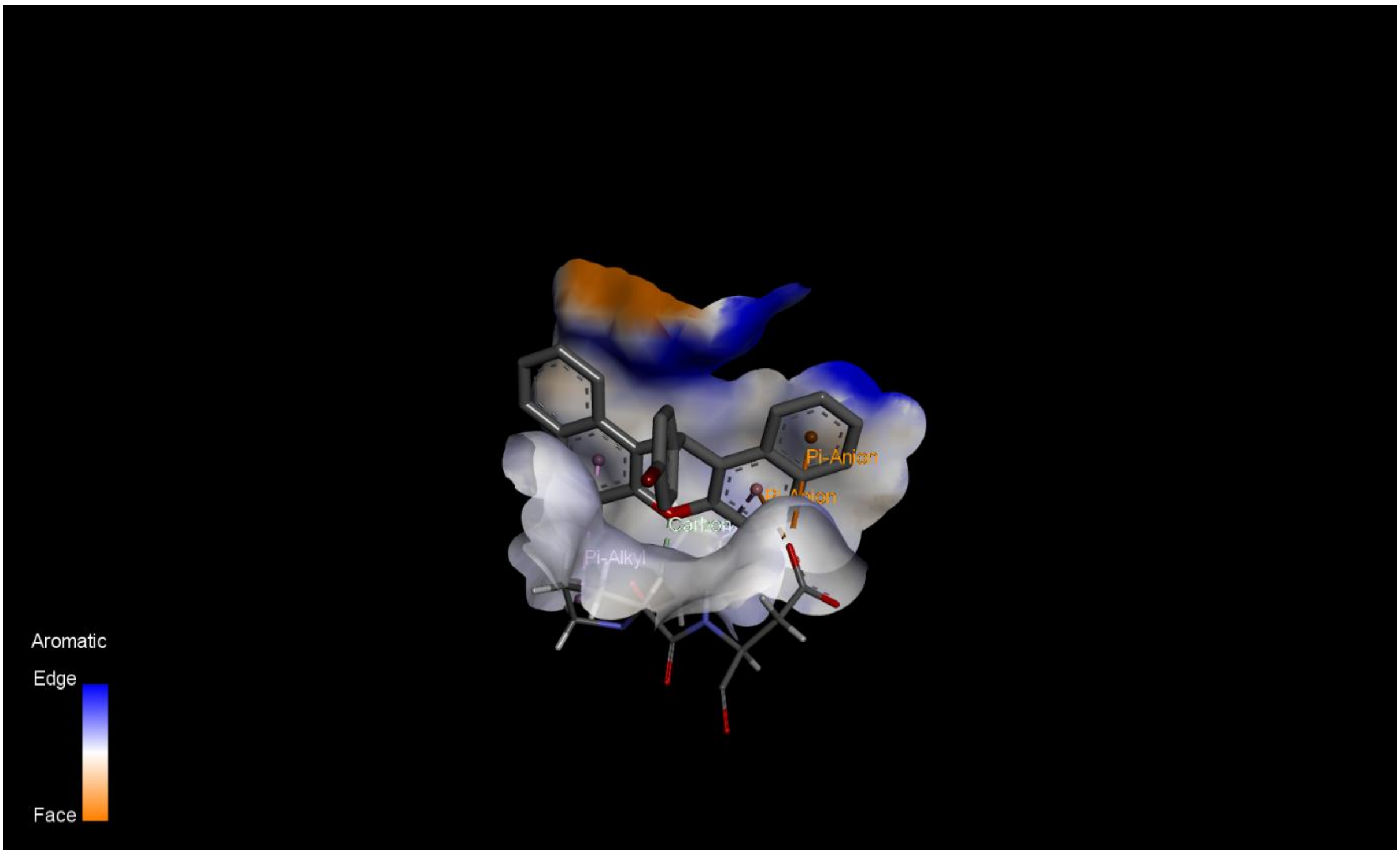

Fig. S81. Compound 6d. Visualization of aromatic face-edge interaction

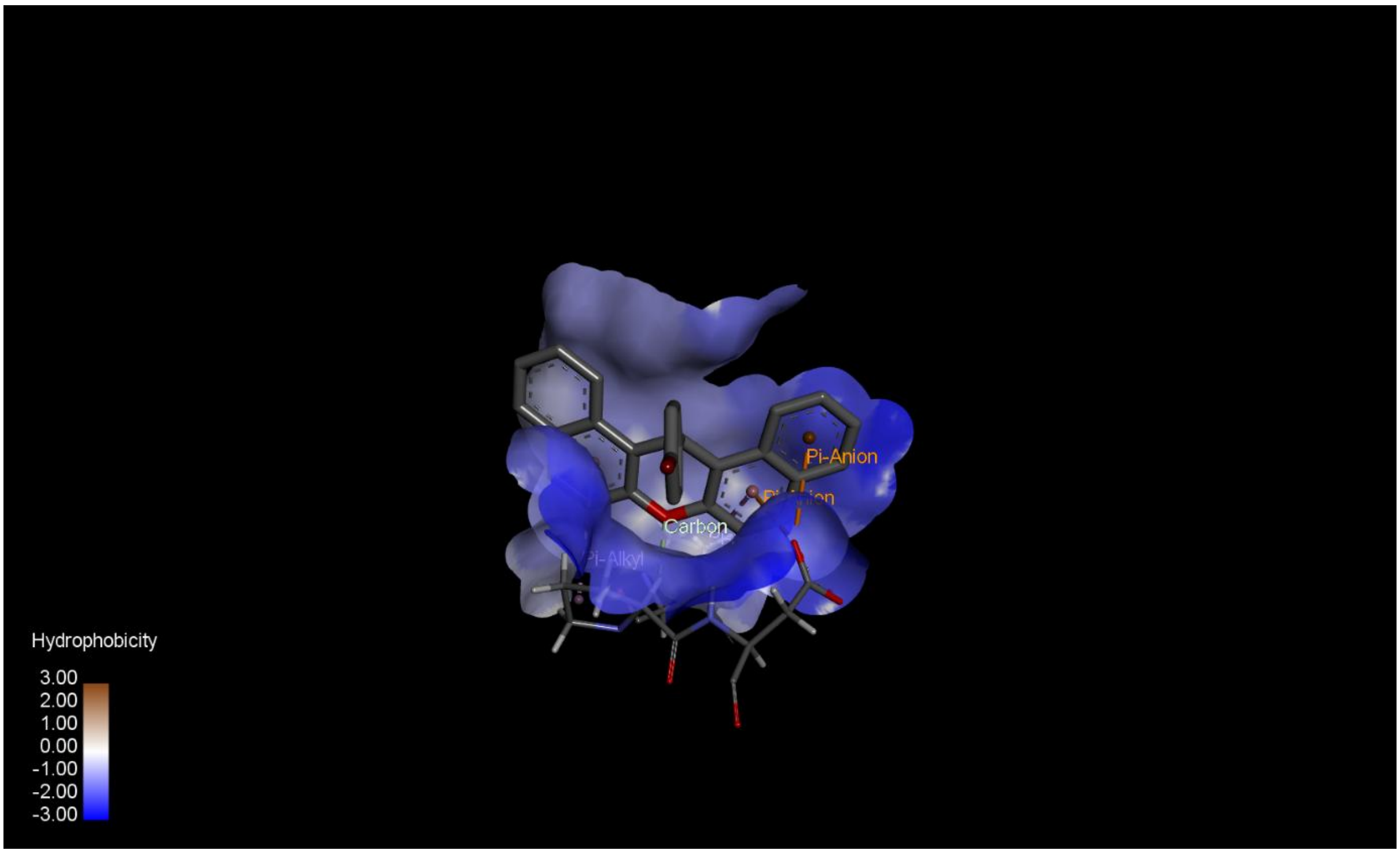

Fig. S82. Compound 6d Visualization of hydrophobic interaction in the active site

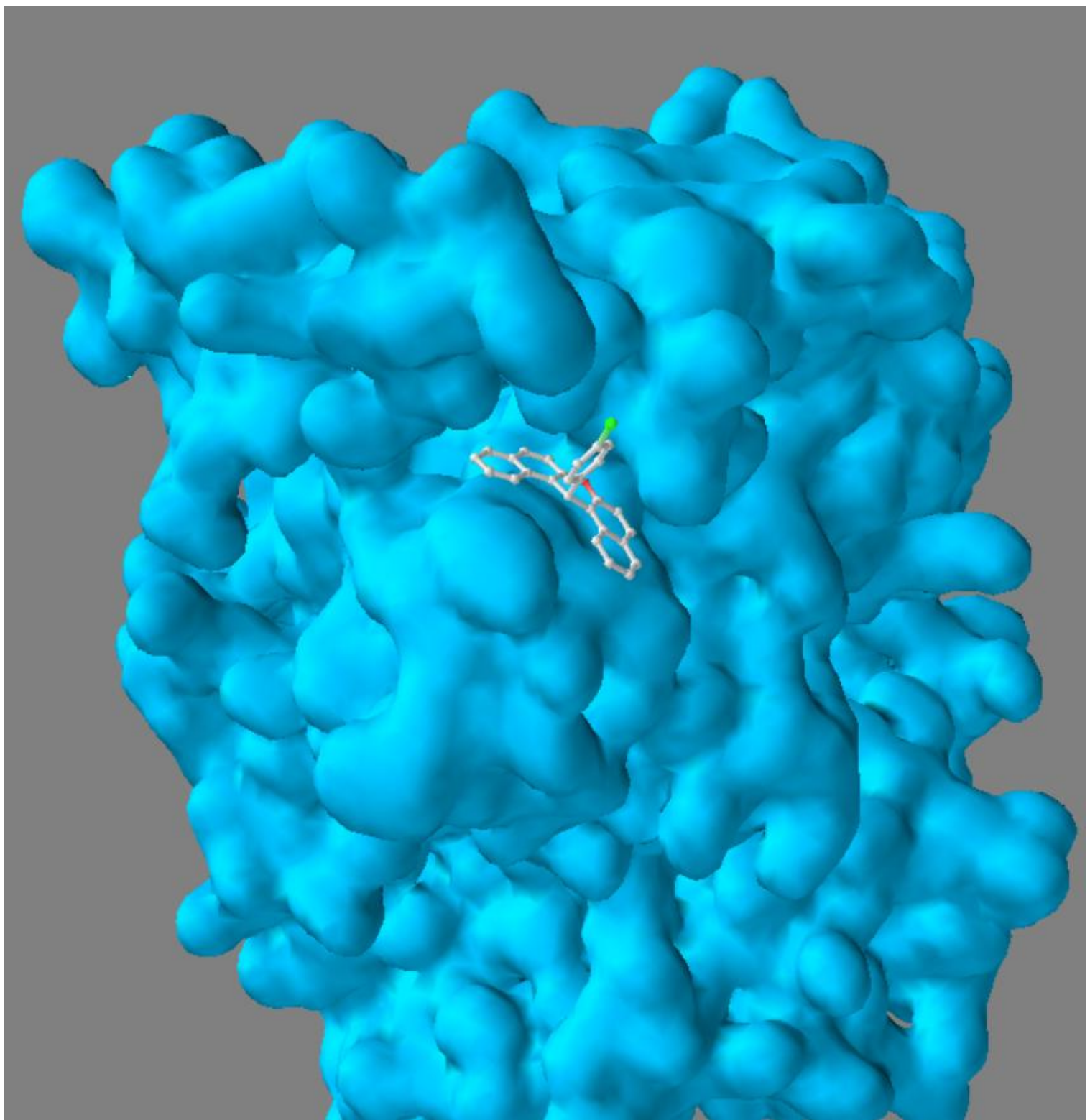

Fig. S83: Compound 6d. Protein surface visualization with bounded ligand

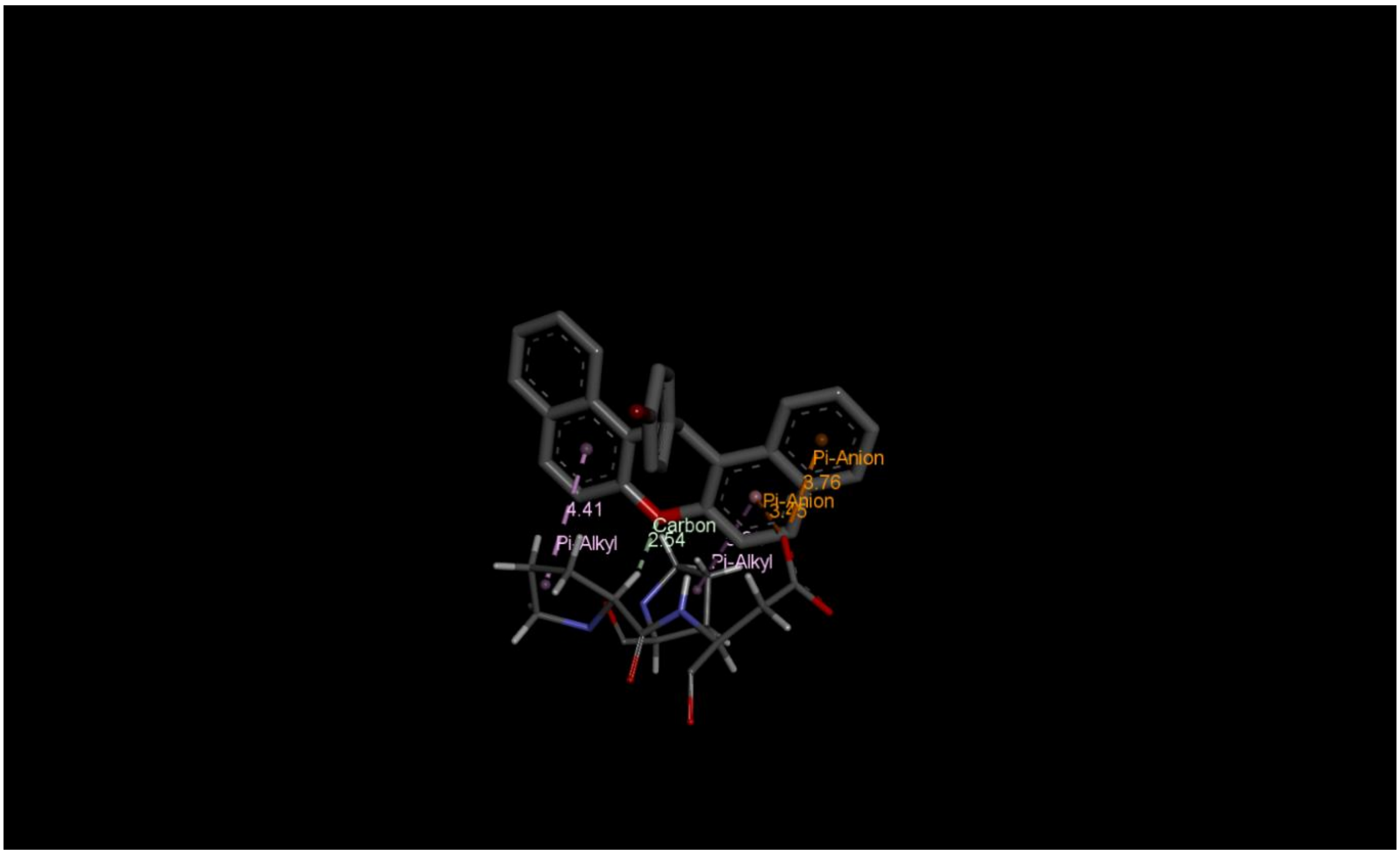

Fig. S84. Compound 6d. Binding pose of the ligand with the active site

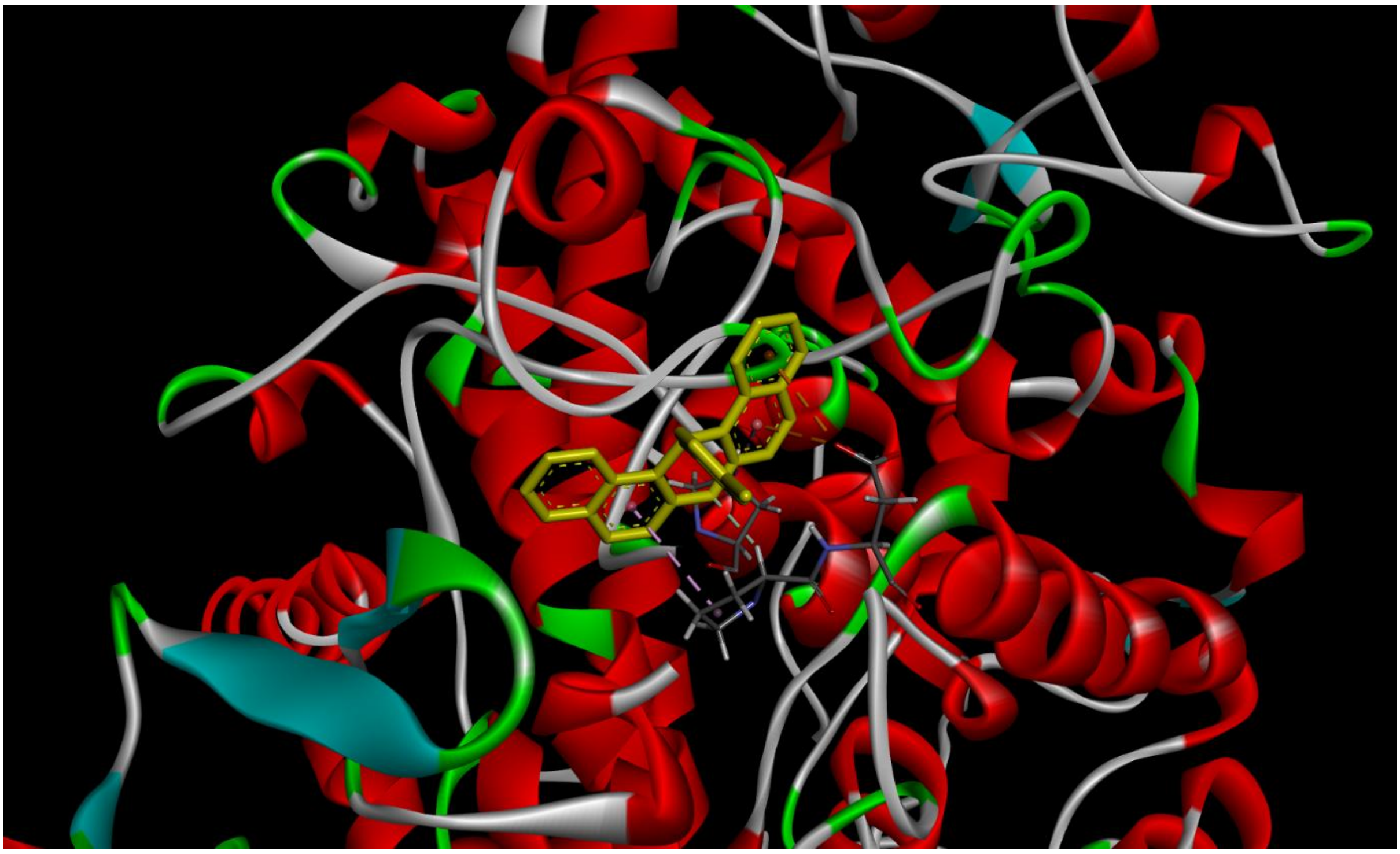

Fig. S85. Compound 6d. Ribbon diagram of the protein-ligand interaction

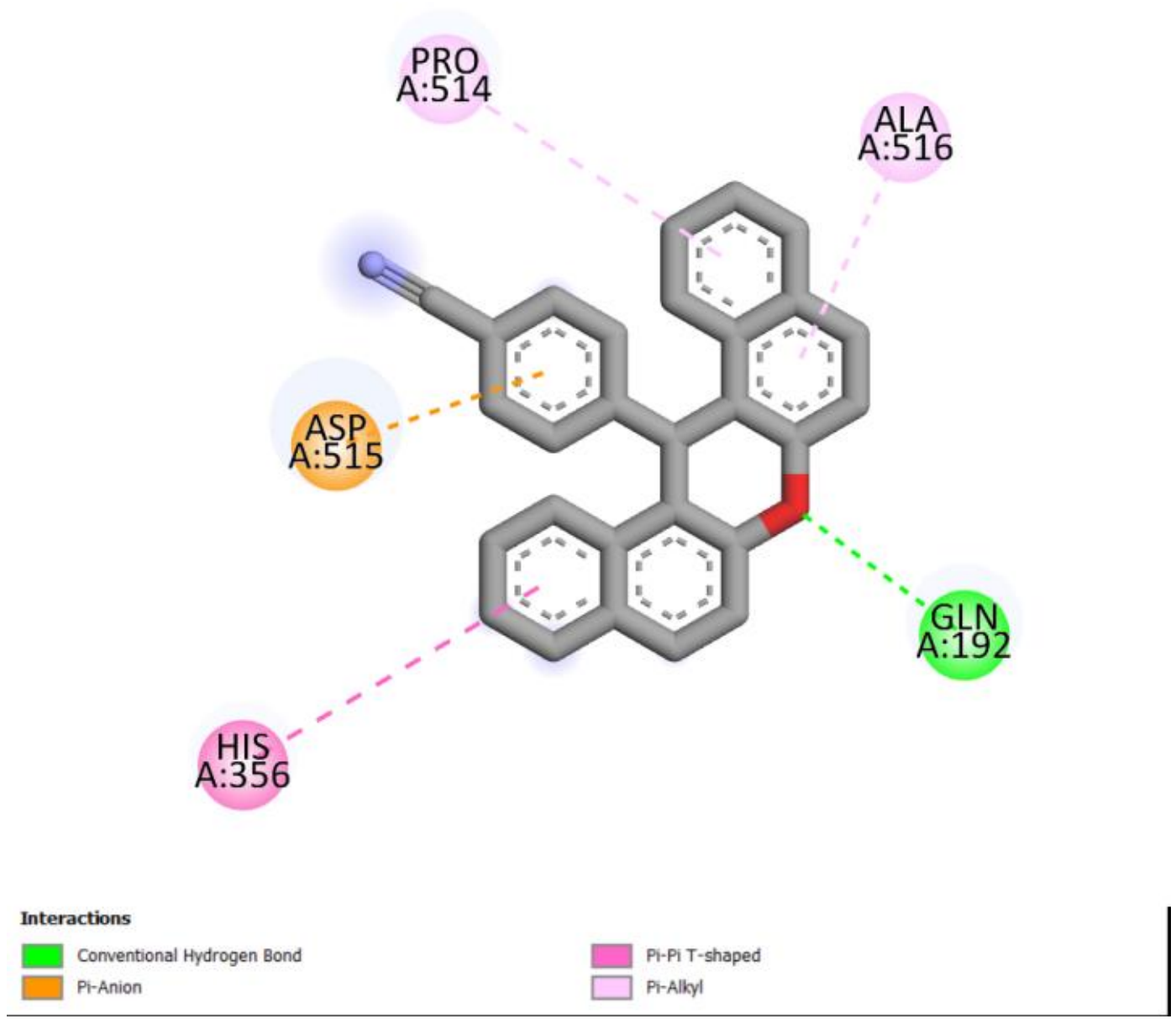

Fig. S86. Compound 6e. 2D-Interaction of ligand-protein

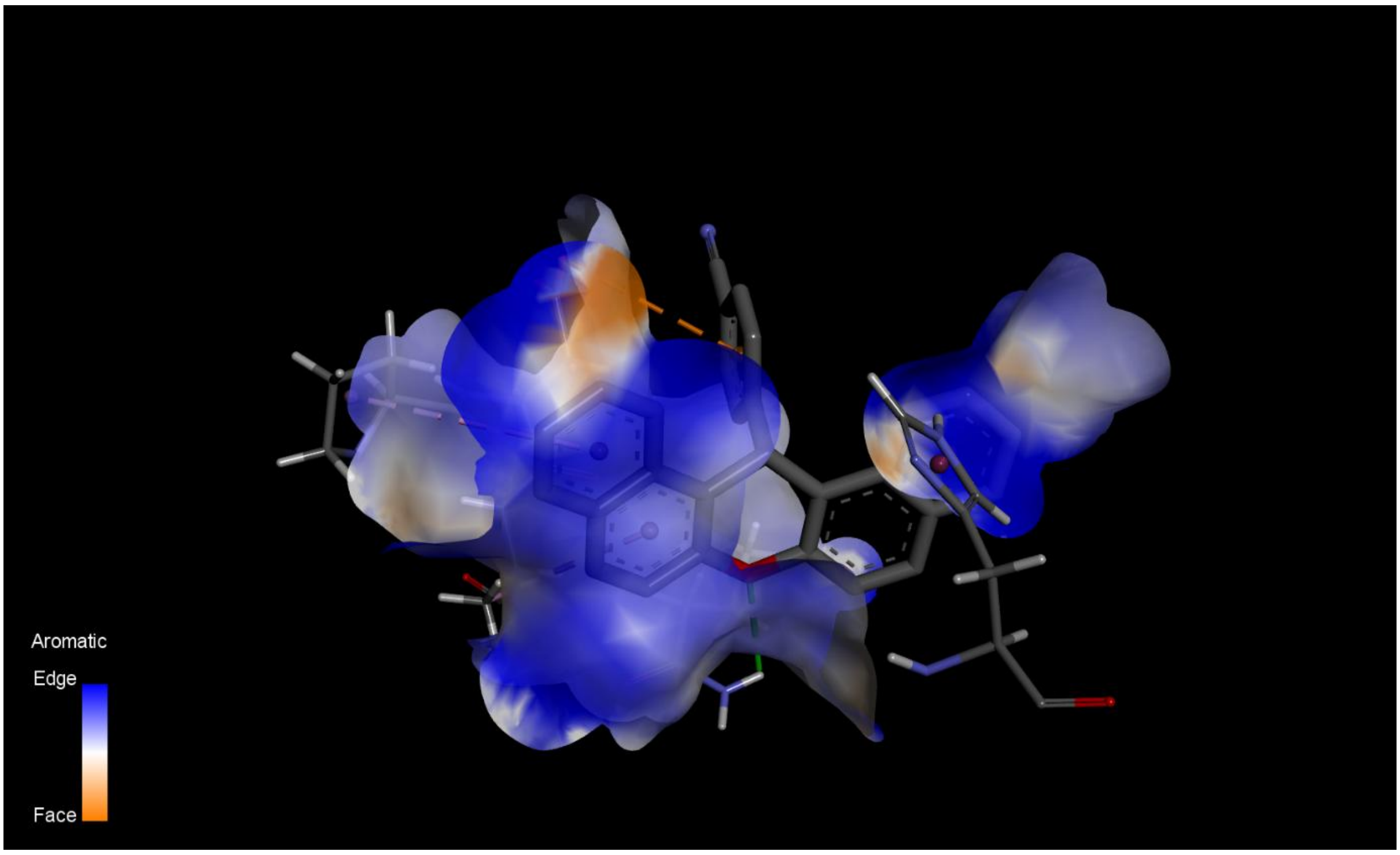

Fig. S87. Compound 6e. Visualization of aromatic face-edge interaction

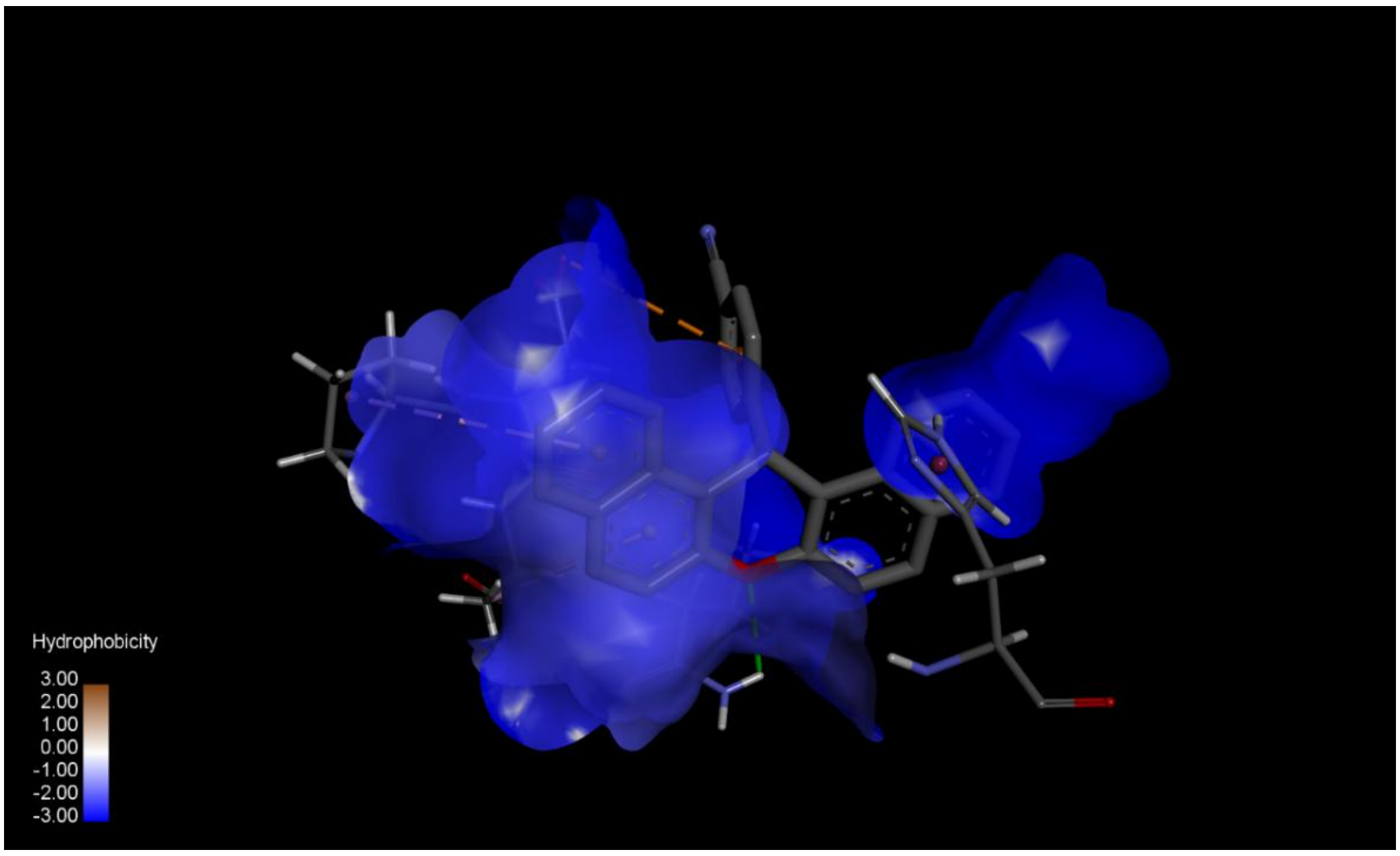

Fig. S88. Compound 6e. Visualization of hydrophobic interaction in the active site

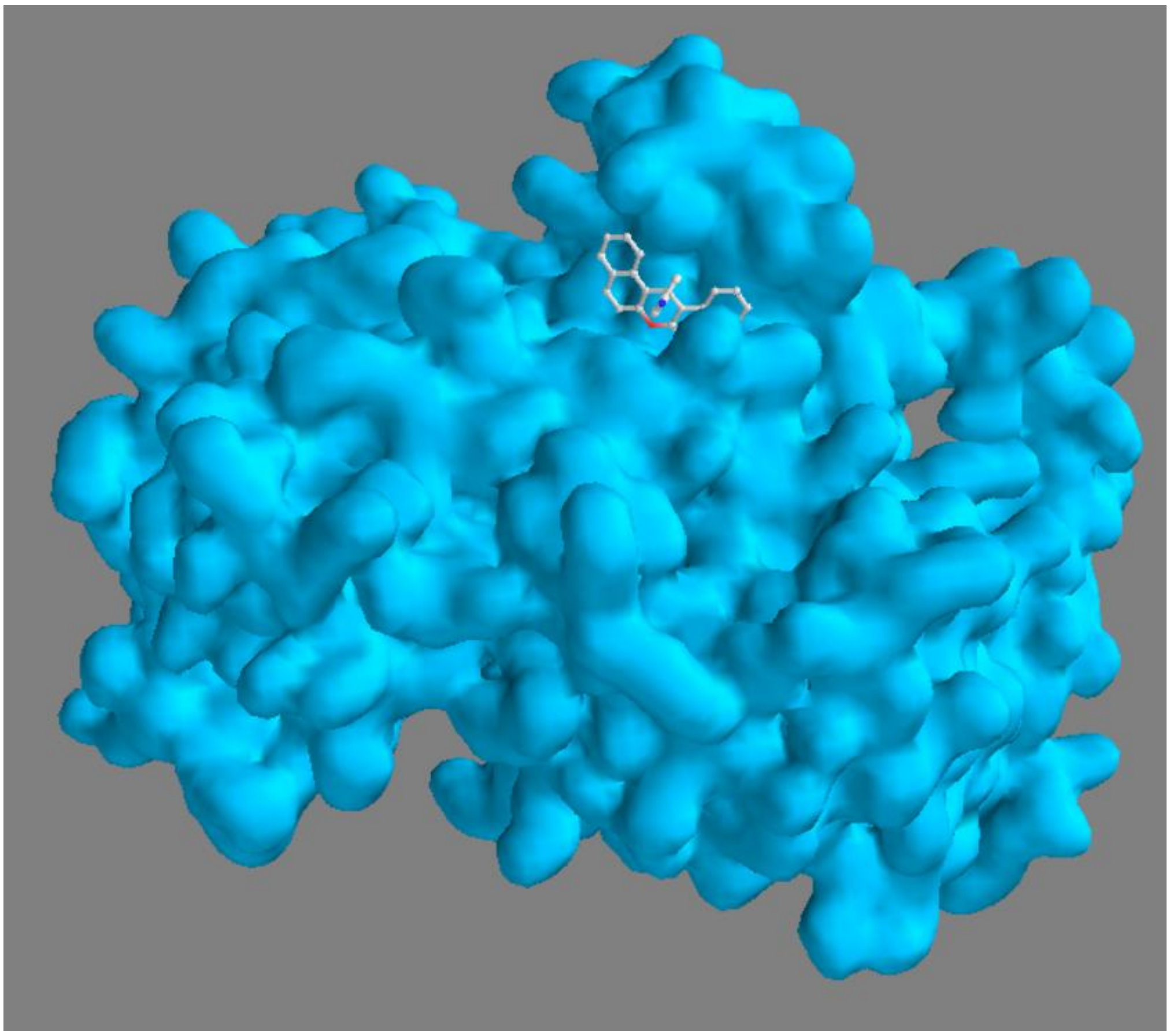

Fig. S89. Compound 6e. Protein surface visualization with bounded ligand

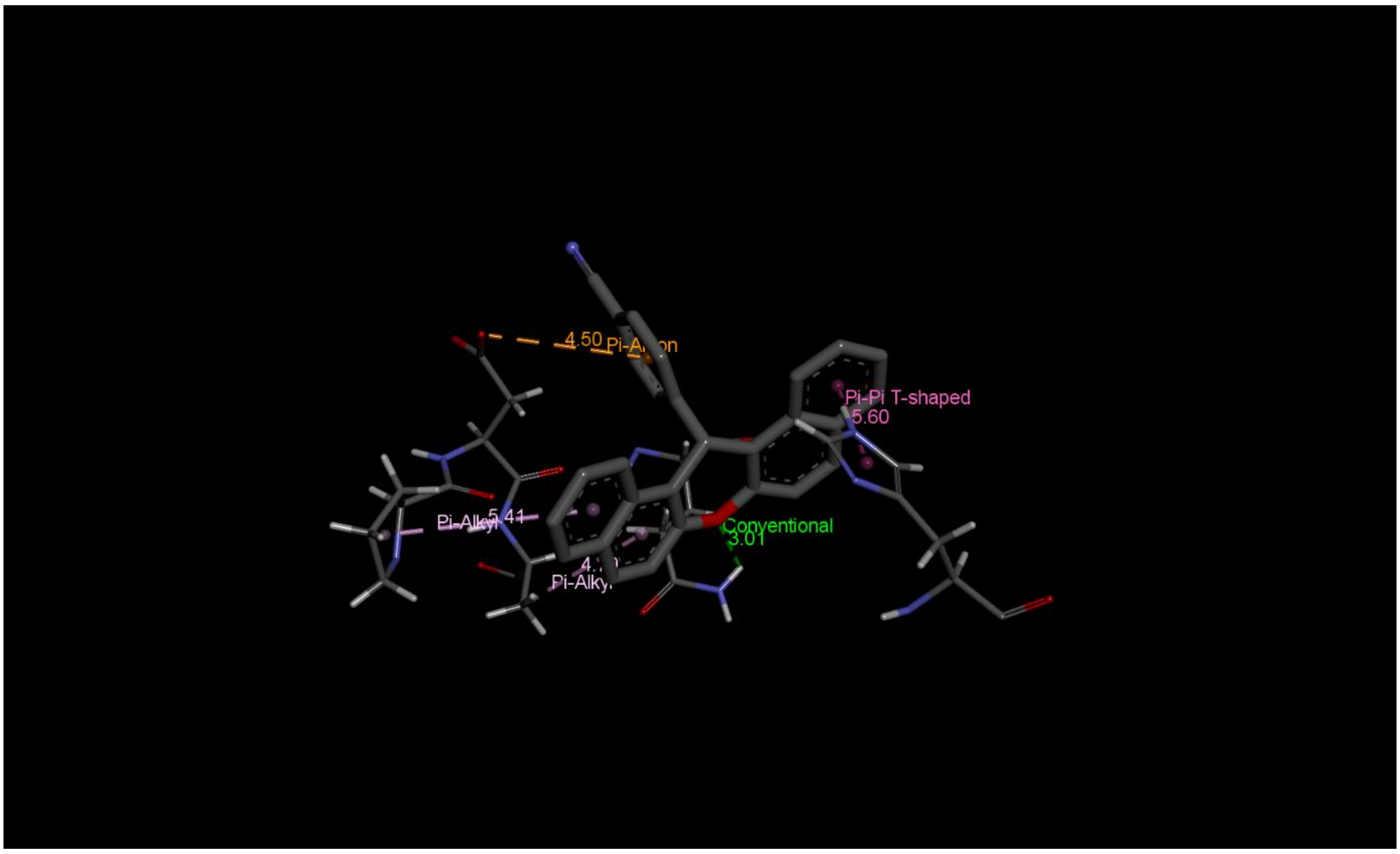

Fig. S90. Compound 6e. Binding pose of the ligand with the active site

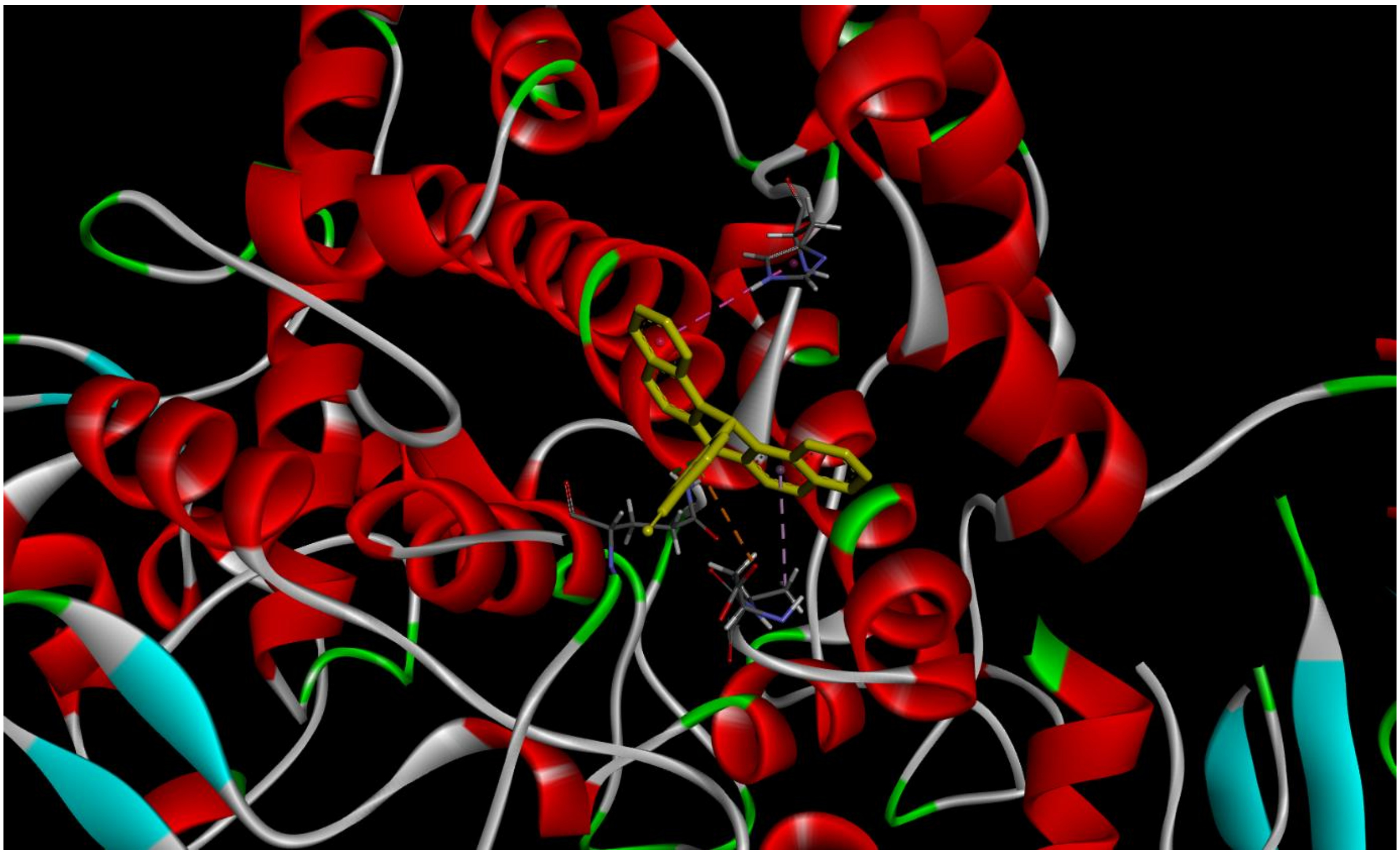

Fig. S91. Compound 6e. Ribbon diagram of the protein-ligand interaction

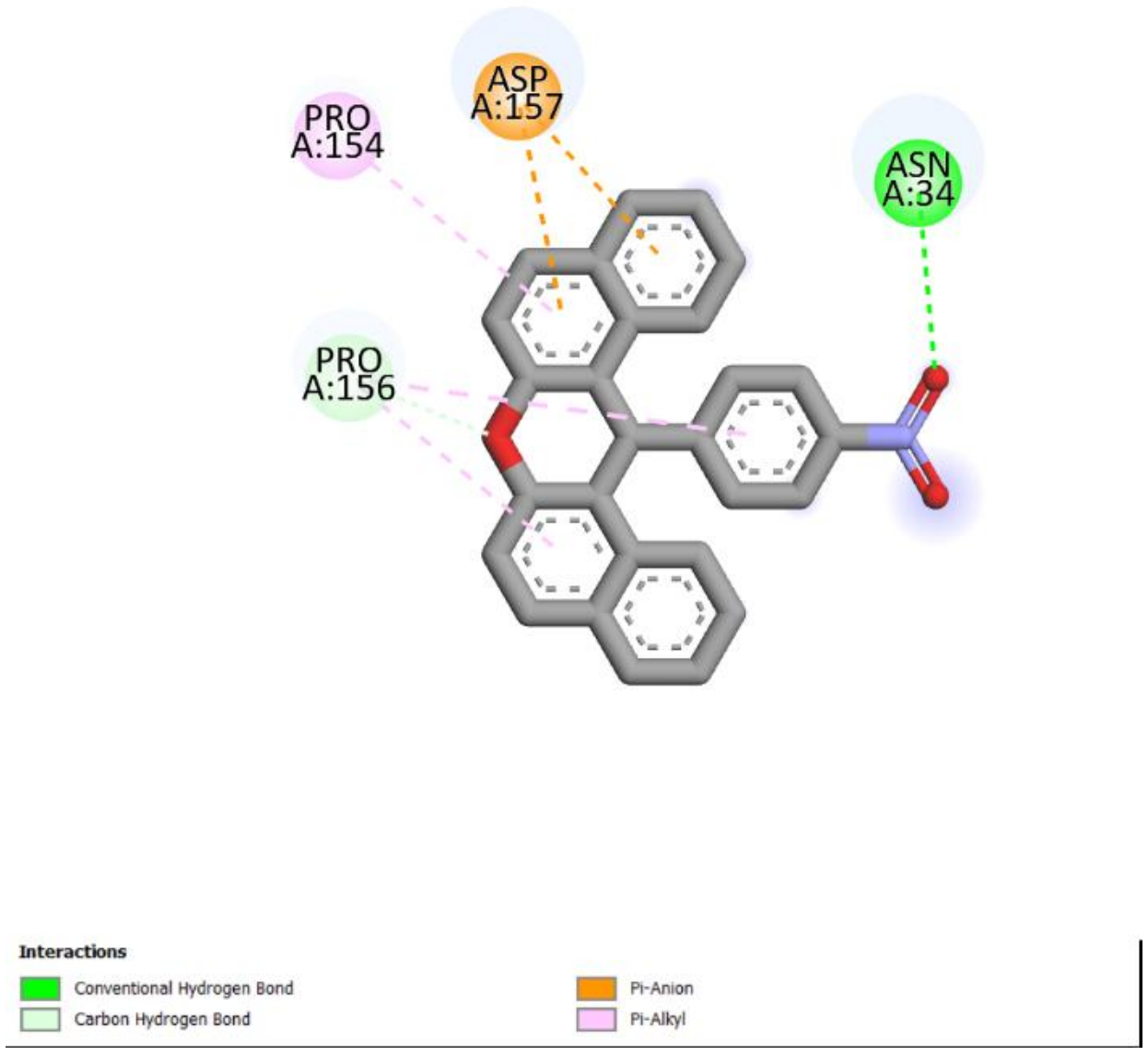

Fig. S92. Compound 6f. 2D-Interaction of ligand-protein

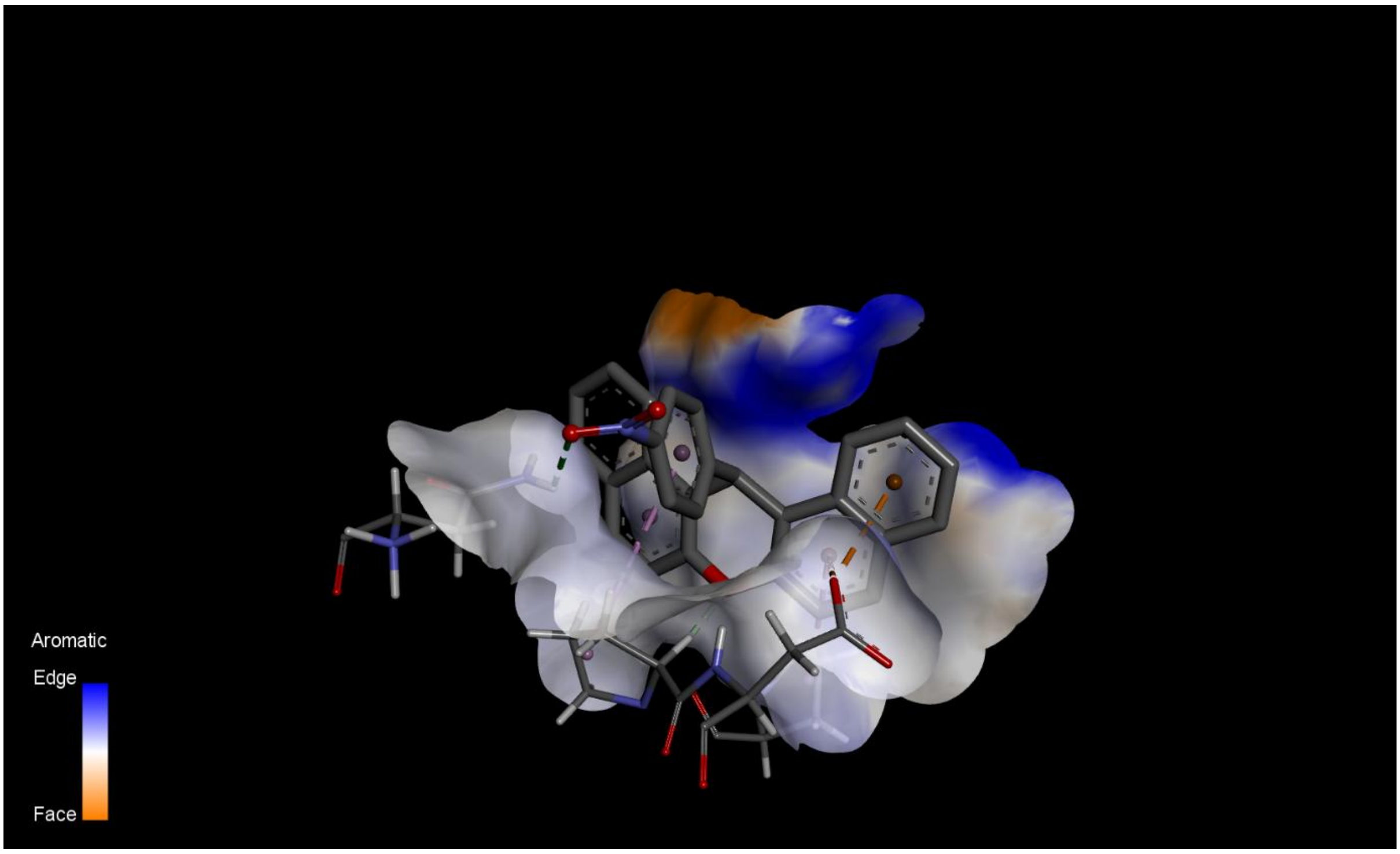

Fig. S93. Compound 6f. Visualization of aromatic face-edge interaction

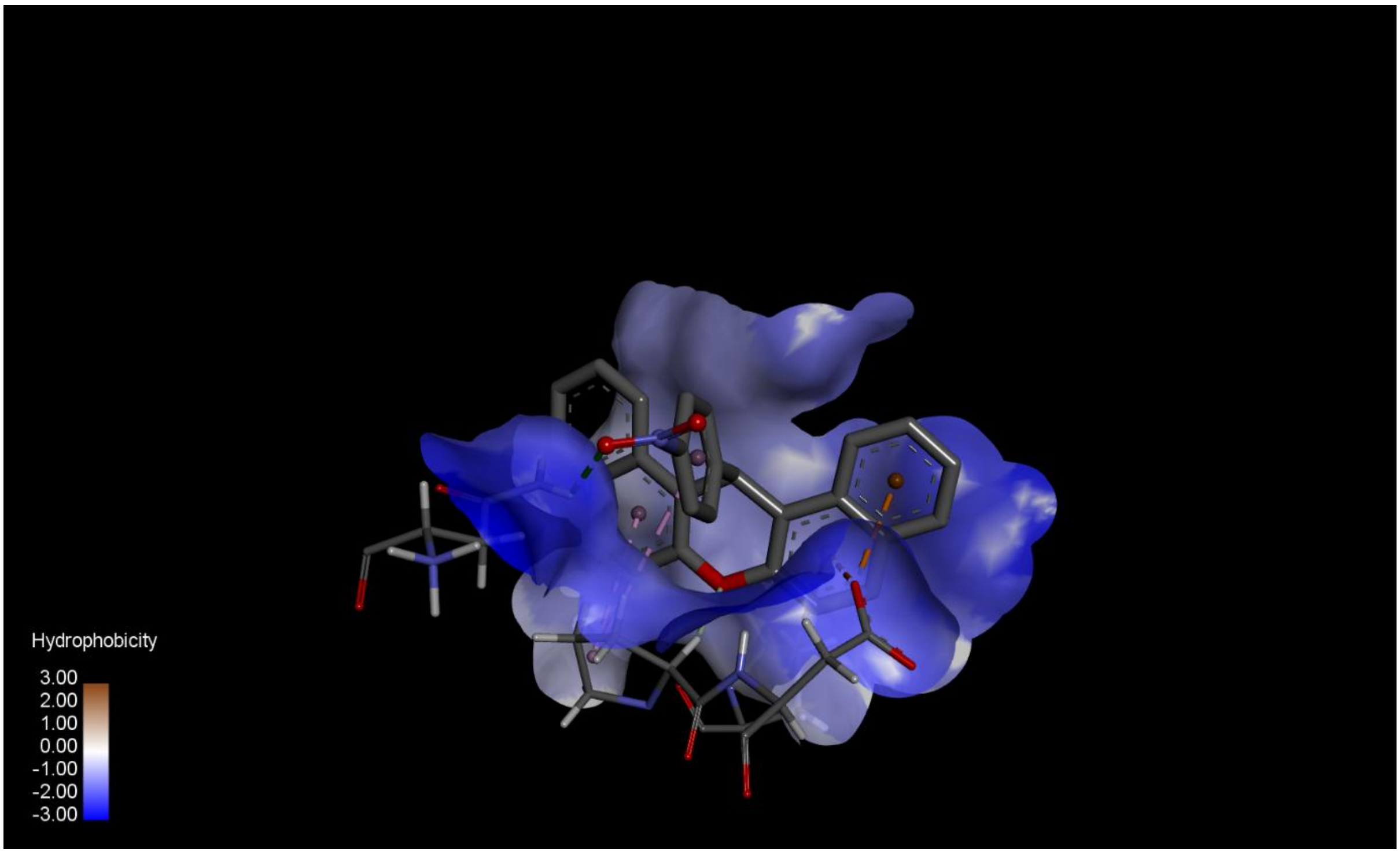

Fig. S94. Compound 6f. Visualization of hydrophobic interaction in the active site

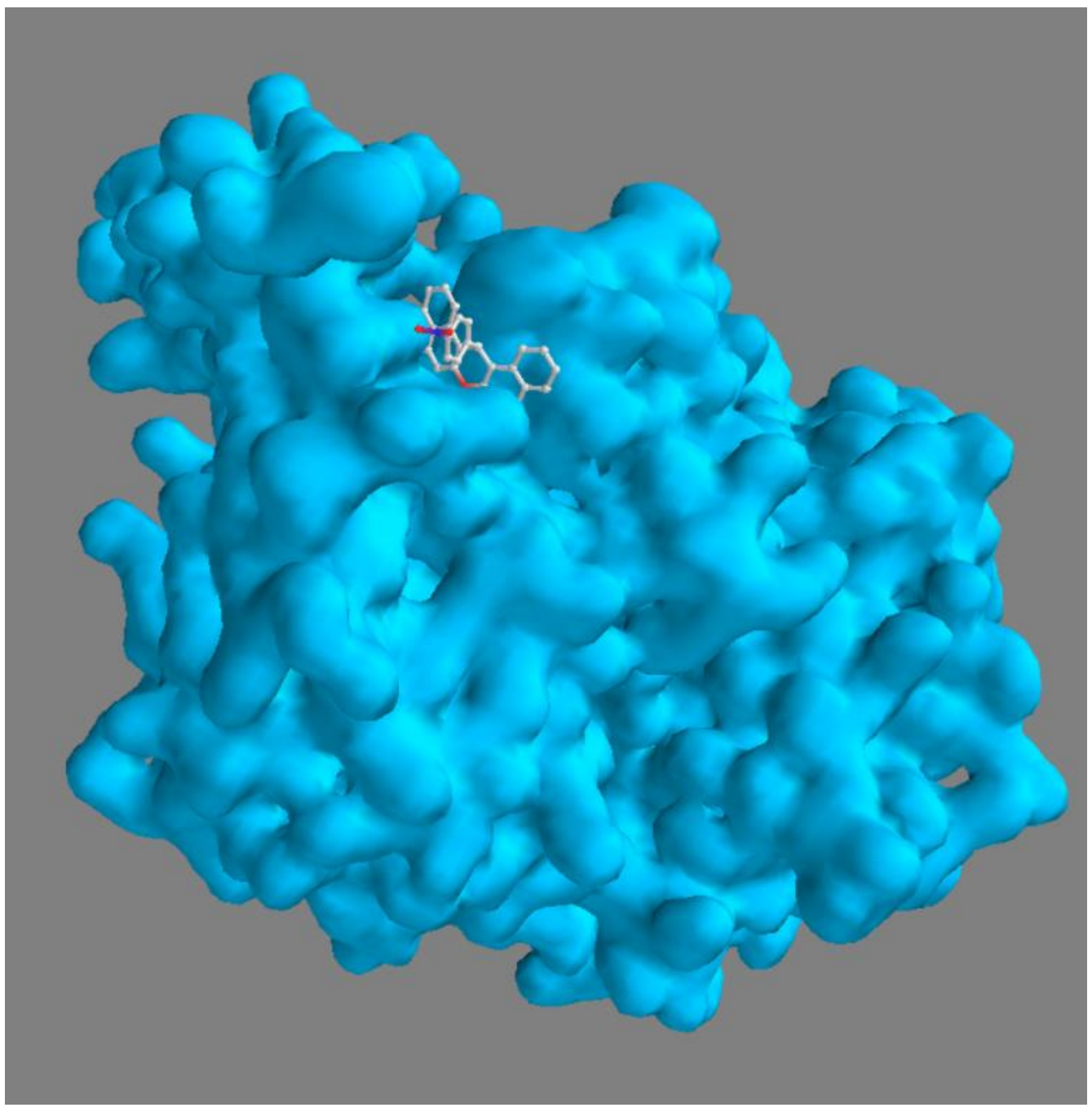

Fig. S95: Compound 6f. Protein surface visualization with bounded ligand

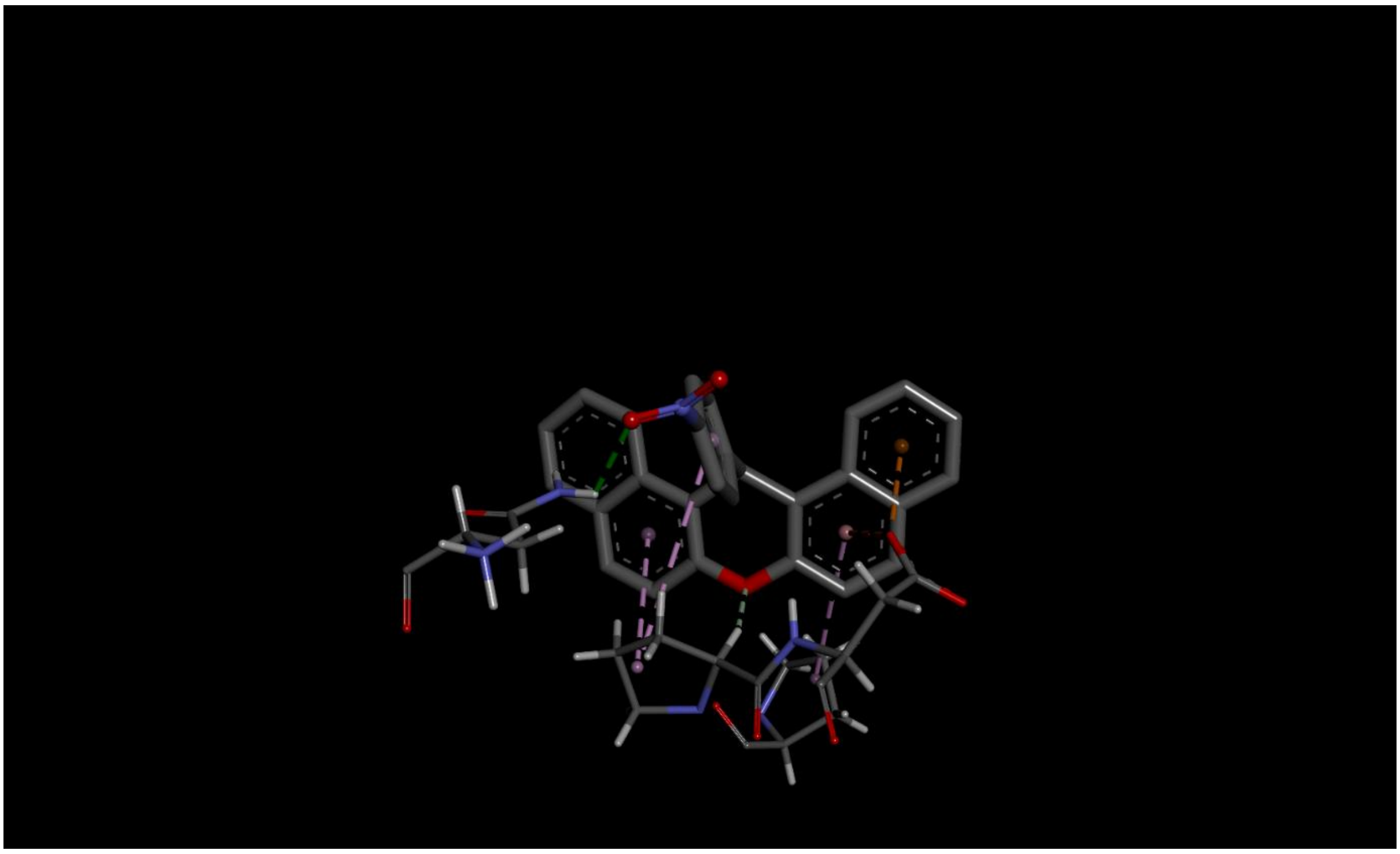

Fig. S96. Compound 6f. Binding pose of the ligand with the active site

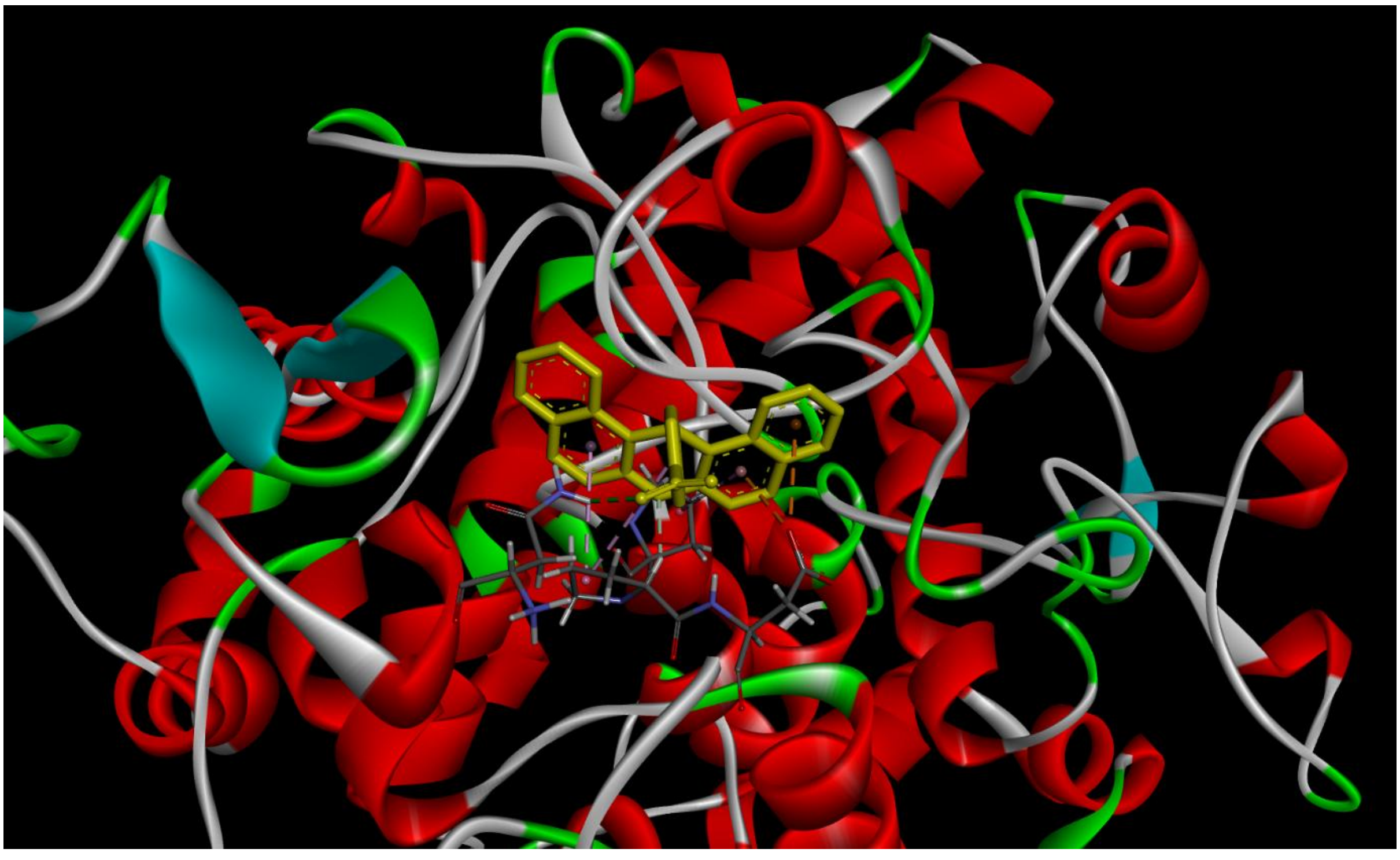

Fig. S97. Compound 6f. Ribbon diagram of the protein-ligand interaction

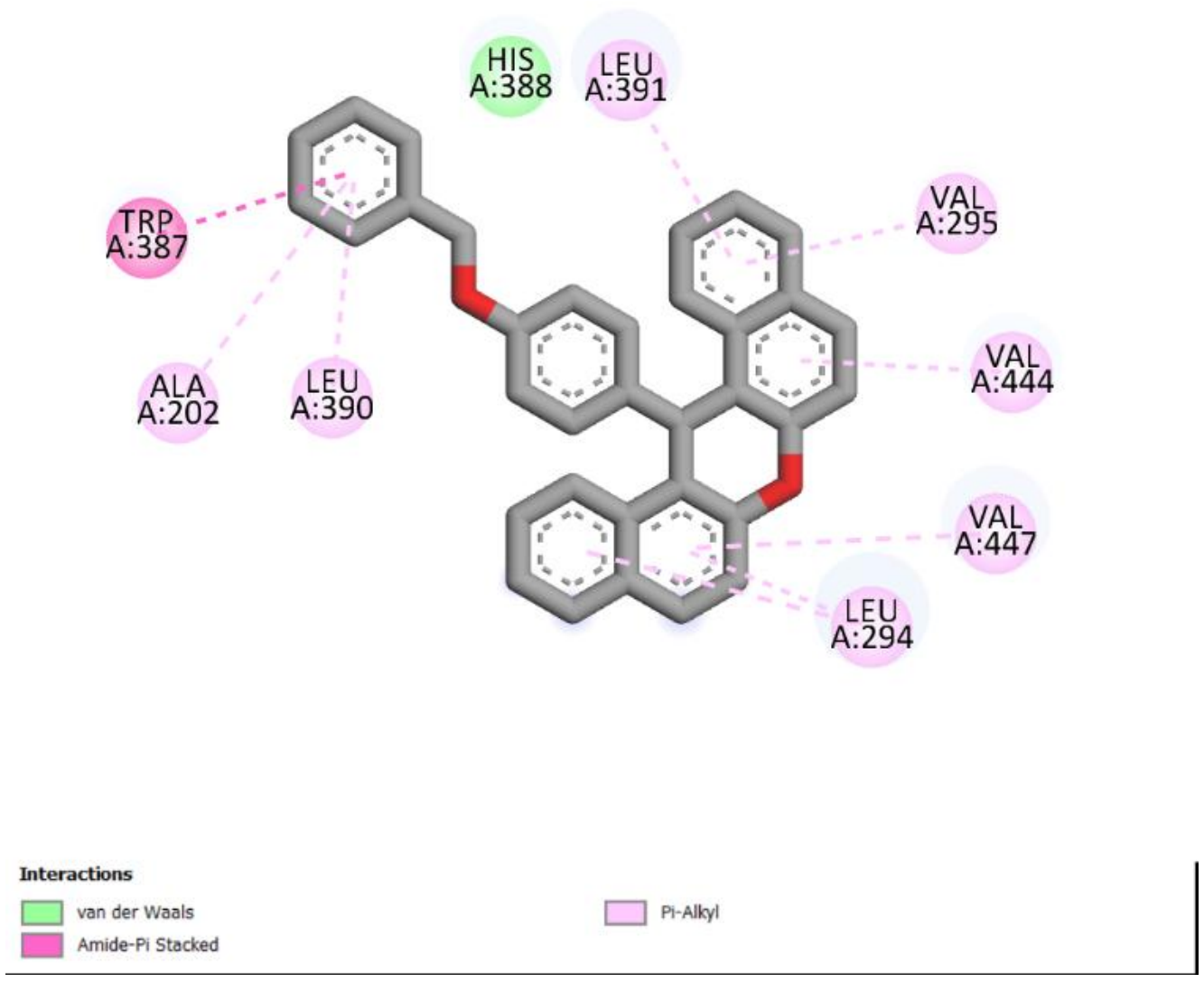

Fig. S98. Compound 6g.2D-Interaction of ligand-protein

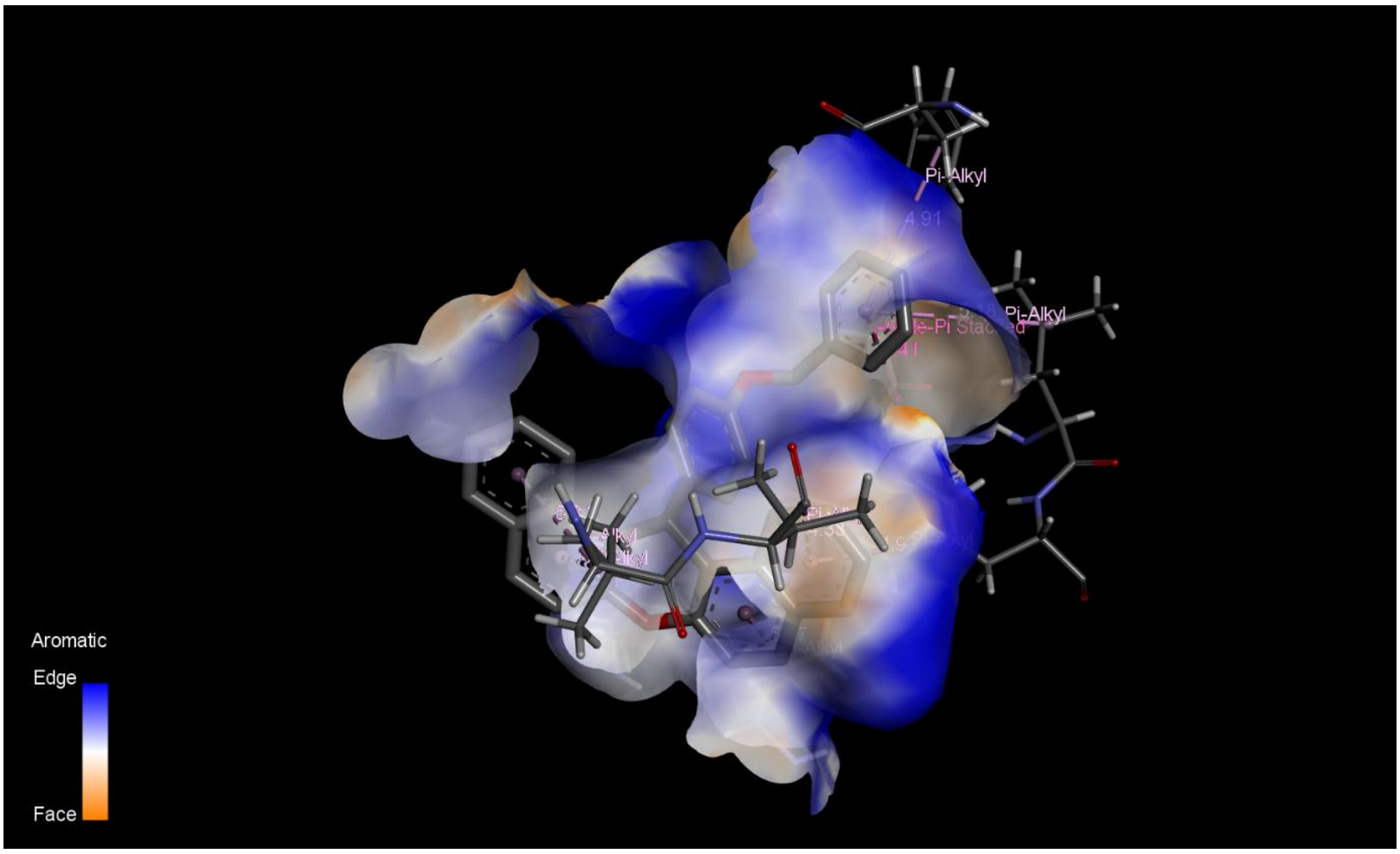

Fig. S99. Compound 6g. Visualization of aromatic face-edge interaction

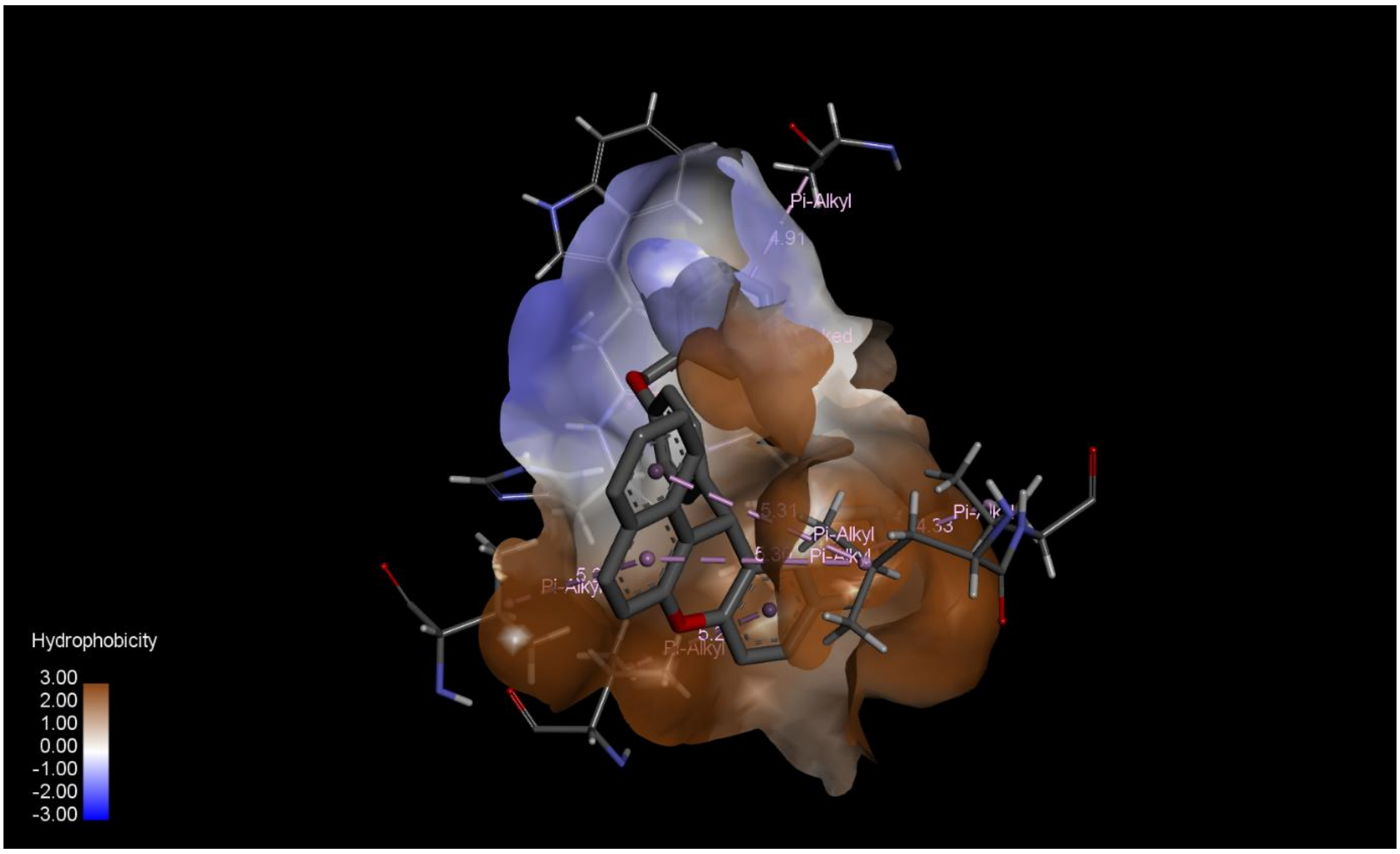

Fig. S100. Compound 6g. Visualization of hydrophobic interaction in the active site

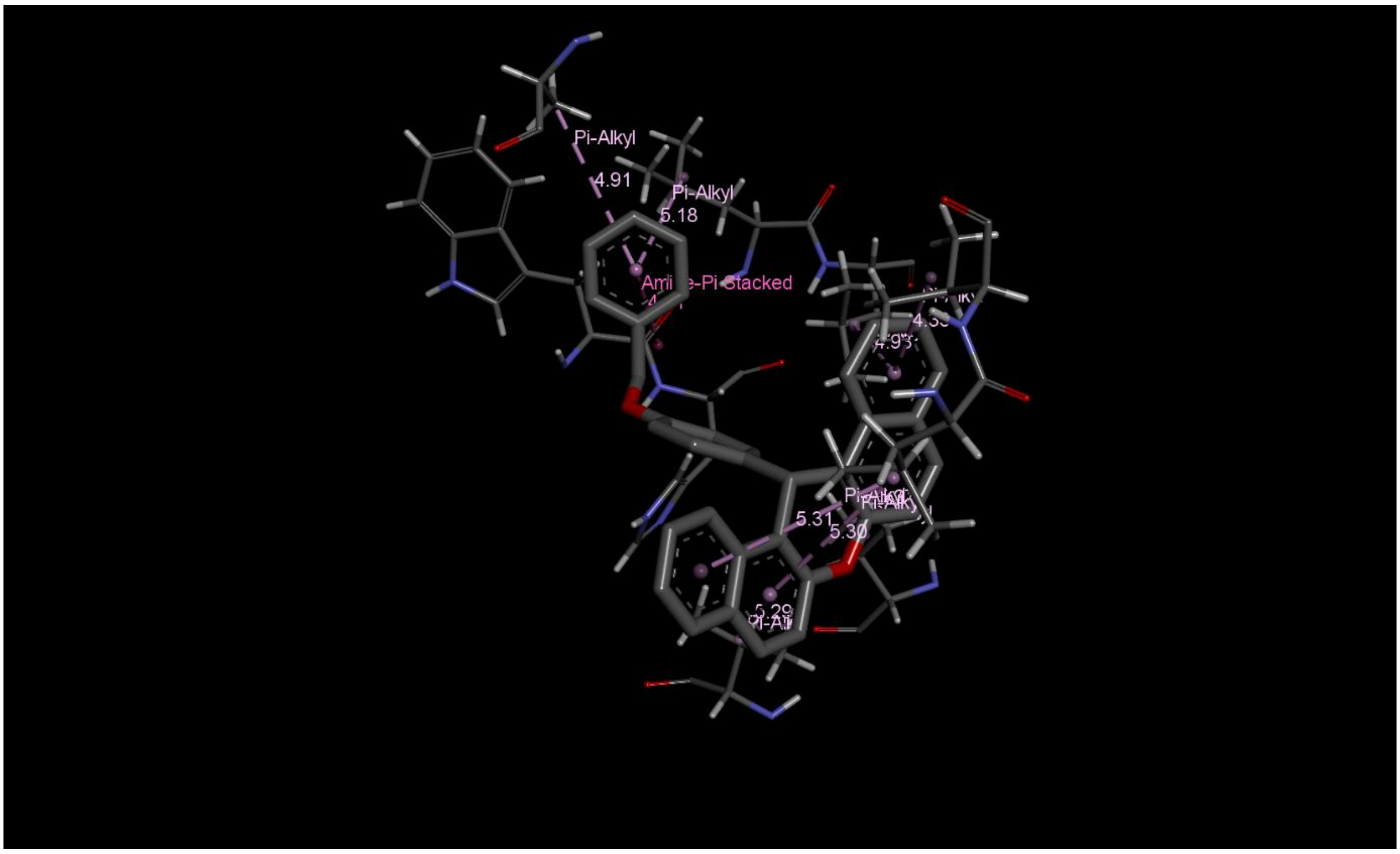

Fig. S101. Compound 6g. Binding pose of the ligand with the active site

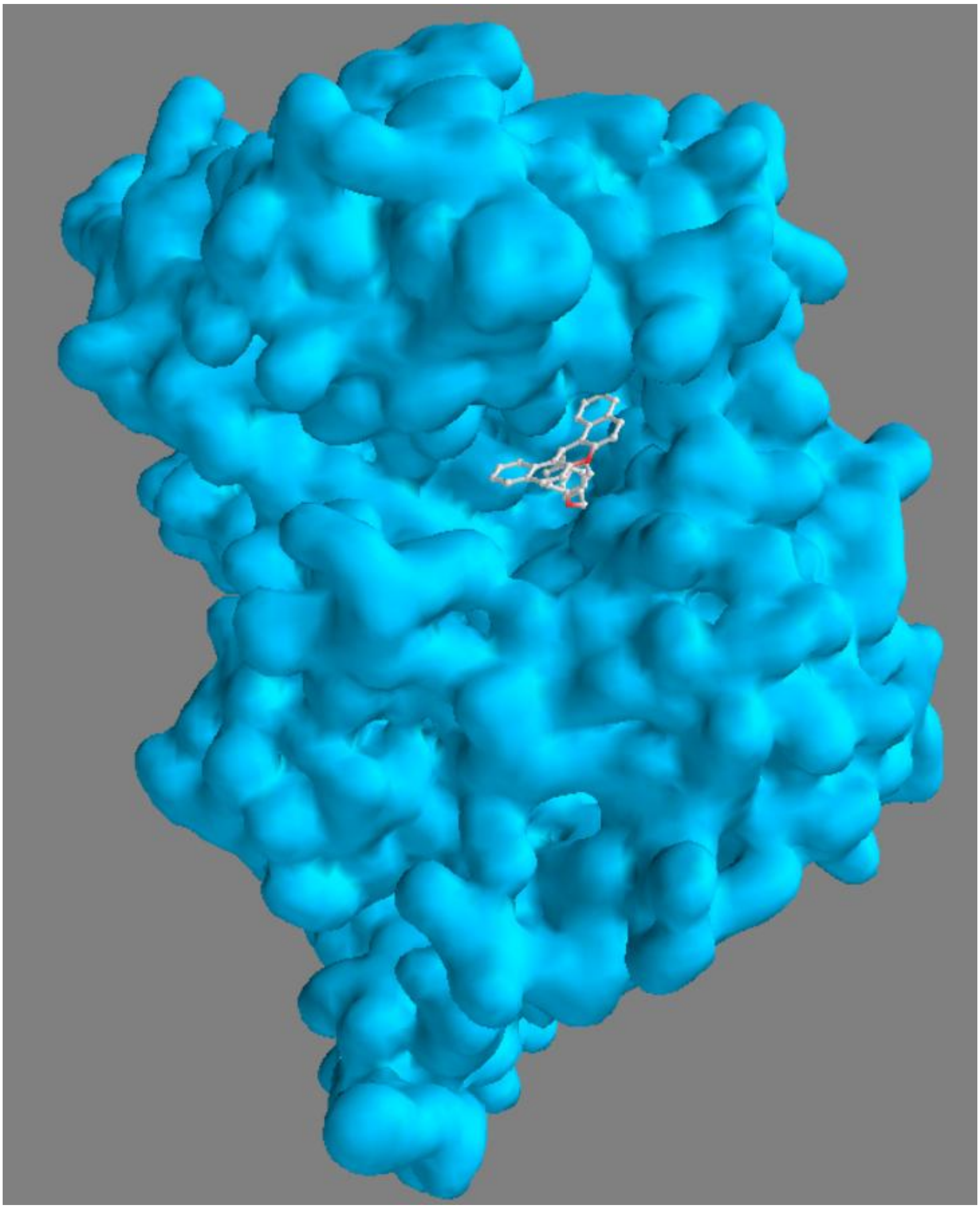

Fig. S102. Compound 6g. Protein surface visualization with bounded ligand

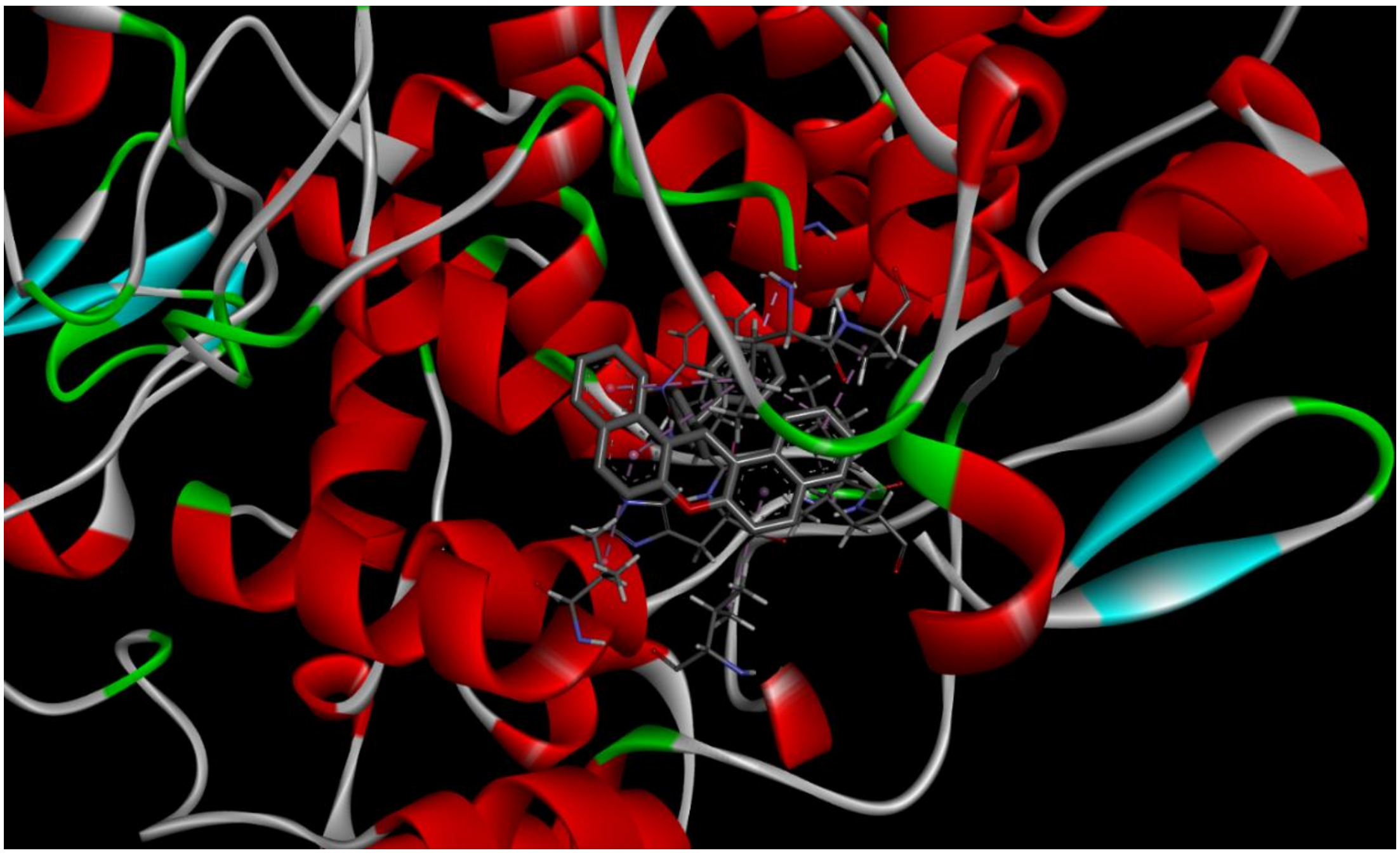

Fig. S103. Compound 6g. Ribbon diagram of the protein-ligand interaction

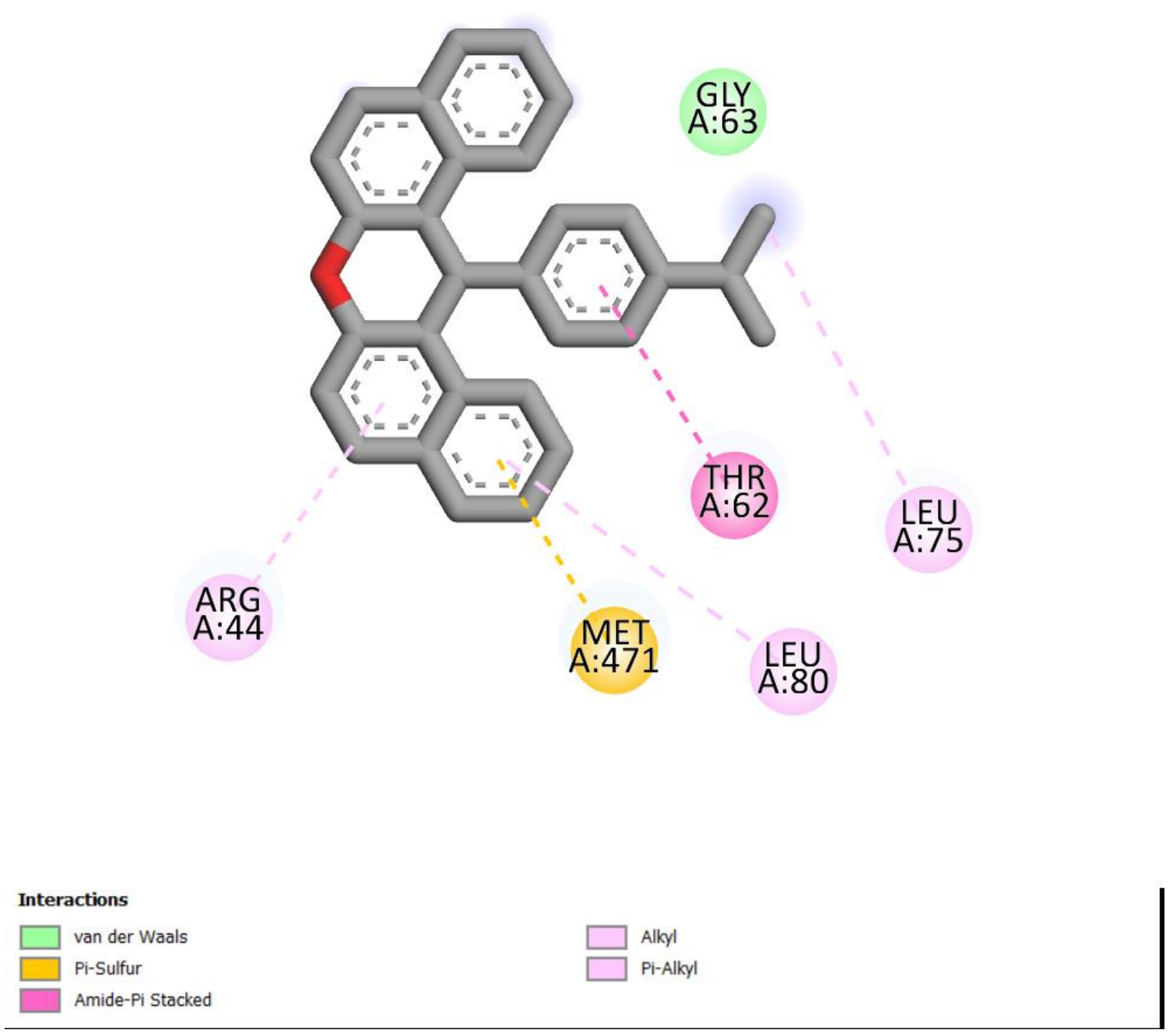

Fig. S104.Compound 6h. 2D-Interaction of ligand-protein

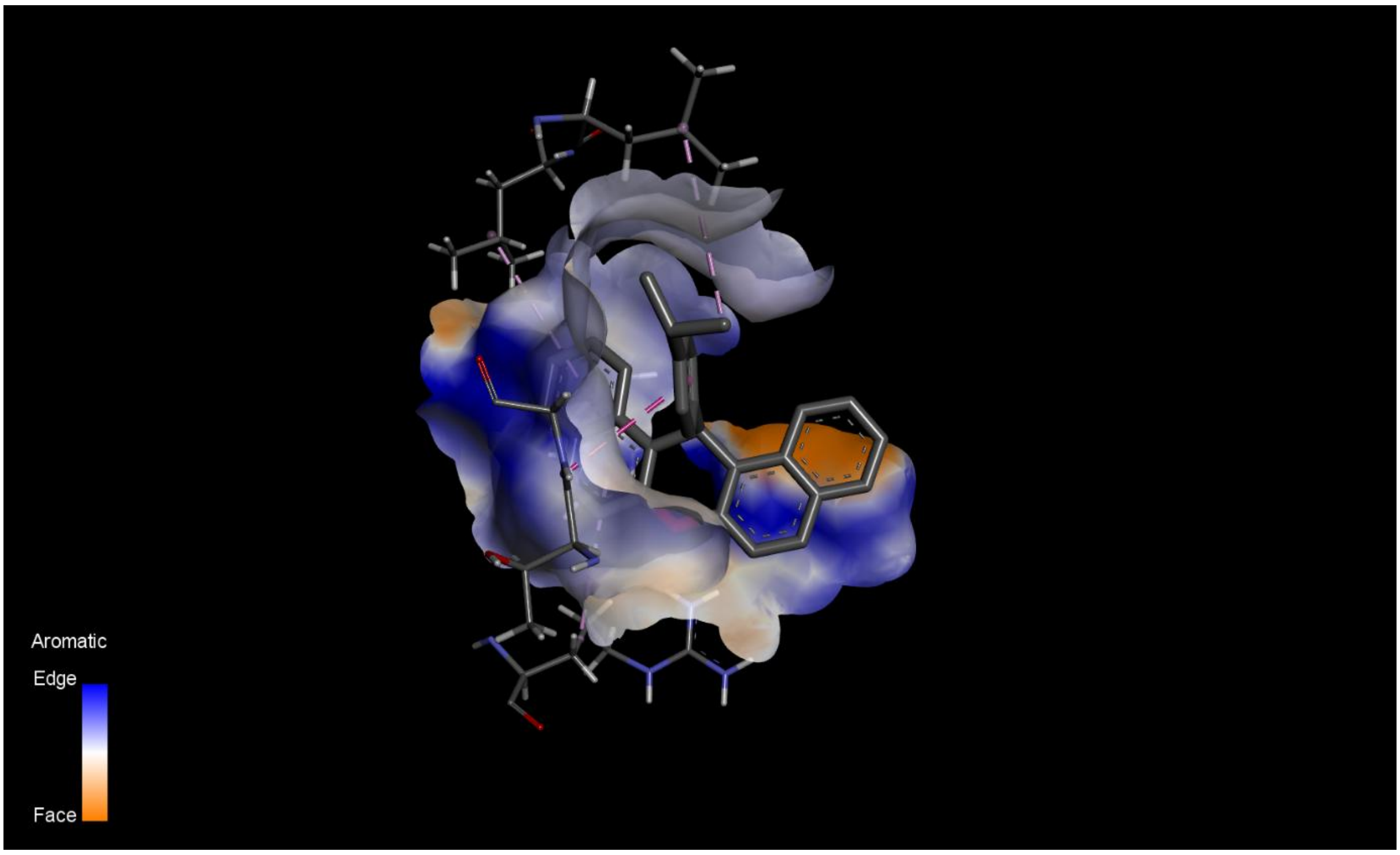

Fig. S105. Compound 6h. Visualization of aromatic face-edge interaction

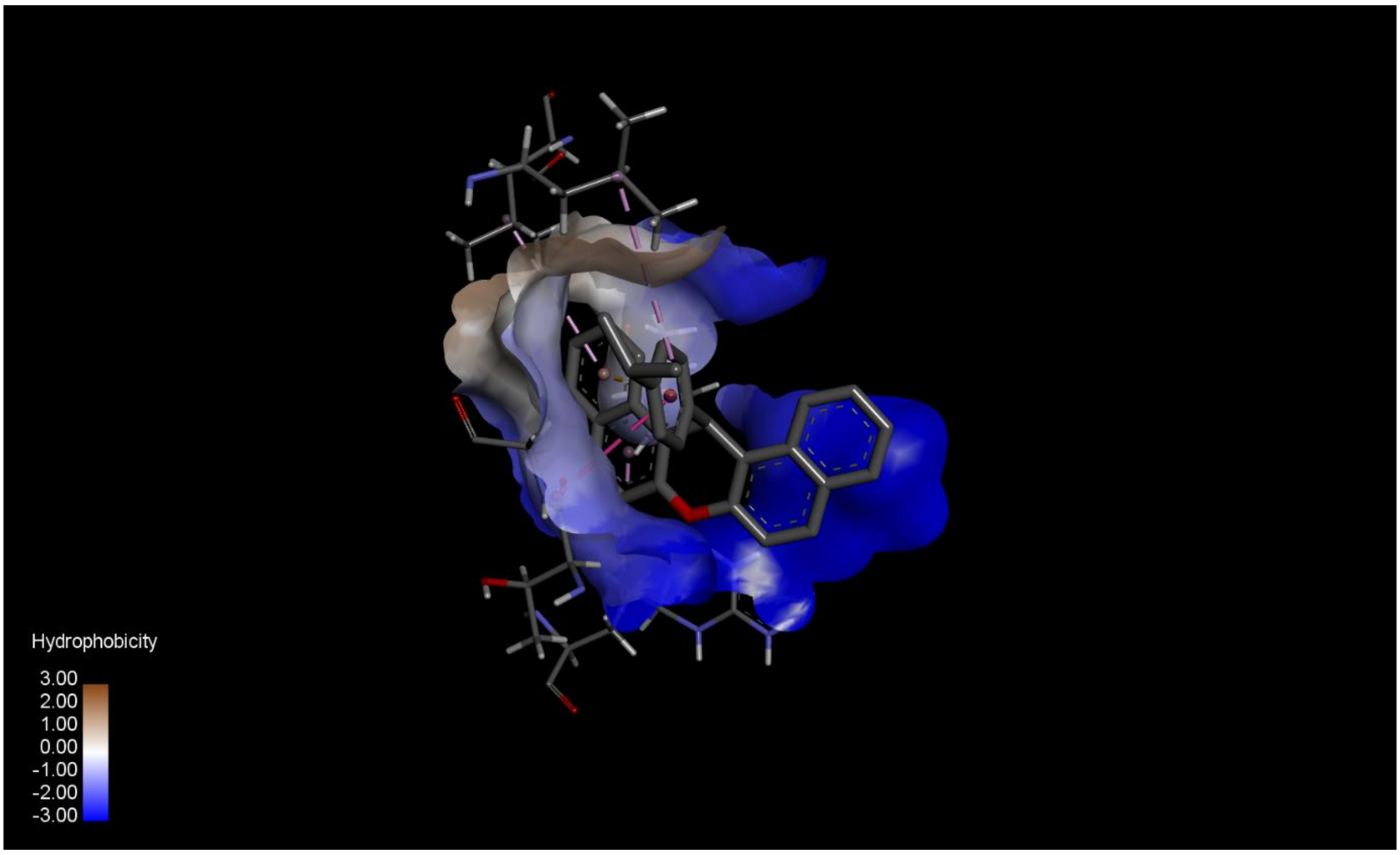

Fig. S106. Compound 6h. Visualization of hydrophobic interaction in the active site

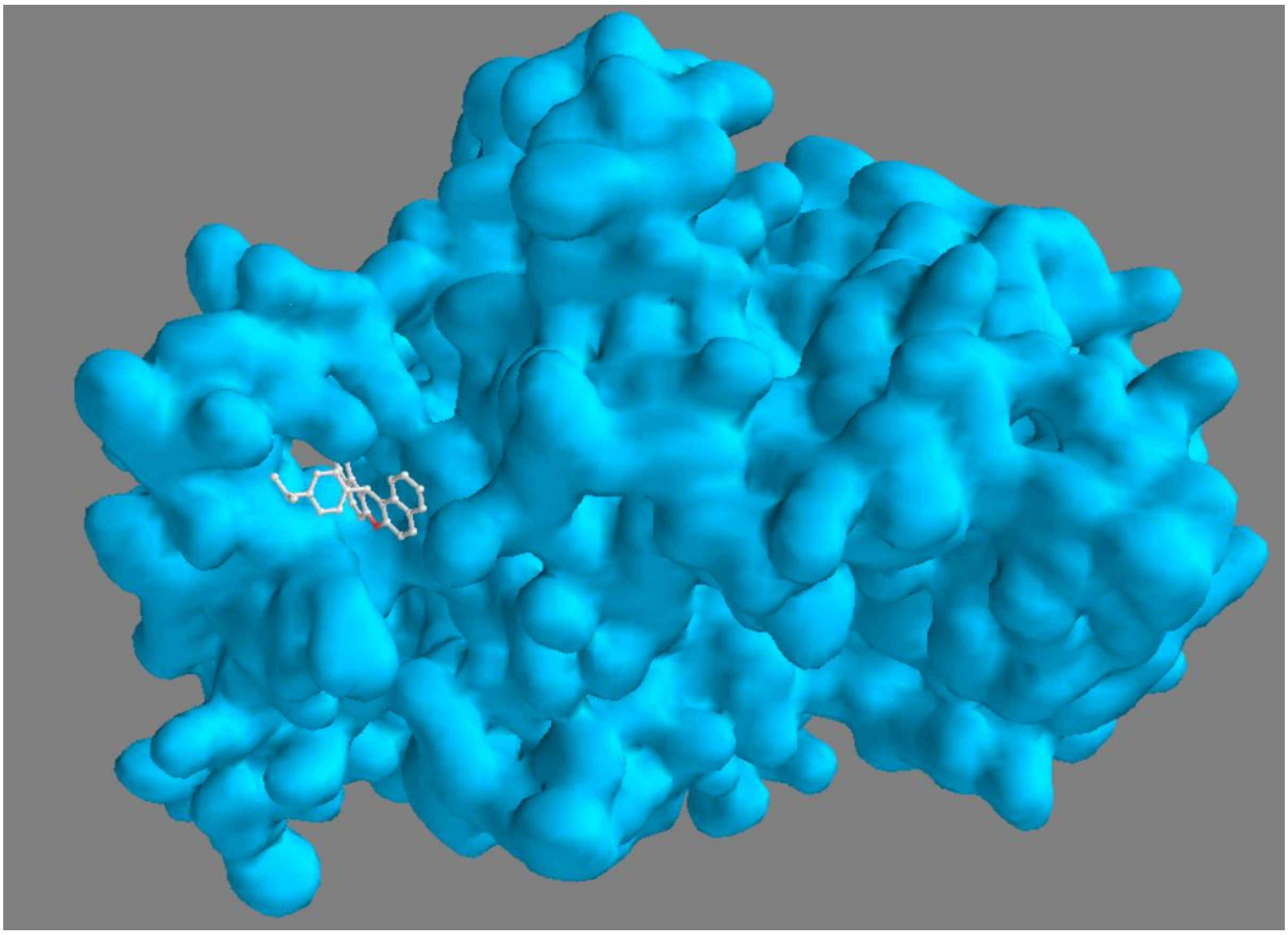

Fig. S107. Compound 6h. Protein surface visualization with bounded ligand

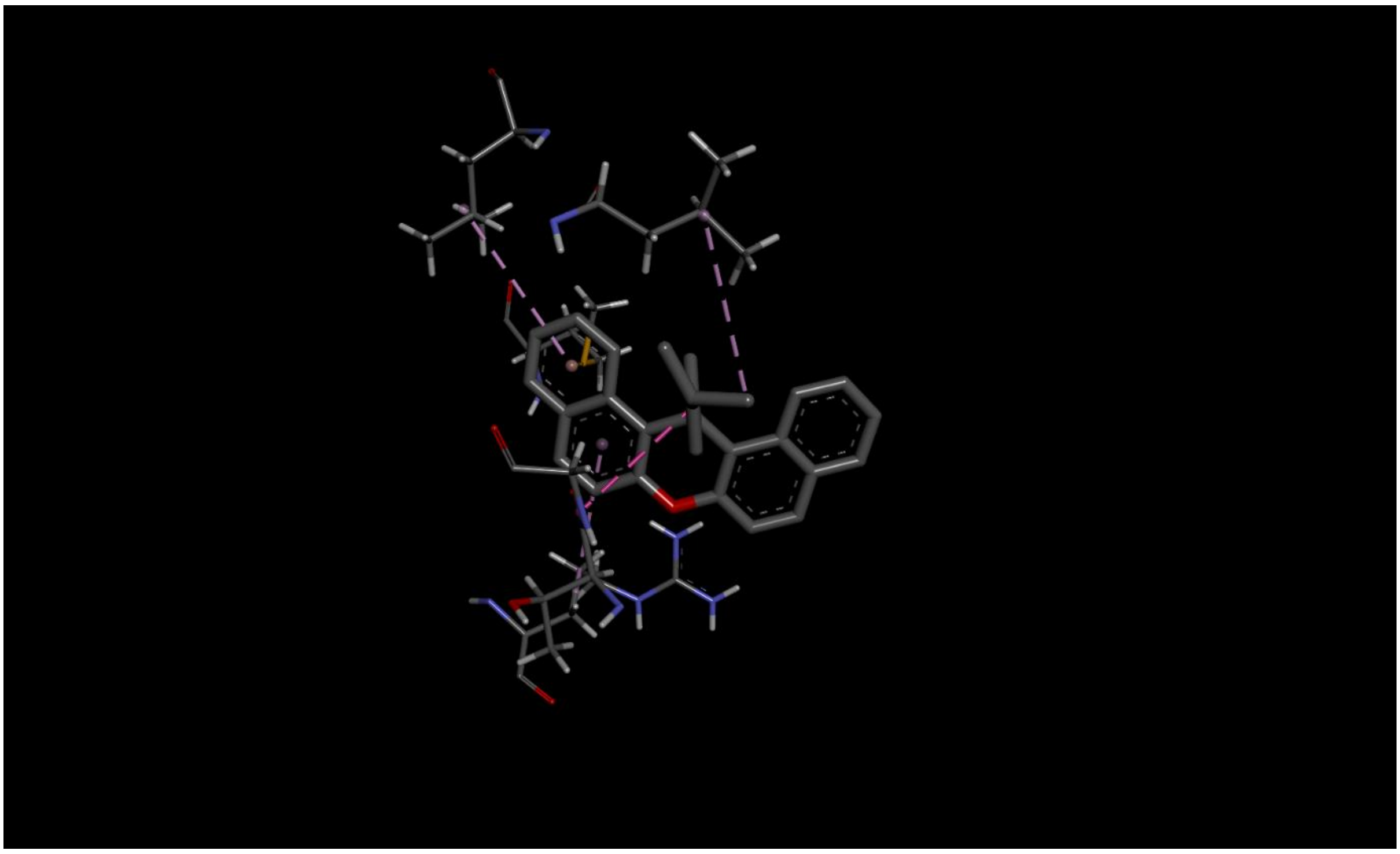

Fig. S108. Compound 6h. Binding pose of the ligand with the active site

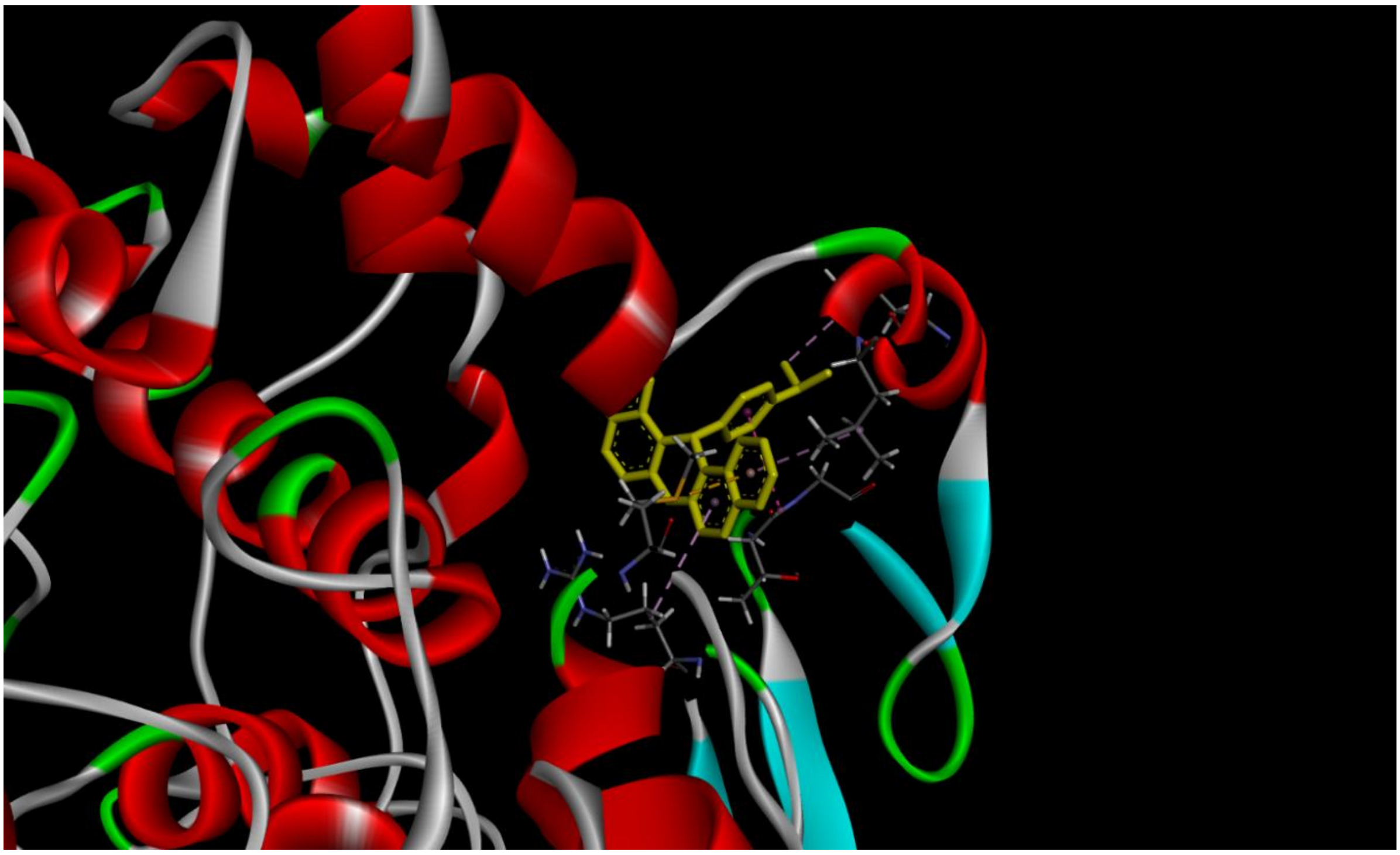

Fig. S109. Compound 6h. Ribbon diagram of the protein-ligand interaction

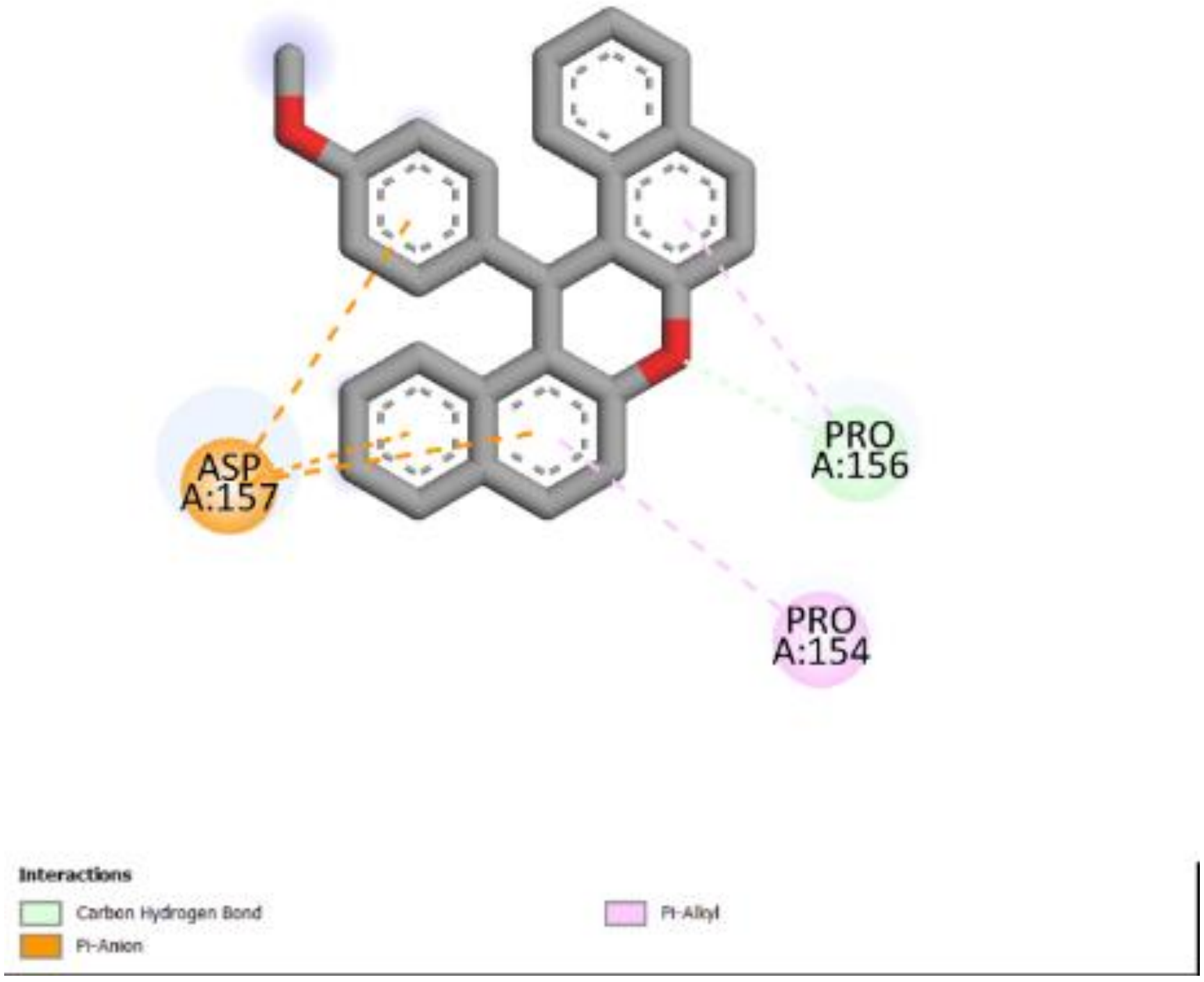

Fig. S110. Compound 6i. 2D-Interaction of ligand-protein

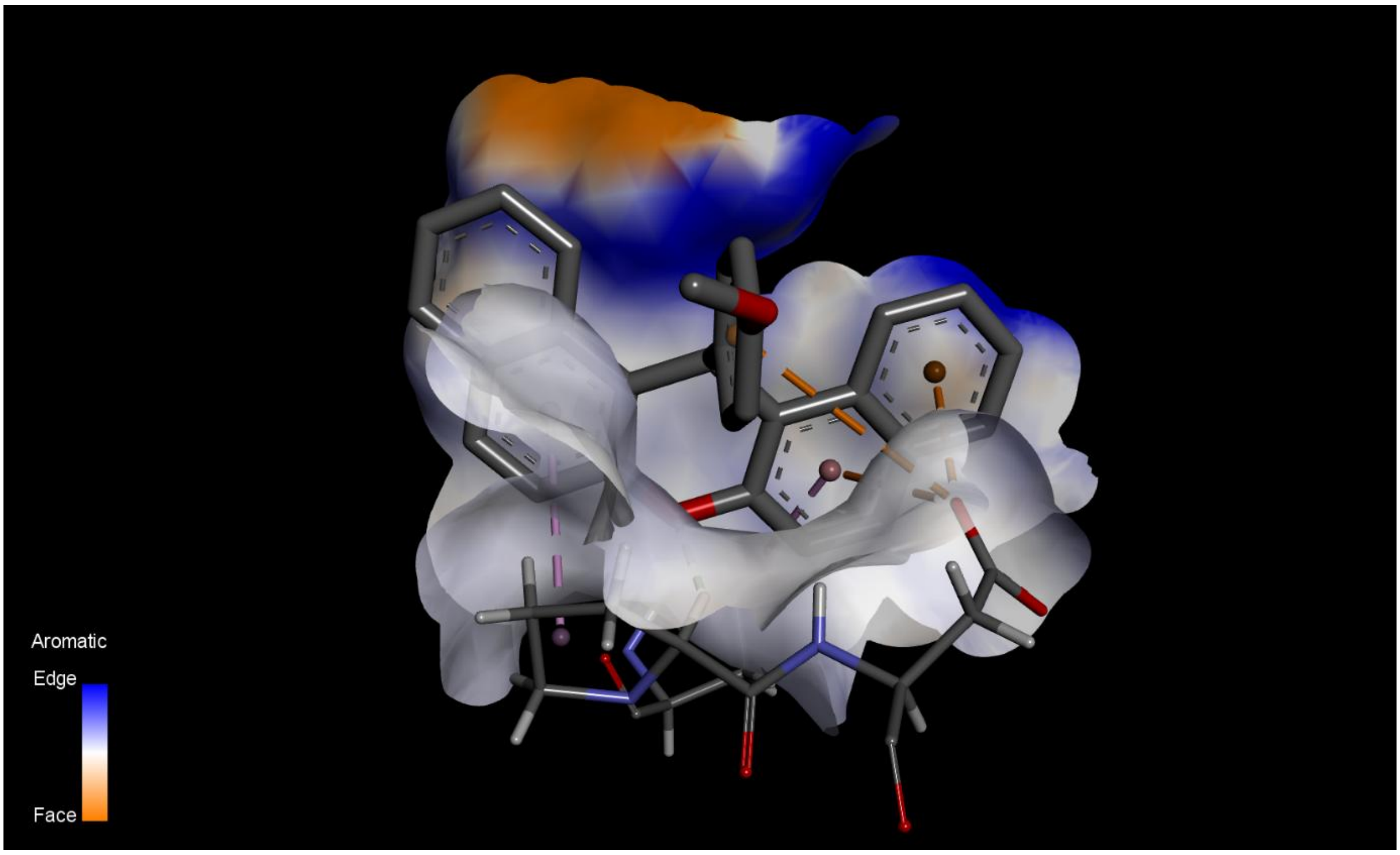

Fig. S111. Compound 6i. Visualization of aromatic face-edge interaction

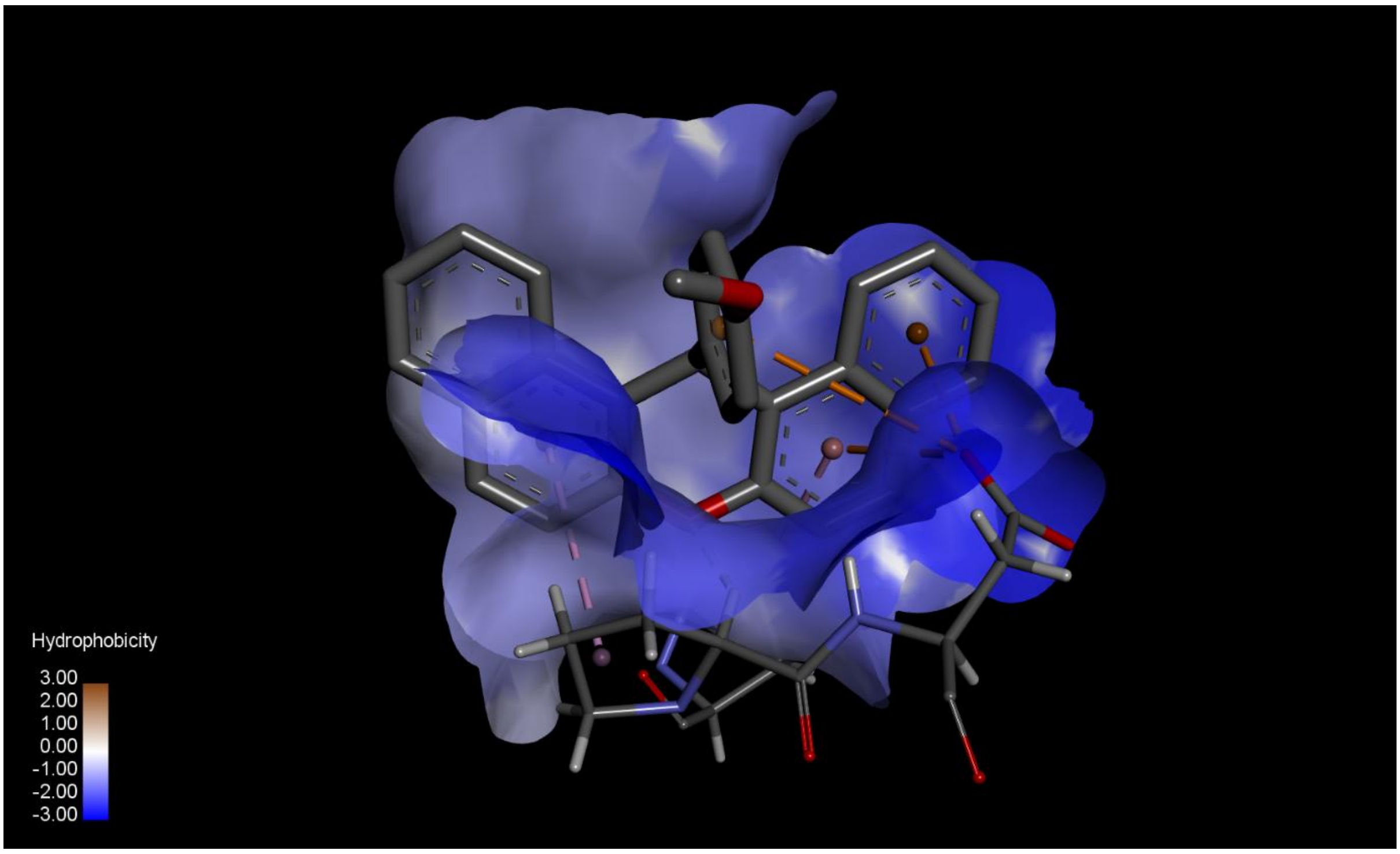

Fig. S112: Compound 6i. Visualization of hydrophobic interaction in the active site

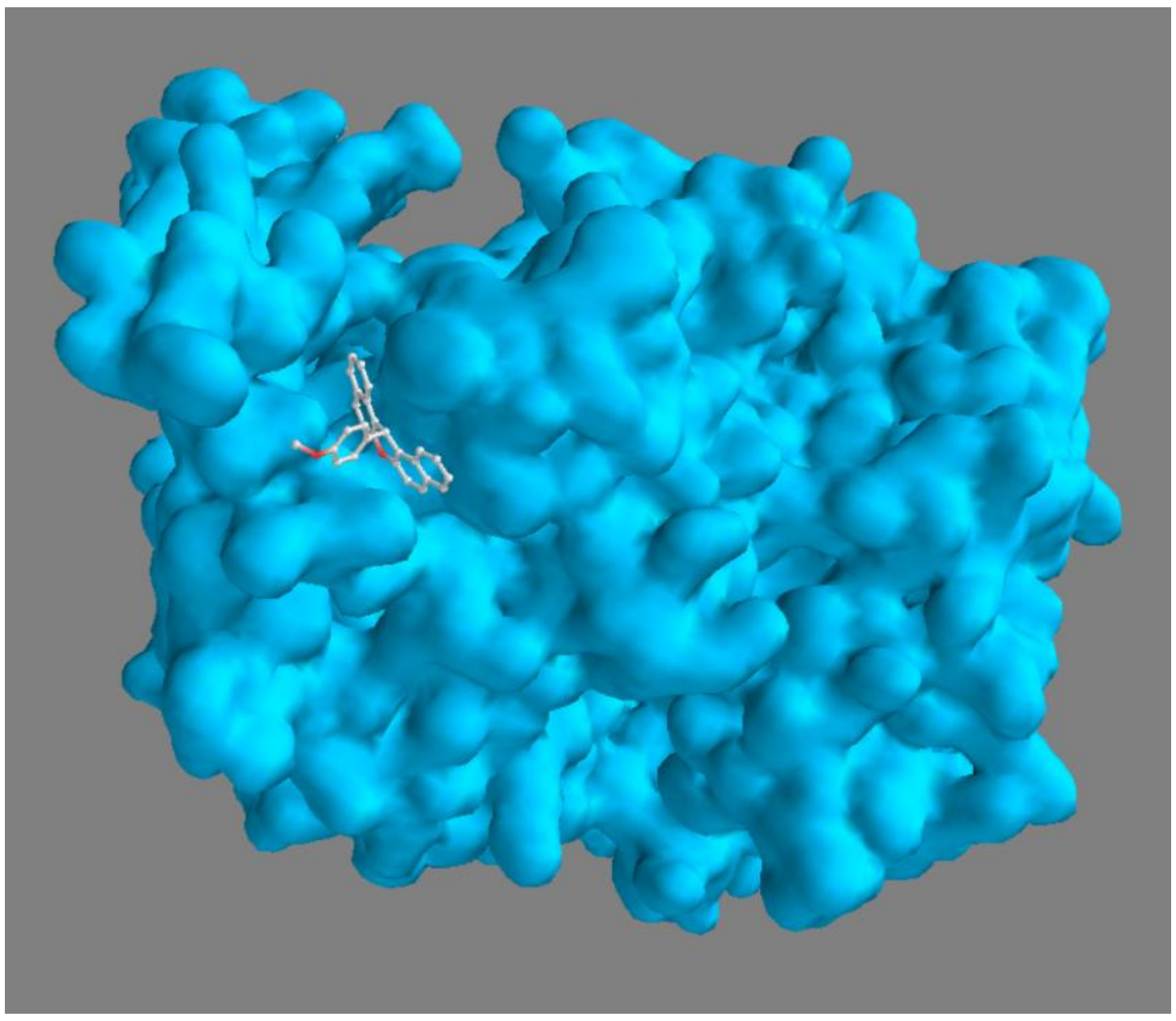

Fig. S113: Compound 6i. Protein surface visualization with bounded ligand

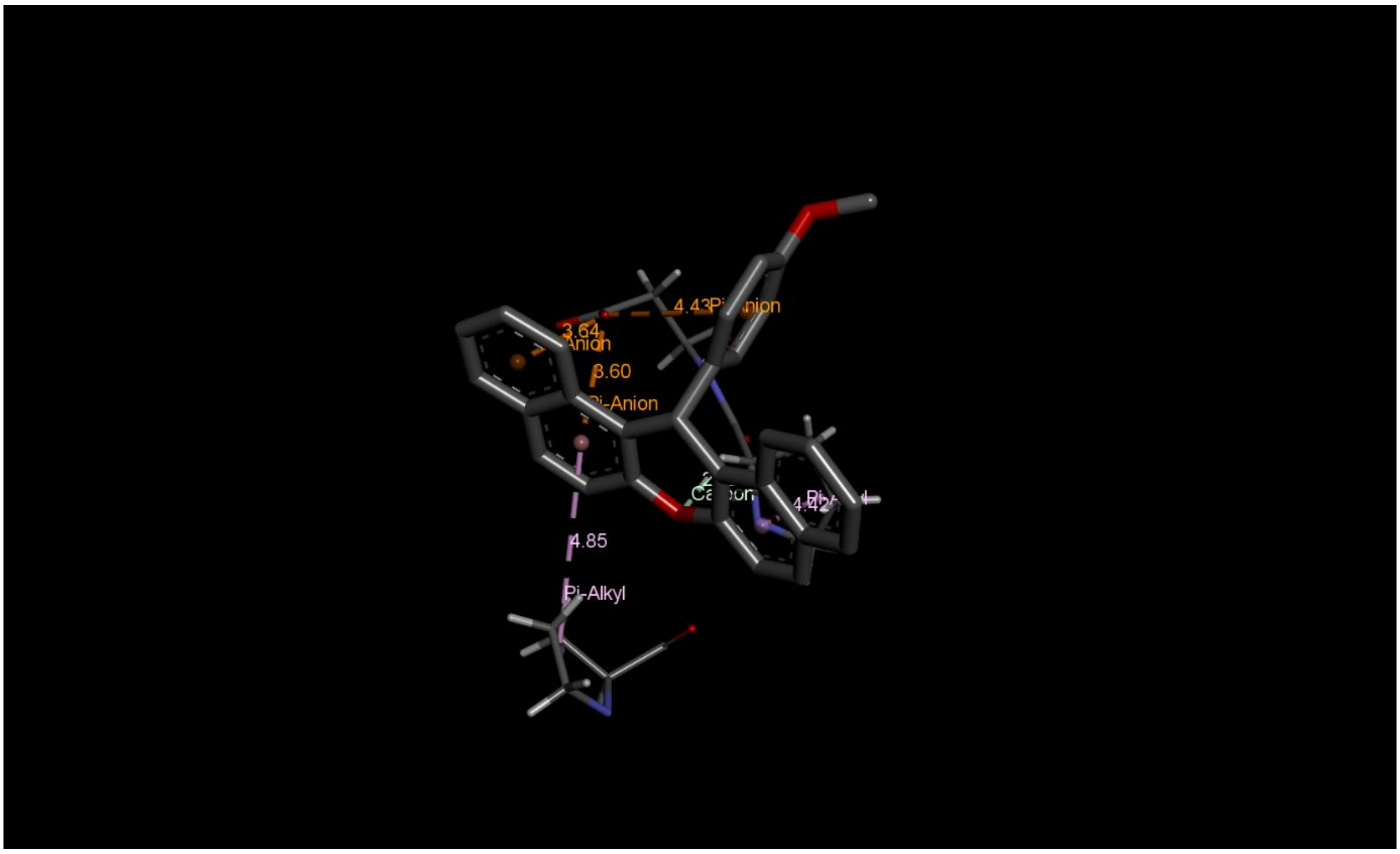

Fig. S114. Compound 6i. Binding pose of the ligand with the active site

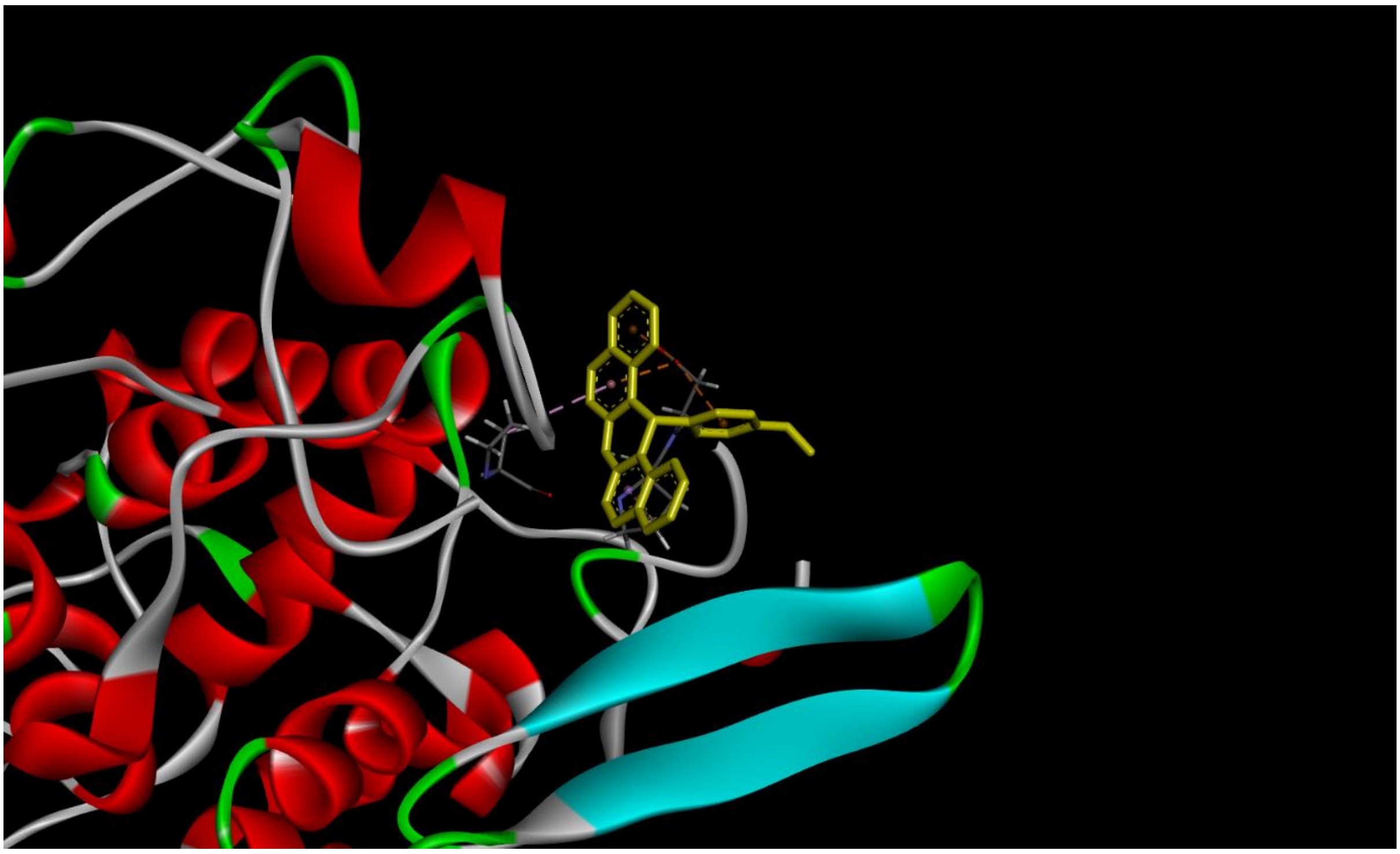

Fig. S115. Compound 6i. Ribbon diagram of the protein-ligand interaction

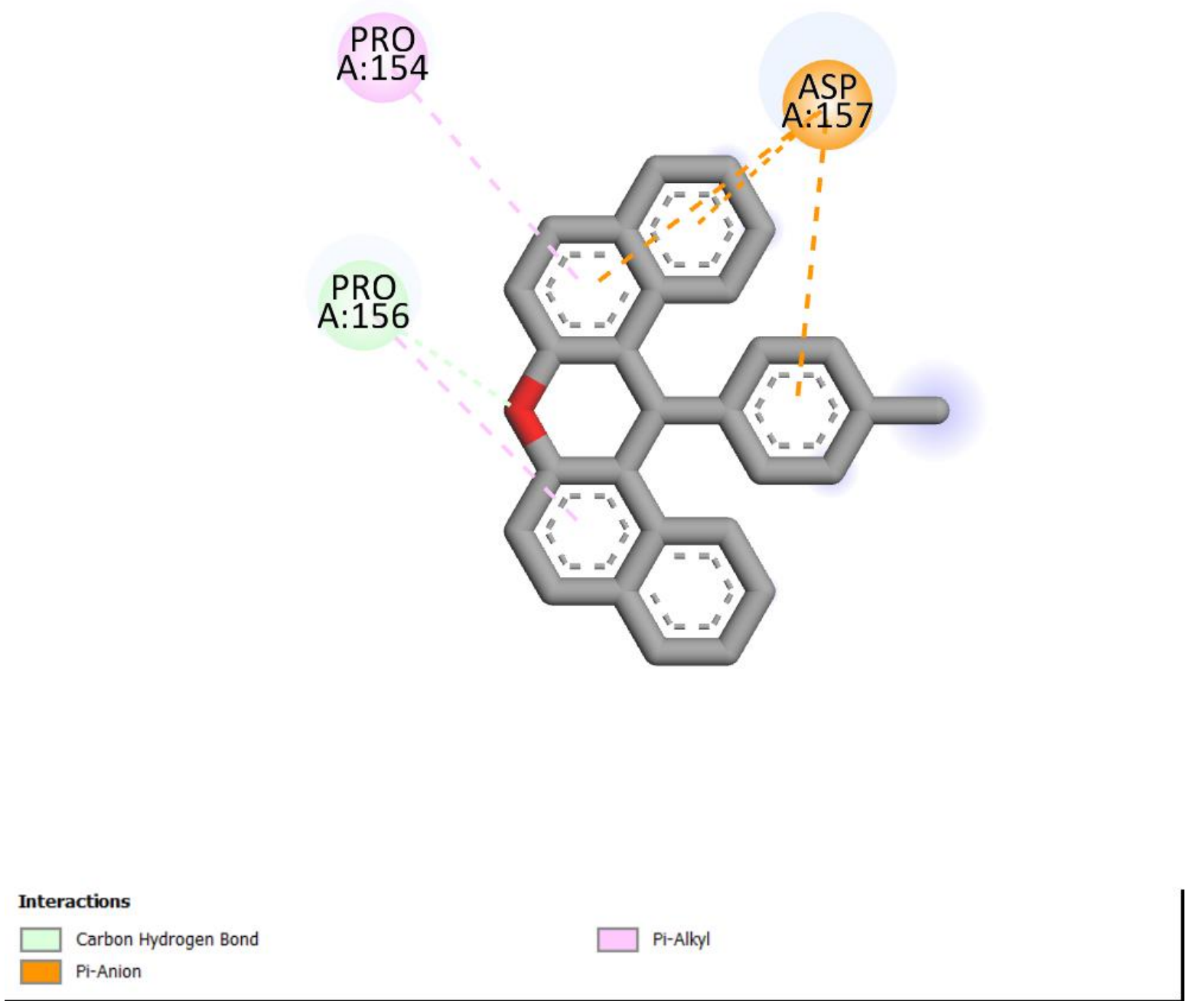

Fig. S116. Compound 6j. 2D-Interaction of ligand-protein

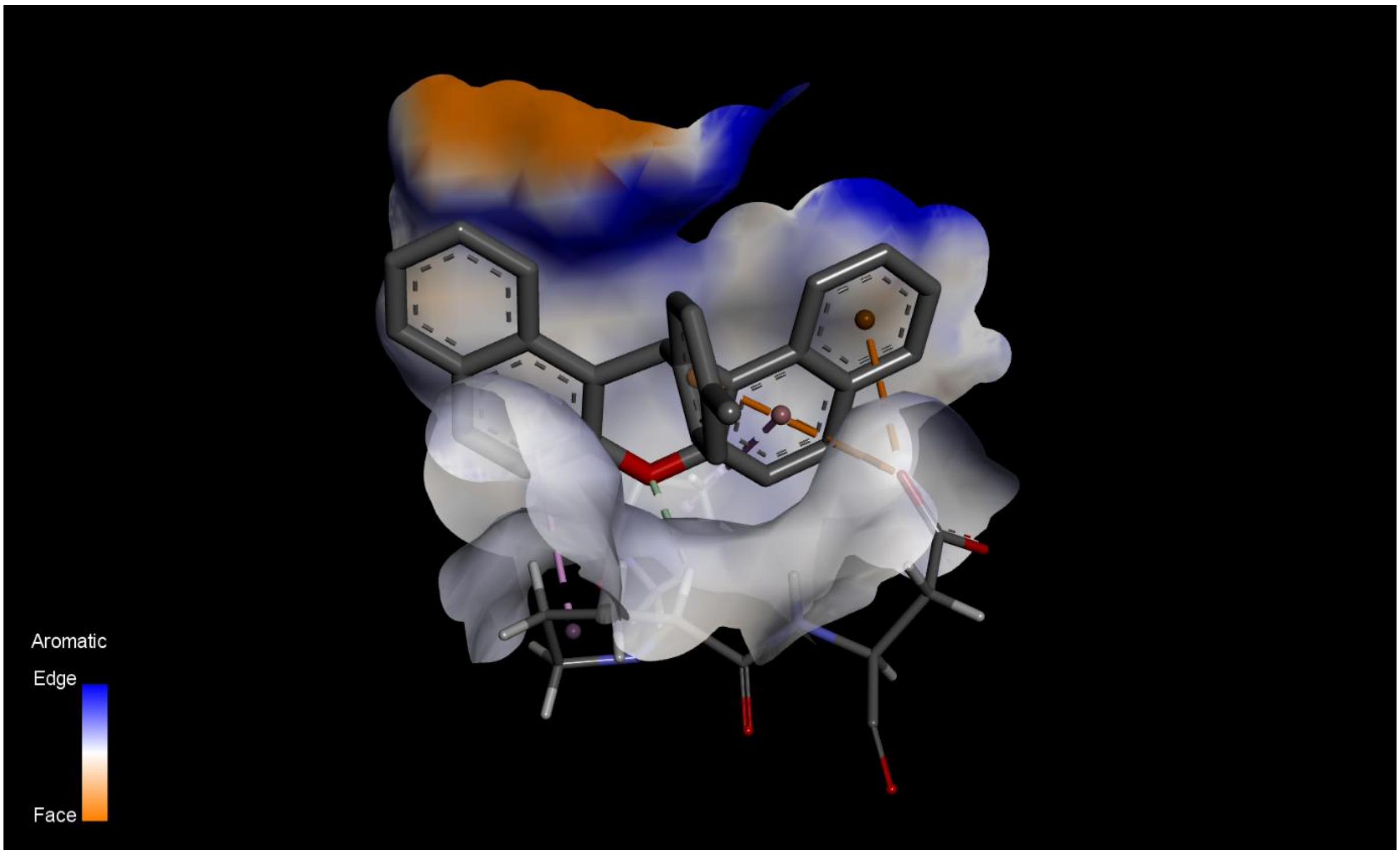

Fig. S117. Compound 6j. Visualization of aromatic face-edge interaction

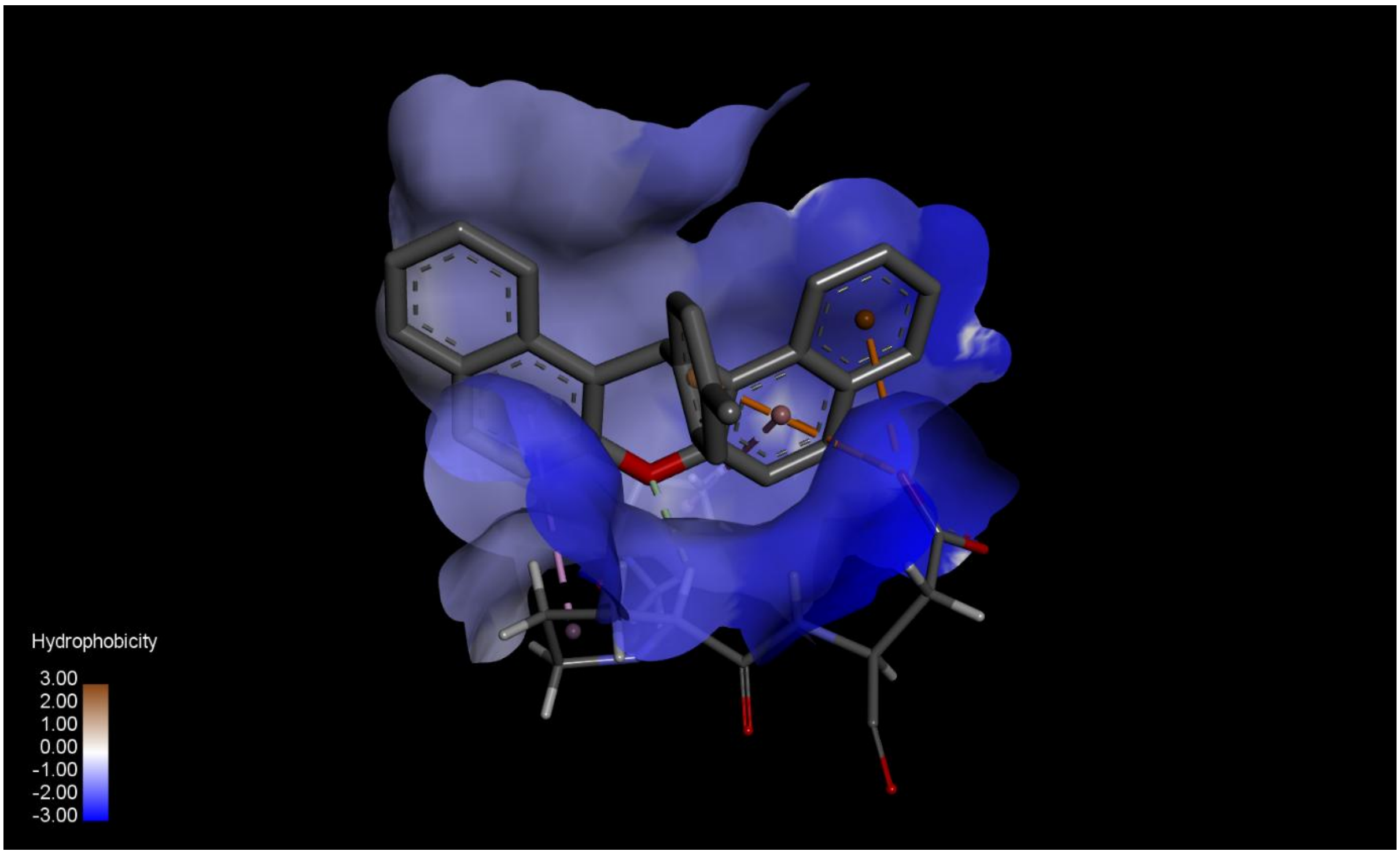

Fig. S118. Compound 6j. Visualization of hydrophobic interaction in the active site

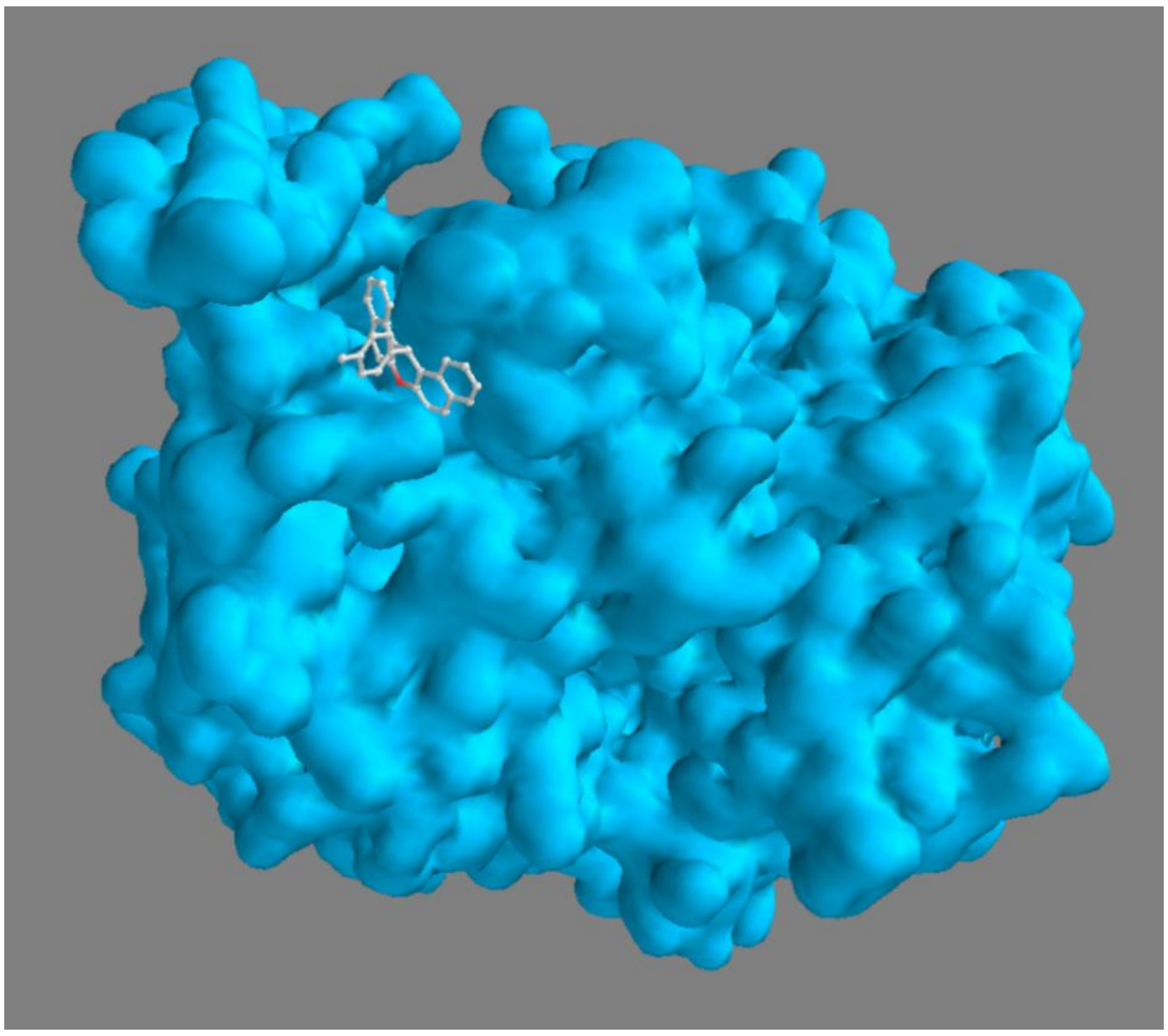

Fig. S119. Compound 6j. Protein surface visualization with bounded ligand

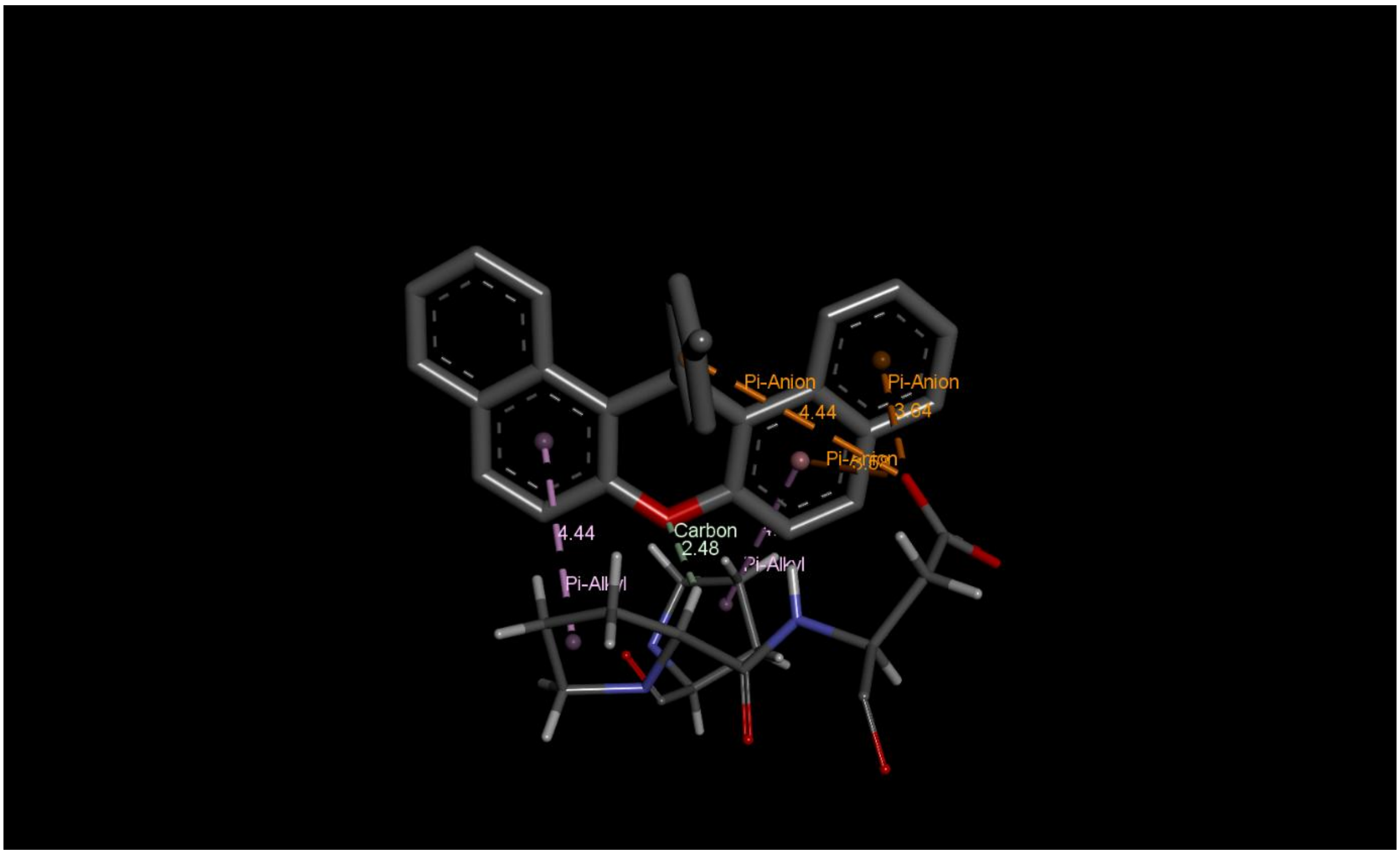

Fig. S120. Compound 6j. Binding pose of the ligand with the active site

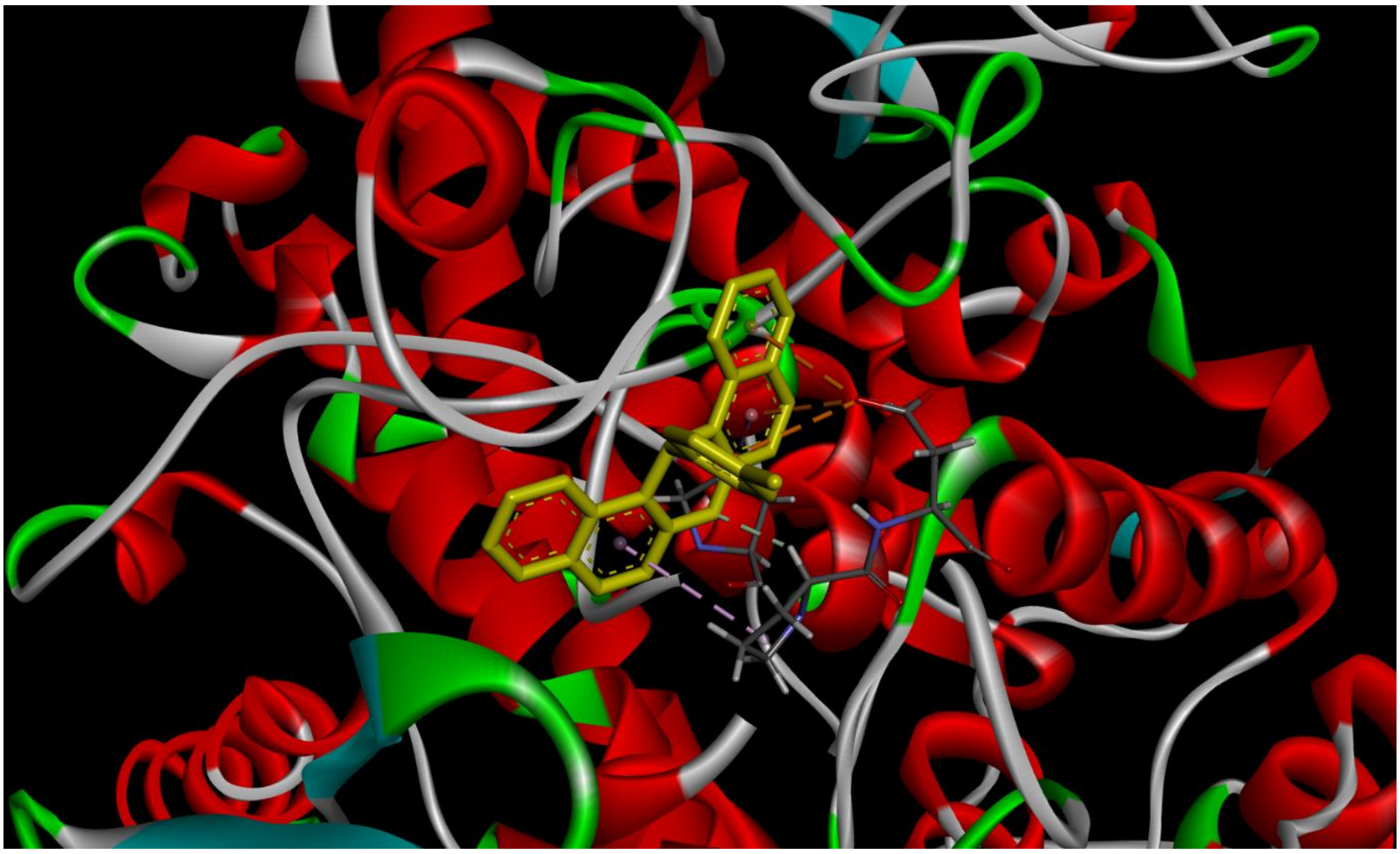

Fig. S121. Compound 6j. Ribbon diagram of the protein-ligand interaction

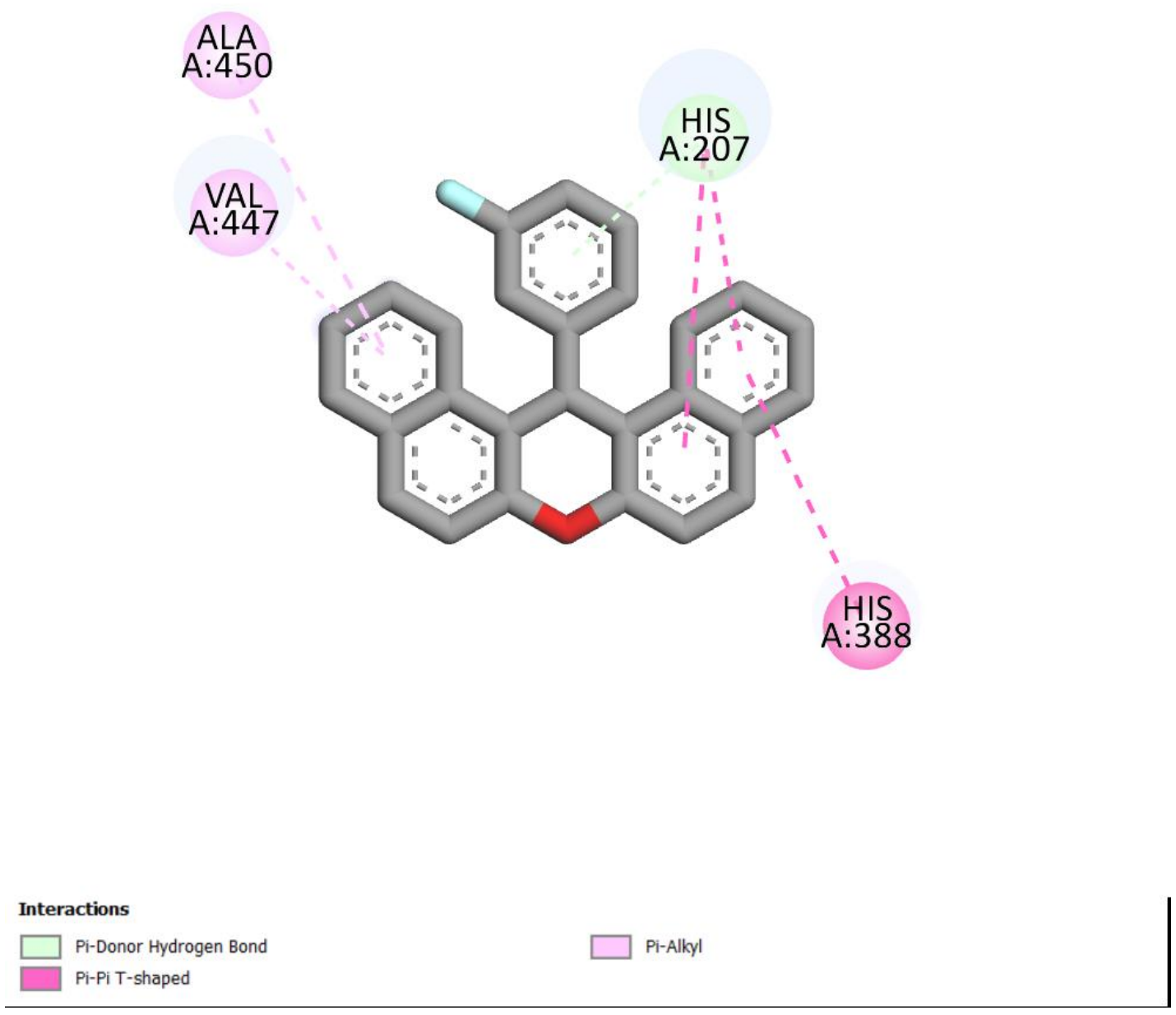

Fig. S122. Compound 6k. 2D-Interaction of ligand-protein

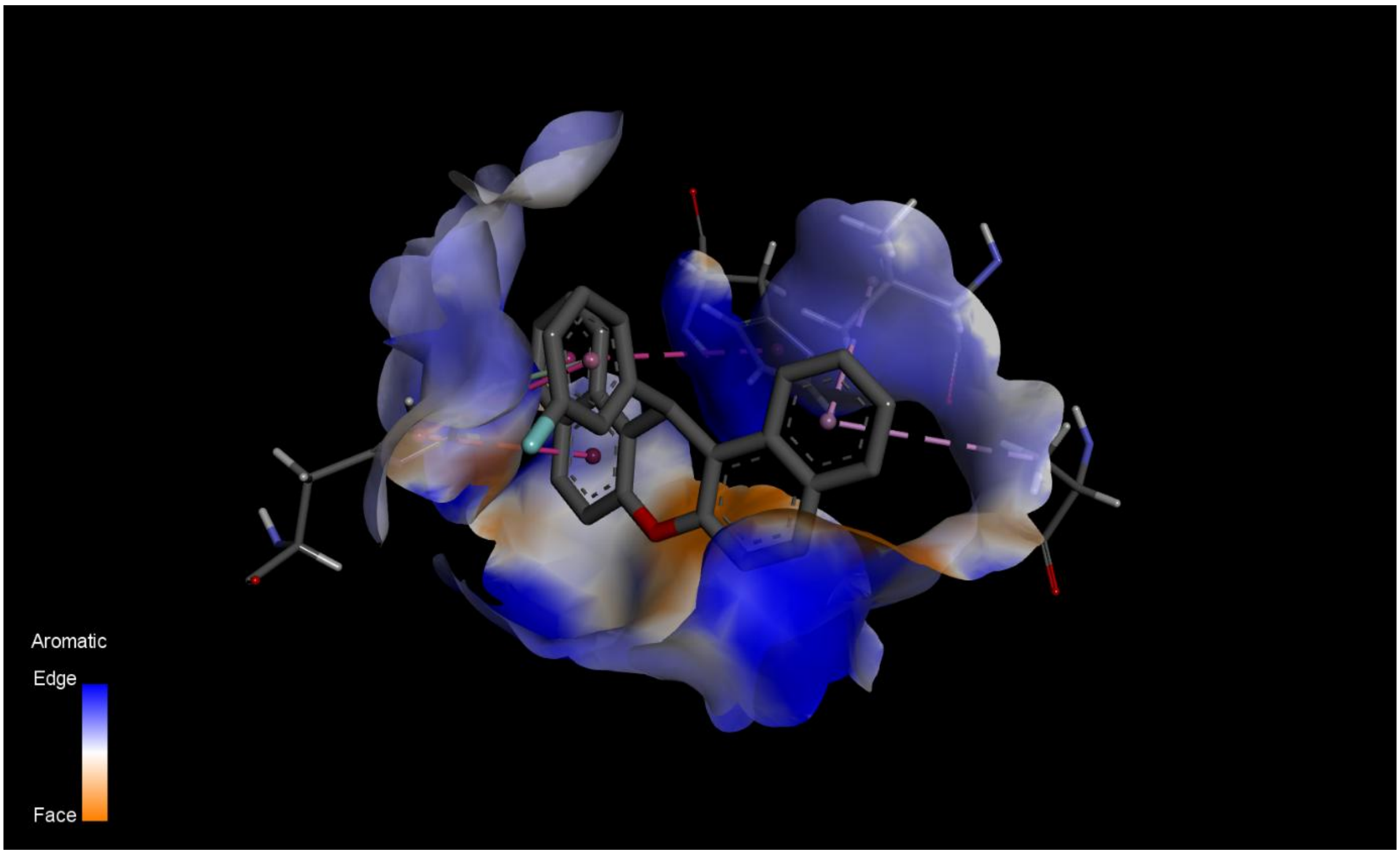

Fig. S123. Compound 6k. Visualization of aromatic face-edge interaction

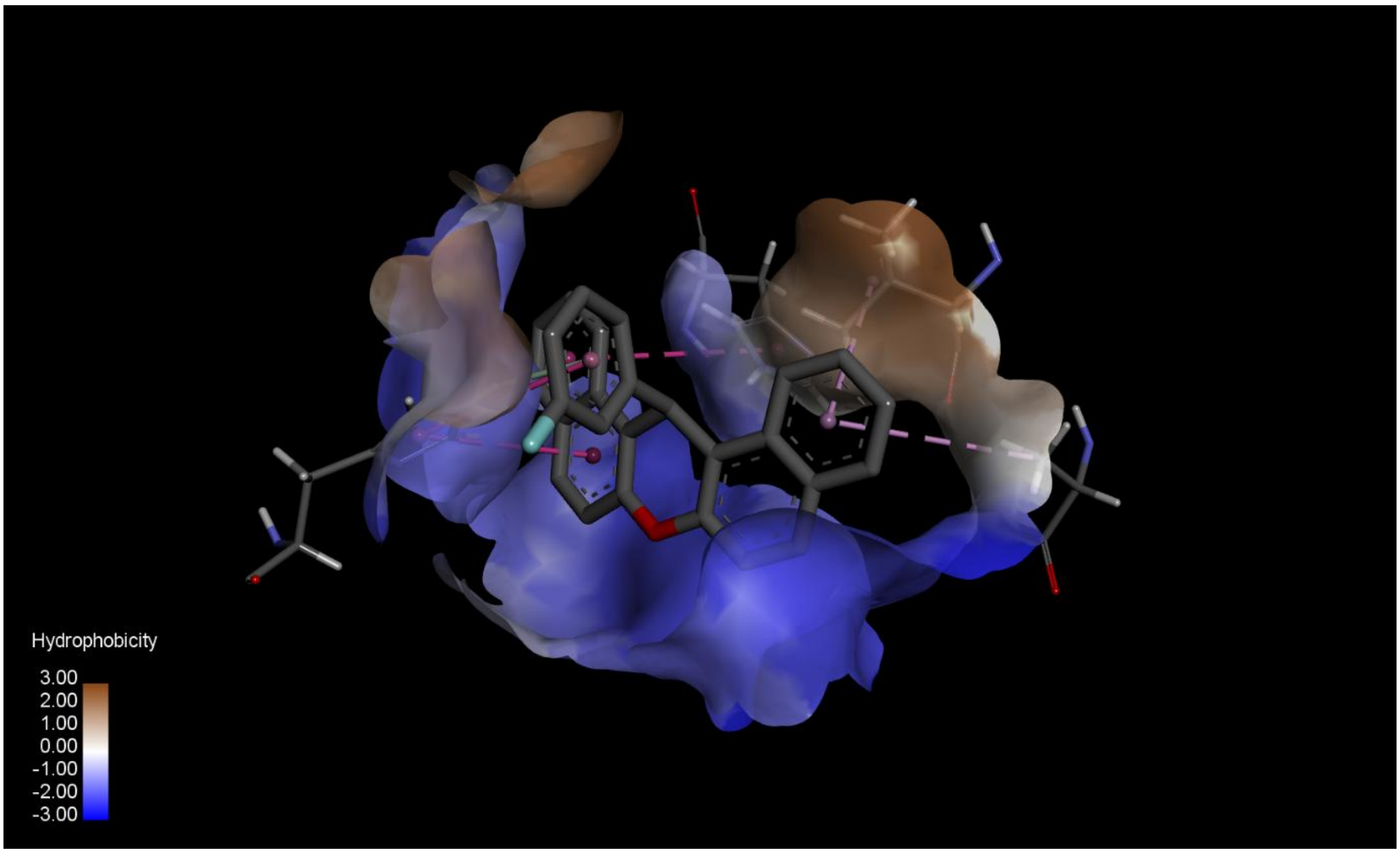

Fig.S124. Compound 6k. Visualization of hydrophobic interaction in the active site

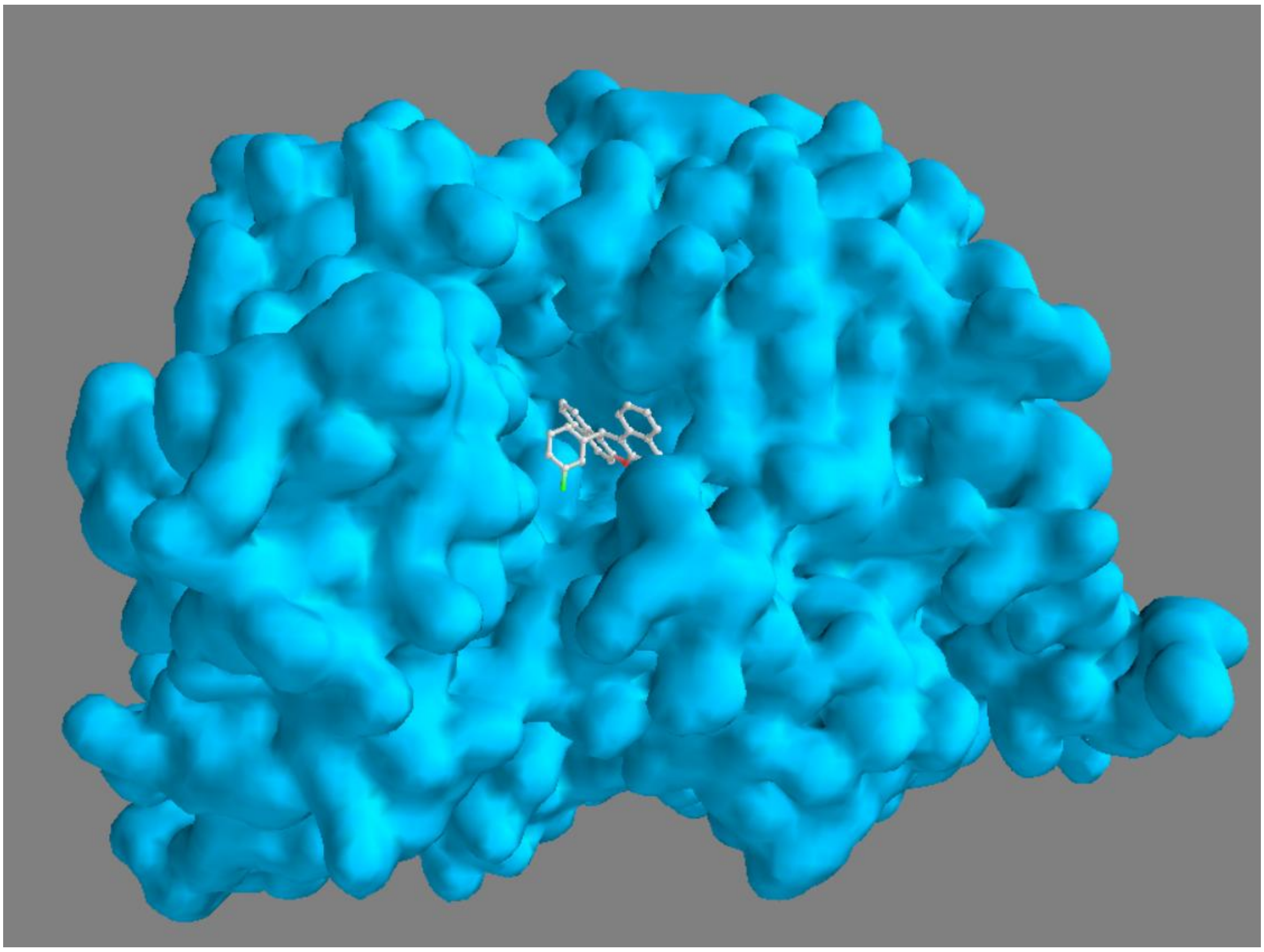

Fig.S125: Compound 6k. Protein surface visualization with bounded ligand

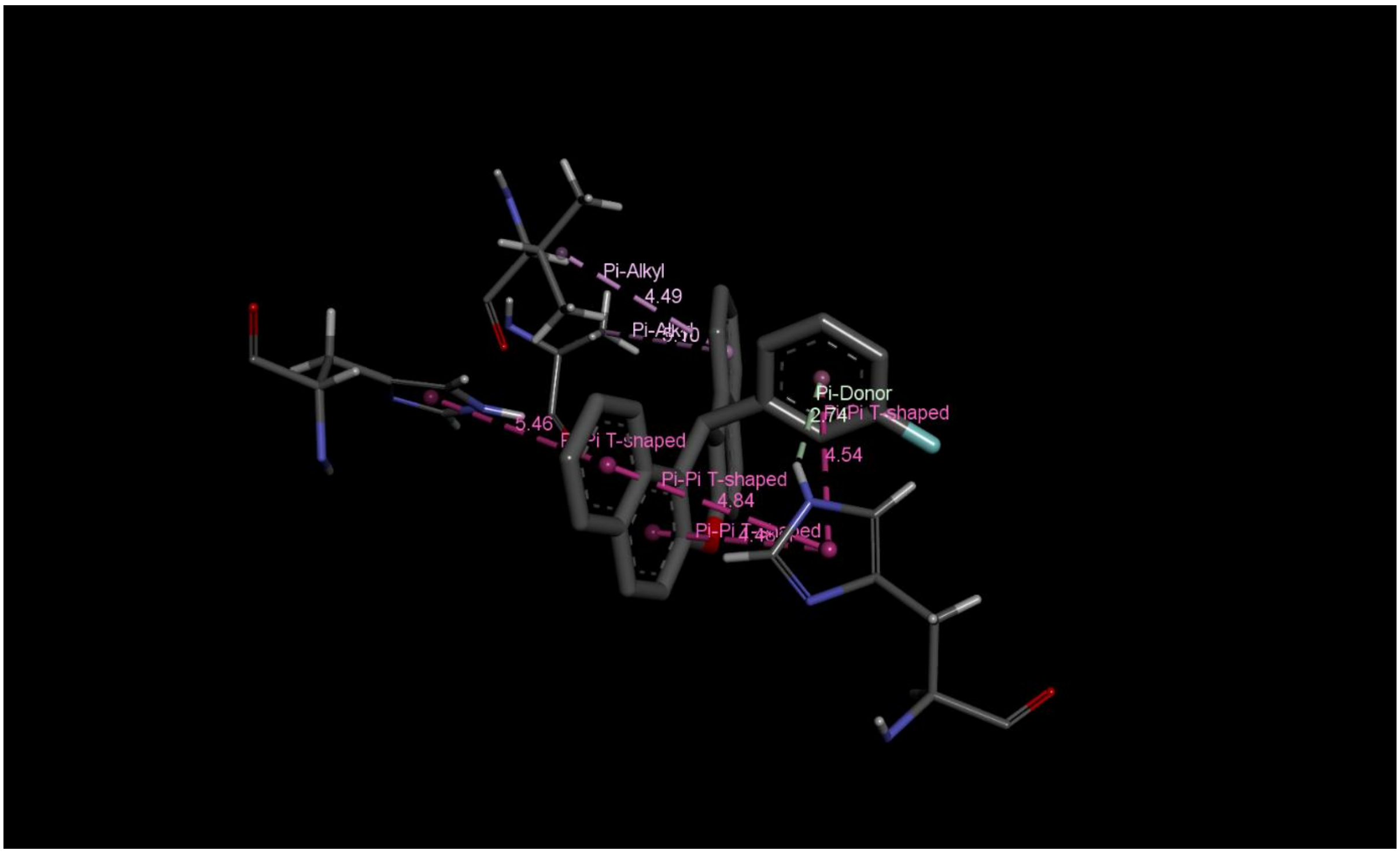

Fig. S126. Compound 6k. Binding pose of the ligand with the active site

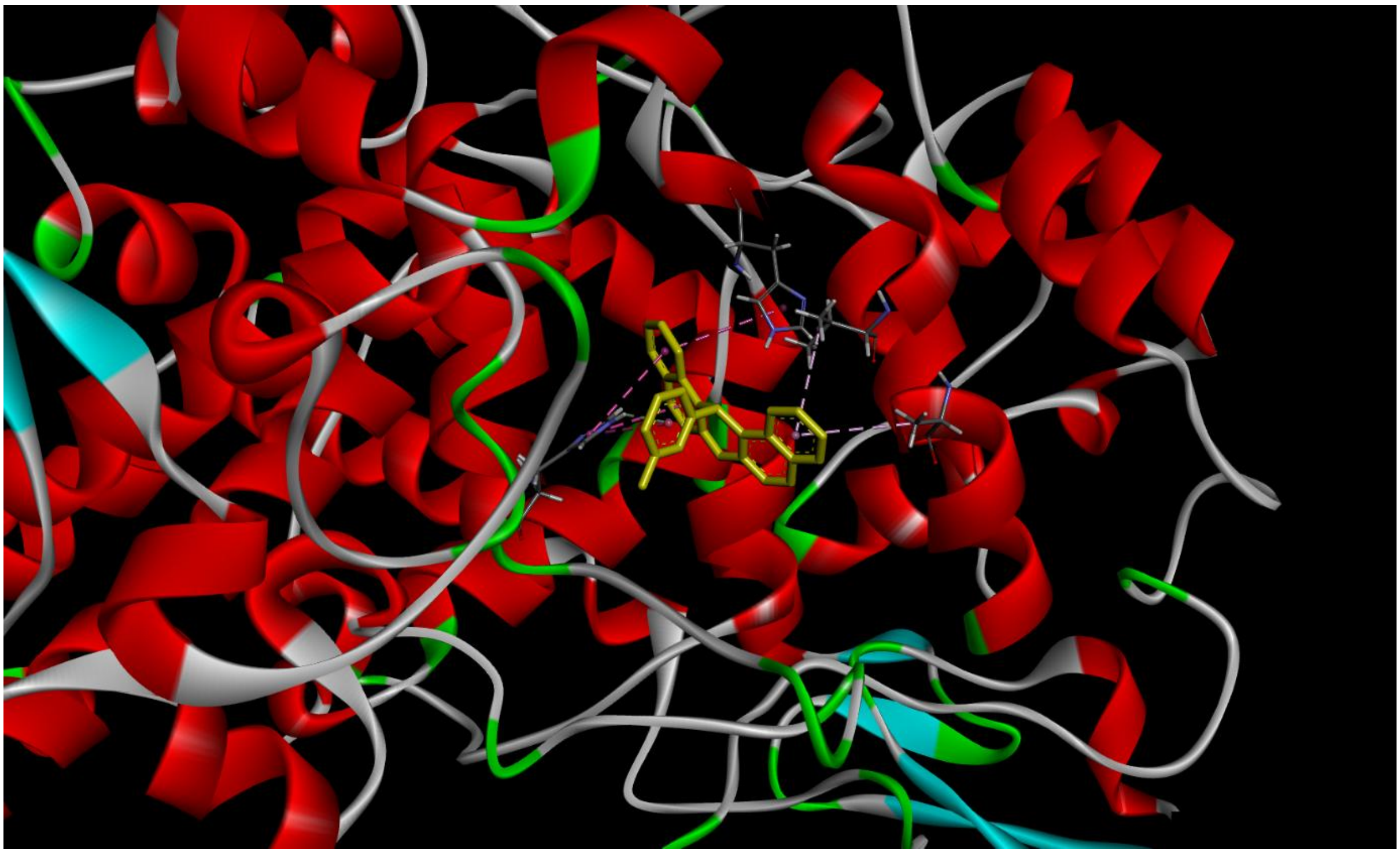

Fig. S127. Compound 6k. Ribbon diagram of the protein-ligand interaction

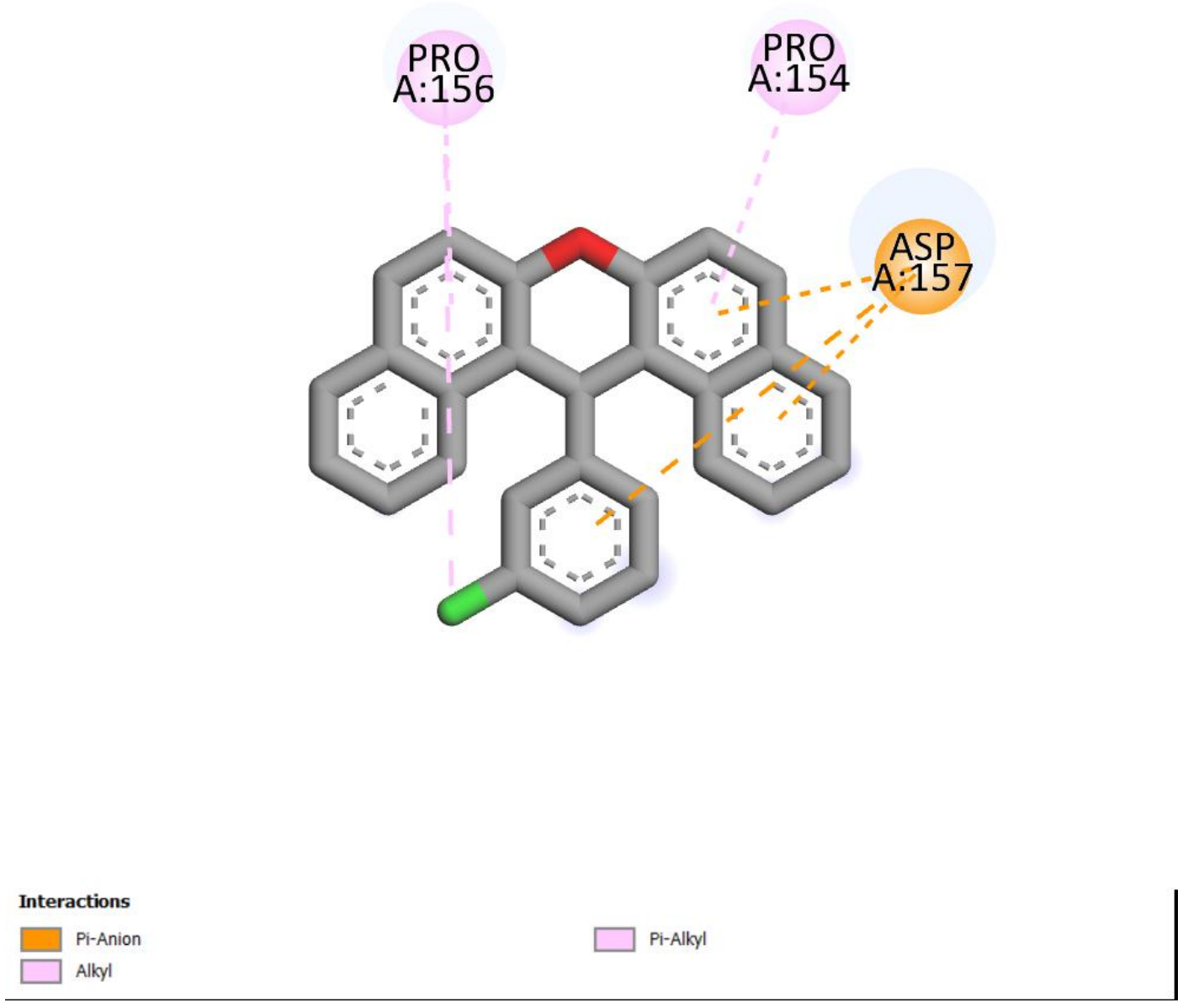

Fig. S128. Compound 6l .2D-Interaction of ligand-protein

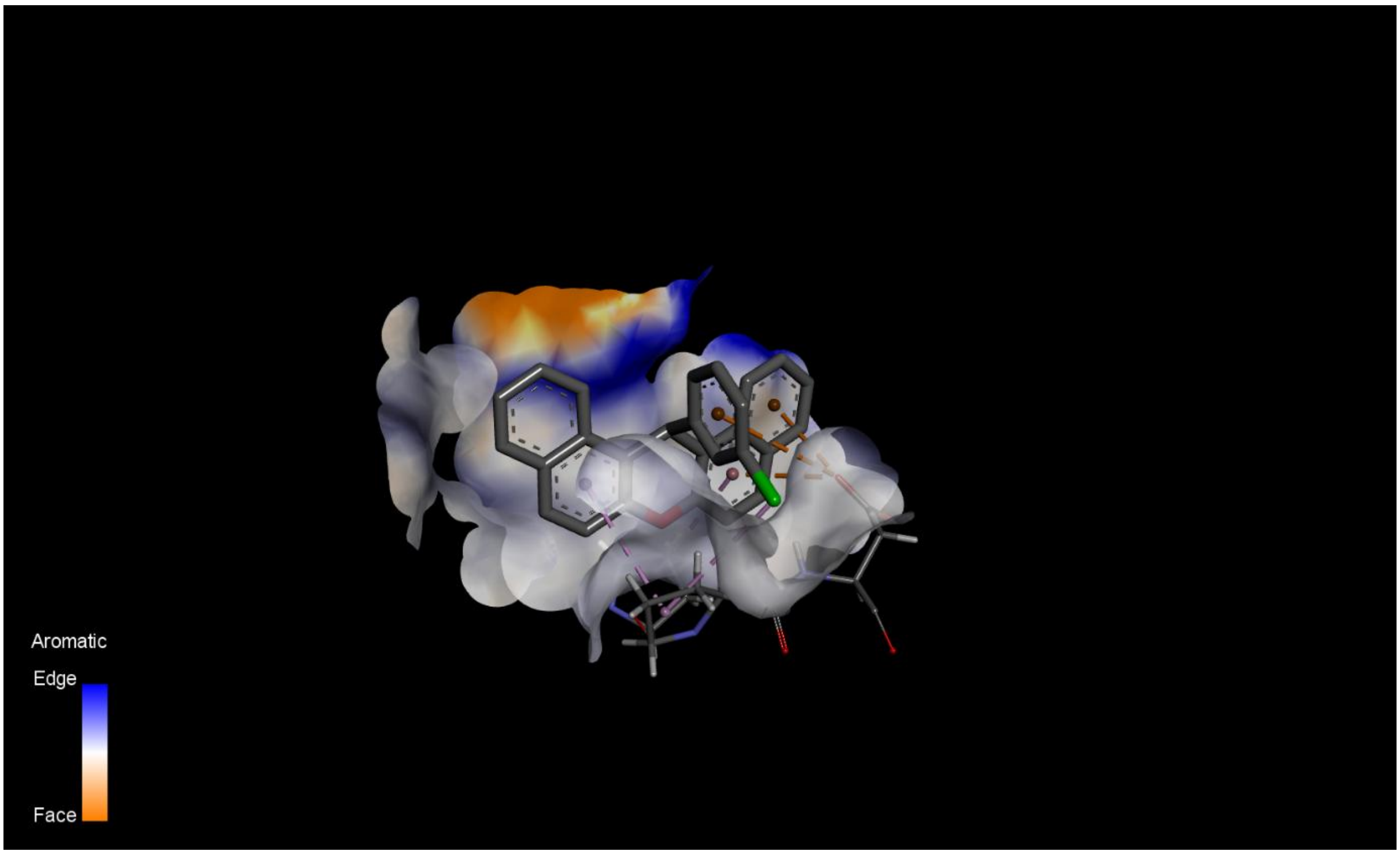

Fig. S129. Compound 6l. Visualization of aromatic face-edge interaction

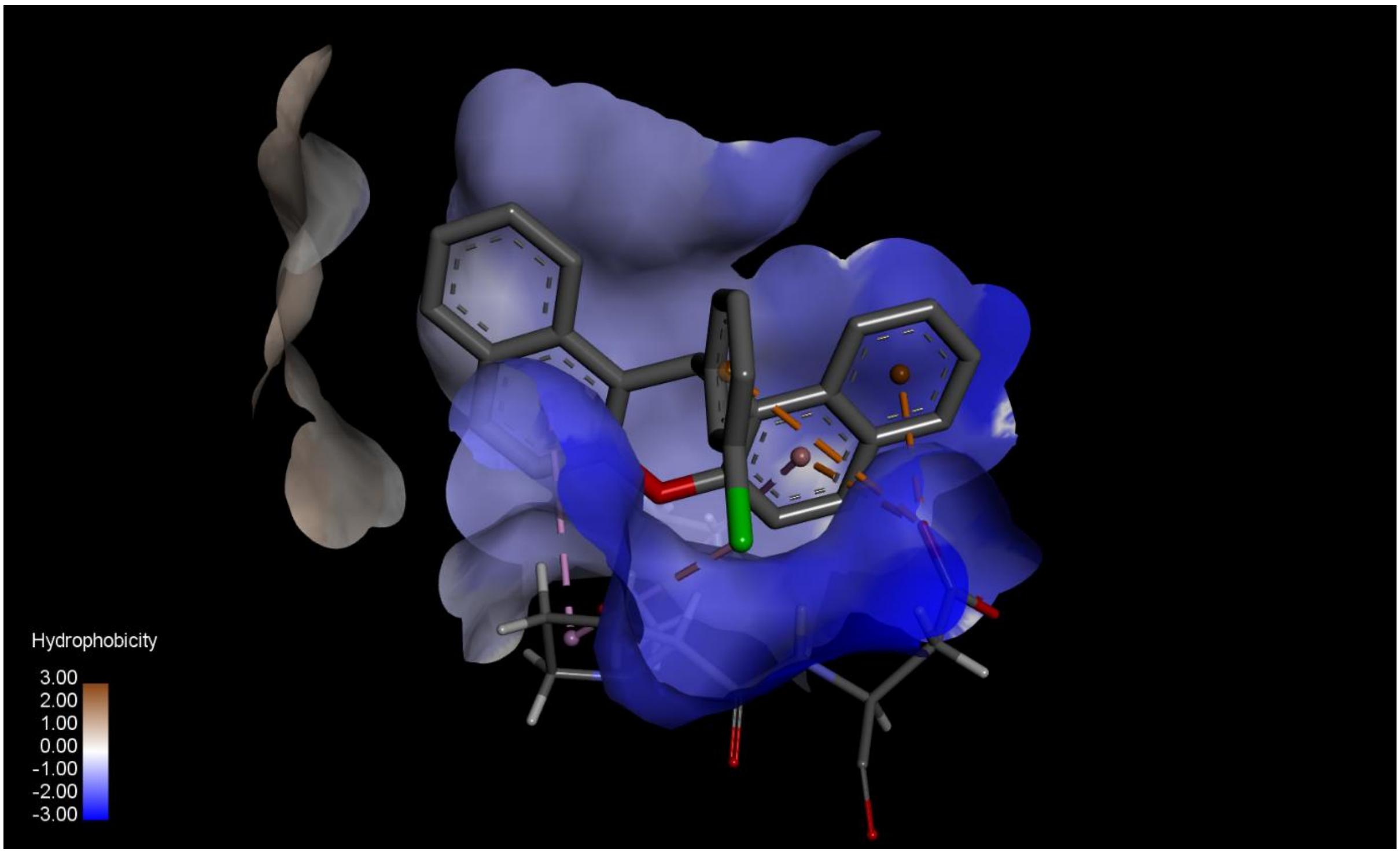

Fig. S130. Compound 6l. Visualization of hydrophobic interaction in the active site

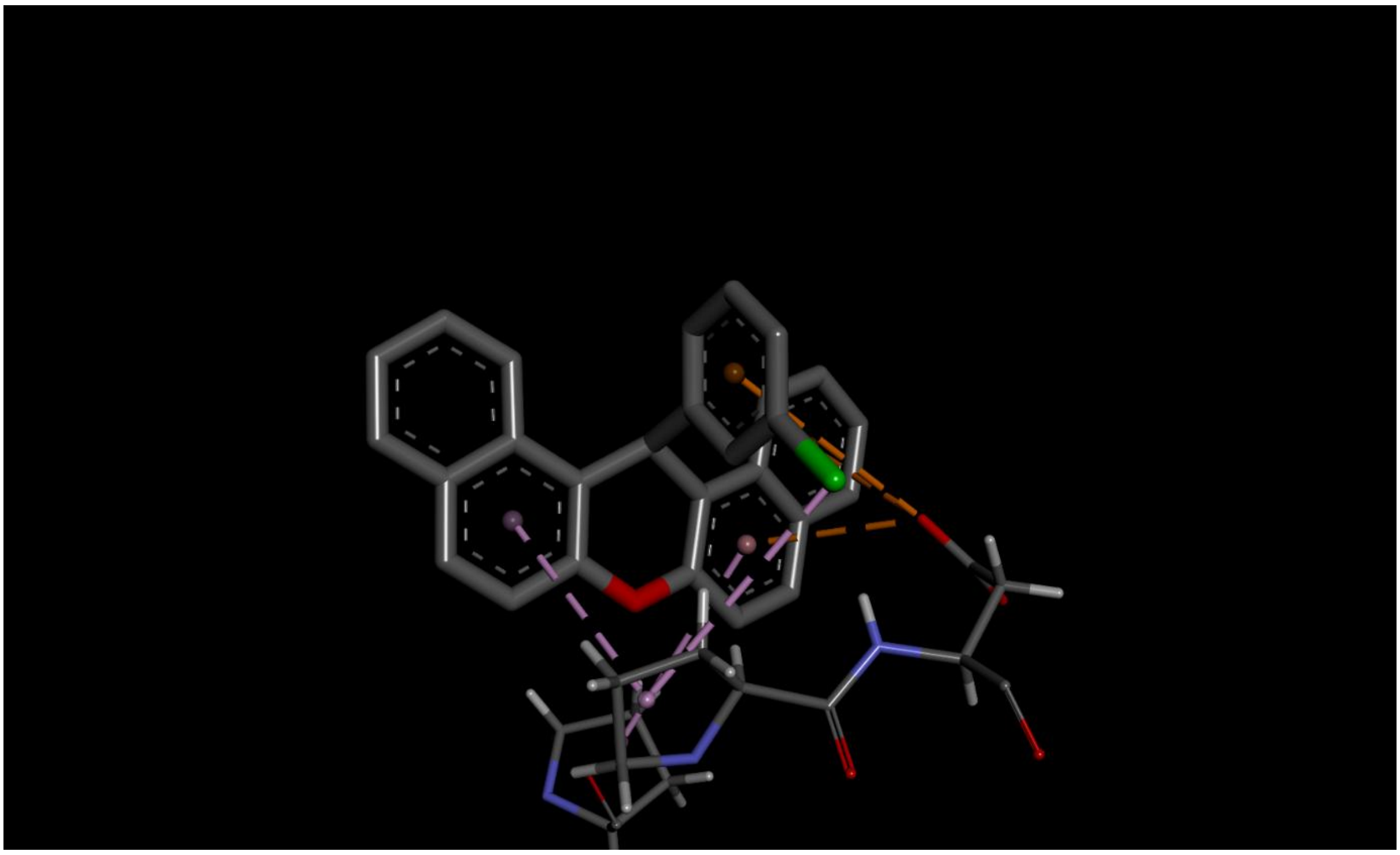

Fig. S131. Compound 6l. Binding pose of the ligand with the active site

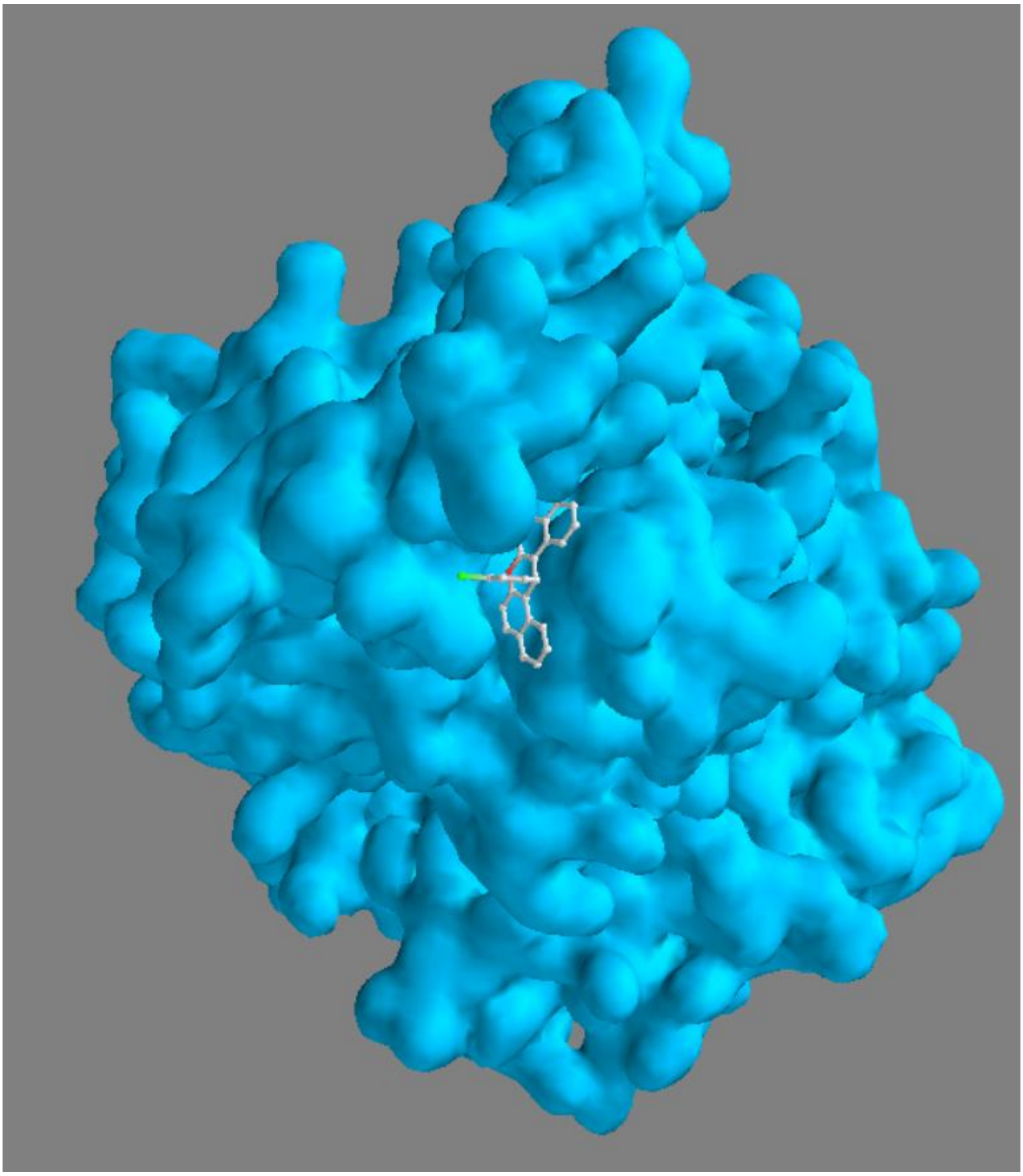

Fig. S132. Compound 6l. Protein surface visualization with bounded ligand

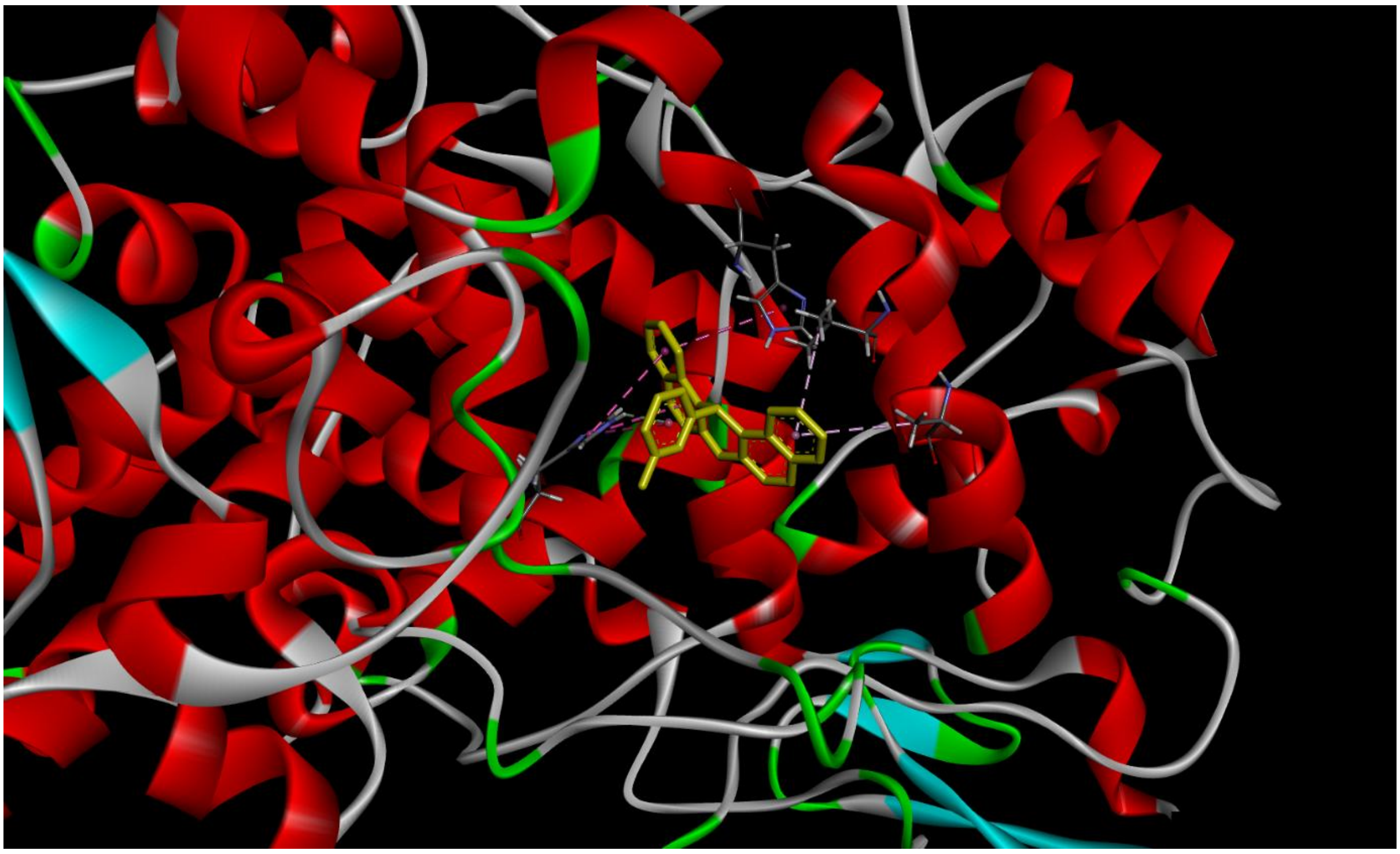

Fig. S133. Compound 6l. Ribbon diagram of the protein-ligand interaction

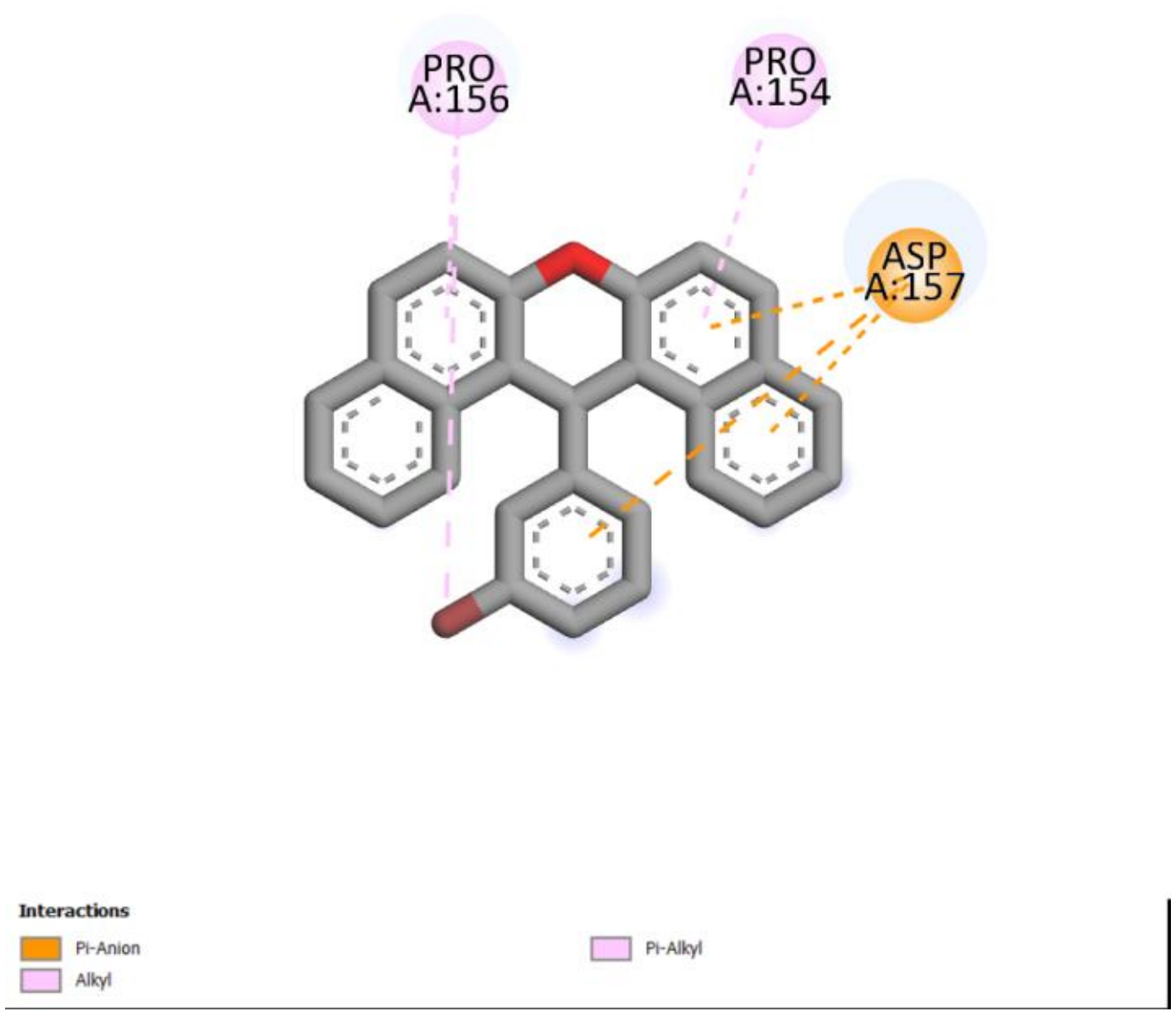

Fig. S134. Compound 6m.2D-Interaction of ligand-protein

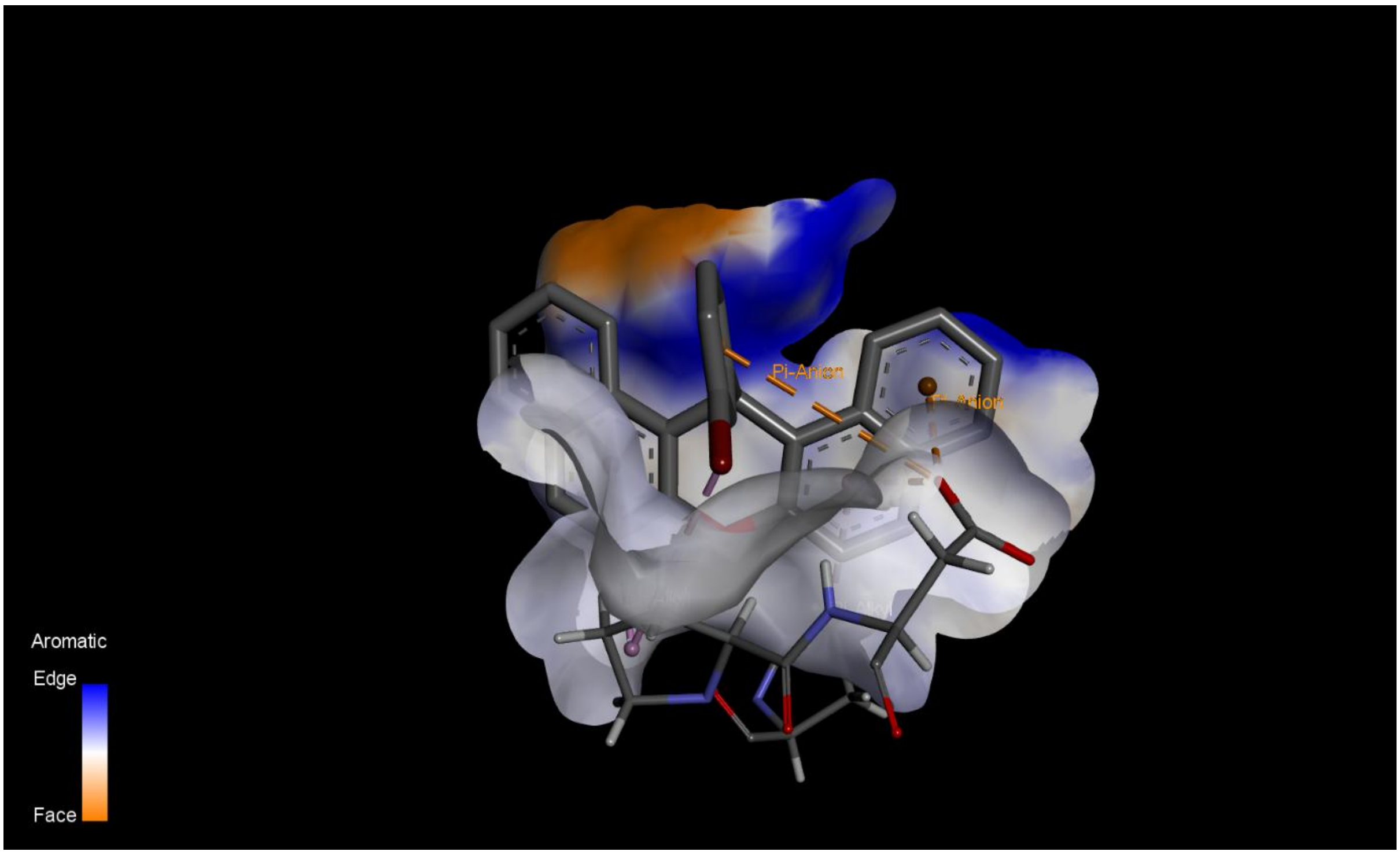

Fig. S135. Compound 6m. Visualization of aromatic face-edge interaction

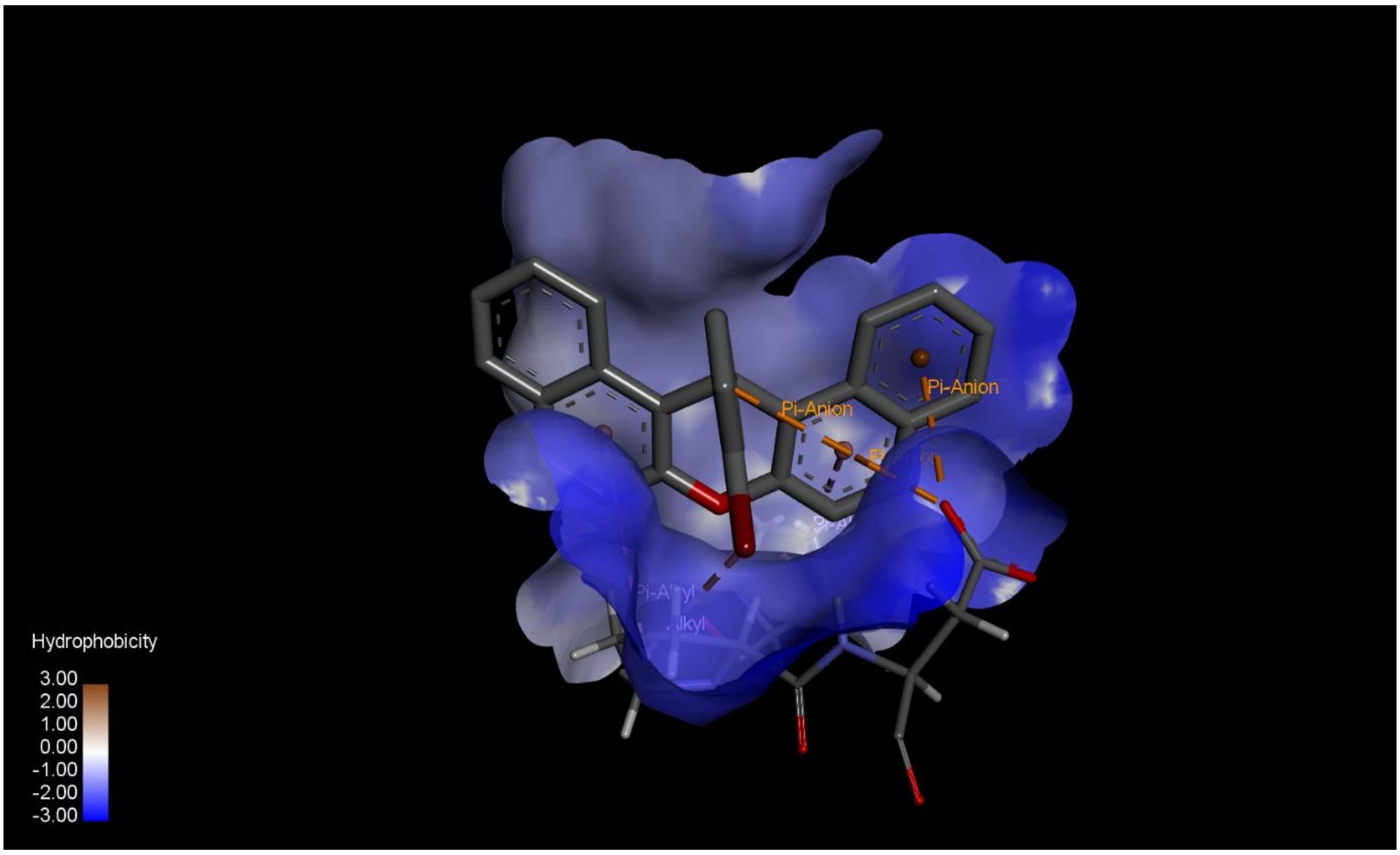

Fig. S136. Compound 6m. Visualization of hydrophobic interaction in the active site

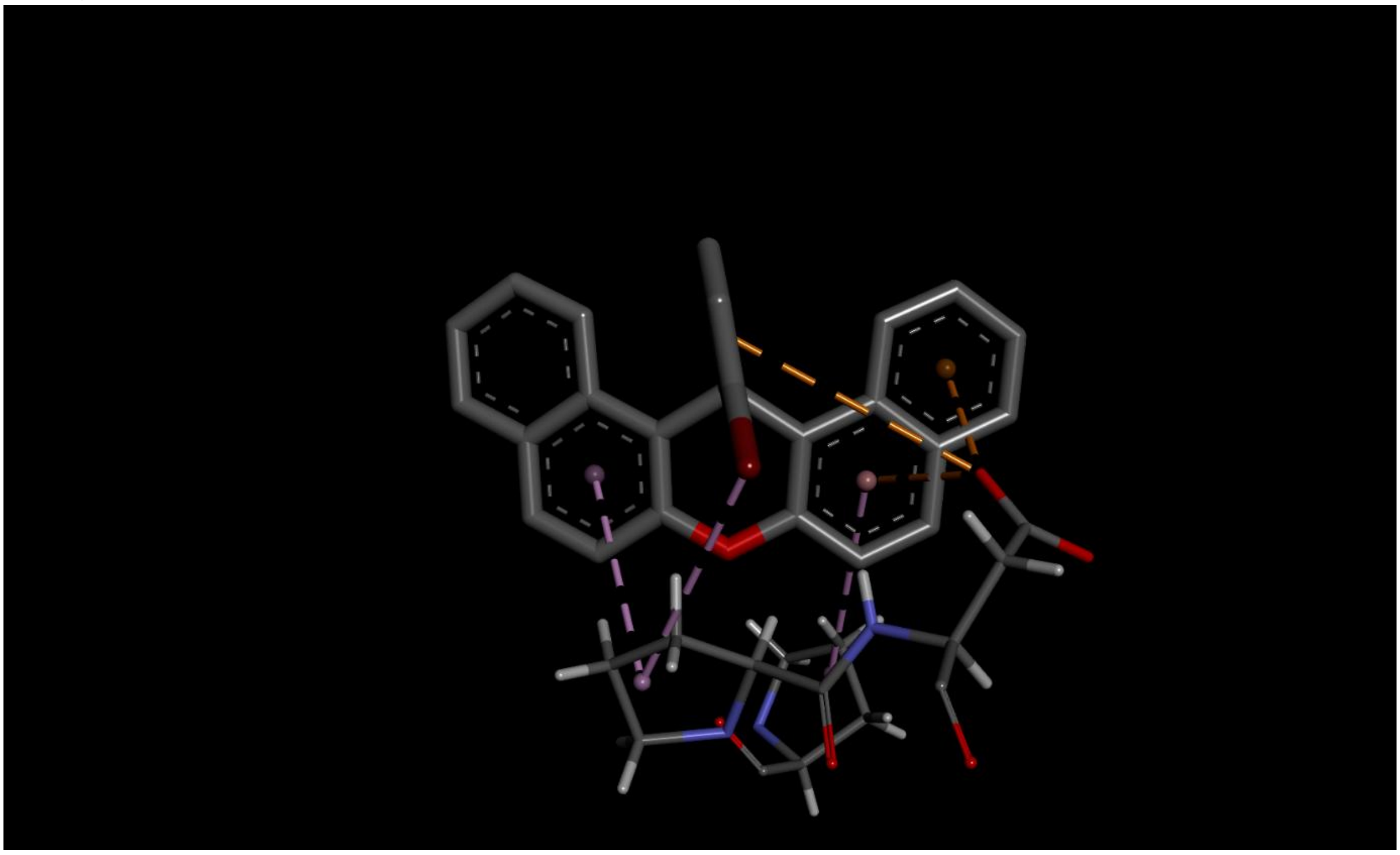

Fig. S137. Compound 6m. Binding pose of the ligand with the active site

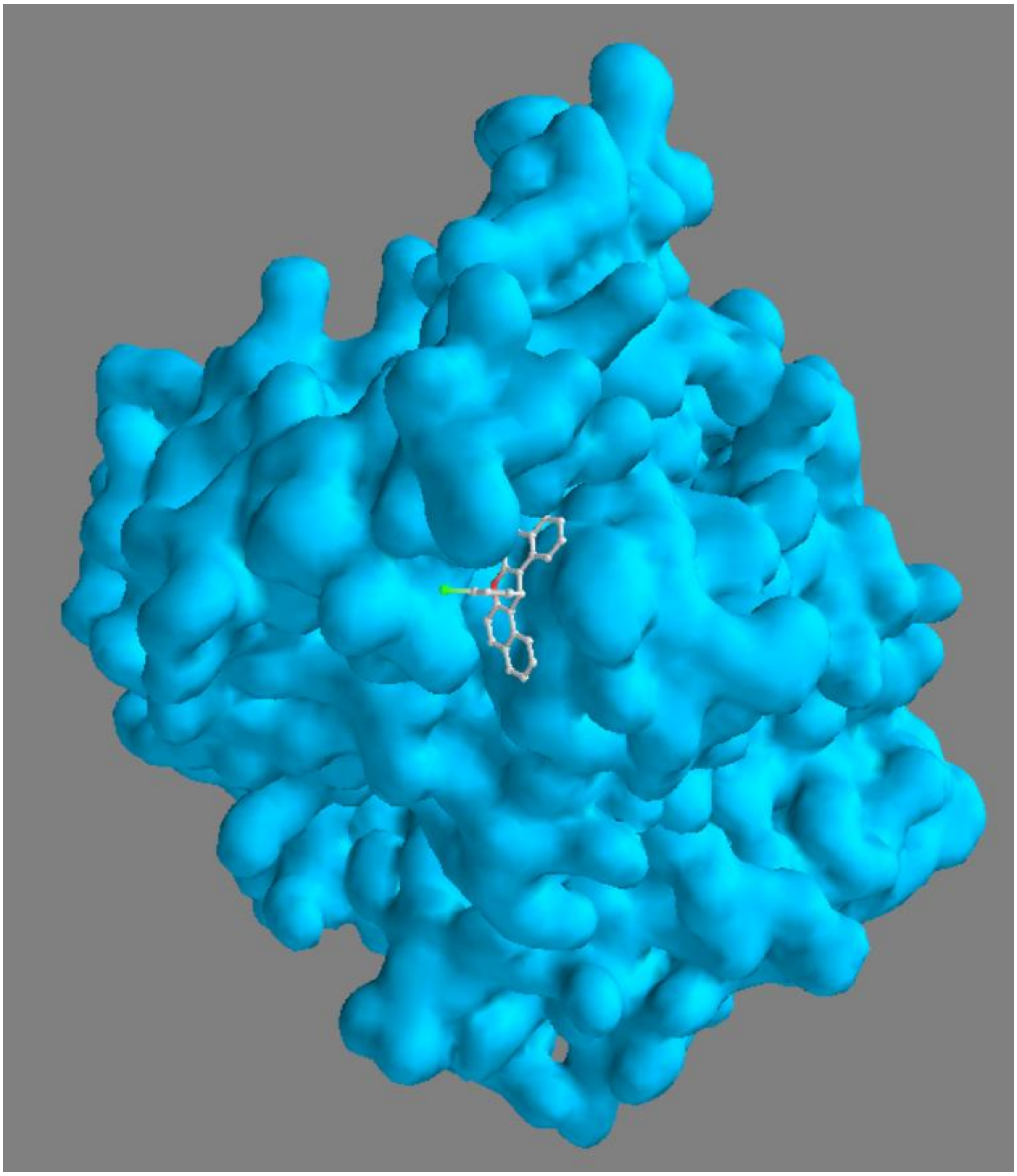

Fig. S138. Compound 6m. Protein surface visualization with bounded ligand

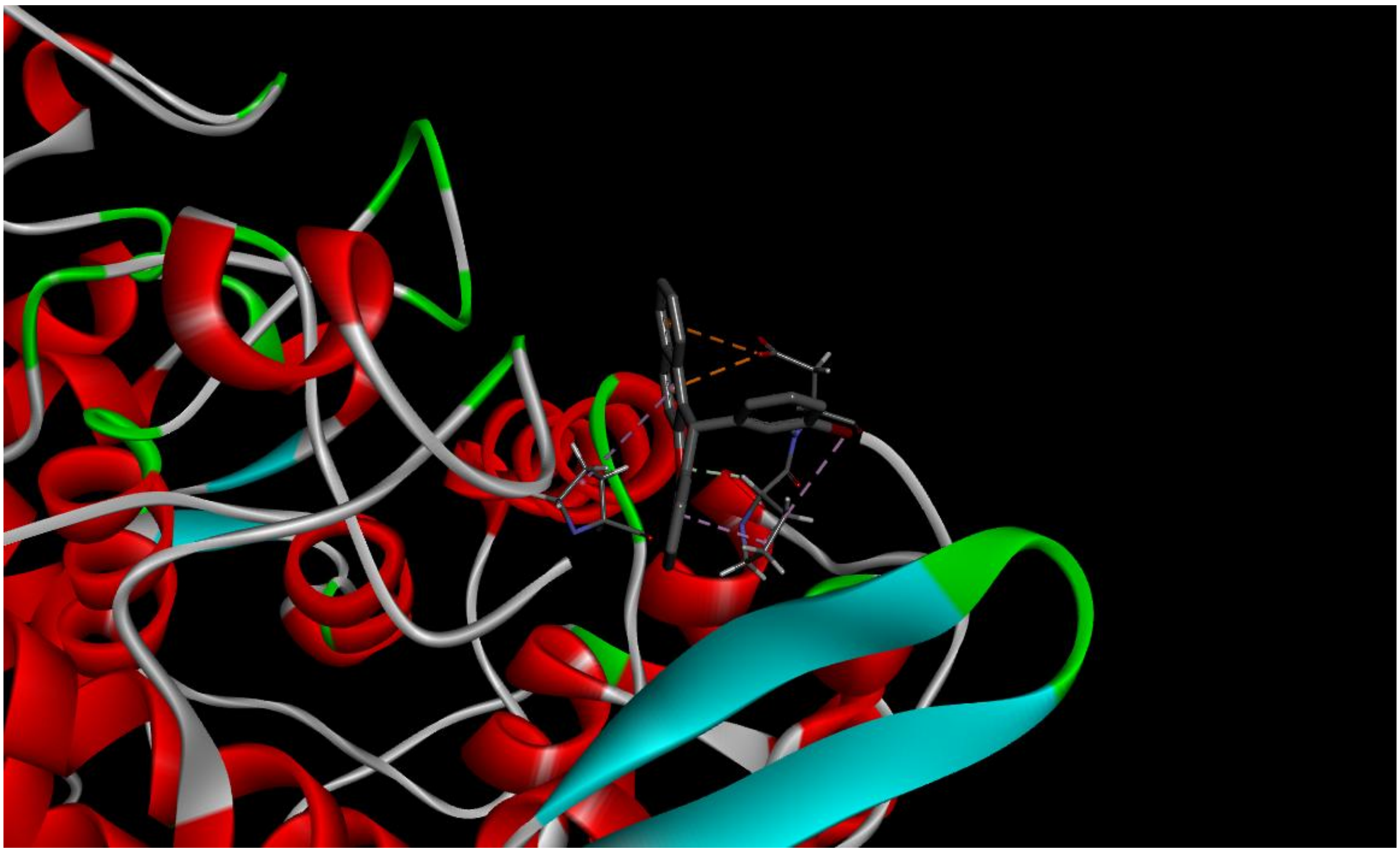

Fig. S139. Compound 6m. Ribbon diagram of the protein-ligand interaction

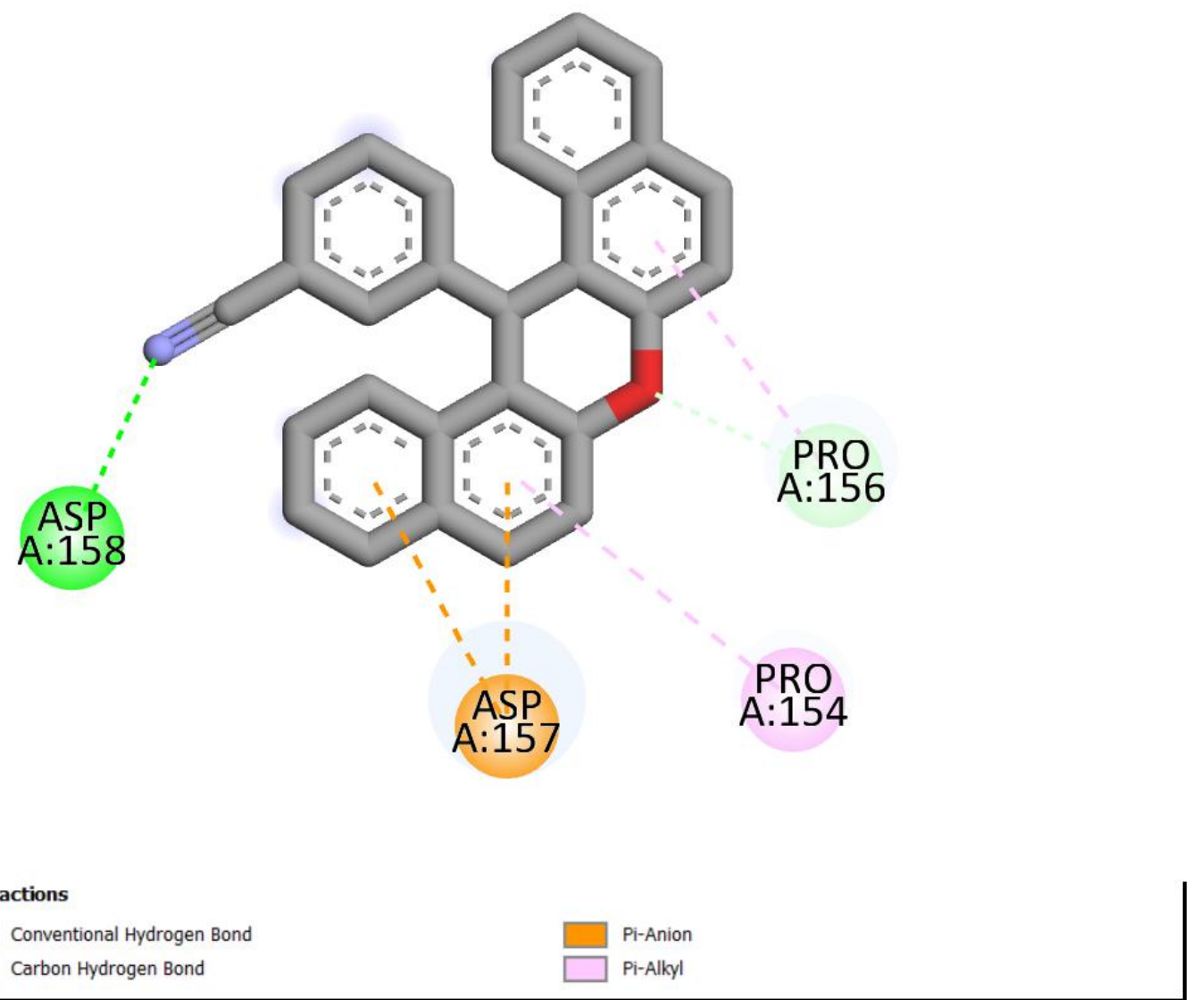

Fig. S140. Compound 6n. 2D-Interaction of ligand-protein

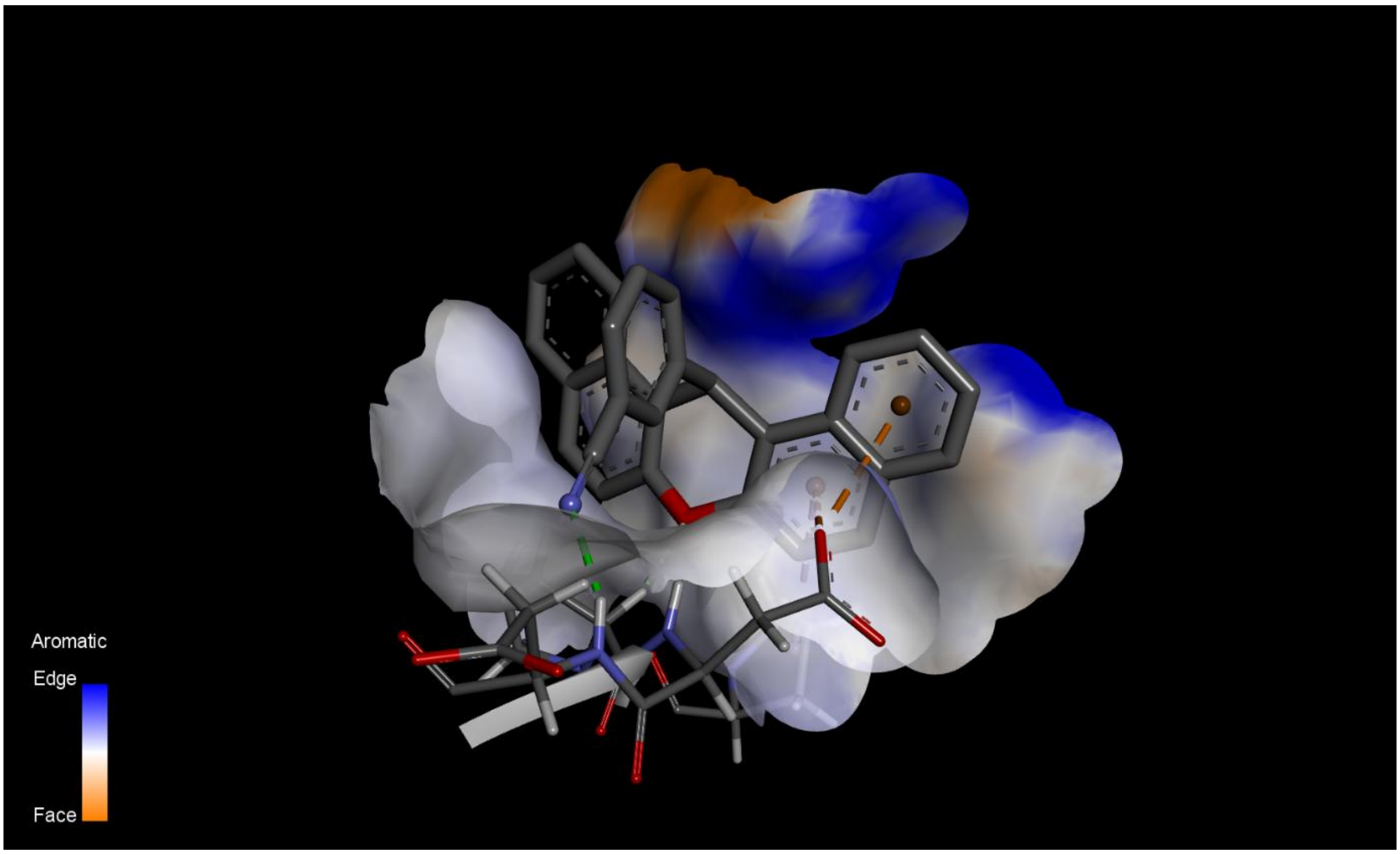

Fig. S141. Compound 6n. Visualization of aromatic face-edge interaction

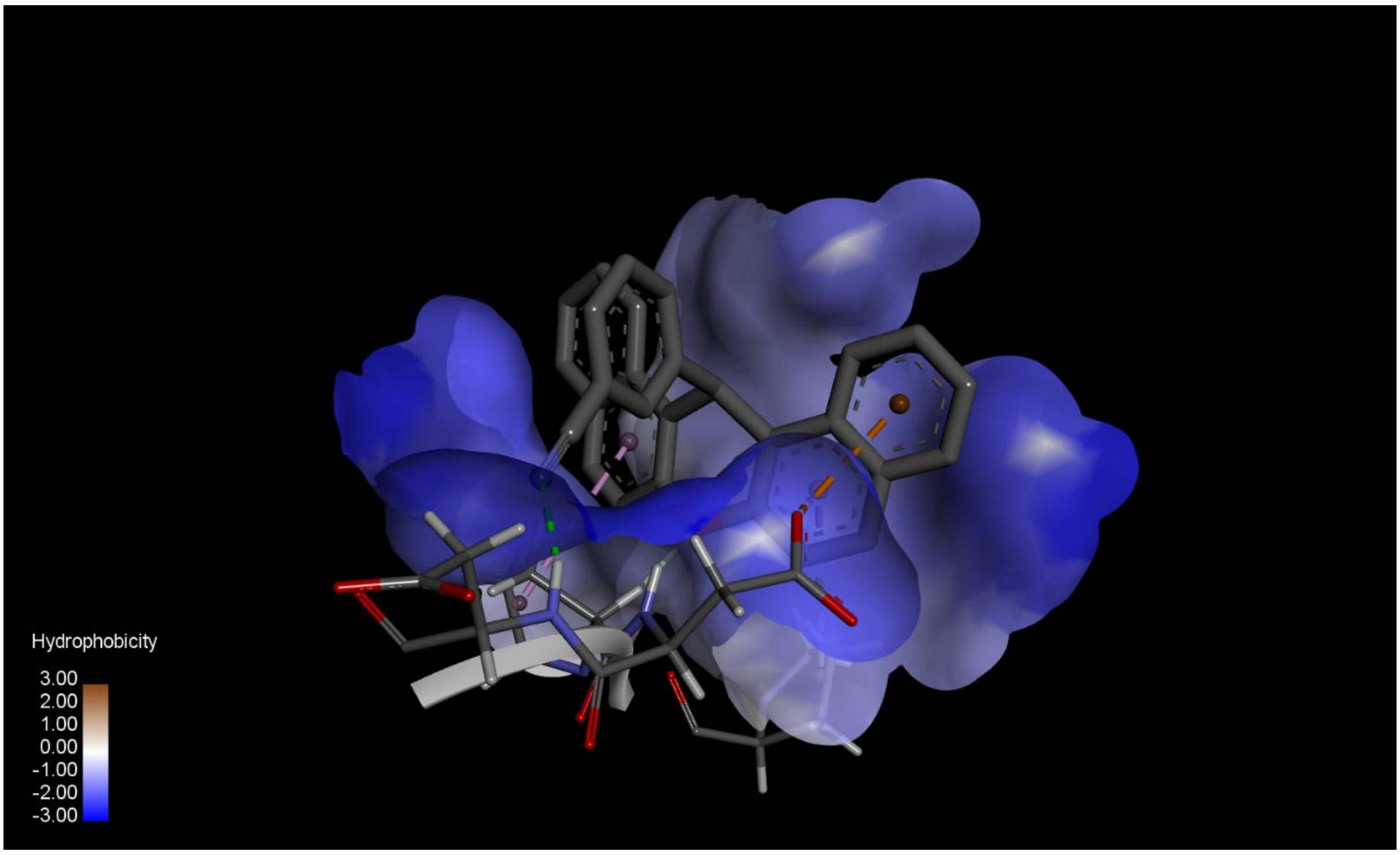

Fig. S142. Compound 6n. Visualization of hydrophobic interaction in the active site

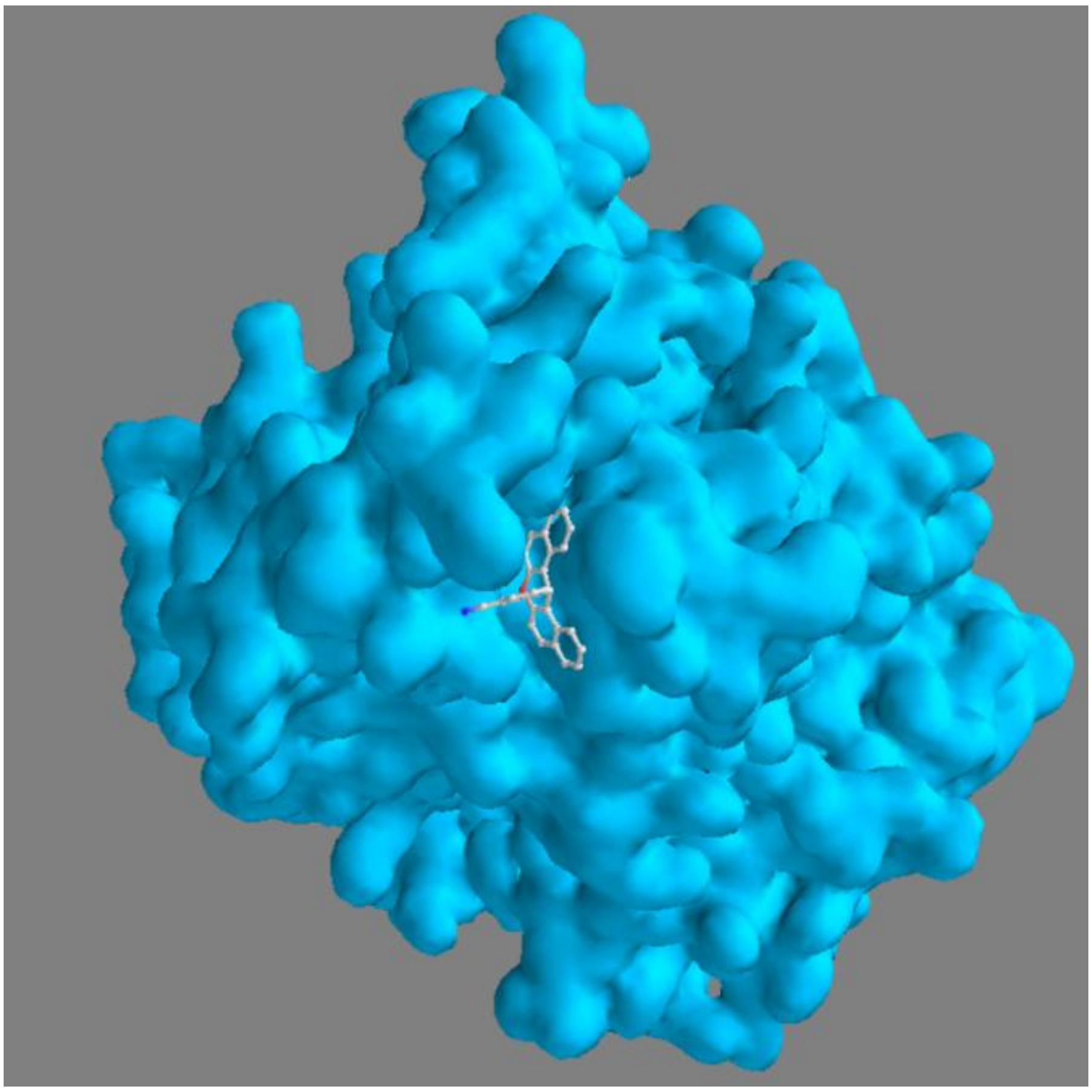

Fig. S143. Compound 6n. Protein surface visualization with bounded ligand

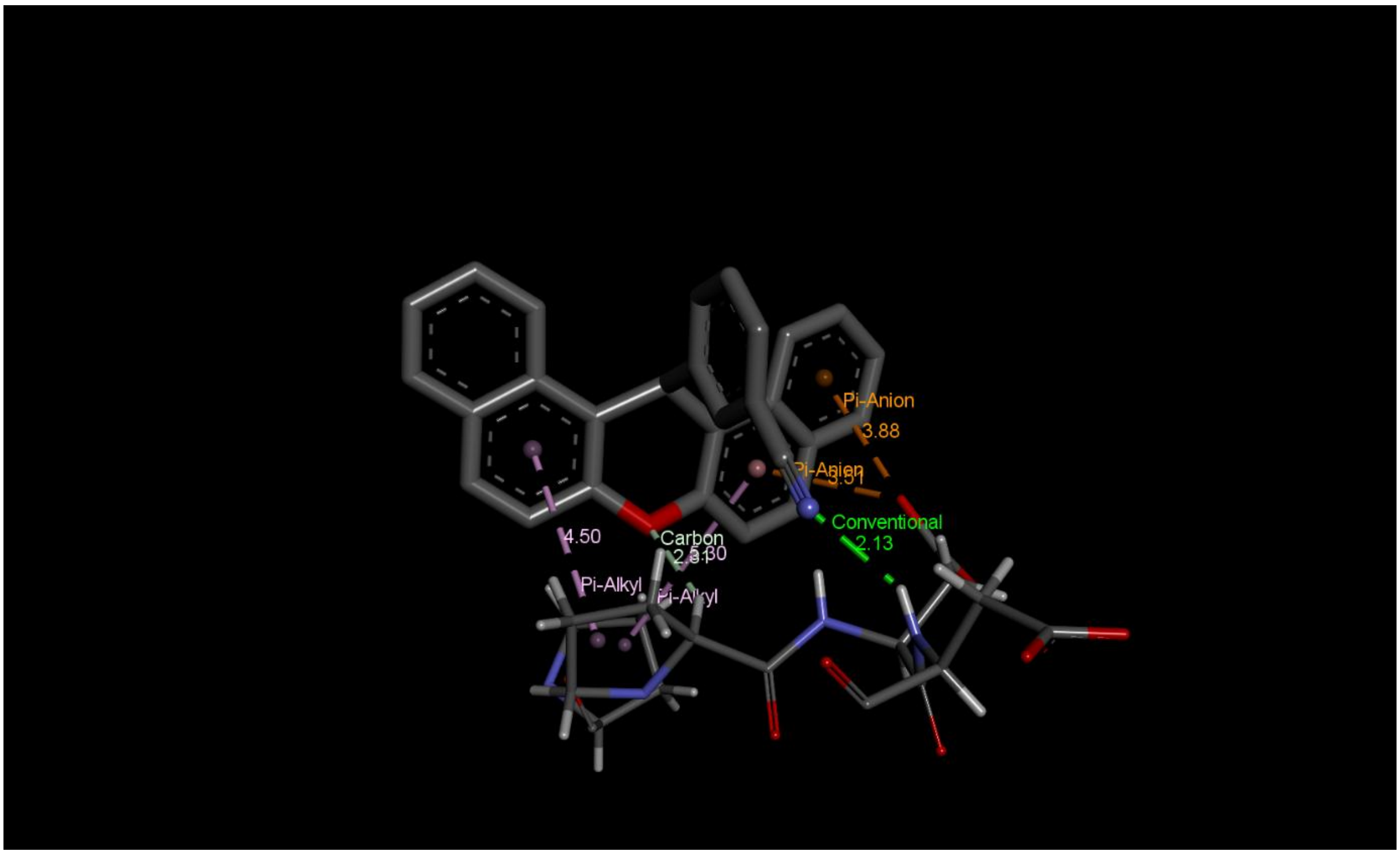

Fig. S144. Compound 6n. Binding pose of the ligand with the active site

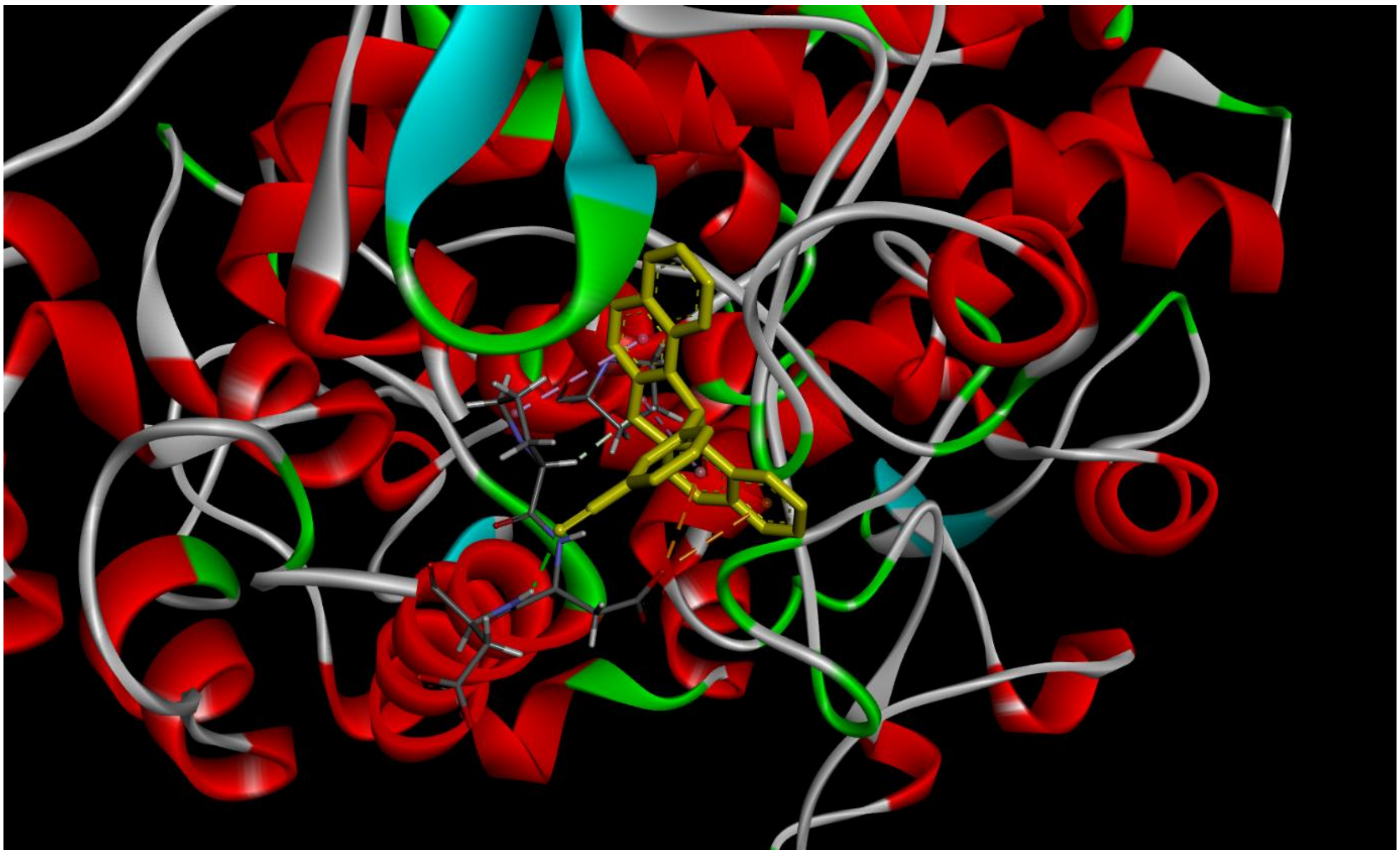

Fig. S145. Compound 6n. Ribbon diagram of the protein-ligand interaction

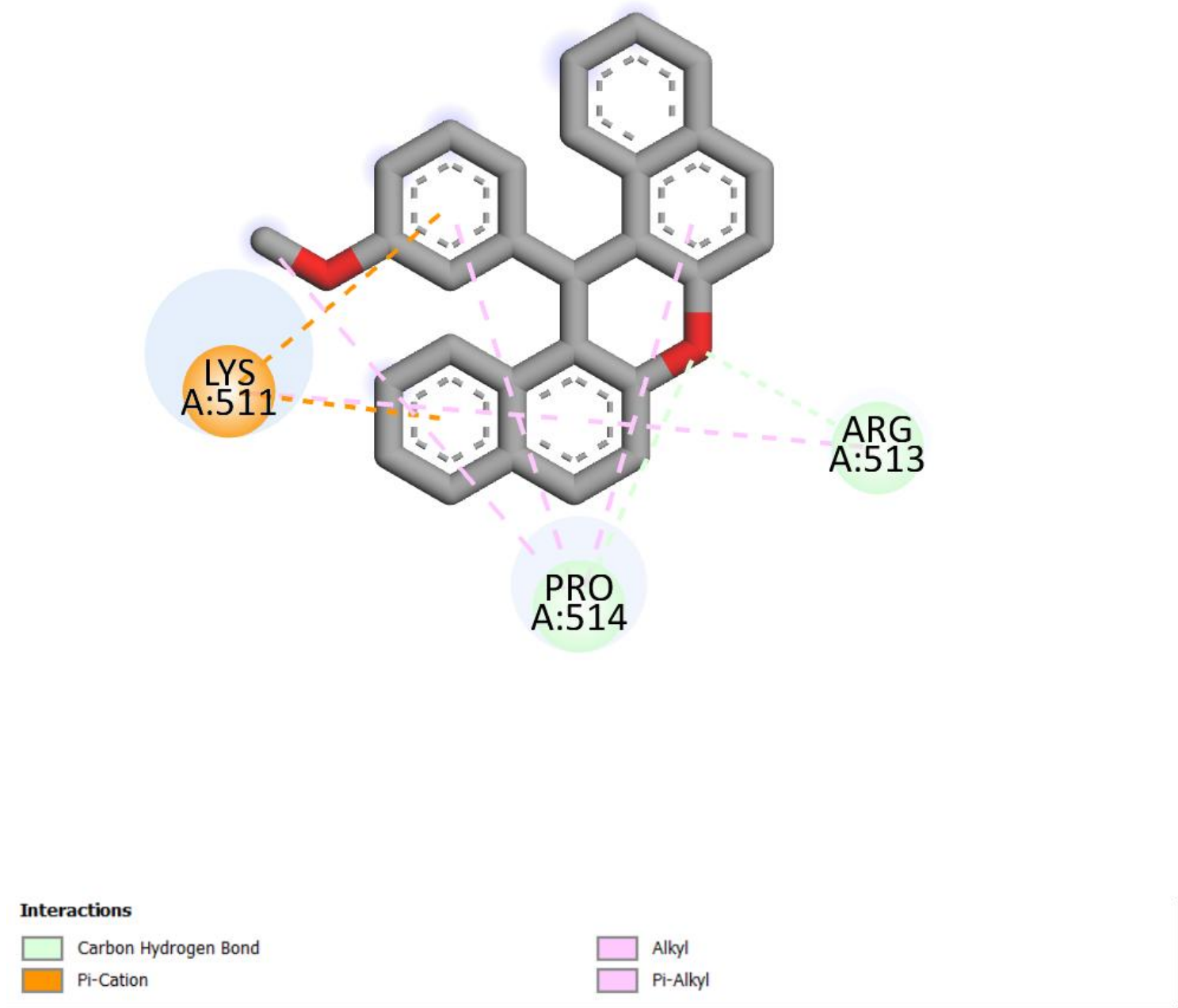

Fig. S146.Compound 6o. 2D-Interaction of ligand-protein

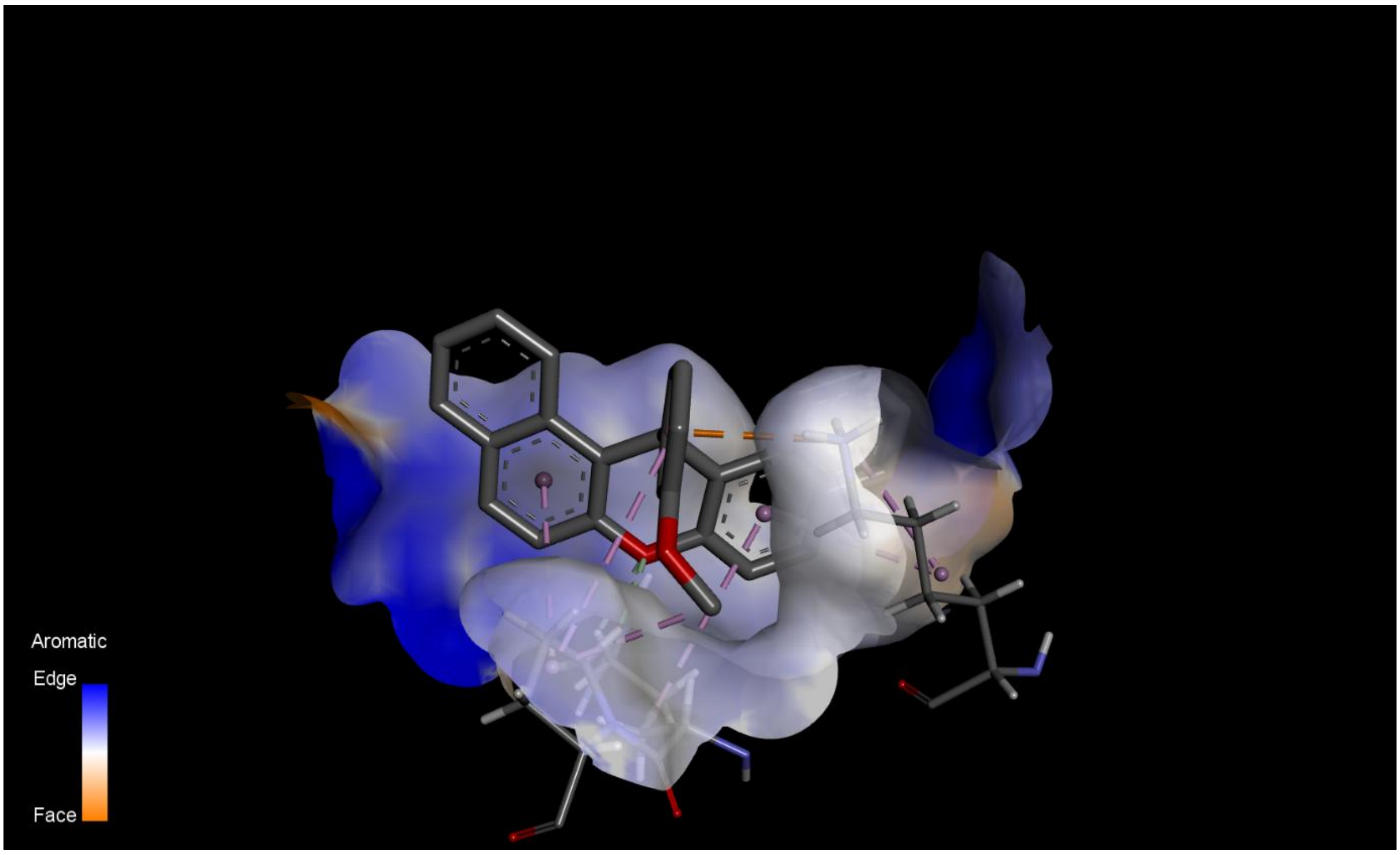

Fig. S147. Compound 6o. Visualization of aromatic face-edge interaction

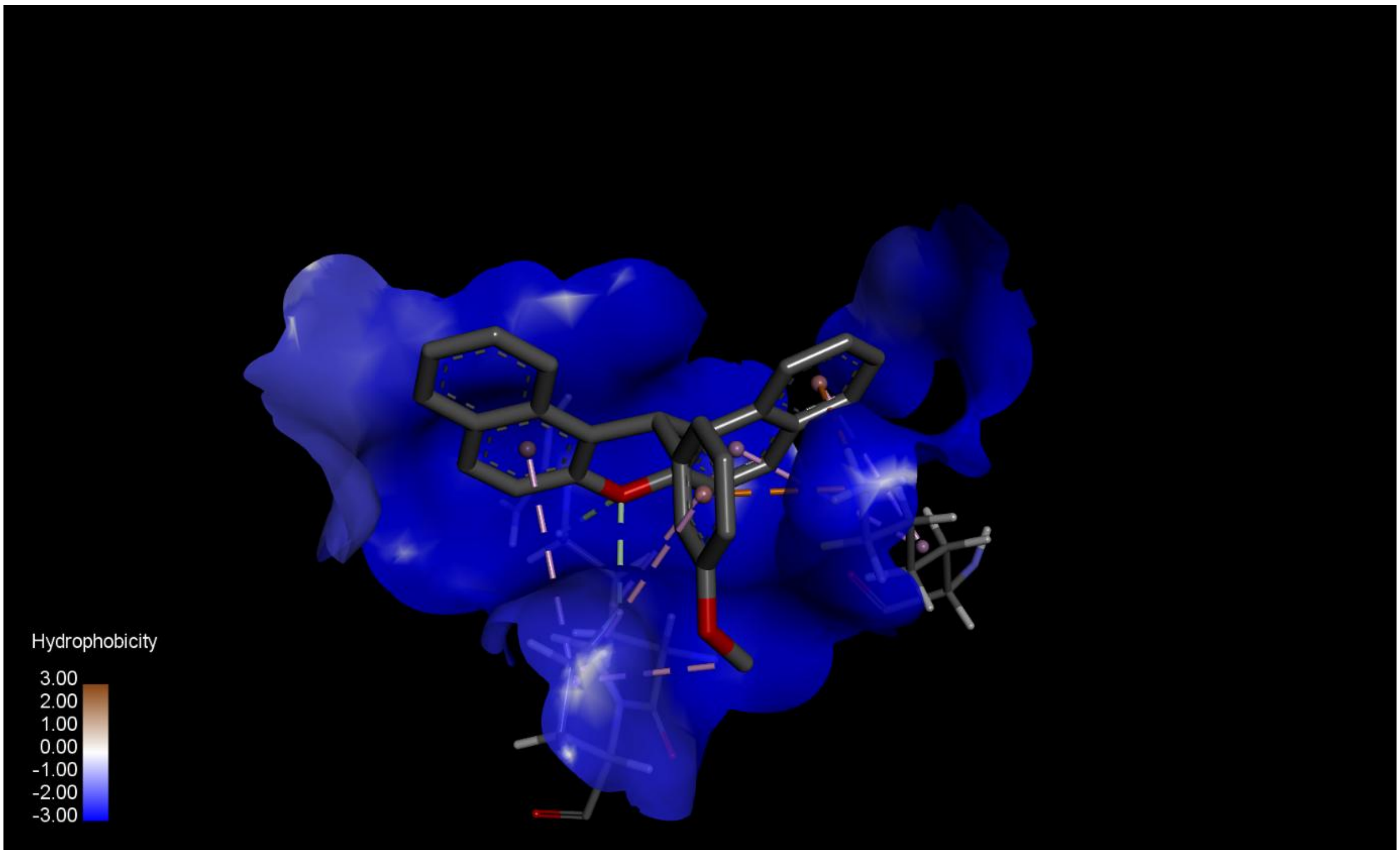

Fig. S148. Compound 6o. Visualization of hydrophobic interaction in the active site

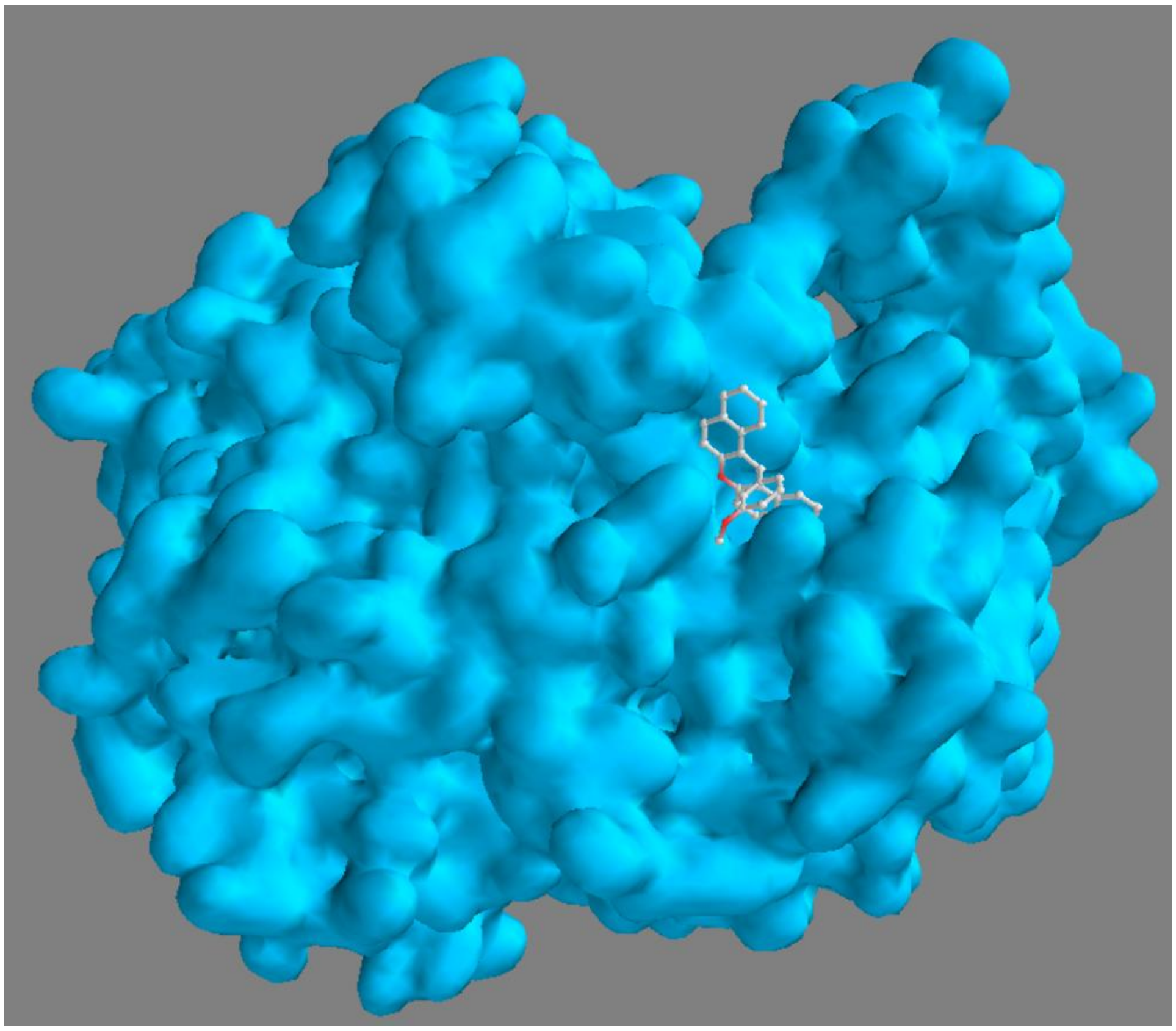

Fig. S149. Compound 6o. Protein surface visualization with bounded ligand

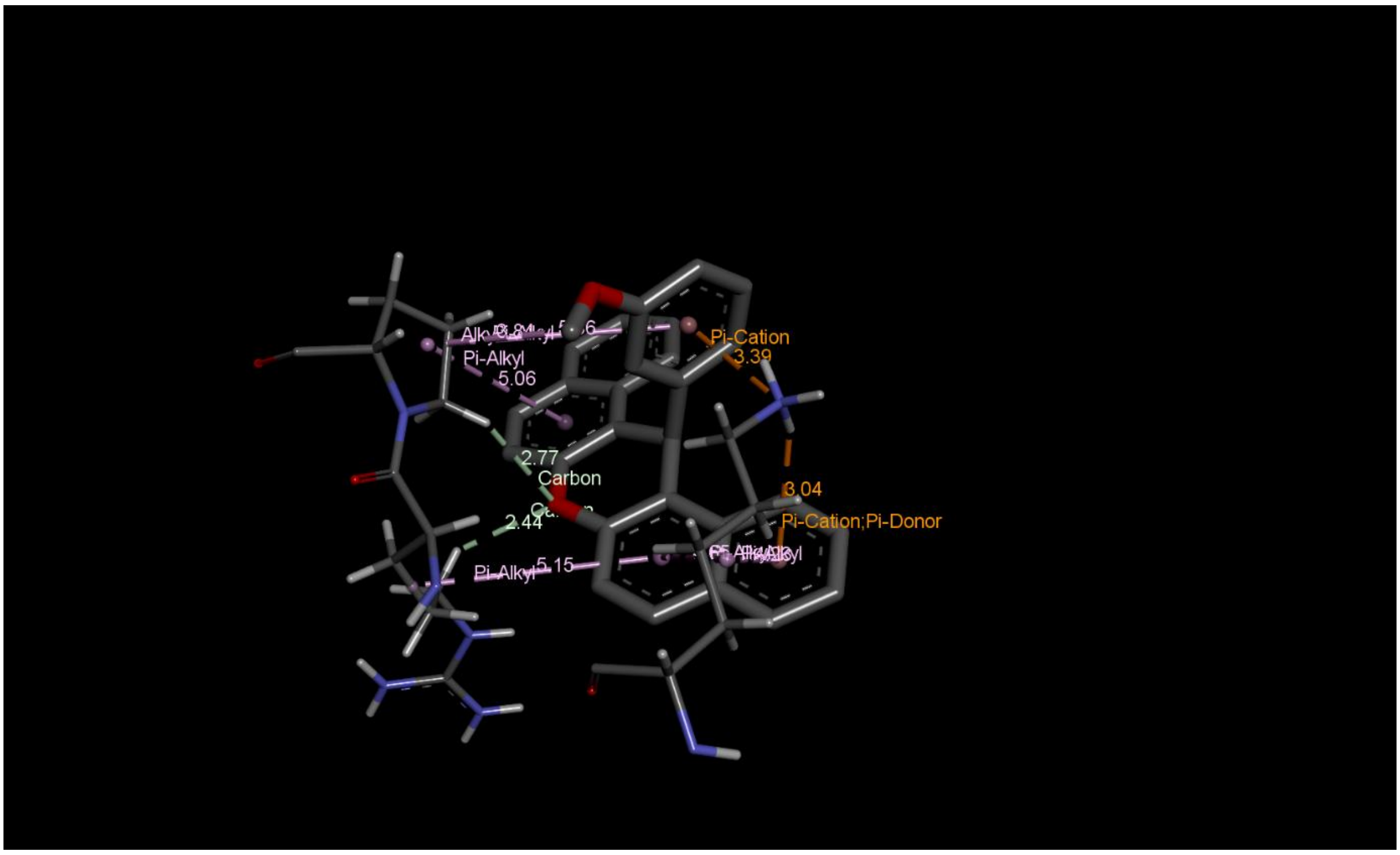

Fig. S150. Compound 6o. Binding pose of the ligand with the active site

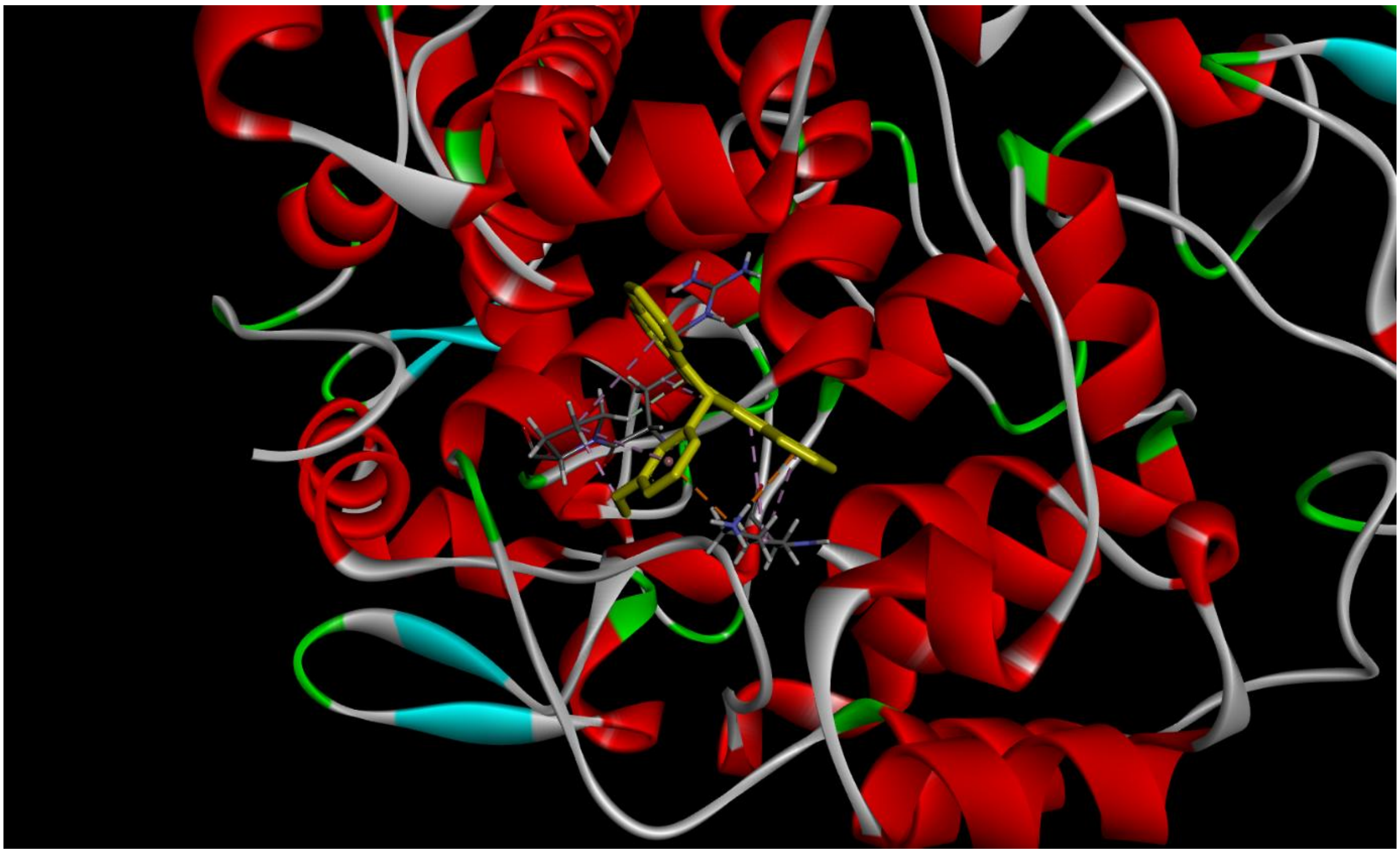

Fig. S151. Compound 6o. Ribbon diagram of the protein-ligand interaction

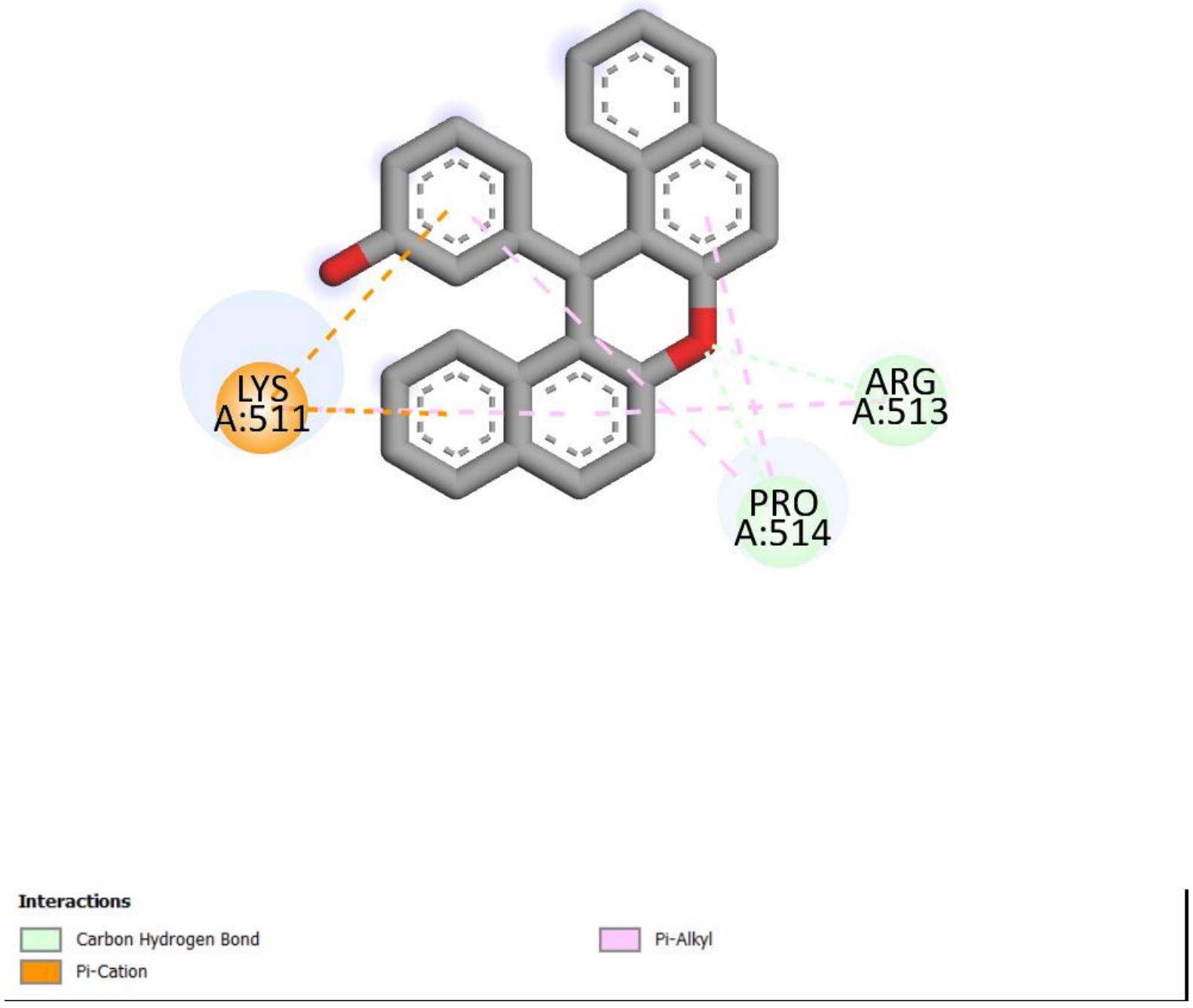

Fig. S152. Compound 6p. 2D-Interaction of ligand-protein

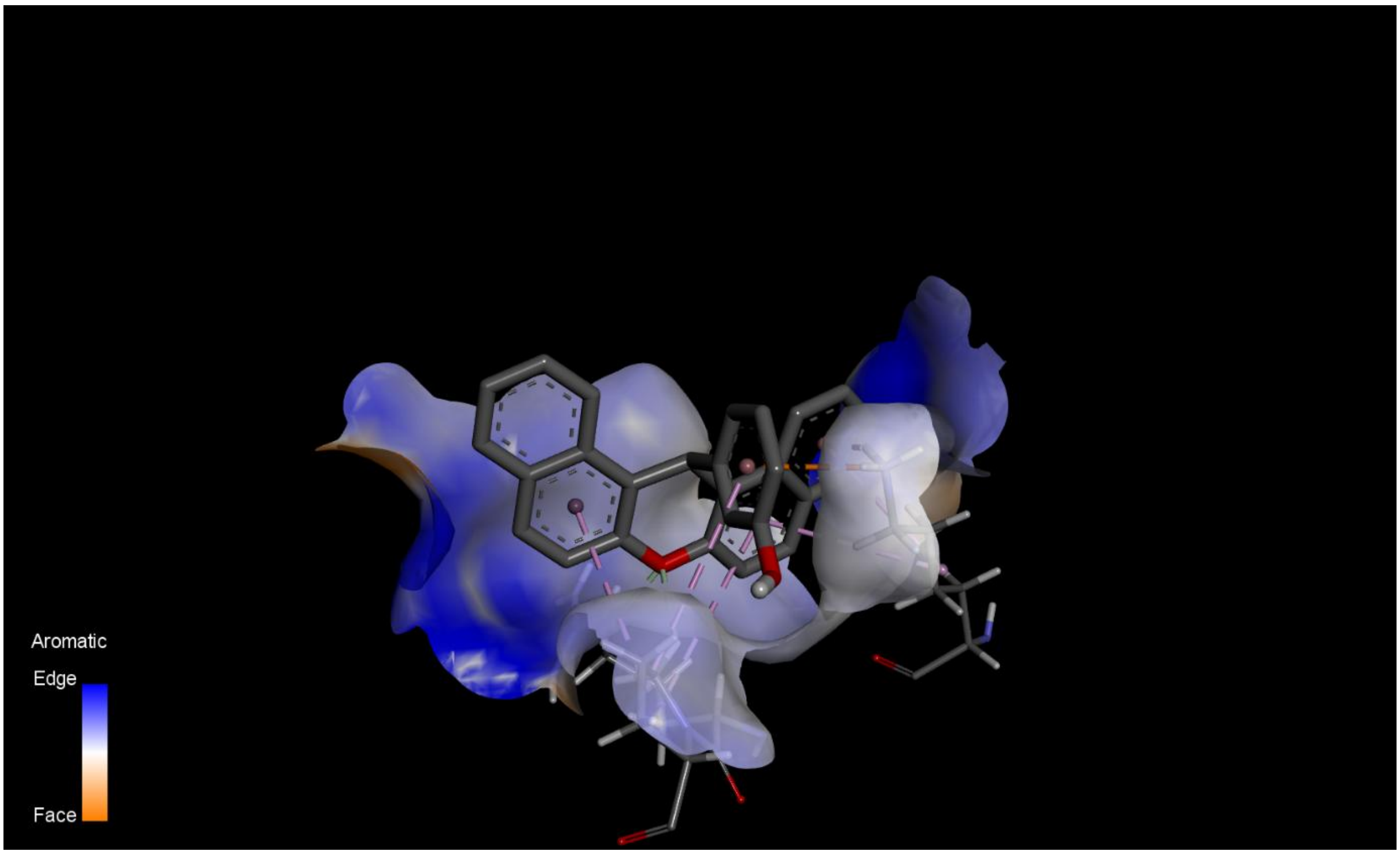

Fig. S153. Compound 6p. Visualization of aromatic face-edge interaction

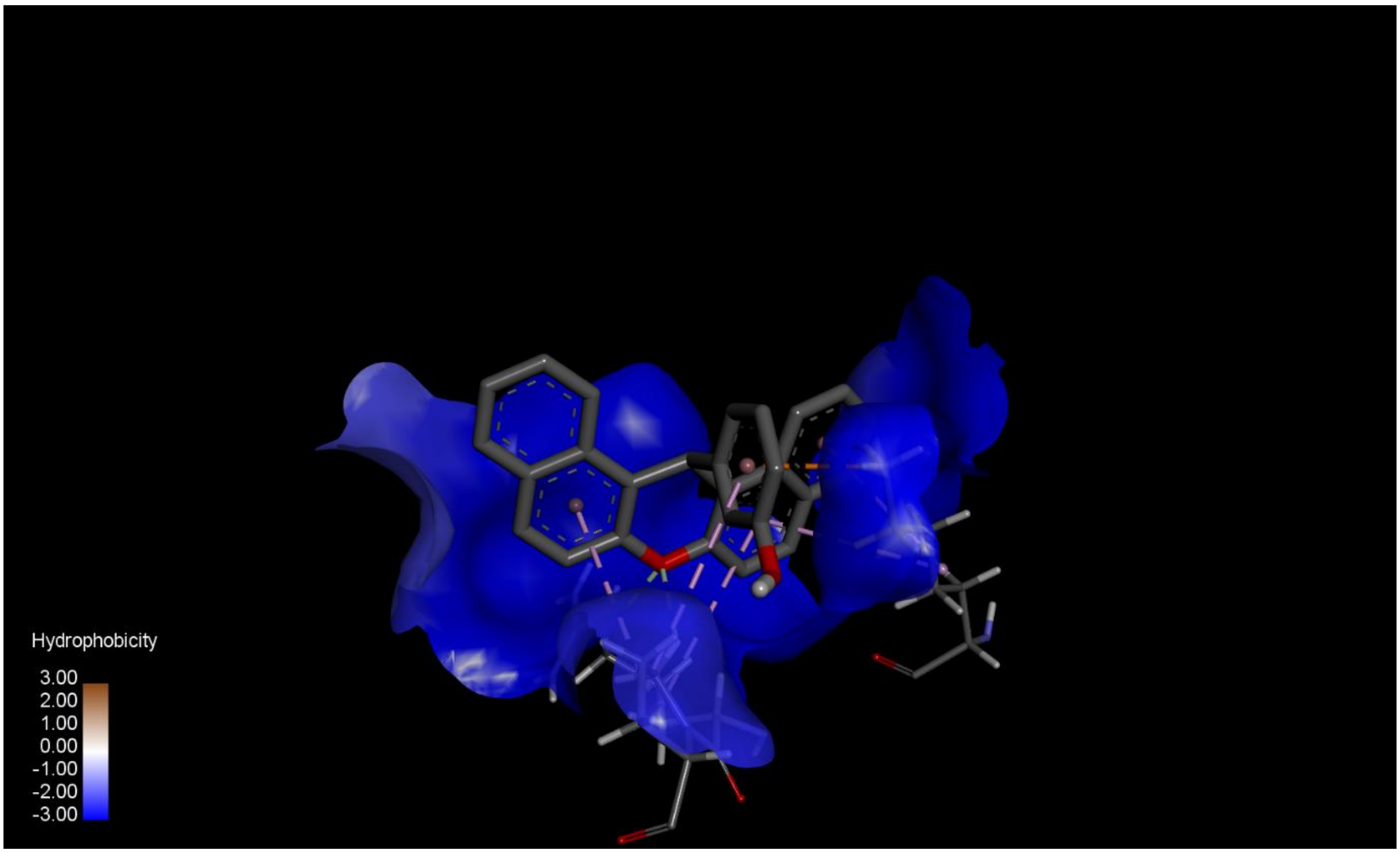

Fig. S154. Compound 6p. Visualization of hydrophobic interaction in the active site

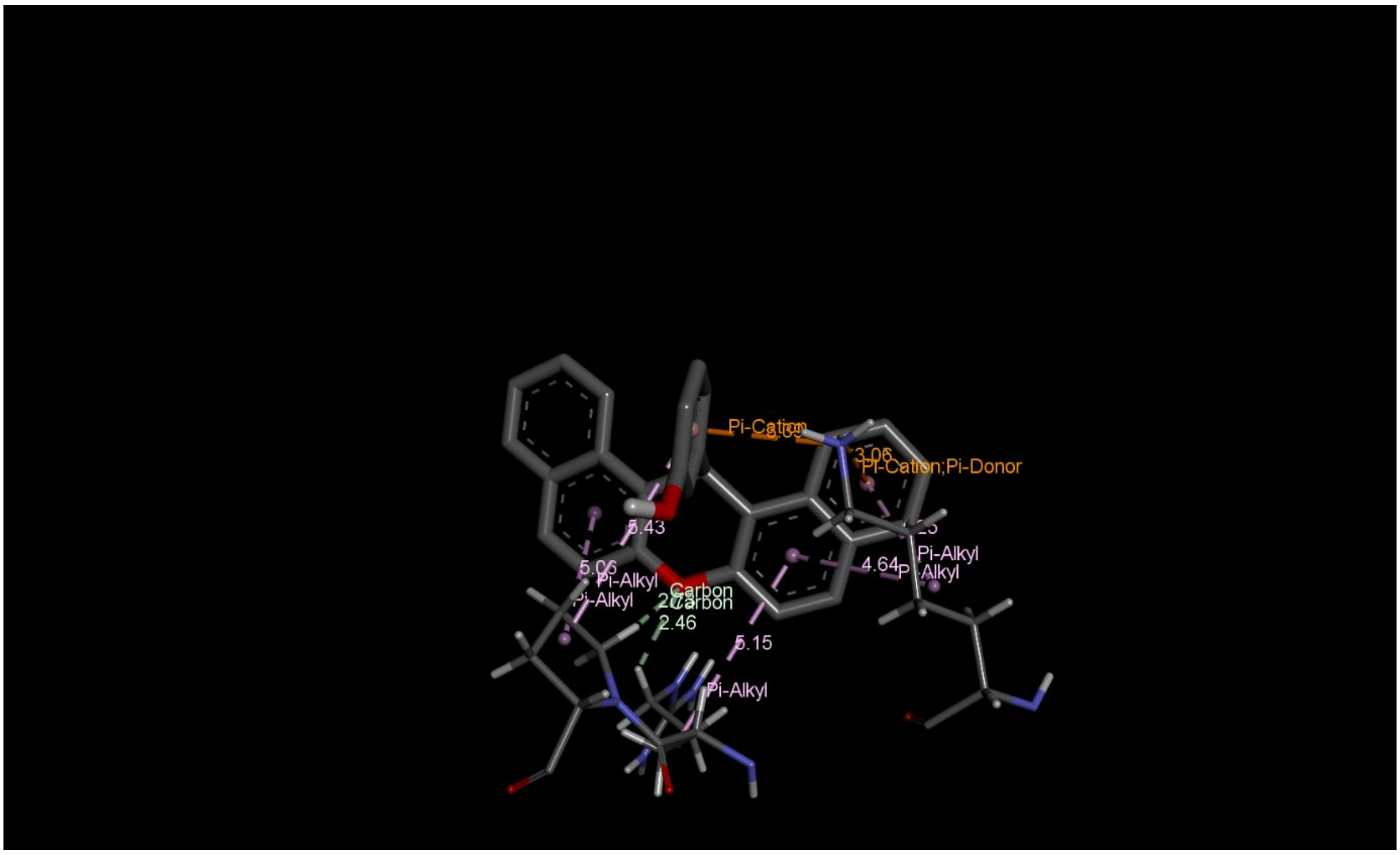

Fig. S155. Compound 6p. Binding pose of the ligand with the active site

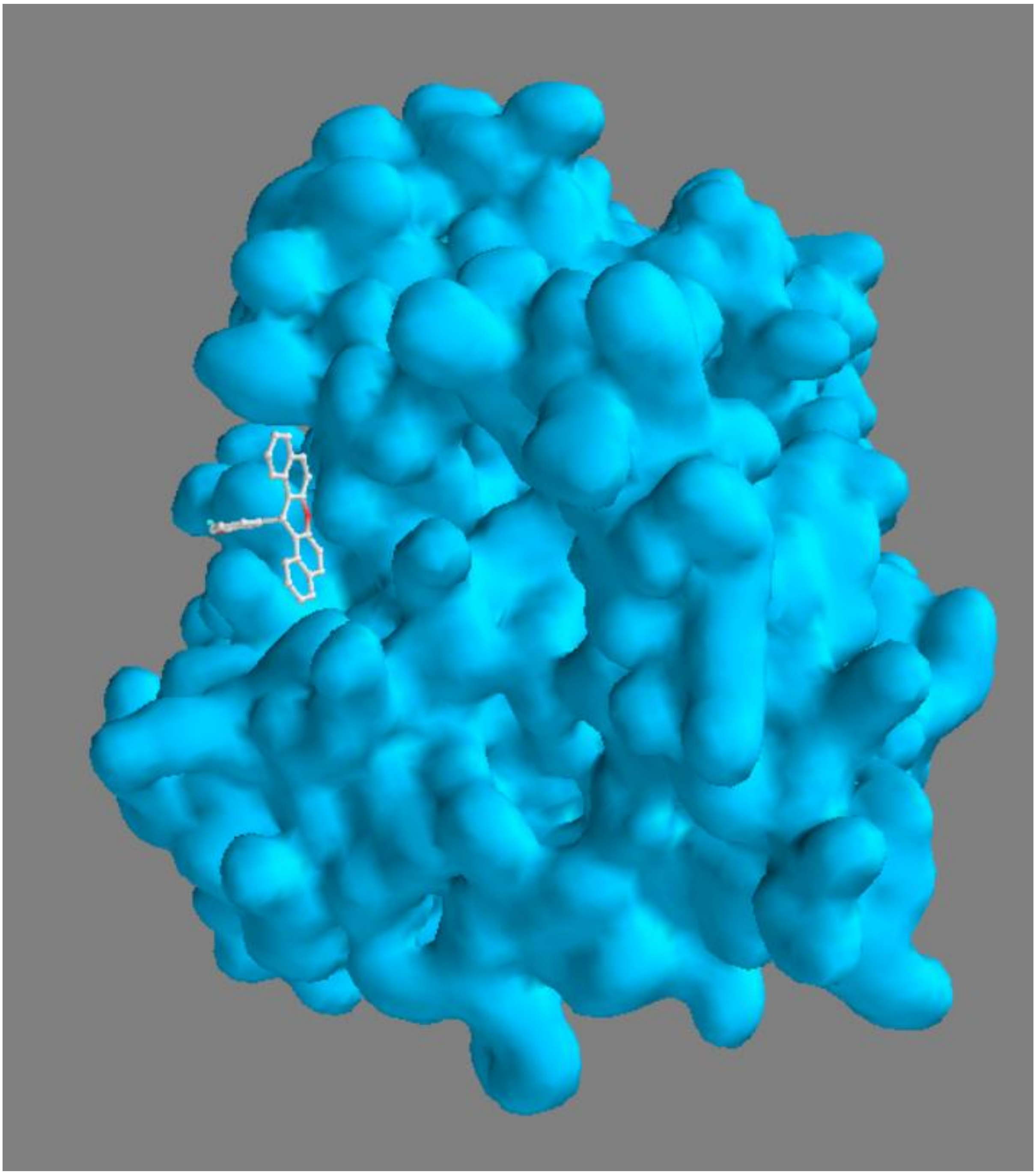

Fig. S156. Compound 6p. Protein surface visualization with bounded ligand

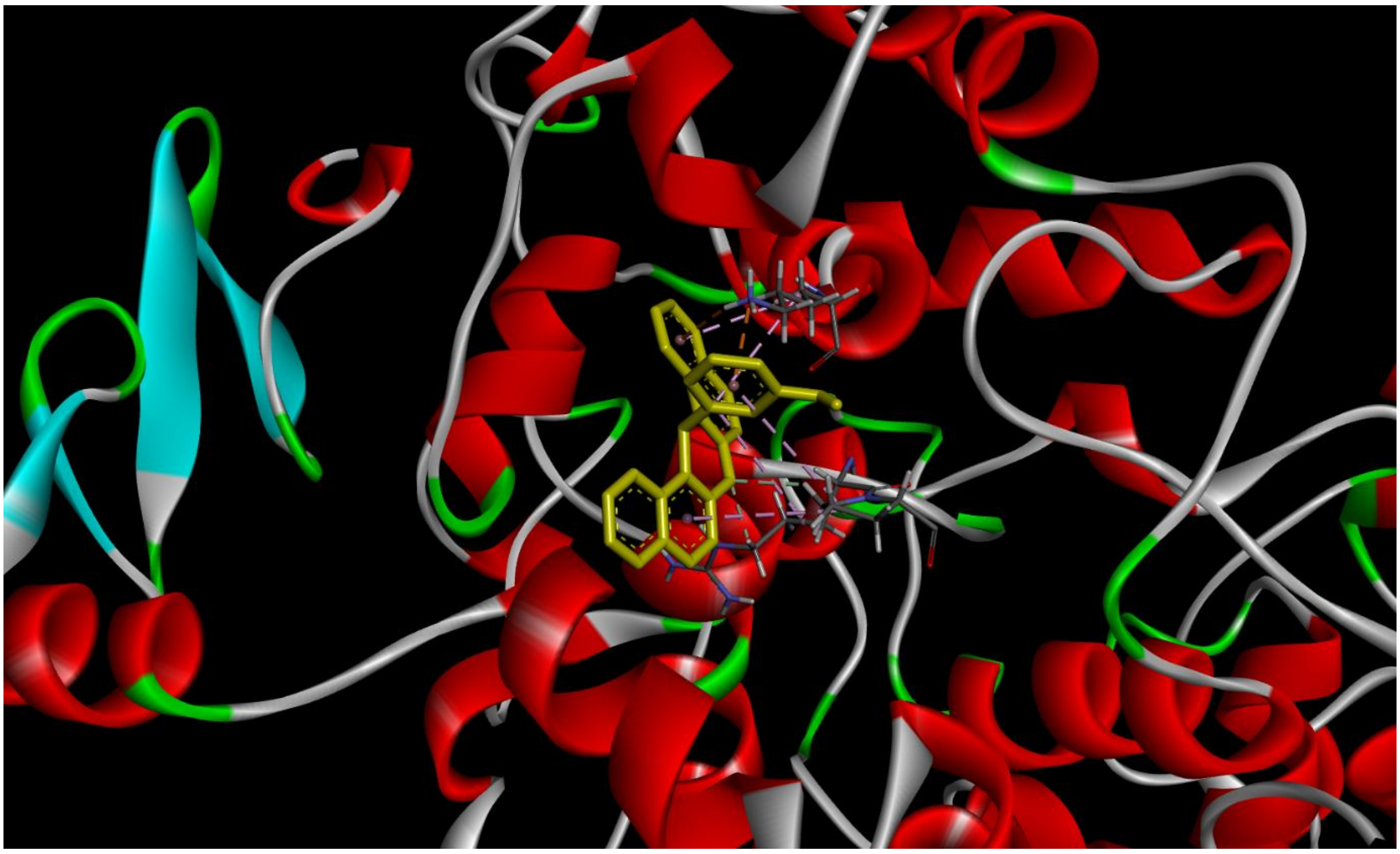

Fig. S157. Compound 6p. Ribbon diagram of the protein-ligand interaction

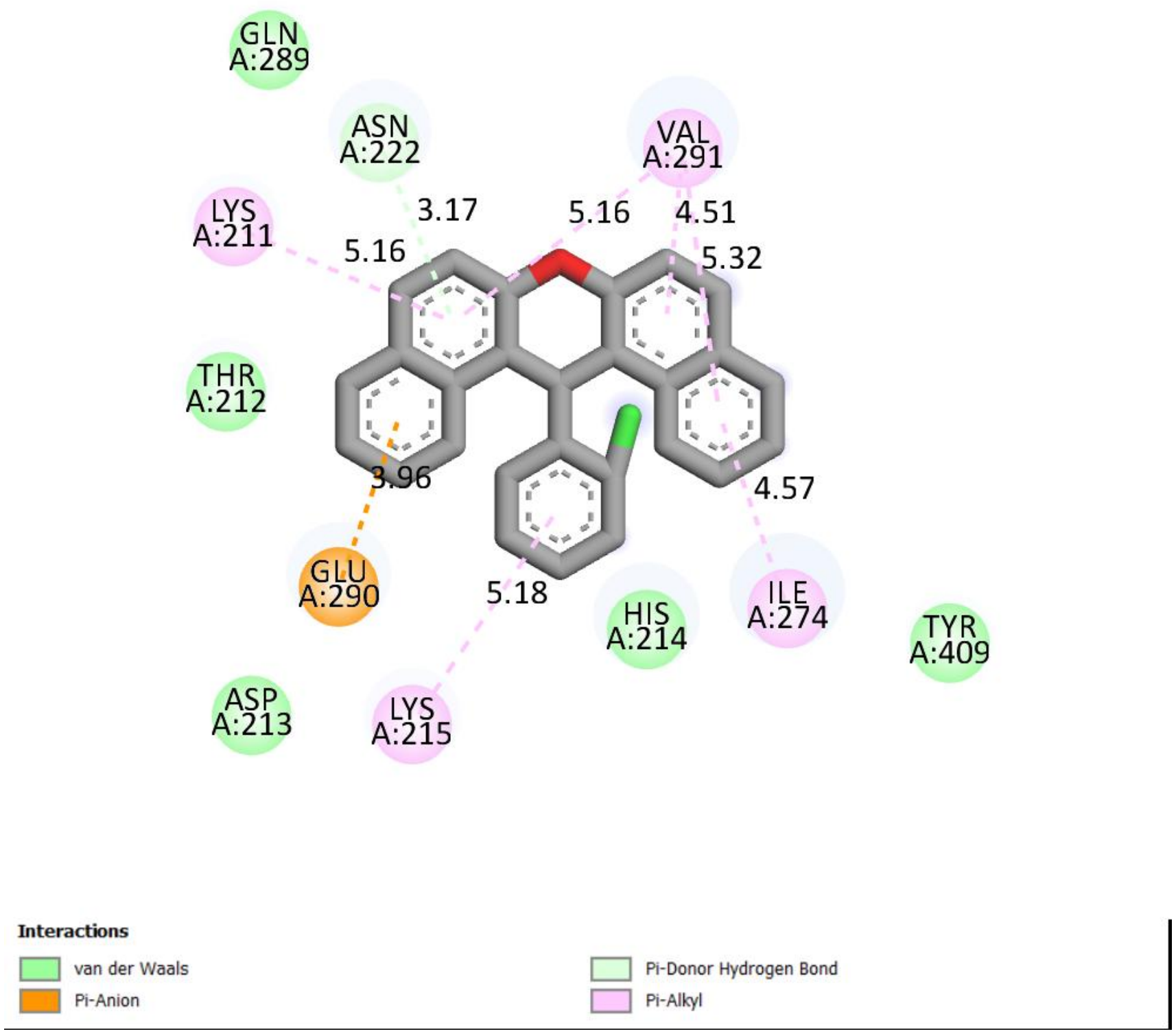

Fig. S158. Compound 6q. 2D-Interaction of ligand-protein

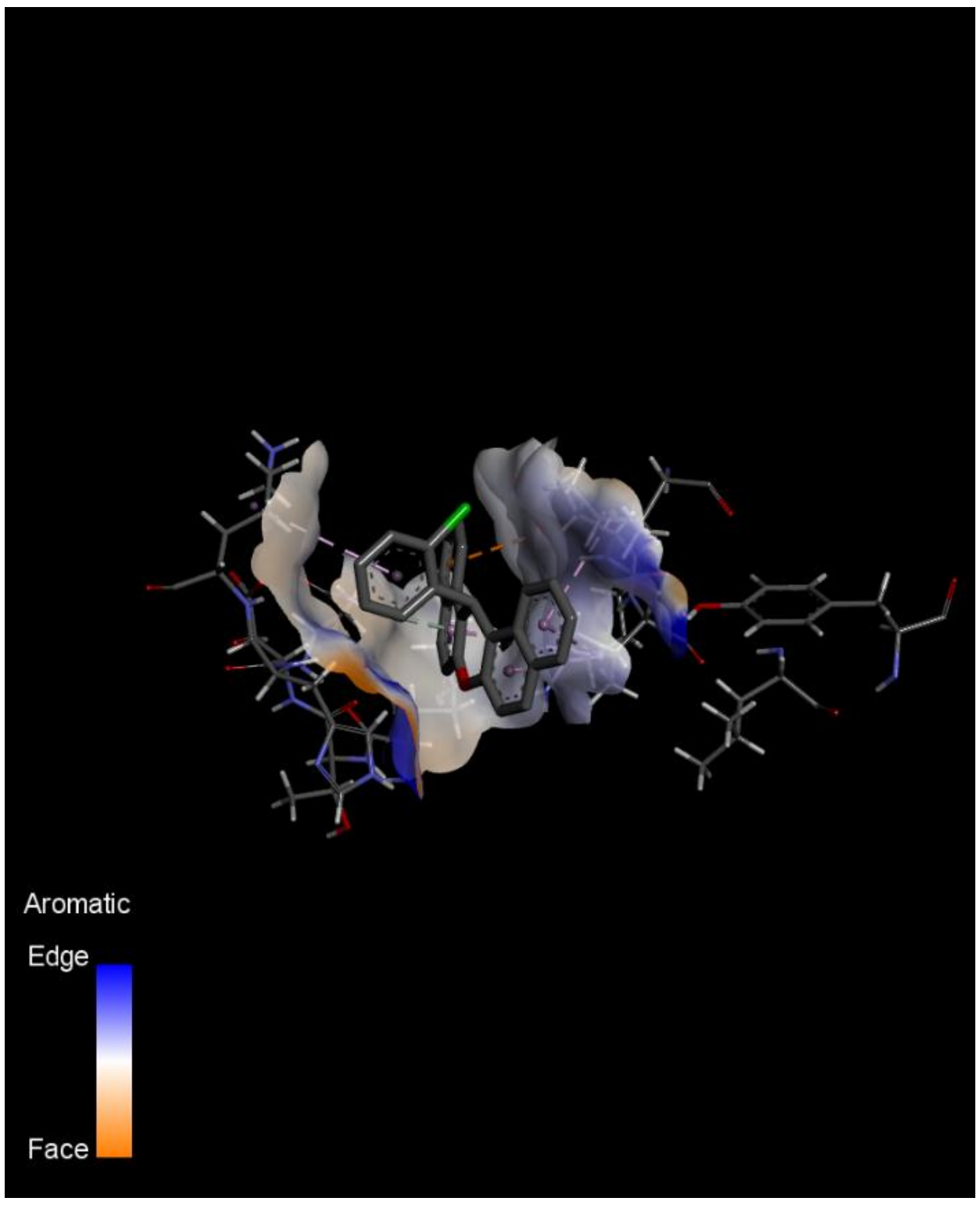

Fig. S159. Compound 6q. Visualization of aromatic face-edge interaction

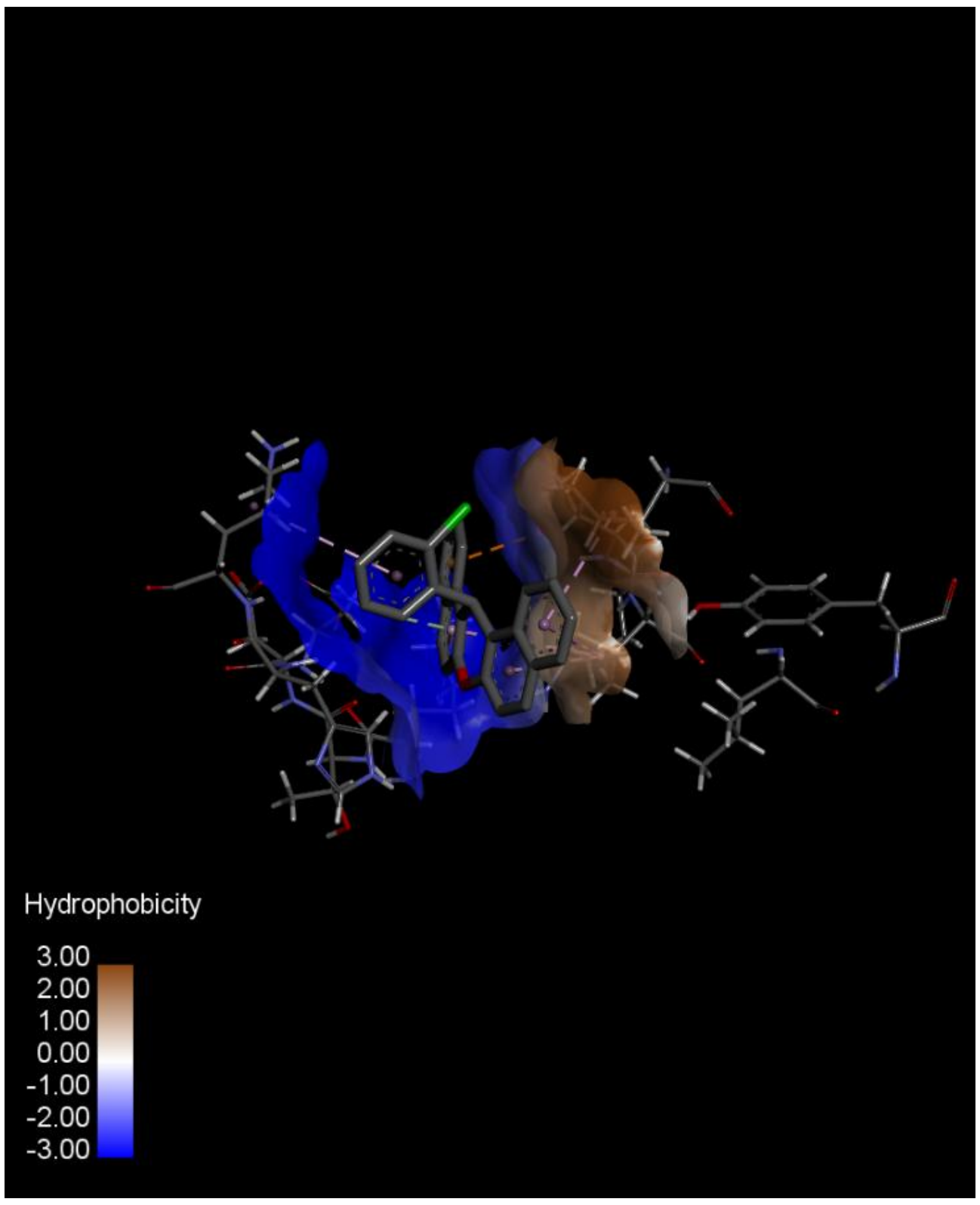

Fig. S160. Compound 6q. Visualization of hydrophobic interaction in the active site

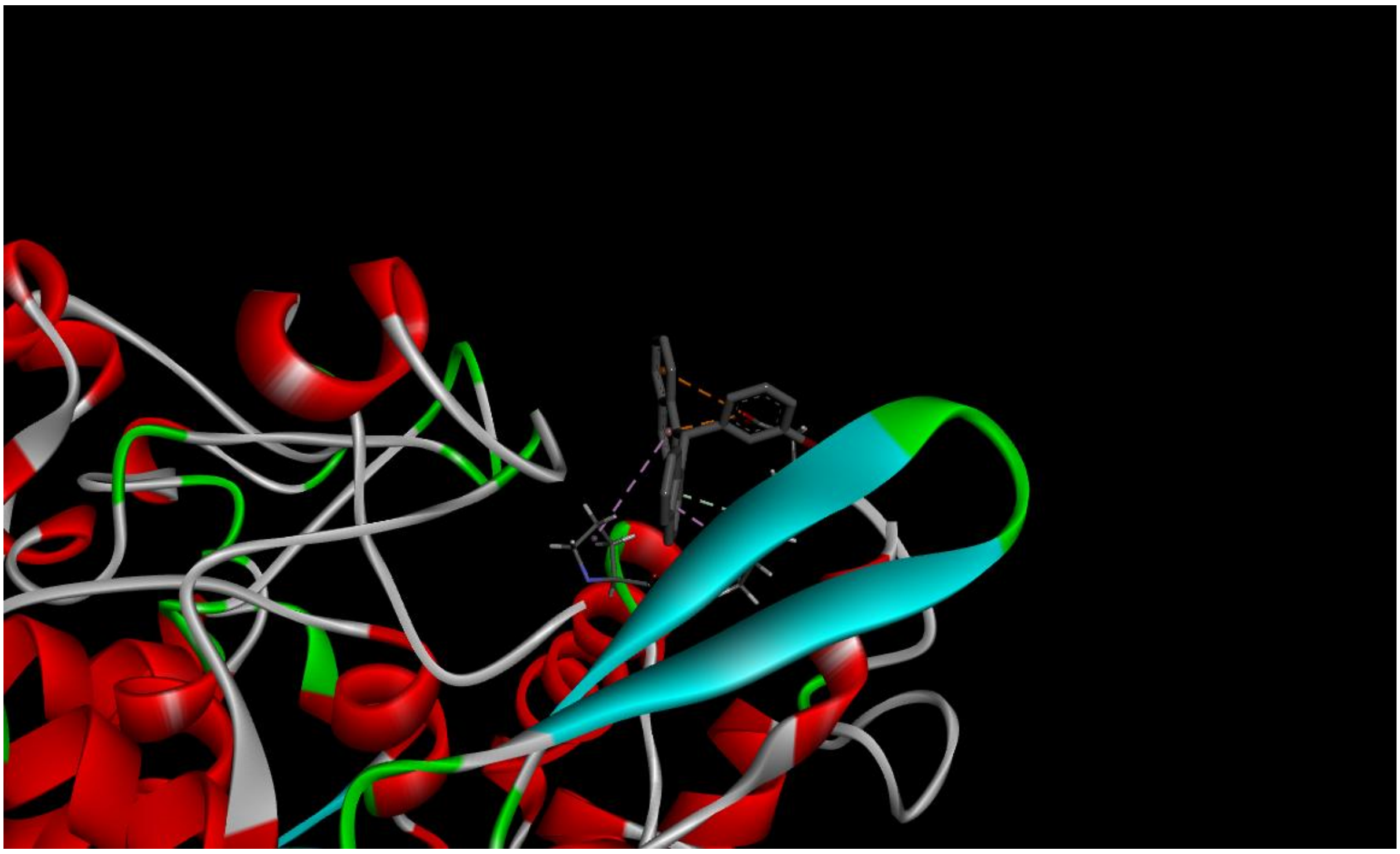

Fig. S161: Compound 6q. Ribbon diagram of the protein-ligand interaction

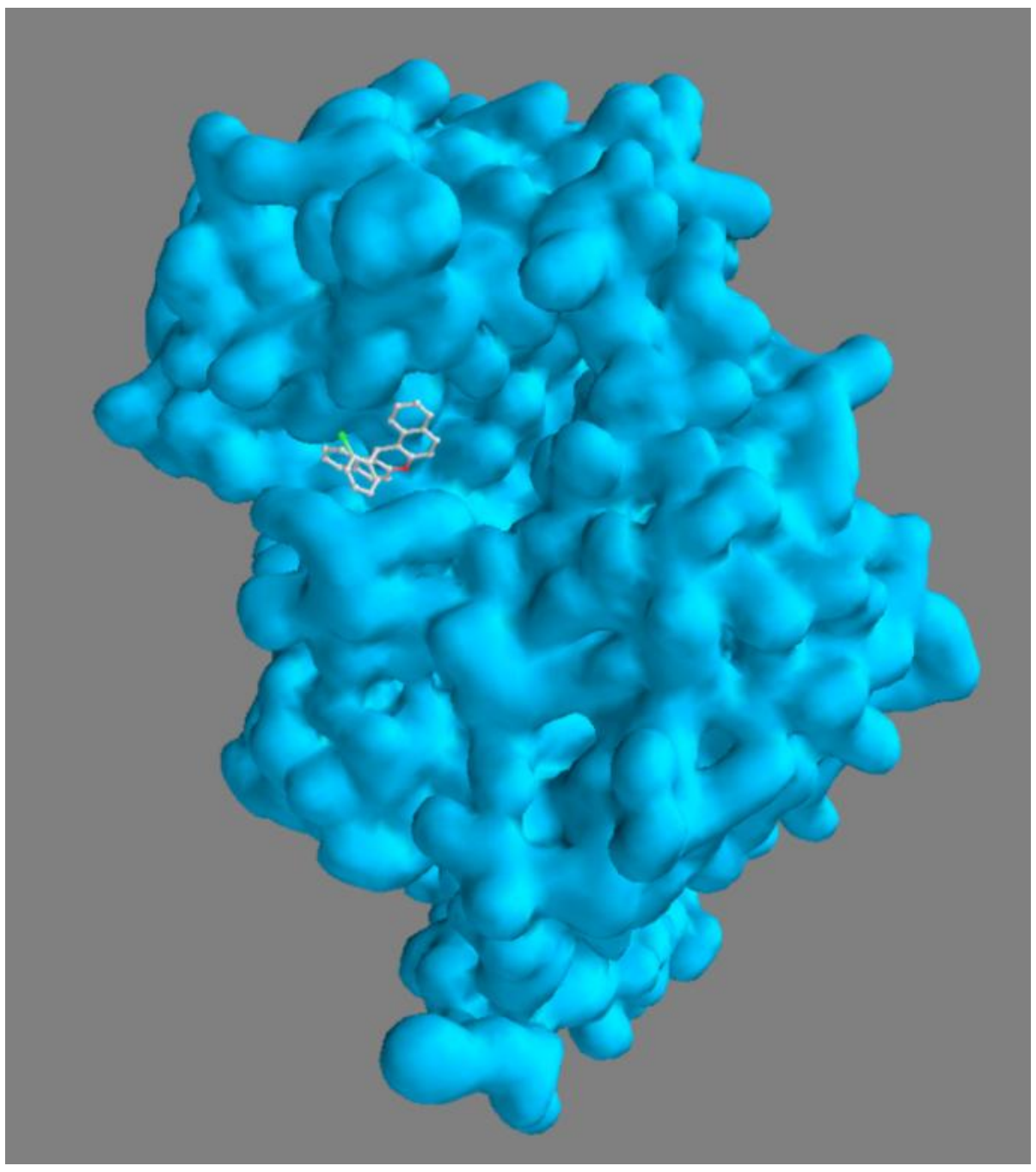

Fig. S162. Compound 6q. Protein surface visualization with bounded ligand

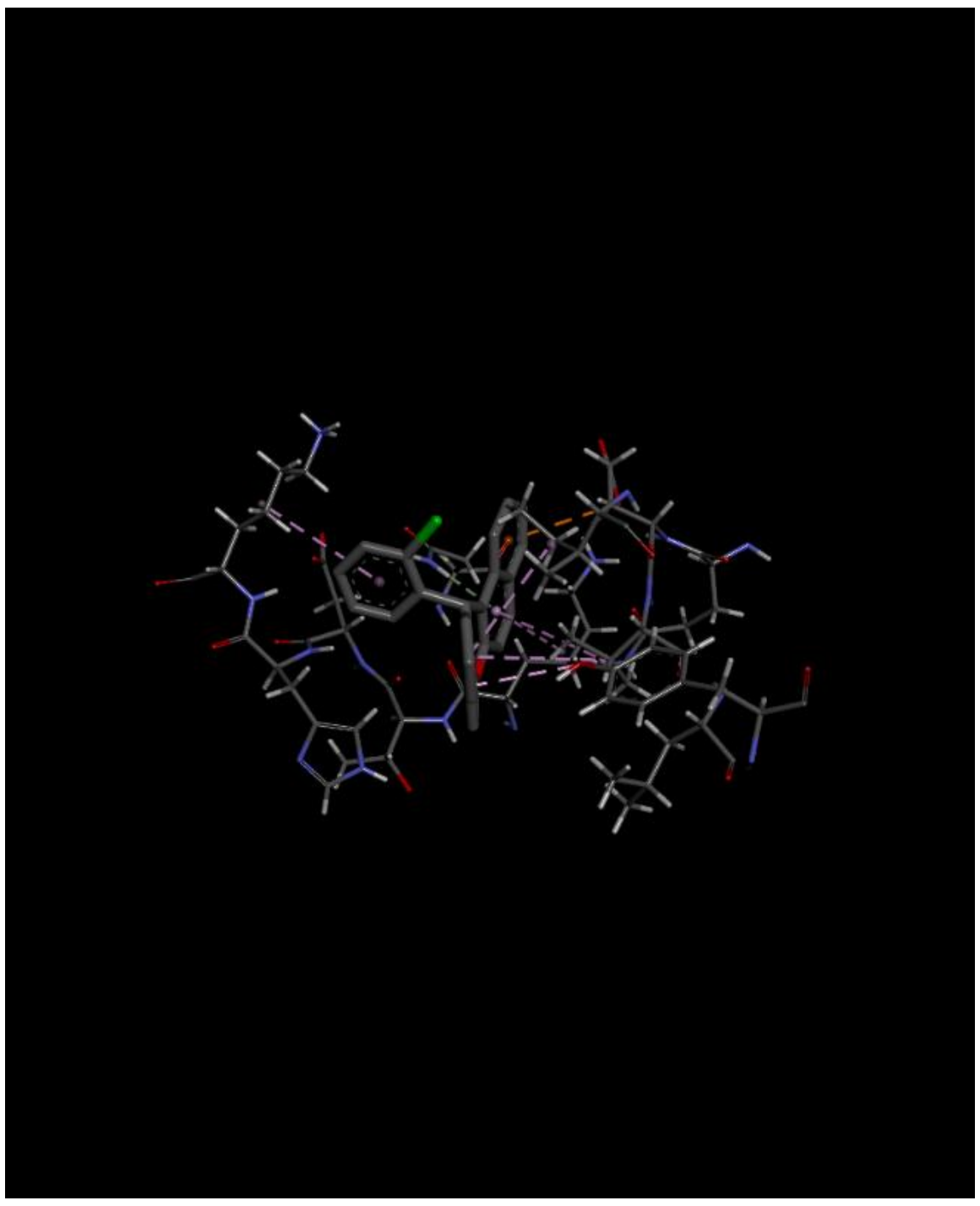

Fig. S163. Compound 6q. Binding pose of the ligand with the active site

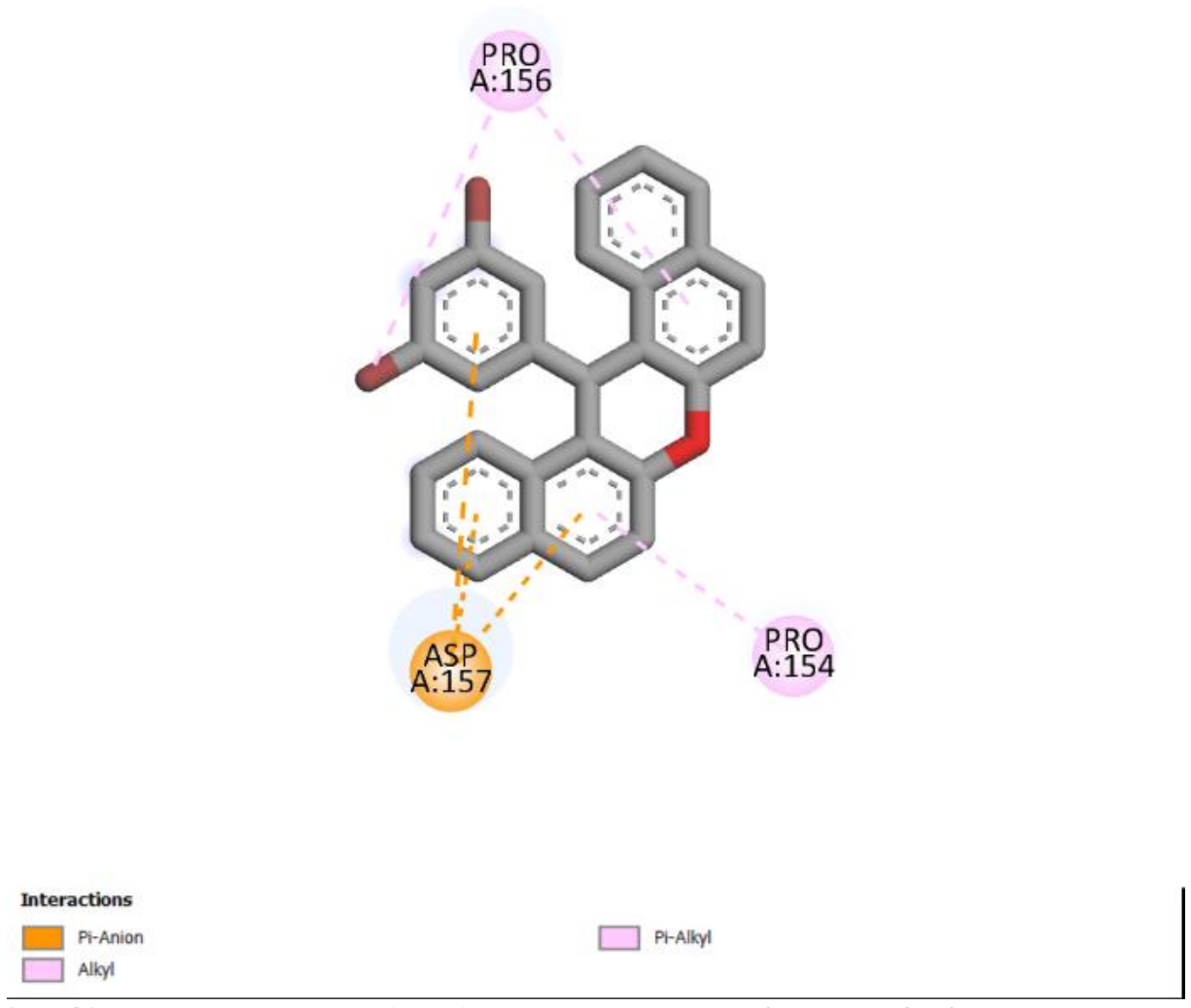

Fig. S164. Compound 6r. 2D-Interaction of ligand-protein

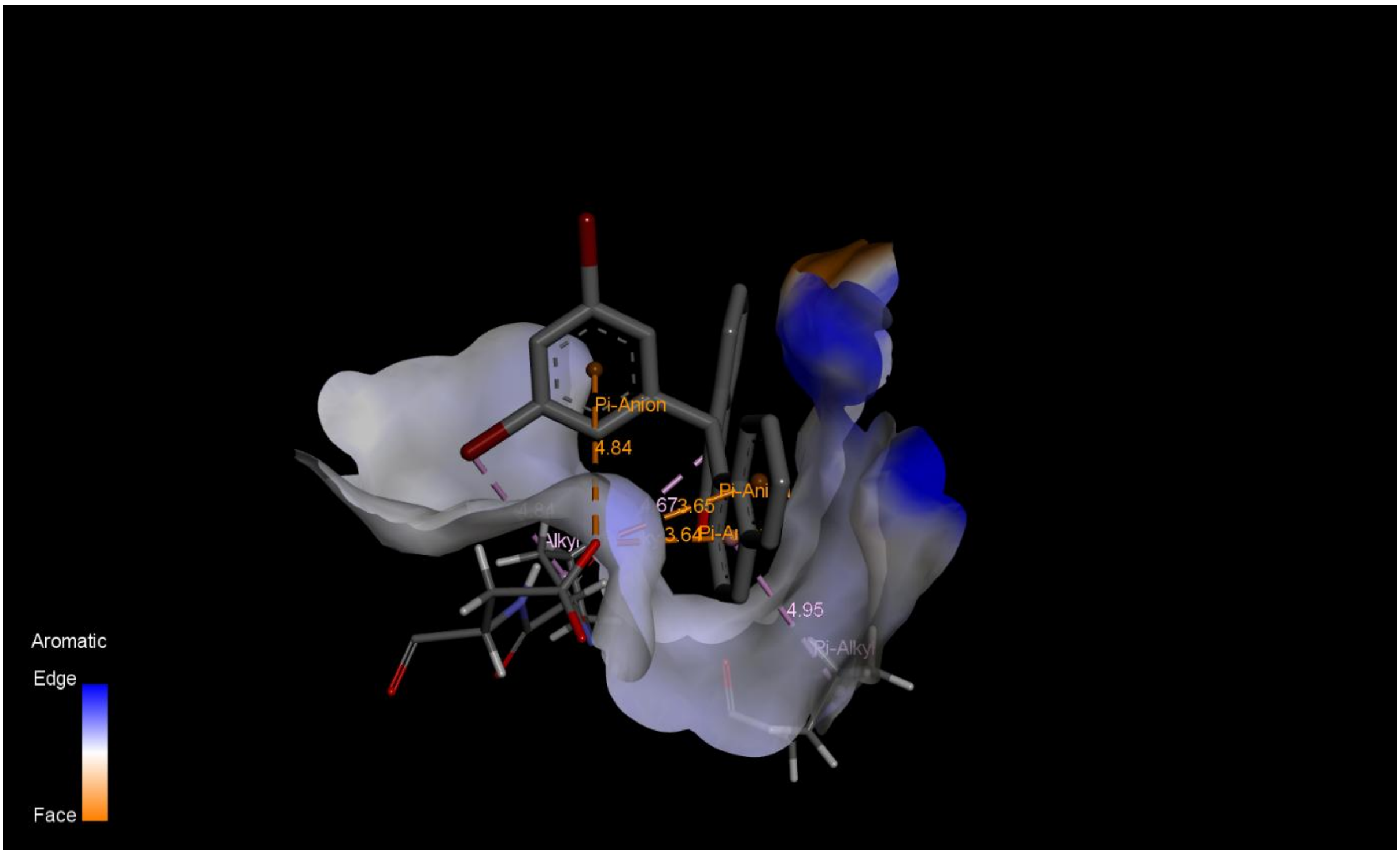

Fig. S165. Compound 6r. Visualization of aromatic face-edge interaction

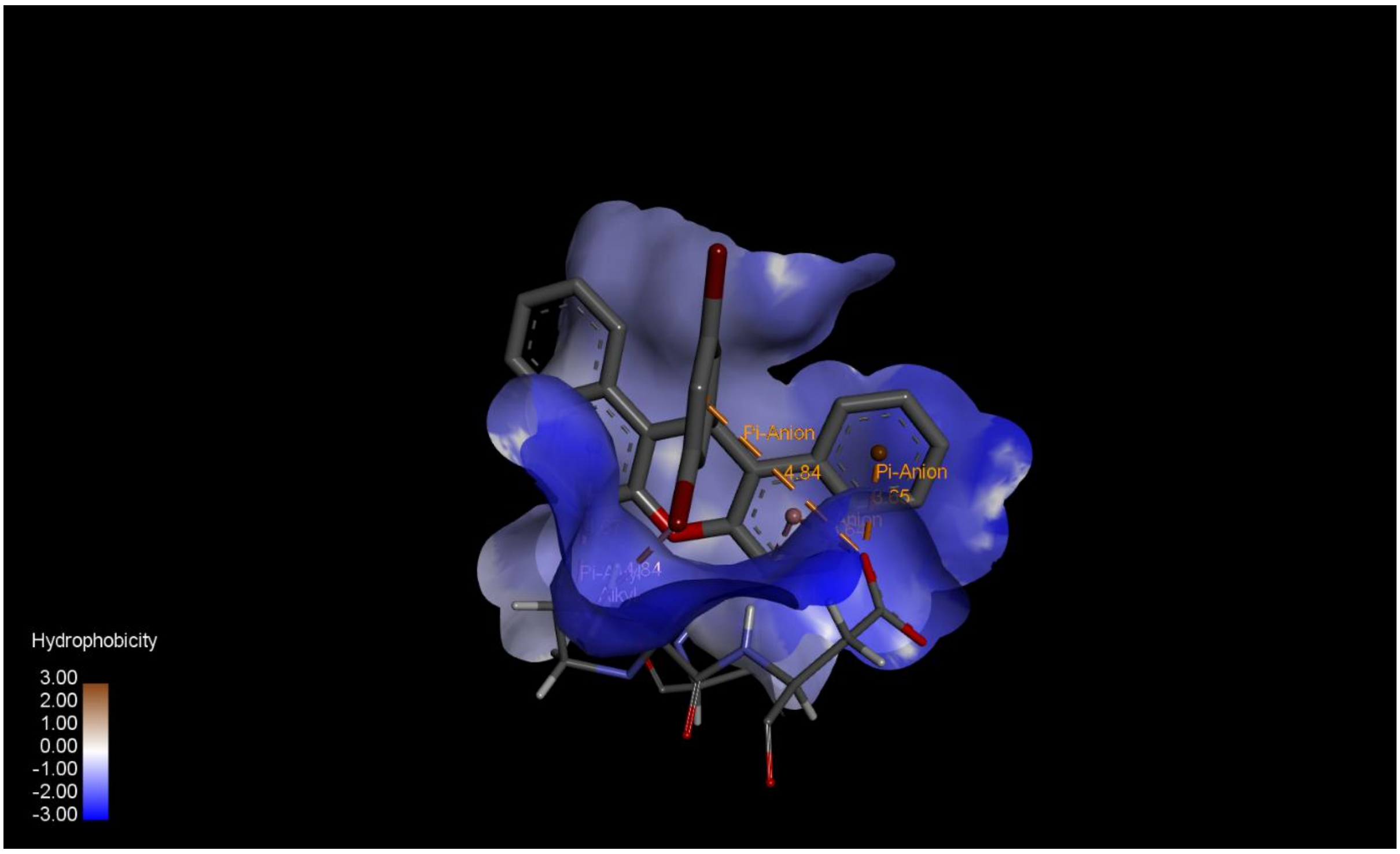

Fig. S166. Compound 6r. Visualization of hydrophobic interaction in the active site

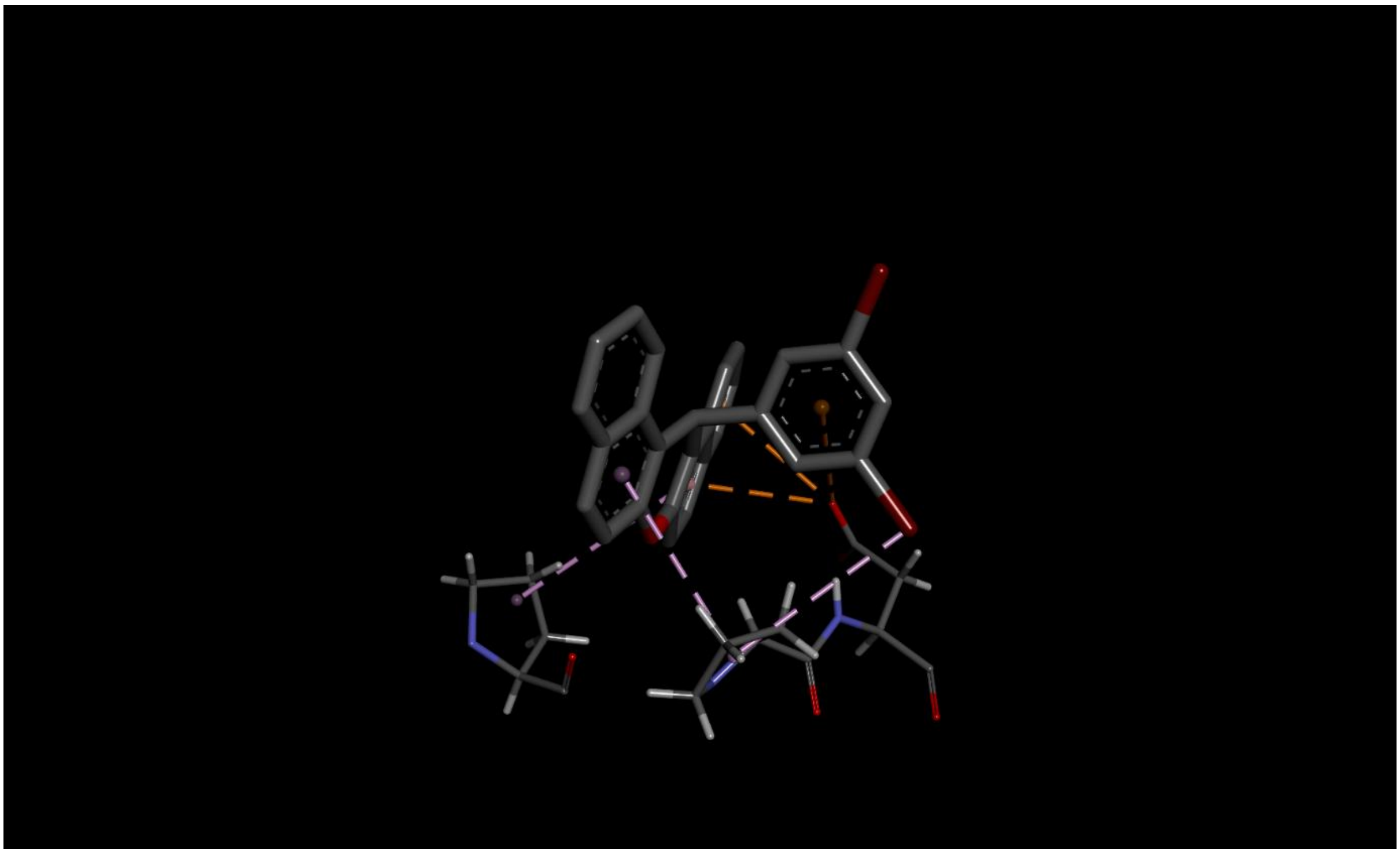

Fig. S167. Compound 6r. Binding pose of the ligand with the active site

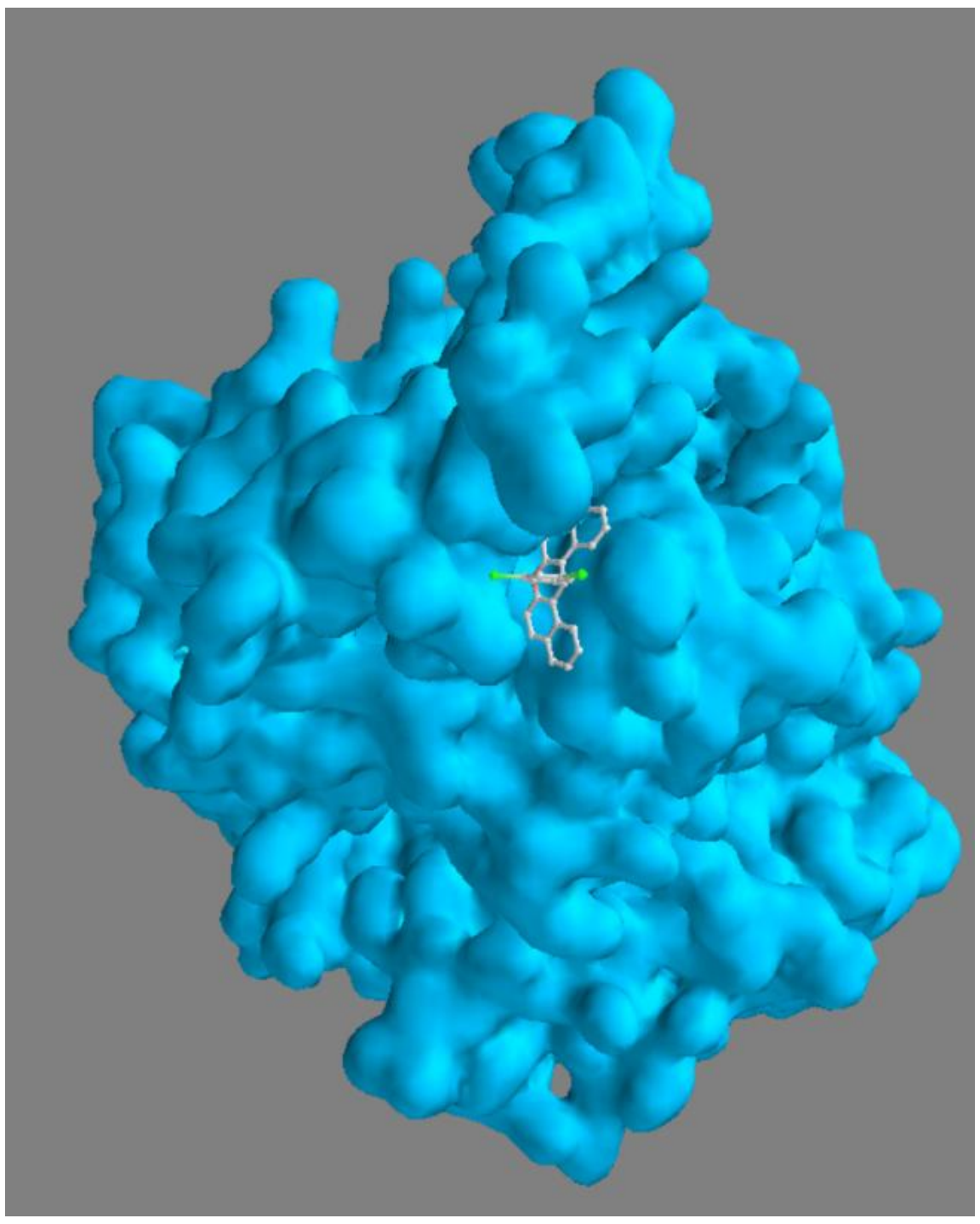

Fig. S168. Compound 6r. Protein surface visualization with bounded ligand

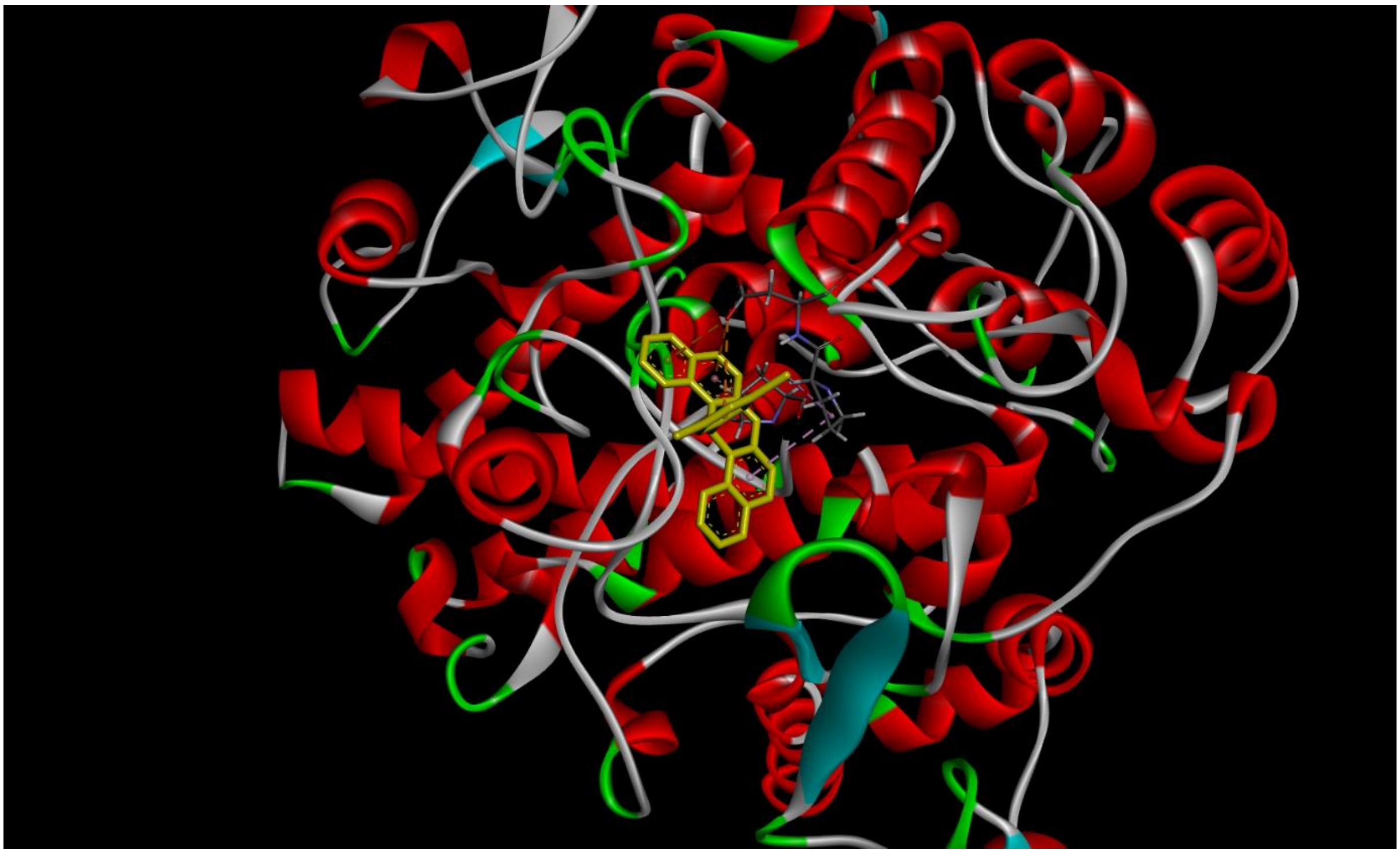

Fig. S169. Compound 6r. Ribbon diagram of the protein-ligand interaction

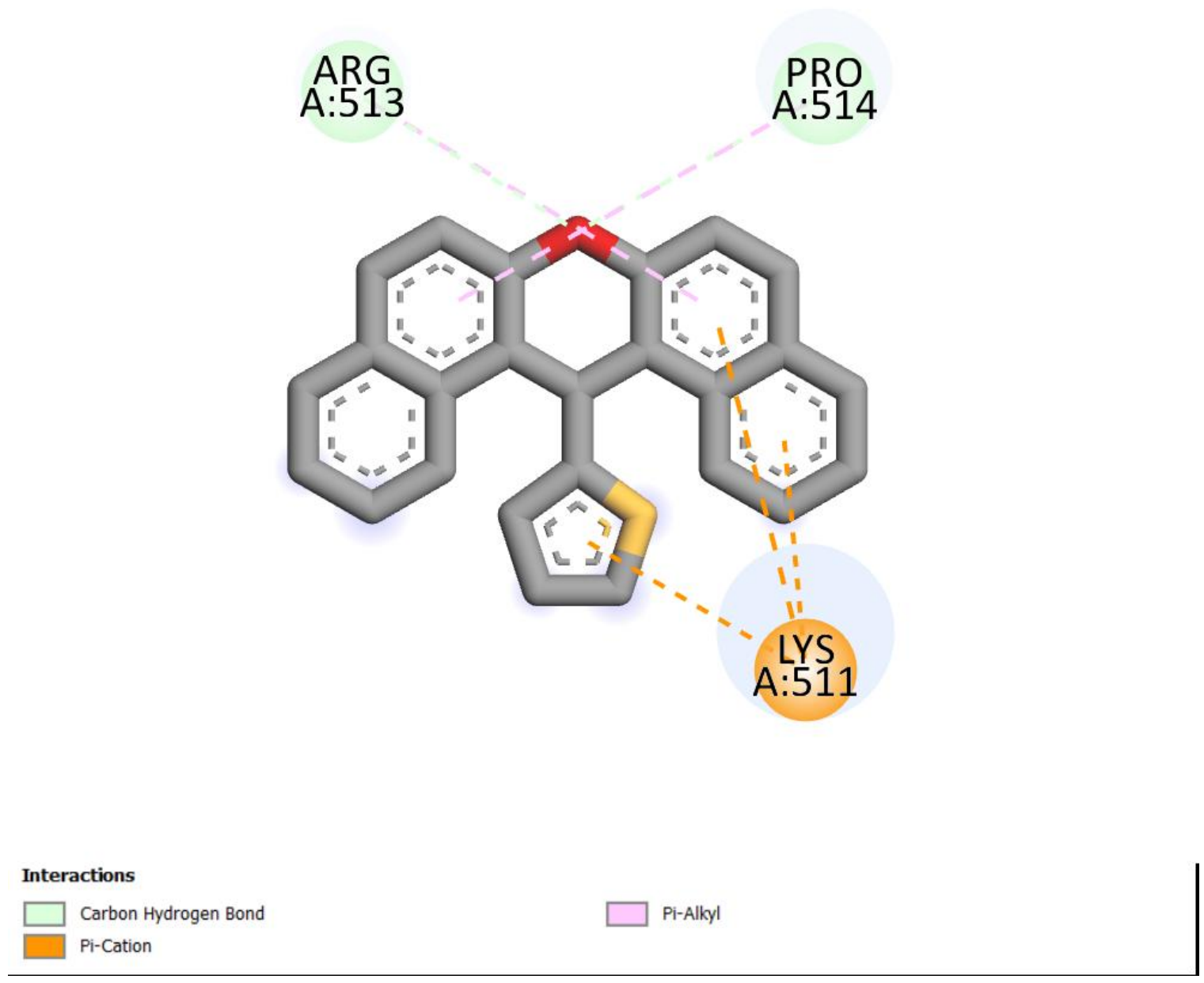

Fig. S170. Compound 6s. 2D-Interaction of ligand-protein

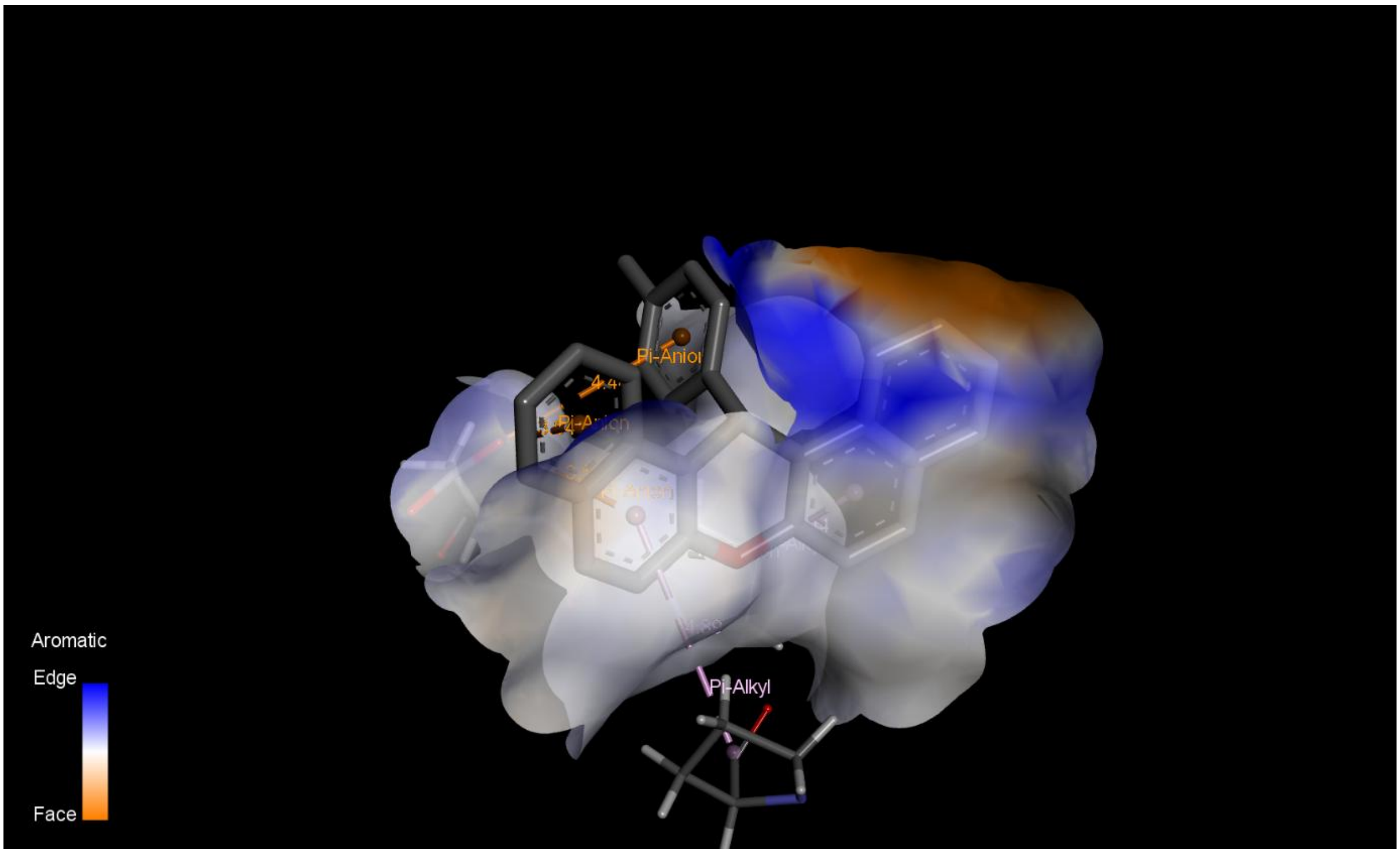

Fig. S171. Compound 6s. Visualization of aromatic face-edge interaction

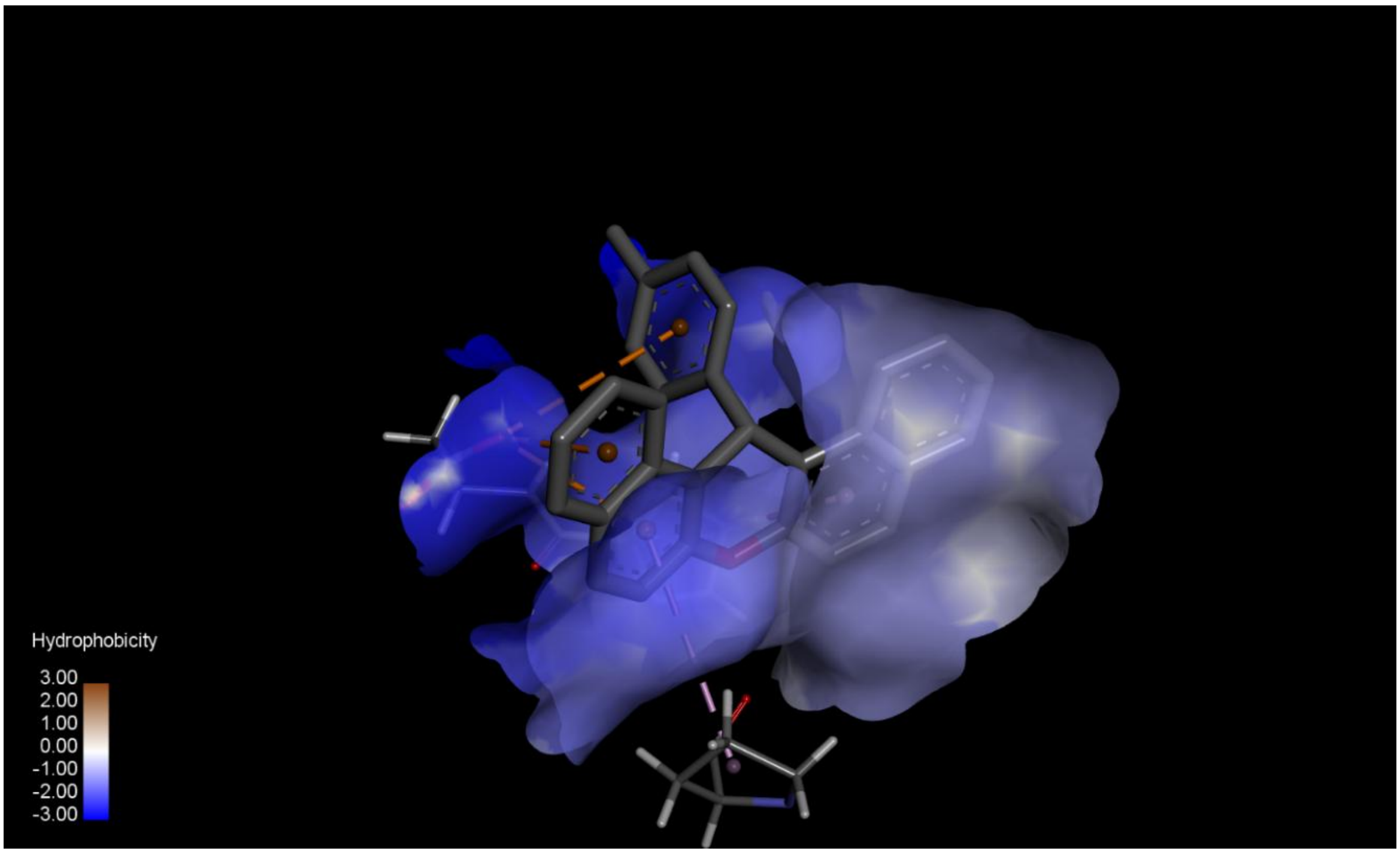

Fig. S172. Compound 6s. Visualization of hydrophobic interaction in the active site

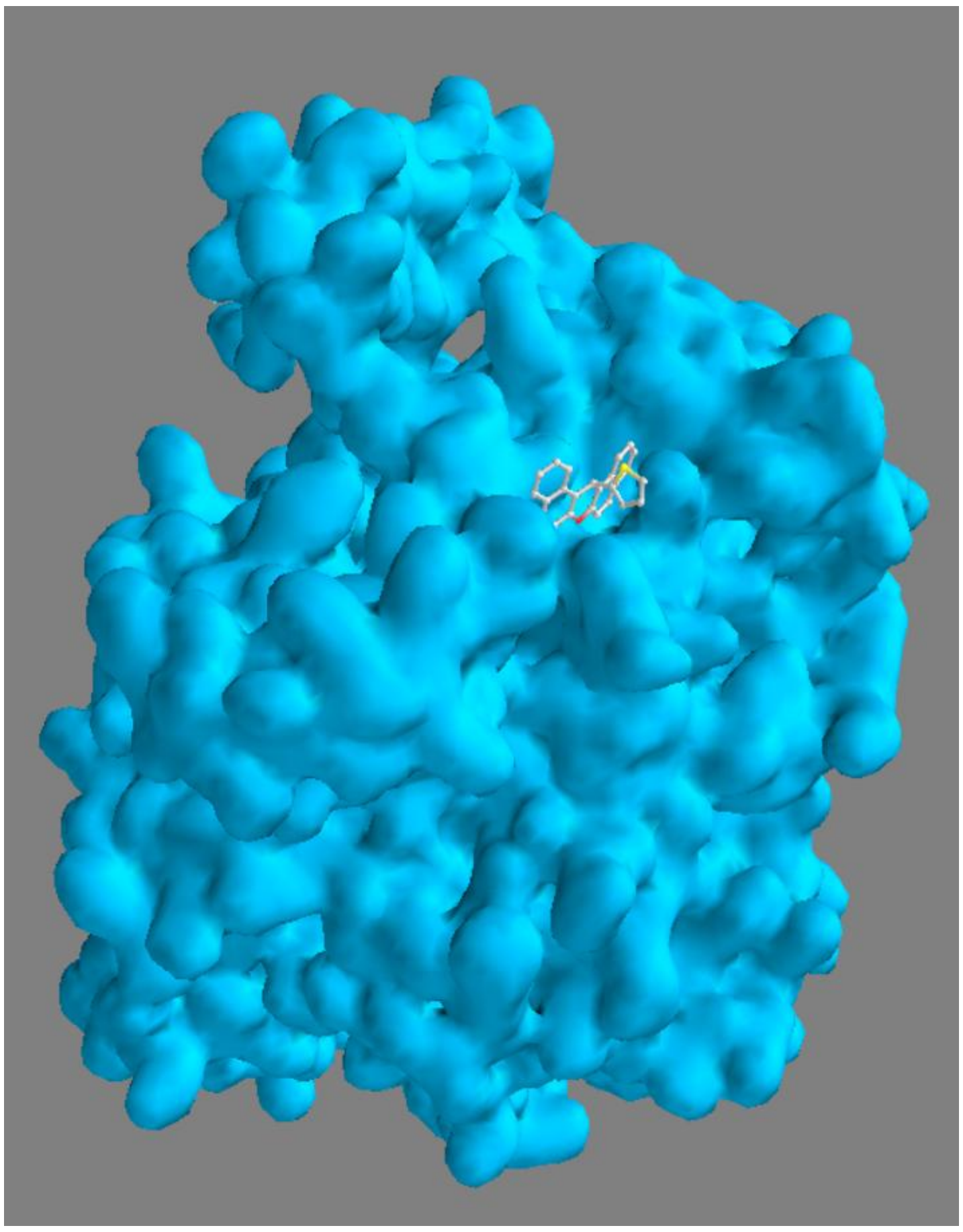

Fig. S173. Compound 6s. Protein surface visualization with bounded ligand

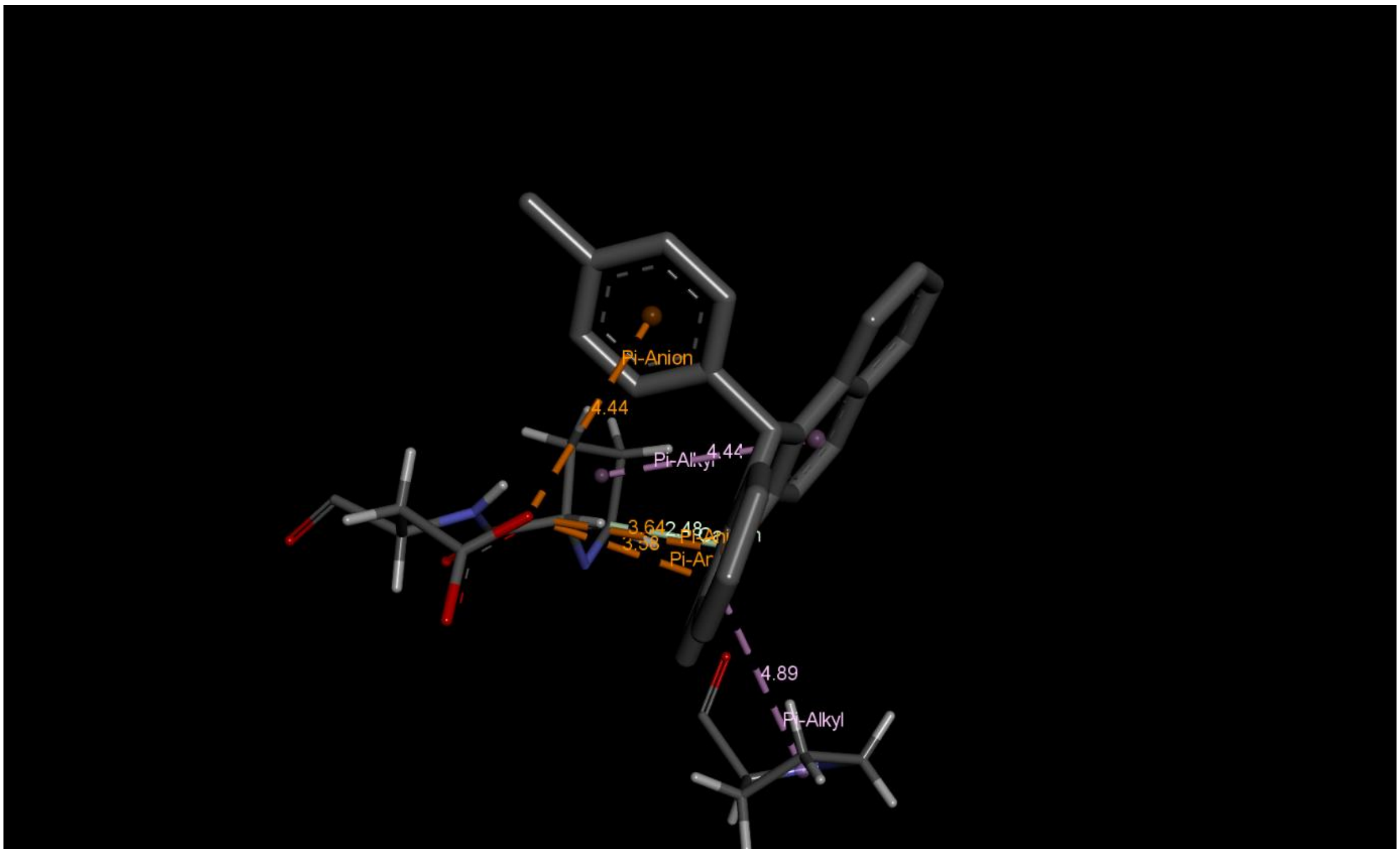

Fig. S174. Compound 6s. Binding pose of the ligand with the active site

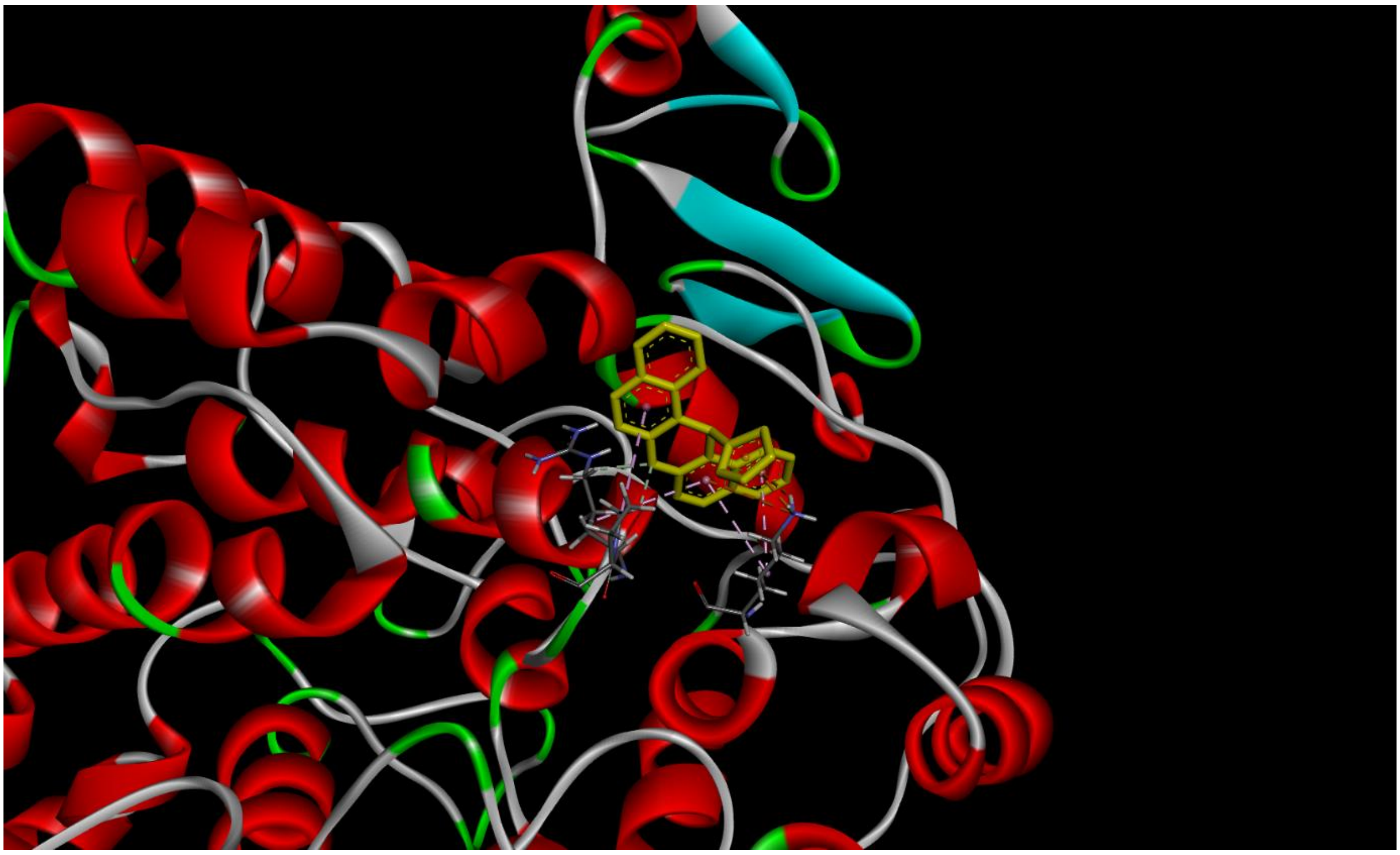

Fig. S175. Compound 6s. Ribbon diagram of the protein-ligand interaction

Table S5. Binding affinity of Cyclooxygenase-2 and ligand calculated from Molecular docking studies (kcal mol-1) using PYRX data for compound (6a-6s)

LIGAND	BINDING AFFINITY
6a	-8.7
6b	-8.6
6c	-8.6
6d	-9.0
6e	-8.5
6f	-9.0
6g	-10.1
6h	-8.6
6i	-8.8
6j	-9.1
6k	-8.7
6l	-9.3
6m	-9.0
6n	-9.7
6o	-8.7
6p	-8.6
6q	-8.0
6r	-9.1
6s	-8.2
CELECOXIB	-9.1
ROFECOXIB	-6.7

12. Crystal Structure Report for 35DB

A specimen of $C_{27}H_{16}Br_2O$, approximate dimensions 0.138 mm x 0.178 mm x 0.271 mm, was used for the X-ray crystallographic analysis. The X-ray intensity data were measured (λ = 0.71073 Å).

The integration of the data using a triclinic unit cell yielded a total of 46493 reflections to a maximum θ angle of 28.32° (0.75 Å resolution), of which 5067 were independent (average redundancy 9.176, completeness = 99.6%, R_{int} = 4.79%, R_{sig} = 3.43%) and 3583 (70.71%) were greater than $2\sigma(F^2)$. The final cell constants of a = 7.6947(4) Å, b = 10.7477(6) Å, c = 13.6192(7) Å, α = 109.084(2)°, β = 98.377(2)°, γ = 100.226(2)°, volume = 1022.06(10) $Å^3$, are based upon the refinement of the XYZ-centroids of reflections above 20 σ(I). The calculated minimum and maximum transmission coefficients (based on crystal size) are 0.4120 and 0.6090.

The structure was solved and refined using the Bruker SHELXTL Software Package, using the space group P -1, with Z = 2 for the formula unit, $C_{27}H_{16}Br_2O$. The final anisotropic full-matrix least-squares refinement on F^2 with 271 variables converged at R1 = 4.18%, for the observed data and wR2 = 11.98% for all data. The goodness-of-fit was 1.077. The largest peak in the final difference electron density synthesis was 0.842 $e^-/Å^3$ and the largest hole was -0.769 $e^-/Å^3$ with an RMS deviation of 0.077 $e^-/Å^3$. On the basis of the final model, the calculated density was 1.677 g/cm^3 and F(000), 512 e^-.

Table S6. Sample and crystal data for 35DB.

**Identification code**	**35DB**	
**Chemical formula**	**$C_{27}H_{16}Br_2O$**	
**Formula weight**	**516.22 g/mol**	
**Temperature**	**300(2) K**	
**Wavelength**	**0.71073 Å**	
**Crystal size**	**0.138 x 0.178 x 0.271 mm**	
**Crystal system**	**triclinic**	
**Space group**	**P -1**	
**Unit cell dimensions**	**a = 7.6947(4) Å**	**α = 109.084(2)°**
	**b = 10.7477(6) Å**	**β = 98.377(2)°**

	c = 13.6192(7) Å γ = 100.226(2)°
**Volume**	**1022.06(10) Å^3**
**Z**	**2**
**Density (calculated)**	**1.677 g/cm^3**
**Absorption coefficient**	**3.983 mm^{-1}**
**F(000)**	**512**

Table S7. Data collection and structure refinement for 35DB.

**Theta range for data collection**	**1.62 to 28.32°**	
**Index ranges**	**-10<=h<=10, -14<=k<=14, -18<=l<=18**	
**Reflections collected**	**46493**	
**Independent reflections**	**5067 [R(int) = 0.0479]**	
**Max. and min. transmission**	**0.6090 and 0.4120**	
**Structure solution technique**	**direct methods**	
**Structure solution program**	**SHELXT 2018/2 (Sheldrick, 2018)**	
**Refinement method**	**Full-matrix least-squares on F^2**	
**Refinement program**	**SHELXL-2019/1 (Sheldrick, 2019)**	
**Function minimized**	**Σ w(F_o^2 - F_c^2)^2**	
**Data / restraints / parameters**	**5067 / 0 / 271**	
**Goodness-of-fit on F^2**	**1.077**	
**Δ/σ_{max}**	**0.001**	
**Final R indices**	**3583 data; I>2σ(I)**	**R1 = 0.0418, wR2 = 0.1082**

	all data	R1 = 0.0689, wR2 = 0.1198
Weighting scheme	$w=1/[\sigma^2(F_o^2)+(0.0565P)^2+0.6149P]$ where $P=(F_o^2+2F_c^2)/3$	
Largest diff. peak and hole	0.842 and -0.769 eÅ^{-3}	
R.M.S. deviation from mean	0.077 eÅ^{-3}	

Table S8. Atomic coordinates and equivalent isotropic atomic displacement parameters (Å^2) for 35DB.

U(eq) is defined as one third of the trace of the orthogonalized U_{ij} tensor.

	x/a	y/b	z/c	U(eq)
Br1	0.18376(5)	0.61727(4)	0.94758(3)	0.05952(14)
Br2	0.91543(5)	0.82678(5)	0.16055(3)	0.07591(17)
O1	0.7962(2)	0.5893(2)	0.44176(16)	0.0421(5)
C1	0.4231(3)	0.7635(3)	0.4703(2)	0.0316(6)
C2	0.2466(4)	0.7592(3)	0.4215(2)	0.0392(7)
C3	0.1579(4)	0.8545(3)	0.4678(3)	0.0484(8)
C4	0.2396(5)	0.9594(3)	0.5641(3)	0.0492(8)
C5	0.4105(5)	0.9680(3)	0.6132(3)	0.0441(7)
C6	0.5063(4)	0.8711(3)	0.5687(2)	0.0349(6)
C7	0.6839(4)	0.8787(3)	0.6199(2)	0.0390(7)
C8	0.7746(4)	0.7846(3)	0.5764(2)	0.0390(7)
C9	0.6909(3)	0.6786(3)	0.4793(2)	0.0333(6)
C10	0.5211(3)	0.6664(3)	0.4246(2)	0.0308(6)
C11	0.7117(4)	0.4668(3)	0.3622(2)	0.0357(6)

	x/a	y/b	z/c	U(eq)
**C12**	**0.5397(4)**	**0.4413(3)**	**0.3038(2)**	**0.0321(6)**
**C13**	**0.4469(3)**	**0.5569(3)**	**0.3156(2)**	**0.0303(6)**
**C14**	**0.8173(4)**	**0.3702(3)**	**0.3443(2)**	**0.0428(7)**
**C15**	**0.7457(4)**	**0.2463(3)**	**0.2686(2)**	**0.0436(7)**
**C16**	**0.5640(4)**	**0.2090(3)**	**0.2109(2)**	**0.0399(7)**
**C17**	**0.4597(4)**	**0.3079(3)**	**0.2290(2)**	**0.0342(6)**
**C18**	**0.4830(5)**	**0.0766(3)**	**0.1384(3)**	**0.0521(8)**
**C19**	**0.3065(6)**	**0.0397(3)**	**0.0871(3)**	**0.0572(9)**
**C20**	**0.2024(5)**	**0.1357(3)**	**0.1051(3)**	**0.0523(8)**
**C21**	**0.2759(4)**	**0.2660(3)**	**0.1727(2)**	**0.0400(7)**
**C22**	**0.4750(3)**	**0.6128(3)**	**0.2276(2)**	**0.0301(6)**
**C23**	**0.3353(4)**	**0.5954(3)**	**0.1438(2)**	**0.0348(6)**
**C24**	**0.3724(4)**	**0.6445(3)**	**0.0648(2)**	**0.0361(6)**
**C25**	**0.5422(4)**	**0.7124(3)**	**0.0667(2)**	**0.0392(7)**
**C26**	**0.6785(4)**	**0.7304(3)**	**0.1517(2)**	**0.0393(7)**
**C27**	**0.6484(4)**	**0.6817(3)**	**0.2311(2)**	**0.0350(6)**

Table S9. Bond lengths (Å) for 35DB.

**Br1-C24**	**1.900(3)**	**Br2-C26**	**1.896(3)**
**O1-C11**	**1.374(3)**	**O1-C9**	**1.384(3)**
**C1-C2**	**1.408(4)**	**C1-C6**	**1.420(4)**
**C1-C10**	**1.426(4)**	**C2-C3**	**1.366(4)**
**C3-C4**	**1.388(5)**	**C4-C5**	**1.358(5)**
**C5-C6**	**1.409(4)**	**C6-C7**	**1.416(4)**
**C7-C8**	**1.357(4)**	**C8-C9**	**1.403(4)**

**C9-C10**	**1.368(4)**	**C10-C13**	**1.512(4)**
**C11-C12**	**1.372(4)**	**C11-C14**	**1.411(4)**
**C12-C17**	**1.422(4)**	**C12-C13**	**1.516(4)**
**C13-C22**	**1.531(4)**	**C14-C15**	**1.348(4)**
**C15-C16**	**1.417(4)**	**C16-C18**	**1.406(4)**
**C16-C17**	**1.423(4)**	**C17-C21**	**1.422(4)**
**C18-C19**	**1.356(5)**	**C19-C20**	**1.397(5)**
**C20-C21**	**1.361(4)**	**C22-C23**	**1.387(4)**
**C22-C27**	**1.392(4)**	**C23-C24**	**1.388(4)**
**C24-C25**	**1.371(4)**	**C25-C26**	**1.381(4)**
**C26-C27**	**1.377(4)**		

Table S10. Bond angles (°) for 35DB.

**C11-O1-C9**	**117.9(2)**	**C2-C1-C6**	**117.7(2)**
**C2-C1-C10**	**123.0(3)**	**C6-C1-C10**	**119.2(2)**
**C3-C2-C1**	**121.1(3)**	**C2-C3-C4**	**121.0(3)**
**C5-C4-C3**	**119.8(3)**	**C4-C5-C6**	**121.1(3)**
**C5-C6-C7**	**121.4(3)**	**C5-C6-C1**	**119.3(3)**
**C7-C6-C1**	**119.3(2)**	**C8-C7-C6**	**120.9(3)**
**C7-C8-C9**	**119.4(3)**	**C10-C9-O1**	**122.7(2)**
**C10-C9-C8**	**122.8(3)**	**O1-C9-C8**	**114.5(2)**
**C9-C10-C1**	**118.4(2)**	**C9-C10-C13**	**119.7(2)**
**C1-C10-C13**	**121.8(2)**	**C12-C11-O1**	**122.7(2)**
**C12-C11-C14**	**122.7(3)**	**O1-C11-C14**	**114.5(2)**
**C11-C12-C17**	**118.1(2)**	**C11-C12-C13**	**119.4(2)**
**C17-C12-C13**	**122.4(2)**	**C10-C13-C12**	**110.2(2)**

**C10-C13-C22**	**111.1(2)**	**C12-C13-C22**	**108.9(2)**
**C15-C14-C11**	**119.0(3)**	**C14-C15-C16**	**121.4(3)**
**C18-C16-C15**	**121.9(3)**	**C18-C16-C17**	**119.2(3)**
**C15-C16-C17**	**118.9(3)**	**C12-C17-C21**	**123.0(3)**
**C12-C17-C16**	**119.5(3)**	**C21-C17-C16**	**117.5(3)**
**C19-C18-C16**	**121.7(3)**	**C18-C19-C20**	**119.5(3)**
**C21-C20-C19**	**120.9(3)**	**C20-C21-C17**	**121.1(3)**
**C23-C22-C27**	**118.9(3)**	**C23-C22-C13**	**122.6(2)**
**C27-C22-C13**	**118.5(2)**	**C22-C23-C24**	**119.3(3)**
**C25-C24-C23**	**122.7(3)**	**C25-C24-Br1**	**117.5(2)**
**C23-C24-Br1**	**119.9(2)**	**C24-C25-C26**	**117.0(3)**
**C27-C26-C25**	**122.3(3)**	**C27-C26-Br2**	**118.6(2)**
**C25-C26-Br2**	**119.0(2)**	**C26-C27-C22**	**119.8(3)**

Table S11. Anisotropic atomic displacement parameters (Å^2) for 35DB.

The anisotropic atomic displacement factor exponent takes the form: $-2\pi^2[h^2 a^{*2} U_{11} + ... + 2 h k a^{*} b^{*} U_{12}]$

	U_{11}	U_{22}	U_{33}	U_{23}	U_{13}	U_{12}
**Br1**	**0.0509(2)**	**0.0770(3)**	**0.0483(2)**	**0.03471(19)**	**-0.00962(15)**	**0.00315(17)**
**Br2**	**0.0434(2)**	**0.1132(4)**	**0.0636(3)**	**0.0437(3)**	**0.00679(17)**	**-0.0181(2)**
**O1**	**0.0286(10)**	**0.0417(12)**	**0.0461(12)**	**0.0068(10)**	**-0.0024(9)**	**0.0104(9)**
**C1**	**0.0311(13)**	**0.0358(15)**	**0.0342(14)**	**0.0191(12)**	**0.0110(11)**	**0.0080(11)**
**C2**	**0.0317(14)**	**0.0432(17)**	**0.0426(16)**	**0.0168(14)**	**0.0057(12)**	**0.0083(12)**
**C3**	**0.0377(16)**	**0.057(2)**	**0.059(2)**	**0.0265(17)**	**0.0118(15)**	**0.0190(15)**
**C4**	**0.055(2)**	**0.0512(19)**	**0.057(2)**	**0.0263(17)**	**0.0251(17)**	**0.0285(16)**
**C5**	**0.060(2)**	**0.0427(17)**	**0.0373(16)**	**0.0181(14)**	**0.0177(14)**	**0.0185(15)**

	U_{11}	U_{22}	U_{33}	U_{23}	U_{13}	U_{12}
**C6**	**0.0388(15)**	**0.0379(15)**	**0.0318(14)**	**0.0165(12)**	**0.0119(12)**	**0.0081(12)**
**C7**	**0.0434(16)**	**0.0406(16)**	**0.0265(14)**	**0.0086(12)**	**0.0034(12)**	**0.0040(13)**
**C8**	**0.0326(14)**	**0.0473(17)**	**0.0336(15)**	**0.0159(13)**	**-0.0007(12)**	**0.0048(13)**
**C9**	**0.0280(13)**	**0.0367(15)**	**0.0359(15)**	**0.0152(12)**	**0.0045(11)**	**0.0072(11)**
**C10**	**0.0290(13)**	**0.0352(14)**	**0.0296(13)**	**0.0148(11)**	**0.0059(10)**	**0.0061(11)**
**C11**	**0.0339(14)**	**0.0373(16)**	**0.0375(15)**	**0.0153(13)**	**0.0063(12)**	**0.0106(12)**
**C12**	**0.0330(13)**	**0.0322(14)**	**0.0335(14)**	**0.0163(12)**	**0.0070(11)**	**0.0052(11)**
**C13**	**0.0228(12)**	**0.0349(14)**	**0.0329(14)**	**0.0145(12)**	**0.0022(10)**	**0.0051(10)**
**C14**	**0.0376(15)**	**0.0479(18)**	**0.0485(18)**	**0.0230(15)**	**0.0057(13)**	**0.0169(13)**
**C15**	**0.0512(18)**	**0.0444(18)**	**0.0448(17)**	**0.0209(15)**	**0.0139(14)**	**0.0234(15)**
**C16**	**0.0569(19)**	**0.0351(16)**	**0.0324(15)**	**0.0163(13)**	**0.0145(13)**	**0.0115(14)**
**C17**	**0.0431(15)**	**0.0331(15)**	**0.0293(14)**	**0.0171(12)**	**0.0081(12)**	**0.0054(12)**
**C18**	**0.076(2)**	**0.0399(18)**	**0.0427(18)**	**0.0142(15)**	**0.0176(17)**	**0.0177(17)**
**C19**	**0.079(3)**	**0.0343(18)**	**0.0418(19)**	**0.0043(15)**	**0.0051(17)**	**-0.0014(17)**
**C20**	**0.058(2)**	**0.0462(19)**	**0.0408(18)**	**0.0135(15)**	**-0.0013(15)**	**-0.0040(16)**
**C21**	**0.0443(16)**	**0.0353(16)**	**0.0367(16)**	**0.0149(13)**	**0.0022(13)**	**0.0026(13)**
**C22**	**0.0307(13)**	**0.0263(13)**	**0.0311(14)**	**0.0083(11)**	**0.0040(11)**	**0.0079(11)**
**C23**	**0.0304(13)**	**0.0335(15)**	**0.0381(15)**	**0.0147(12)**	**0.0022(11)**	**0.0024(11)**
**C24**	**0.0357(14)**	**0.0334(15)**	**0.0348(15)**	**0.0123(12)**	**-0.0031(12)**	**0.0065(12)**
**C25**	**0.0456(17)**	**0.0390(16)**	**0.0338(15)**	**0.0154(13)**	**0.0095(13)**	**0.0072(13)**
**C26**	**0.0311(14)**	**0.0437(17)**	**0.0386(16)**	**0.0136(13)**	**0.0088(12)**	**-0.0006(12)**
**C27**	**0.0283(13)**	**0.0405(16)**	**0.0326(14)**	**0.0125(12)**	**0.0021(11)**	**0.0046(11)**

Table S12. Hydrogen atomic coordinates and isotropic atomic displacement parameters (Å^2) for 35DB.

	x/a	y/b	z/c	U(eq)
H2	0.1895	0.6903	0.3566	0.047000
H3	0.0410	0.8491	0.4344	0.058000
H4	0.1776	1.0236	0.5948	0.059000
H5	0.4651	1.0390	0.6773	0.053000
H7	0.7392	0.9493	0.6842	0.047000
H8	0.8910	0.7902	0.6107	0.047000
H13	0.3169	0.5220	0.3075	0.036000
H14	0.9347	0.3916	0.3842	0.051000
H15	0.8170	0.1839	0.2539	0.052000
H18	0.5521	0.0126	0.1252	0.062000
H19	0.2552	-0.0489	0.0403	0.069000
H20	0.0812	0.1103	0.0704	0.063000
H21	0.2048	0.3286	0.1823	0.048000
H23	0.2180	0.5512	0.1407	0.042000
H25	0.5645	0.7448	0.0131	0.047000
H27	0.7436	0.6947	0.2869	0.042000

13. Determination of Hammett Acidity (H_0) of the Synthesized Ionic Liquid Using UV–Vis Spectroscopy

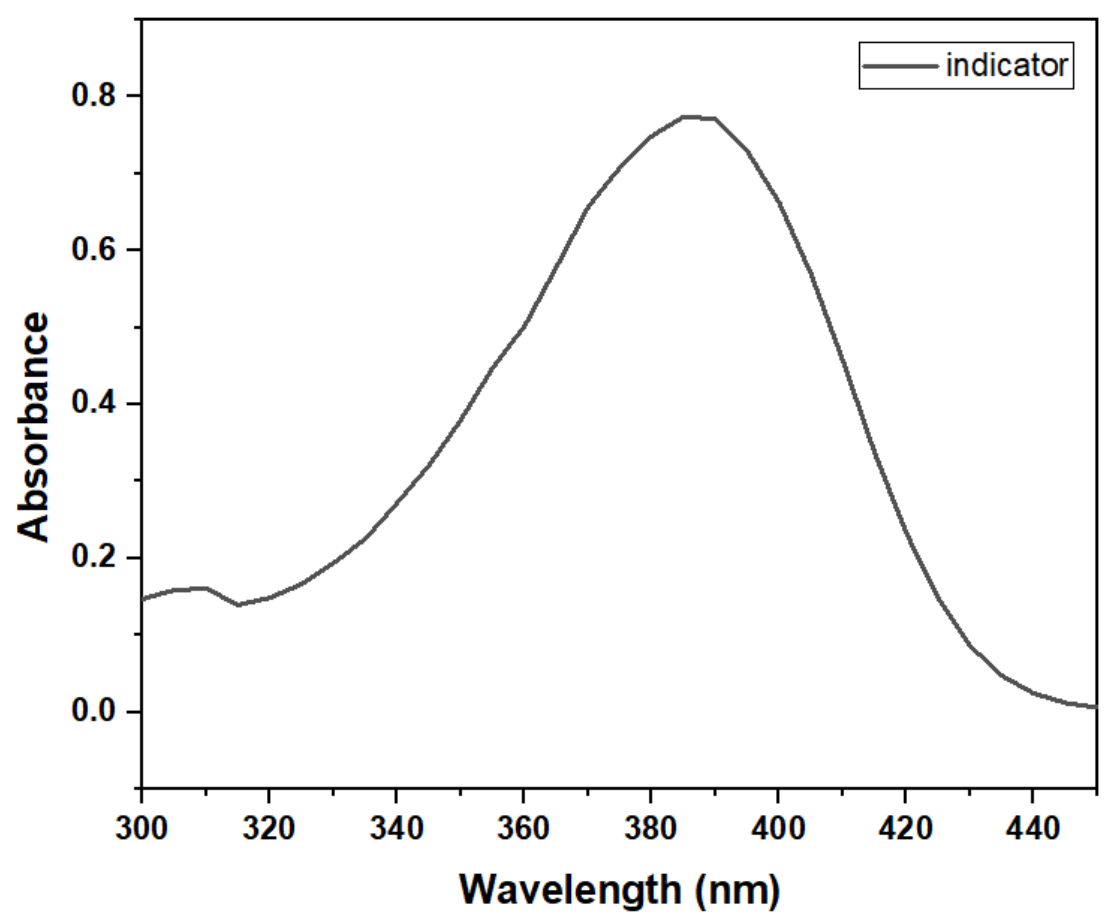

Figure S176. UV–Vis absorption spectrum of p-nitroaniline indicator in DMSO

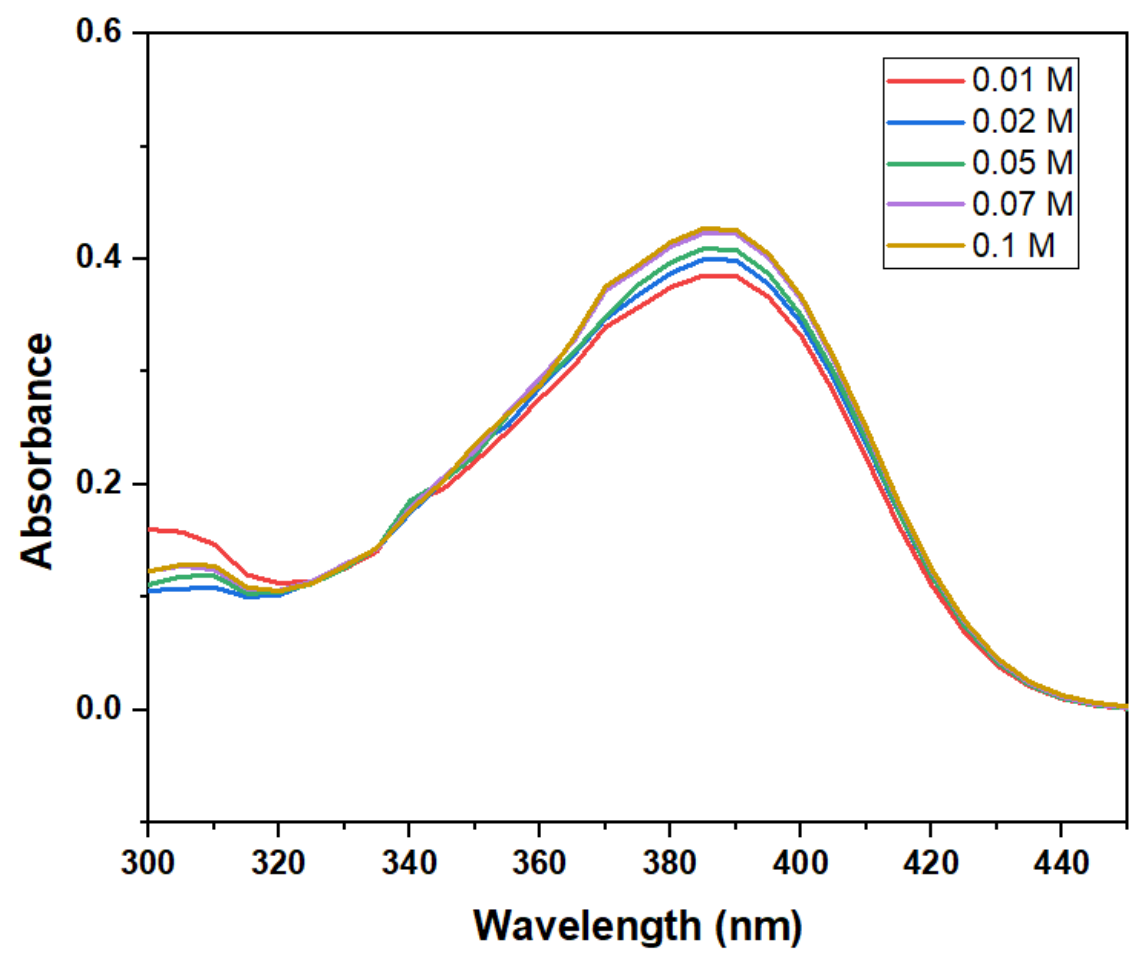

Figure S177. UV–Vis absorption spectra of p-nitroaniline in the presence of different concentrations of the ionic liquid (0.01–0.10 M) in DMSO. [9]

Table S13. Absorbance values and calculated Hammett acidity function (H_0) for different concentrations of the ionic liquid.

Entry	Concentration	Absorbance	[I]%	$[IH^+]$	H_0^a
**1**	0.01	0.3847	9.90	90.09	1.09
**2**	0.02	0.3989	6.58	93.41	1.07
**3**	0.05	0.4093	4.14	95.85	1.04
**4**	0.07	0.4223	1.10	98.89	1.01
**5**	0.1	0.4278	0	100	-

14. Comparison with Reported Catalysts

Several catalysts have been reported for the synthesis of dibenzoxanthene derivatives. To evaluate the efficiency of **[VSBIM][Cl]**, its catalytic performance was compared with previously reported catalysts. The comparison in **Table S14** is based on catalyst loading, reaction time, yield, and recyclability.

Table: S14 Comparison of **[VSBIM][Cl]** with other works for 14-phenyl-14*H*-dibenzo[a,j]xanthene synthesis.

Entry	Catalyst	Catalyst loading (mol%)	Time	Tempe rature	Yield (%)	Recyclability
1	Sulfamic acid	10	6-12 h	125	90-95	no[10]
2	Amberlyst-15	-	90-120 min	125	94-89	No[11]
3	$FeCl_3$	-	10 h	100	91	No[12]
4	I_2	25	20 min	90-95	90	No[13]
5	([Msim]Cl)	15	30 min	120	93	Yes[14]
6	$[Et_3N\text{-}SO_3H]$ Cl	15	15-40 min	120	96-97	Yes[15]
7	$[DBU]_2$[EDS]	40	5 min	120	92	Yes[16]
8	[bmim]$[HSO_4]$	5	30 min	100	85	Yes[17]
**9**	**[VSBIM][Cl] (This work)**	**5**	**40 min**	**110**	**95**	**5 Cycles**

14. Green chemistry metrics calculation

Here, we have calculated different parameters of the green chemistry metrics[18]. The parameters are as follows:

1) E-factor or environmental factor
2) Atom economy (AE)
3) Atom efficiency
4) Carbon efficiency
5) Product mass intensity (PMI)
6) Reaction mass efficiency (RME)

Calculation of Green Chemistry Metrics for the Synthesis of Xanthene Derivatives

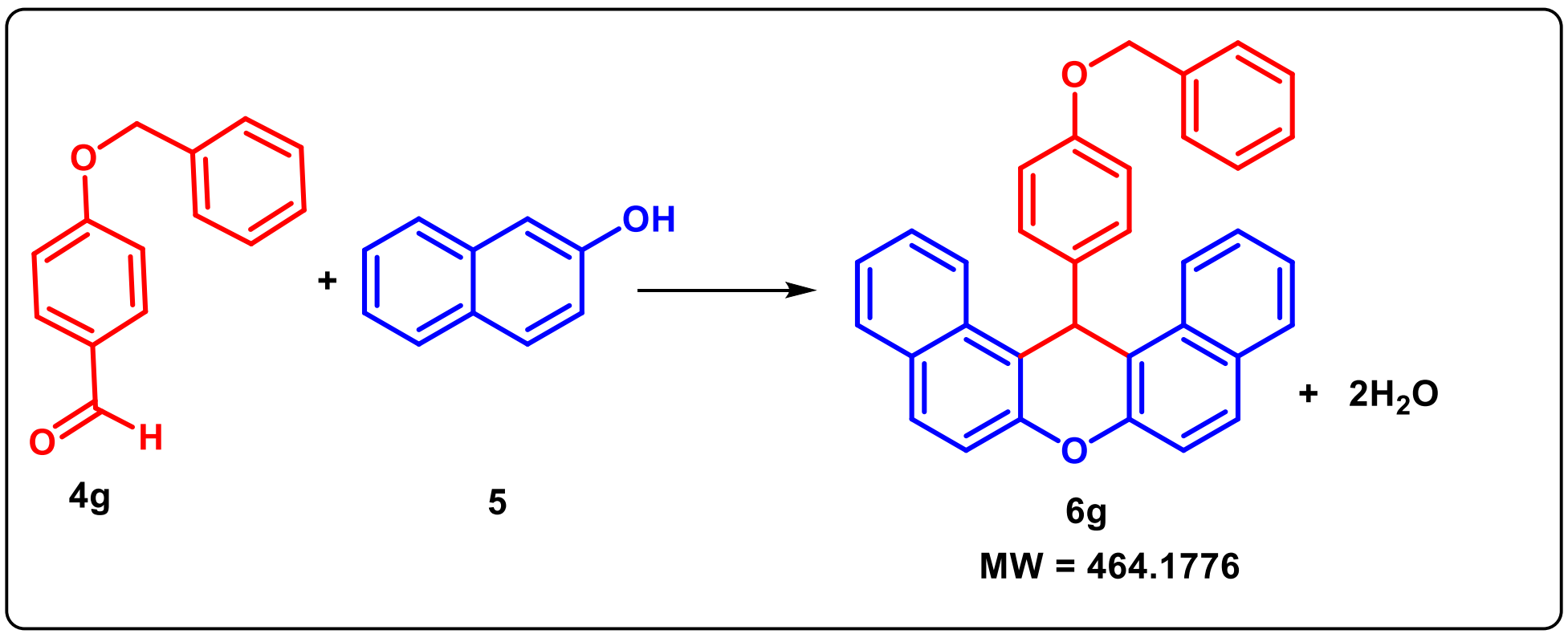

MW	212.0837	144.17 g/mol
Amount	0.7 mmol	1.4 mmol

	Name	Chemical formula	Molecular weight	Millimole	mg
Reactant 1	4-Benzyloxybenzaldehyde	$C_{14}H_{12}O_2$	212.0837	0.7	150
Reactant 2	β-naphthol	$C_{10}H_8O$	144.17	1.4	204
Product	14-(4-(benzyloxy)phenyl)-14H-dibenzo[a,j]xanthene	$C_{34}H_{24}O_2$	464.1776	-	312

Yield of product: 95%.

E-factor or environmental factor: The E-factor measures the mass of waste generated per unit mass of product, and its ideal value is zero.

$$Efactor = \frac{Total\ mass\ of\ the\ waste}{Mass\ of\ the\ product}$$

$$Where\ total\ mass\ of\ waste$$
$$= total\ mass\ of\ raw\ materials\ - the\ total\ mass\ of\ product$$

$$E\ factor = \frac{\{(212.0837 \times 0.71) + (144.17 \times 1.416) - 312\}\ mg}{312}$$

$E\text{-}factor = 0.13$

Atom economy (AE): Atom economy (AE) measures the percentage of reactant atoms present in the final product. The ideal value of AE is 100%.

$$Atom\ economy = \frac{MW\ of\ product}{\Sigma\ (MW\ of\ reactants)} \times 100$$

$$= \frac{464.17}{212.08 + 2 \times 144.17} = 92.76\%$$

$$Atom\ efficiency = \frac{\%\ yield\ of\ product\ \times\ \%\ atom\ economy}{100}$$

$$= \frac{95 \times 92.76}{100} = 88.122\%$$

$$Carbon\ efficiency = \frac{No\ of\ carbon\ atoms\ in\ product}{\Sigma(No\ of\ carbon\ atoms\ in\ reactants)} \times 100$$

$$= \frac{34}{14 + 20} \times 100 = 100\%$$

Product mass intensity (PMI): PMI measures the total mass of input materials (reactants), including solvents, used in a chemical reaction per unit mass of product.

$$PMI = \frac{\Sigma(Mass\ of\ reactants\ including\ solvent)}{Mass\ of\ the\ product}$$

$$= \frac{(212.08 \times 0.71) + (144.17 \times 1.41)}{321}$$

$$= 1.15$$

Ideal value of PMI = **E-factor + 1**
Here, PMI = 0.136+1 **= 1.136**

Reaction mass efficiency (RME): RME is a mass-based metric that measures the percentage of reactant mass incorporated into the final product. It is defined as the mass of the product divided by the total mass of stoichiometric reactants. The value of RME ranges from 0 to 100%, and higher RME values indicate a greener reaction

$$RME = \frac{Mass\ of\ product}{\Sigma\ (mass\ of\ reactants)} \times 100$$

$$= \frac{312}{(212.08 \times 0.71) + (144.17 \times 1.41)} \times 100$$

$$= 88.17\%$$

Table-S15 Summary of green metric parameters:

**E- factor**	0.136
**Atom economy (AE)**	92.76%
**Atom efficiency**	88.12%
**Carbon efficiency**	100%
**PMI**	1.15
**RME**	88.17

(1) Pasuparthy, S. D.; Somkuwar, P.; Kali, V.; Somanahalli Kalleshappa, A. K.; Maiti, B. Synthesis of Dimeric Indoles from Friedel–Crafts Reaction of Indoles with Ketones Catalysed by a Brønsted Acid Ionic Liquid and Their Interactions with BSA and DNA. *New Journal of Chemistry* **2024**, *48* (34), 14904–14923.

(2) Mohurle, S.; C, V. D.; Pasuparthy, S. D.; Talamarlla, D.; Kali, V.; Maiti, B. [BCMIM][Cl] Ionic Liquid Catalyzed Diastereoselective Synthesis of β-Amino Ketones via Facile, One-pot, Multicomponent Mannich Reaction under Solvent-free Condition. *J. Heterocycl. Chem.* **2023**, *60* (9), 1545–1557.

(3) Pasuparthy, S. D.; Maiti, B. Facile Synthesis of Bis (Indol-3-Yl) Methane Derivatives Catalyzed by Carboxylic Acid Functionalized Ionic Liquid at Room Temperature: Investigation of Photophysical Properties, DFT Calculations and Molecular Docking with Bovine Serum Albumin. *Tetrahedron* **2024**, *153*, 133845.

(4) Deepak Pasuparthy, S.; Joseph, S.; Somkuwar, P.; S. K., A. K.; Maiti, B. Facile Synthesis of Bis-Coumarins Using [BCMIM][Cl] Zwitter Ionic Liquid and Their Binding with Bovine Serum Albumin. *Asian J. Org. Chem.* **2023**, *12* (8).

(5) Daina, A.; Michielin, O.; Zoete, V. SwissADME: A Free Web Tool to Evaluate Pharmacokinetics, Drug-Likeness and Medicinal Chemistry Friendliness of Small Molecules. *Sci. Rep.* **2017**, *7* (1), 42717.

(6) Pires, D. E. V.; Blundell, T. L.; Ascher, D. B. PkCSM: Predicting Small-Molecule Pharmacokinetic and Toxicity Properties Using Graph-Based Signatures. *J. Med. Chem.* **2015**, *58* (9), 4066–4072.

(7) Lipinski, C. A.; Lombardo, F.; Dominy, B. W.; Feeney, P. J. Experimental and Computational Approaches to Estimate Solubility and Permeability in Drug Discovery and Development Settings. *Adv. Drug Deliv. Rev.* **2001**, *46* (1–3), 3–26.

(8) Kaur, G.; Kumar, M. AVCPred: An Integrated Web Server for Prediction and Design of Antiviral Compounds. *Chem. Biol. Drug Des.* **2017**, *89* (1), 74–83.

(9) Wei, C.; Liu, Z.; Tan, H.; Huang, L.; Li, J. A Non-Metal Route to Realize the Bio-Based Polyester of Poly (Hexylene Succinate): Preparation Conditions, Side-Reactions and Mechanism in Sulfonic Acid-Functionalized Brønsted Acidic Ionic Liquids. *RSC Adv.* **2020**, *10* (58), 35381–35388.

(10) Khodaei, M.; Khosropour, A.; Moghanian, H. A Simple and Efficient Procedure for the Synthesis of Amidoalkyl Naphthols by *p* -TSA in Solution or under Solvent-Free Conditions. *Synlett* **2006**, *2006* (06), 916–920.

(11) Ko, S.; Yao, C.-F. Heterogeneous Catalyst: Amberlyst-15 Catalyzes the Synthesis of 14-Substituted-14H-Dibenzo [a, j] Xanthenes under Solvent-Free Conditions. *Tetrahedron Lett.* **2006**, *47* (50), 8827–8829.

(12) Wang, B.; Li, P.; Zhang, Y.; Wang, L. $FeCl_3$ -Catalyzed Condensation of 2-Naphthol and Aldehydes under Solvent-Free Reaction Conditions: An Efficient and Green Alternative for the Synthesis of 14-Aryl (Alkyl)-14- *H* -dibenzo [*a*, *j*] Xanthenes. *Chin. J. Chem.* **2010**, *28* (12), 2463–2468.

(13) Pasha, M. A.; Jayashankara, V. P. Molecular Iodine Catalyzed Synthesis of Aryl-14H-Dibenzo [a, j] Xanthenes under Solvent-Free Condition. *Bioorg. Med. Chem. Lett.* **2007**, *17* (3), 621–623.

(14) Huynh, C. C.; Tran, P. H.; Le, T. N. Brønsted Acidic Ionic Liquid-Catalyzed Synthesis of 14-Aryl-14H-Dibenzo [a, j] Xanthenes with Controlled Microwave Heating under Solvent-Free Conditions. *Russian Journal of Organic Chemistry* **2020**, *56* (3), 504–508.

(15) Zare, A.; Moosavi-Zare, A. R.; Merajoddin, M.; Zolfigol, M. A.; Hekmat-Zadeh, T.; Hasaninejad, A.; Khazaei, A.; Mokhlesi, M.; Khakyzadeh, V.; Derakhshan-Panah, F.; Beyzavi, M. H.; Rostami, E.; Arghoon, A.; Roohandeh, R. Ionic Liquid Triethylamine-Bonded Sulfonic Acid {[$Et_3N–SO_3H$]Cl} as a Novel, Highly Efficient and Homogeneous Catalyst for the Synthesis of β-Acetamido Ketones, 1,8-Dioxo-Octahydroxanthenes and 14-Aryl-14H-Dibenzo[a,j]Xanthenes. *J. Mol. Liq.* **2012**, *167*, 69–77.

(16) Maleki, B.; Akbarzadeh, E.; Babaee, S. New Basic Ionic Liquid from Ethan-1,2-Diyl Bis (Hydrogen Sulfate) and DBU (1,8-Diazobicyclo [5.4.0] Undec-7-Ene) as an Efficient Catalyst for One-Pot Synthesis of Xanthene Derivatives. *Dyes and Pigments* **2015**, *123*, 222–234.

(17) Fang, D.; Liu, Z. Synthesis of 14-aryl-14 *H* -dibenzo [*a, j*] Xanthenes Catalyzed by Acyclic Acidic Ionic Liquids. *J. Heterocycl. Chem.* **2010**, *47* (3), 509–512.

(18) Mahapatra, J.; Dastidar, S. G.; Jagankar, D.; Roy, N.; Sharma, J.; Mukherjee, A.; Maity, C.; Panda, T. K.; Mal, S. S. Catalytic Synthesis of Xanthene and Unprecedented Evolution of Naphthopyrans Using Heteropoly Acid-Tantalum(V) Oxide Hybrid Composite as Promoter. *ChemistrySelect* **2025**, *10* (3).
